# Global-scale modeling of early factors and country-specific trajectories of COVID-19 incidence: a cross-sectional study of the first 6 months of the pandemic

**DOI:** 10.1186/s12889-022-14336-w

**Published:** 2022-10-14

**Authors:** Sujoy Ghosh, Saikat Sinha Roy

**Affiliations:** 1 Centre for Computational Biology & Program in Cardiovascular and Metabolic Disorders, Duke-NUS Medical School, Singapore, Singapore; 2 Laboratory of Computational Biology, Penninton Biomedical Research Center, Baton Rouge, LA USA; 3 Department of Economics, Jadavpur University, Kolkata, West Bengal India

**Keywords:** COVID-19, Global, Regression, Factors, Lockdown, Modeling, Clustering

## Abstract

**Background:**

Studies examining factors responsible for COVID-19 incidence have been mostly focused at the national or sub-national level. A global-level characterization of contributing factors and temporal trajectories of disease incidence is currently lacking. Here we conducted a global-scale analysis of COVID-19 infections to identify key factors associated with early disease incidence. Additionally, we compared longitudinal trends of COVID-19 incidence at a per-country level, and classified countries based on COVID-19 incidence trajectories and effects of lockdown responses.

**Methods:**

This is an observational cross-sectional study covering COVID-19 incidence over the first 6 months of the pandemic (Jan 1, 2020 to June 30, 2020). A retrospective analysis was performed using publicly available data for total confirmed COVID-19 cases by country, and using recent data on demographic, meteorological, economic and health-related indicators per country. Data was analyzed in a regression modeling framework. Longitudinal trends were assessed via linear and non-linear model fitting. Competing models of disease trajectories were ranked by the Akaike’s Information Criterion (AIC). A novel approach involving hierarchical clustering was developed to classify countries based on the effects of lockdown measures on new COVID-19 caseloads surrounding the lockdown period.

**Results:**

Univariate analysis identified 11 variables (employments in the agriculture, service and industrial sectors, percent population residing in urban areas, population age, number of visitors, and temperatures in the months of Jan-Apr) as independently associated with COVID-19 infections at a global level (variable *p* < 1E-05). Multivariable analysis identified a 5-variable model (percent urban population, percent employed in agriculture, population density, percent population aged 15–64 years, and temperature in March) as optimal for explaining global variations in COVID-19 (model adjusted R-squared = 0.68, model *p* < 2.20E-16). COVID-19 case trajectories for most countries were best captured by a log-logistic model, as determined by AIC estimates. Six predominant country clusters were identified when characterizing the effects of lockdown intervals on variations in COVID-19 new cases per country.

**Conclusions:**

Globally, economic and meteorological factors are important determinants of early COVID-19 incidence. Analysis of longitudinal trends and lockdown effects on COVID-19 highlights important nuances in country-specific responses to infections. These results provide valuable insights into disease incidence at a per-country level, possibly allowing for more informed decision making by individual governments in future disease outbreaks.

**Supplementary Information:**

The online version contains supplementary material available at 10.1186/s12889-022-14336-w.

## Background

First manifesting as an acute respiratory illness from infection with a zoonotically derived novel coronavirus named severe acute respiratory syndrome coronavirus 2 (SARS-CoV-2) [1], the associated coronavirus disease of 2019 (COVID-19) has rapidly spread worldwide with devastating impacts on public health and global economic activity [2], resulting in over 6.4 million reported deaths worldwide (as of July, 2022). Insights into the early epidemiological landscape of disease transmission, along with effects of public policy interventions, are crucial for providing evidence-based information that will help national authorities respond more effectively to future epidemics by tailoring public policy responses to specific geographic and social contexts. Along these lines, some important findings have been reported with respect to the effects of contact tracing and travel restrictions on COVID-19 spread [3, 4], as well as the evolving epidemiology and transmission dynamics of disease [4, 5]. However, important gaps in our understanding of the global nature and the scope of original interactions between the virus and its environments still remain [6].

Although the devastating second and third waves of the pandemic currently underway in many countries are much discussed, these waves are largely a result of variable viral mutations, diverse government policies and increased social interactions that has less to do with pre-existing natural and human factors that facilitated the pandemic in the first place [7, 8]. In this manuscript, we have focused exclusively on the association of these potential early factors to COVID-19 transmission by restricting our examinations to the global spread of the pandemic over the first 6 months, ending June 30, 2020.

A large body of pre-existing literature makes it clear that virus transmission is the result of an interaction among several factors, including host behavior and defense mechanisms, virus infectivity, population density and environmental determinants [9]. Previous studies on respiratory disorders have also emphasized the prevalent role of meteorological parameters on virus transmission and infectivity [10, 11]. For coronavirus infections, epidemiological and laboratory studies have identified ambient temperature to be a critical factor in the survival and transmission of other coronaviruses such as MERS-CoV and SARS-Cov-2 [12], and climate components including temperature, rainfall and wind speed have been postulated as biological catalysts for human-COVID-19 interactions in independent studies from several locations worldwide [11, 13, 14]. However, results from these studies are sometimes in conflict regarding the association between COVID-19 infection and the effect of temperature [15–20], highlighting the need for further investigations into these findings.

A retrospective analysis of government responses to epidemics and pandemics over the last century suggests that governments vary considerably in their adoption of non-medical interventions including quarantine, social distancing and contact tracing to stem the tide of public health disruptions [21]. In the absence of vaccines or effective pharmaceuticals, the majority of governments necessarily adopt some policy interventions to mitigate the spread of the disease. As COVID-19 assumed pandemic proportions, mitigating strategies by necessity had to become more stringent in order to flatten the curve of virus transmission. Consequently, contact suppression through lockdown emerged as the foremost administrative defense strategy in almost all countries to reduce mortality, preserve health-care service capacity, and buy time to develop effective pandemic control measures. However, socioeconomic pressures also necessitate that lockdowns be relaxed or lifted, even if temporarily, to prevent economic collapse. How such imposition and lifting of mandatory lockdowns affects COVID-19 caseloads is important for understanding the effectiveness of large-scale quarantine efforts.

Continuing along the lines of these prior reports, we have investigated the possible roles of specific pre-existing demographic, health, meteorological and economic variables in determining the first phase of COVID-19 infection burden globally. Additionally, we have characterized the heterogeneity in COVID-19 incidence trajectories across countries, and explored patterns in the differential influence of government-imposed lockdowns on the trajectories of new cases surrounding the lockdown periods. The goal of the present work is not so much to build predictive models of disease incidence or other outcomes, but rather to characterize the early factors associated with COVID-19 incidence, and investigate similarities and differences in the courses of disease incidence at a per-country level. Taken together, these analyses provide valuable information on global variations in disease incidence that would allow for more informed decision making for future infections.

## Methods

### Study design

This is an observational, cross-sectional study involving COVID-19 incidence data (total confirmed cases) from 203 countries over the first 6 months of the pandemic (Jan 1, 2020 to June 30, 2020), along with data on selected demographic, meteorological, economic and health-related determinants by country. All data are publicly available, aggregated at the country level, and do not contain any individual identifications.

### Data collection

Data for COVID-19 confirmed cases was obtained from https://ourworldindata.org/coronavirus-source-data, which is updated daily, and in turn is based on data maintained by Johns Hopkins University. Data on additional demographic, meteorological, health or economic variables were downloaded from a variety of sources listed in Table 1. For each variable, values from the most recent year for which data on the greatest number of countries were available were utilized (varied between 2016 and 2019). Variables were broadly categorized into Demographic, Meterological, Health or Economic domains. A full copy of the source datasets has been made available in Dryad (doi:10.5061/dryad.612jm6465).

**Table 1:** Sources for demographic, meteorological, health and economic data utilized in the current analysis. *Col 1*, abbreviated variable name; *col 2*, variable description; *col 3*, variable domain; *col 4*, year for which variable data was obtained; *col 5*, web-link to the source of the variable data

Variable	Description	Domain	Data_Year	Source
D_Age_15_64y_2018	Total population between the ages 15 to 64 as a percentage of the total population in 2018. Population is based on the de facto definition of population, which counts all residents regardless of legal status or citizenship.	Demographic	2018	data.worldbank.org
D_Pop_over65_2018	Population ages 65 and above as a percentage of the total population in 2018. Population is based on the de facto definition of population, which counts all residents regardless of legal status or citizenship.	Demographic	2018	data.worldbank.org
D_Popden2018	Population density in 2018. Calculated from total population and land area	Demographic	2018	Calculated from total population and land area
E_Employ_agri_%totemp_2018	Employment in agriculture, male (% total male employment), based on International Labour Organization (ILO) estimate	Economic	2018	data.worldbank.org
E_Employ_ind_%totemp_2018	Employment in agriculture, male (% total male employment), based on International Labour Organization (ILO) estimate	Economic	2018	data.worldbank.org
E_Employ_serv_%totemp_2018	Employment in agriculture, male (% total male employment), based on International Labour Organization (ILO) estimate	Economic	2018	data.worldbank.org
E_Total_visitors2018	Number of total registered visitors in 2018. International inbound tourists (overnight visitors) are the number of tourists who travel to a country other than that in which they have their usual residence, but outside their usual environment, for a period not exceeding 12 months and whose main purpose in visiting is other than an activity remunerated from within the country visited.	Economic	2018	data.worldbank.org
E_Urban_pct2018	Percentage of urban living population. Urban population refers to people living in urban areas as defined by national statistical offices. The data are collected and smoothed by United Nations Population Division.	Economic	2018	data.worldbank.org
G_Land_area_sqkm	Land area in square kilometers. Land area is a country’s total area, excluding area under inland water bodies, national claims to continental shelf, and exclusive economic zones. In most cases the definition of inland water bodies includes major rivers and lakes.	Meterological	2016	data.worldbank.org
G_Rain_mm_Apr2016	Average rainfall in millimeters in April 2016	Meterological	2016	https://climateknowledgeportal.worldbank.org/download-data
G_Rain_mm_Dec2016	Average rainfall in millimeters in December 2016	Meterological	2016	https://climateknowledgeportal.worldbank.org/download-data
G_Rain_mm_Feb2016	Average rainfall in millimeters in February 2016	Meterological	2016	https://climateknowledgeportal.worldbank.org/download-data
G_Rain_mm_Jan2016	Average rainfall in millimeters in January 2016	Meterological	2016	https://climateknowledgeportal.worldbank.org/download-data
G_Rain_mm_Mar2016	Average rainfall in millimeters in March 2016	Meterological	2016	https://climateknowledgeportal.worldbank.org/download-data
G_Temp_C_Apr2016	Average temperature in degrees Celsius in April 2016	Meterological	2016	https://climateknowledgeportal.worldbank.org/download-data
G_Temp_C_Feb2016	Average temperature in degrees Celsius in February 2016	Meterological	2016	https://climateknowledgeportal.worldbank.org/download-data
G_Temp_C_Jan2016	Average temperature in degrees Celsius in January 2016	Meterological	2016	https://climateknowledgeportal.worldbank.org/download-data
G_Temp_C_Mar2016	Average temperature in degrees Celsius in March 2016	Meterological	2016	https://climateknowledgeportal.worldbank.org/download-data
H_covid_duration_9Apr	A calculated estimate between April 9, 2020 and the first day of reported COVID-19 case(s) in a country	Health	2020	calculated
H_DALY_CVD_70yrs	One Disability Adjusted Life Year (DALY) is the equivalent of losing one year in good health because of either *premature death or disease or disability.* DALY estimates calculated per country by cardiovascular disease incidence for people aged 70 yrs. or more	Health	2016	https://www.who.int/data/gho/data/themes/mortality-and-global-health-estimates/global-health-estimates-leading-causes-of-dalys
H_DALY_CVD_all	One Disability Adjusted Life Year (DALY) is the equivalent of losing one year in good health because of either *premature death or disease or disability.* DALY estimates calculated per country by cardiovascular disease incidence across all age groups	Health	2016	https://www.who.int/data/gho/data/themes/mortality-and-global-health-estimates/global-health-estimates-leading-causes-of-dalys
H_DALY_resp_70yrs	One Disability Adjusted Life Year (DALY) is the equivalent of losing one year in good health because of either *premature death or disease or disability.* DALY estimates calculated per country by respiratory symptoms for people aged 70 yrs. or more	Health	2016	https://www.who.int/data/gho/data/themes/mortality-and-global-health-estimates/global-health-estimates-leading-causes-of-dalys
H_DALY_resp_all	One Disability Adjusted Life Year (DALY) is the equivalent of losing one year in good health because of either *premature death or disease or disability.* DALY estimates calculated per country by respiratory symptoms for all age groups	Health	2016	https://www.who.int/data/gho/data/themes/mortality-and-global-health-estimates/global-health-estimates-leading-causes-of-dalys
H_Diabetes2019	Diabetes prevalence refers to the percentage of people ages 20–79 who have type 1 or type 2 diabetes.	Health	2019	data.worldbank.org
H_Total_COVID_cases	Number of total reported COVID-19 cases per day per country	Health	Daily	https://covid19.who.int/

### Statistical analysis

The majority of the statistical analysis and visualizations were conducted via software packages in R (version 3.6.3, February 2020) through the R Studio IDE (version 1.2.5033–1, Dec 2019) [22]. All R scripts and accompanying data files area available from https://github.com/sg3451/covid-19-related.

#### Analysis of longitudinal trends in COVID-19 incidence per country

For each country, daily COVID-19 total confirmed case data was obtained from the day of the first reported infection until June 10, 2020. As the number of total COVID-19 cases varied widely between countries, we expressed the daily country-level increases in COVID-19 infections as a proportion of the maximum number of cases observed for that country (June 10, 2020), essentially scaling the data between 0 and 1 for each country. We assessed longitudinal trends in the rise in COVID-19 cases in each country by considering them as growth curves and fitting the number of confirmed COVID-19 infections using linear (quadratic) and nonlinear (exponential, logistic, log-logistic, and Gompertz) regression models. The modeling equations are given as below [23]:

*Logistic:* $f(x)=\alpha +\frac{\beta -\alpha }{1+{\left(\frac{x}{\gamma}\right)}^{\delta }}$*,* where α,β,γ,and δ are 4 estimable parameters representing the maximum asymptote (α), minimum asymptote (β), S-curve inflection point (γ) and Hill coefficient (δ), respectively.

*Log-logistic:* $f(x)=\alpha +\frac{\beta -\alpha }{1+{\left(\frac{\ln x}{\ln \gamma}\right)}^{\delta }}$*,* where all four parameters have the same meaning as for logistic regression.

*Gompertz: f*(*x*) = *β* + (*α* − *β*) *exp* (− *exp* (*γ*(*x* − *δ*)))*,* where β is the lower asymptote, α is the upper asymptote, γ is the growth-rate coefficient and δ is the time at inflection.

*Exponential*: $f(x)=\alpha +\left(\beta -\alpha \right)\mathit{\exp}\left(\frac{-x}{y}\right)$, a 3-parameter model where α is the lower asymptote, β is the upper asymptote and γ is the steepness of the growth curve.

*Quadratic: f*(*x*) = *α* + *β*_1_*x* + *β*_2_*x*^2^*,* where α is the value of f(x) at x = 0, and β_1_ and β_2_ are the polynomial regression coefficients.

A 4-parameter model was found to be optimum for logistic, log-logistic, and Gompertz fitted data. For each country, non-nested models were compared using the AIC criterion, and the model with the lowest AIC was selected. These analyses were conducted via the *drc* [23], *aomisc (*https://rdrr.io/github/OnofriAndreaPG/aomisc/*)* or *tidyverse* packages in R(v4.1.0) [24]. The *drc* and *aomisc* packages were used for their advantages of employing numerical optimization based self-starter functions for calculating initial values for the nonlinear regression models [25]. As we generated models on all countries simultaneously, it was considered judicious to use the data-guided self-starter functions available in these packages, rather than having the user guess the initial parameters for each model for each country separately.

#### Effect of lockdown on COVID-19 incidence per country

To identify the effect of the ‘lockdown’ period on new COVID-19 case trajectories in a country-specific manner, we obtained data on lockdown dates from https://auravision.ai/COVID-19-lockdown-tracker/, as well as internet-based reports from individual searches (Additional file 1), considering data until June 30, 2020. Countries that either had not imposed, or imposed but not withdrawn their lockdown by June 30 were excluded from the analysis (Peru, Belarus, Nepal, etc.), resulting in a final list of 106 countries with documented lockdown start and end dates. For countries with multiple lockdown dates (e.g. USA, China), the most common value (mode) of the lockdown start and end dates was taken to be representative for that country. The beginning and end of lockdown period was then overlaid on plots showing the number of daily new confirmed COVID-19 cases versus time. We considered a 5-point criteria to characterize a country’s response to the lockdown: (a) percent change in the number of daily cases between the beginning and end of lockdown, (b) presence of a peak in the number of daily cases within the lockdown period, (c) percent change in the number of daily cases 5 days after lifting of lockdown (early post-lockdown effects), (d) percent change in the number of daily cases 14 days after lifting of lockdown (later post-lockdown effects), and (e) percent change between day 5 and day 14 post-lockdown. The percent change values were then thresholded as follows: For (a),  > 20% change was indicated as 1,  < − 20% change was indicated as − 1 and a change between − 20 to 20% was indicated as 0. For (b), the presence of a peak was ascertained by visual inspection and indicated as 1 or 0 depending on the presence or absence of a peak. For (c, d, e), changes > 10% were indicated by 1, changes <− 10% were indicated by − 1 and changes between − 10 to 10% were indicated by 0. The thresholded data was used to cluster the countries via hierarchical clustering using the *pheatmap* function in R [26], such that countries with similar lockdown-related COVID-19 case patterns were grouped together. Specifically, we employed agglomerative hierarchical clustering where all countries were first treated as single clusters, followed by iterative joining of the least dissimilar countries to form larger clusters, until a single cluster was obtained. The pairwise similarity between any two countries was assessed via the Euclidean distance, defined as the shortest distance between two samples, whereas the distance between any two clusters was computed via the ‘complete linkage’ method [27]. In order to test the robustness of these cutoffs, we considered alternate values of 10 and 30% for (a), and 5 and 15% for (c,d,e), and calculated the number of countries with altered status based on the Hamming distance (calculated in R) [28] (Additional file 2). The results show that there was not a large change in the number of countries with altered status based on different cutoffs – out of 106 countries, only between 0 and 12 countries showed a change in status depending upon the criteria (a,c,d,e). Thus the results of hierarchical clustering are unlikely to change drastically as a function of different thresholds.

#### Regression analysis of global COVID-19 incidence

Bivariate linear regression analysis was conducted by examining the association of each demographic, meteorological, health or economic variable to the total number of confirmed COVID-19 cases (log10 transformed). A subset of the independent variables was also log transformed. Regression modeling was performed via the *tidyverse* package in R (www.tidyverse.org), by setting the modeling method to “lm” in the *geom_smooth* argument in *ggplot*. Results of the linear regression modeling were included in the graphs via the *ggpubr* and *ggpmisc* packages in R. A copy of the dataset used for bivariate regression is available from Dryad (doi:10.5061/dryad.612jm6465).

In addition to the bivariate analysis, we carried out variable subset selection in order to identify a parsimonious set of predictors for COVID-19 incidence. Models including all variables that were significant in bivariate analysis were first compared, and optimal sub-models, containing a combination of selected variables, were identified based on the Akaike Information Criterion (AIC). These analyses were conducted using the *lmSubsets* package in R [29], based on newly developed theoretical strategies for the ‘all-subset regression’ problem. The variables selected in the optimized models were then included in a multivariable linear regression model to assess their relative contributions to COVID-19 cases. Power analysis for multivariable linear regression was conducted via the *pwr* package in R [30, 31]. Multicollinearity among the selected variables was assessed via the variance inflation factor metric (VIF) through the ‘*car*’ package in R [32].

## Results

### Longitudinal trends in COVID-19 associations by country

Out of a total of 210 countries with available data, 38 countries with a maximum COVID-19 case load of less than 100 were excluded from the analysis. We further excluded Benin (BEN) because of an anomaly in its cumulative daily reported COVID-19 data which increased and then decreased over time. This resulted in a final list of 171 countries for longitudinal analysis of confirmed COVID-19 case patterns. For each country, the trajectory of total COVID-19 cases over time was examined via regression analysis, including both linear and non-linear regression models. The fits obtained with the various models were then compared using the AIC criterion and the model with the lowest AIC was selected as optimal for that country (Additional file 3). The longitudinal trends results show that the selected model fits the data for individual country well.

**Fig. 1:**
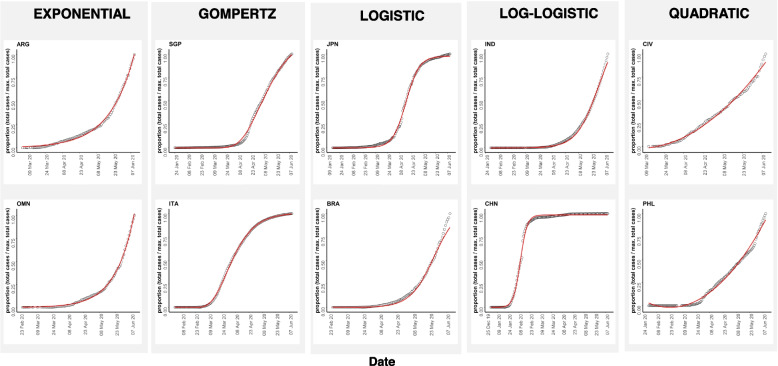
Analysis of the time-course of increase in COVID-19 total cases by country, using different growth-curve models. For each plot, the actual number of COVID-19 cases are shown as open circles and the fitted curve is shown in red. The y-axis refers to the proportion of daily total cases to the maximum total cases recorded in the time interval studied (0–1 scale), and the x-axis refers to the time-course as dates. The best growth-curve model for each country was determined by minimization of the AIC. Two exemplar countries for each model-type are shown with model names listed at the top. Countries are indicated by their ISO codes

From the 5 models considered, the COVID-19 trajectory for the majority of countries was best explained by the log-logistic model (70 countries), followed by logistic (44 countries) and Gompertz models (41 countries), whereas fewer countries were optimally explained by the quadratic (9 countries) and exponential models (6 countries). Figure 1 shows representative countries with optimal fits from the 5 modeling approaches (optimal model fits for all countries shown in Additional file 4).

### Association of individual variables to COVID-19 cases

Linear regression modeling of the logarithm of confirmed total COVID-19 reported cases for each country (normalized to per million population) against selected demographic, meteorological, economic and health indicators identified several variables as being significantly associated to the COVID-19 cases. To test the robustness of these findings, we analyzed total COVID-19 confirmed cases at 3 different time points approximately 1 month apart (April 10, May 11 and June 10). Table 2 shows the results of bivariate analysis for all 24 variables tested across the 3 time points, including coefficient estimates with standard error, adjusted coefficients of determination (R-squared) and significance of the regression fits.

**Table 2:** Results from bivariate regression analysis of demographic, meteorological, health and economic determinants of COVID-19 incidence tested across 3 time points. *Columns 1* and *2* show the variable names and an associated brief description, respectively. *Columns 3–5, 6–8, and 9–11* show results for the coefficients(+ standard error), adjusted r-squared and *p*-values from the regression fits of each variable to total COVID-19 cases in April, May and June 2020, respectively

Variable		coefficient(**+**SE)			Adjusted r-squared			***p***-value		
	Description	Apr 2020	May 2020	Jun 2020	Apr 2020	May 2020	Jun 2020	Apr 2020	May 2020	Jun 2020
D_Age_15_64y_2018	% total population aged 15–64 yrs. in 2018	0.094 (0.010)	0.076 (0.009)	0.064 (0.009)	0.325	0.278	0.236	8.99E-17	3.51E-14	4.69E-12
D_Pop_over65_2018	% total population over 65 yrs. in 2018	0.116 (0.009)	0.085 (0.009)	0.059 (0.009)	0.496	0.349	0.201	6.47E-28	3.47E-18	2.74E-10
D_Popden2018	Population density in 2018	0.380 (0.124)	0.368 (0.109)	0.255 (0.100)	0.043	0.053	0.029	0.002542	0.000919	0.011711
E_Employ2018 agri_pct_tot_emp	% total male employment in agriculture in 2018	−0.037 (0.002)	− 0.029 (0.002)	− 0.023 (0.002)	0.613	0.486	0.360	4.55E-37	1.39E-26	1.99E-18
E_Employ2018 ind_pct_tot_emp	% total male employment in industry in 2018	0.064 (0.009)	0.052 (0.008)	0.041 (0.007)	0.234	0.195	0.149	1.09E-11	7.69E-10	1.02E-07
E_Employ2018 serv_pct_tot_emp	% total male employment in service in 2018	0.044 (0.003)	0.035 (0.003)	0.027 (0.003)	0.566	0.442	0.324	6.86E-33	1.53E-23	2.2E-16
E_Urban_pct2018	% total population in urban areas in 2018	0.029 (0.003)	0.024 (0.002)	0.021 (0.002)	0.372	0.355	0.343	3.66E-21	4.73E-20	2.74E-19
G_Land_area_sqkm	land area in square kilometers	−0.358 (0.049)	−0.255 (0.044)	−0.166 (0.041)	0.207	0.139	0.071	6.12E-12	2.68E-08	7.8E-05
G_Rain_mm_Apr2016	avg. rainfall in mm in April 2016	0.128 (0.161)	0.036 (0.142)	−0.075 (0.128)	−0.002	− 0.005	−0.004	0.427862	0.801857	0.554773
G_Rain_mm_Feb2016	avg. rainfall in mm in Feb 2016	0.608 (0.120)	0.372 (0.109)	0.225 (0.100)	0.125	0.057	0.023	9.73E-07	0.000823	0.025551
G_Rain_mm_Jan2016	avg. rainfall in mm in Jan 2016	0.519 (0.120)	0.227 (0.110)	0.047 (0.100)	0.092	0.019	−0.005	2.56E-05	0.039809	0.640364
G_Rain_mm_Mar2016	avg. rainfall in mm in Mar 2016	0.343 (0.155)	0.143 (0.137)	0.008 (0.124)	0.022	0.000	−0.006	0.027904	0.300263	0.950179
G_Temp_C_Apr2016	avg. temp in ^o^C in April 2016	−0.064 (0.007)	−0.048 (0.006)	− 0.038 (0.006)	0.315	0.235	0.174	1.73E-16	3.71E-12	3.58E-09
G_Temp_C_Feb2016	avg. temp in ^o^C in Feb 2016	−0.047 (0.006)	−0.036 (0.005)	− 0.029 (0.005)	0.263	0.211	0.163	1.25E-13	6.03E-11	1.31E-08
G_Temp_C_Jan2016	avg. temp in ^o^C in Jan 2016	−0.040 (0.005)	−0.031 (0.005)	− 0.025 (0.004)	0.236	0.195	0.150	3.48E-12	3.79E-10	4.98E-08
G_Temp_C_Mar2016	avg. temp in ^o^C in Mar 2016	−0.054 (0.006)	−0.041 (0.005)	− 0.033 (0.005)	0.316	0.242	0.181	1.58E-16	1.55E-12	1.76E-09
H_covid_duration	duration (days) of COVID-19 from first reported date	1.707 (0.367)	2.574 (0.591)	2.964 (0.765)	0.093	0.082	0.065	6E-06	2.13E-05	0.000145
H_DALY_CVD_70yrs	DALY estimates by cardiovascular disease for > 70 yrs	−0.004 (0.101)	0.051 (0.090)	0.071 (0.082)	−0.006	−0.004	− 0.001	0.966794	0.570639	0.38638
H_DALY_CVD_all	DALY estimates by cardiovascular disease for all ages	−0.183 (0.102)	−0.078 (0.091)	− 0.018 (0.083)	0.013	− 0.002	−0.006	0.073734	0.389982	0.833299
H_DALY_resp_70yrs	DALY estimates by respiratory disease for > 70 yrs	−0.014 (0.103)	0.032 (0.091)	0.049 (0.083)	−0.006	−0.005	− 0.004	0.890447	0.727317	0.554885
H_DALY_resp_all	DALY estimates by respiratory disease for all ages	−0.241 (0.101)	− 0.134 (0.091)	− 0.068 (0.083)	0.027	0.007	−0.002	0.018512	0.139592	0.411349
H_Diabetes2019	% population between 20 and 79 yrs. with Type 1/Type 2 diabetes in 2019	0.028 (0.019)	0.016 (0.016)	0.013 (0.015)	0.007	0.000	−0.001	0.135637	0.330341	0.364124
E_TotVisitor2018 permillion	Total registered visitors per million population in 2018	0.911 (0.074)	0.693 (0.073)	0.527 (0.075)	0.489	0.363	0.237	1.42E-24	4.13E-17	6.06E-11

**Fig. 2:**
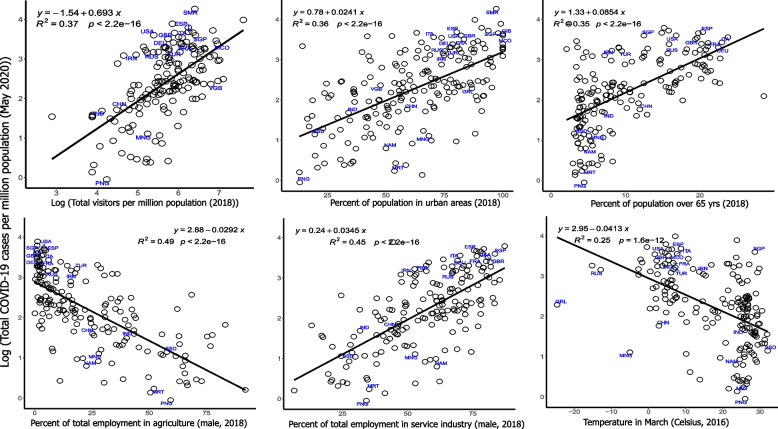
Association of selected variables with total COVID-19 cases in May 2020. Each plot shows the change in total COVID-19 cases per million population (expressed in log10 units) on the y-axis and the relevant variables on the x-axis. The line of best fit is shown along with its equation, the coefficient of determination (R2) and the associated significance of the regression model. Some selected countries with very high or very low COVID-19 cases are annotated by their ISO codes

A total of 11 variables including employments in the agriculture, service and industrial sectors, percent population residing in urban areas, population age, number of visitors, and temperatures in the months of Jan-Apr were found to be significant across all 3 time points tested (*p* < 1E-05), with the coefficient of determination (R^2^) ranging from 0.2–0.49 (May 11 data). Regression plots of the top 6 most significantly associated variables are shown for the May 11 data in Fig. 2 (plots for all 24 variables available in Additional file 5).

### Multivariable regression modeling of COVID-19 association

**Fig. 3:**
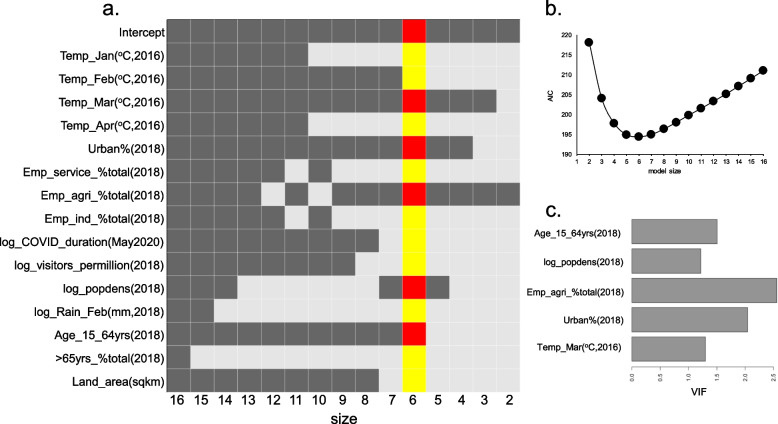
Multivariable regression analysis of variables associated with COVID-19 cases. **a** results from all-subsets regression analysis to identify the best sub-model with a smaller list of variables, based on minimization of the AIC. Selected variables are highlighted in red (in addition to the intercept). The x-axis refers to the model size (number of variables in each sub-model), and y-axis lists all the variables tested as follows: *Temp_Jan(*^*o*^*C,2016)*, temperatures in January in degrees Celsius in 2016; *Temp_Feb(*^*o*^*C, 2016)*, temperatures in February in 2016; *Temp_Mar(*^*o*^*C, 2016)*, temperatures in March, 2016; *Temp_Apr(*^*o*^*C, 2016)*, temperatures in April, 2016; *Urban%(2018)*, percentage of urban living population in 2018; *Emp_service_%total(2018)*, percentage of total male employment in service sector in 2018; *Emp_agri_%total(2018)*, percentage of total male employment in agriculture in 2018; *Emp_ind_%total(2018)*, percentage of total male employment in industry in 2018; *log-COVID_duration(May2020)*; duration (in days) between May 11, 2020 and the first reported COVID-19 case in a country (log10 scale); *log_popdens(2018)*, population density in 2018 (log10 scale); *log_Rain_Feb(mm,2018)*, rain in millimetres in February 2018 (log10 scale); *Age_15_64yrs(2018)*, population between the ages 15 to 64 as percentage of the total population in 2018; *>65yrs_%total(2018)*, population aged 65 and above as percentage of the total population in 2018; *Land_area(sqkm)*, land area in square kilometres. **b** change in AIC scores as a function of the number of variables included in the model. **c** Variance inflation factor (VIF) test of multicollinearity among the 5 variables in the sub-model identified from all-subsets regression analysis. The x-axis refers to the VIF scores and the y-axis refers to the selected variables

We used multivariable linear regression to identify a parsimonious subset of variables that can jointly explain the variation in the number of confirmed COVID-19 cases across countries. An all-subsets regression analysis was undertaken using variables with *p* < 0.01 in their respective bivariate analyses (15 variables), resulting in a series of sub-models consisting of different subsets of the variables included in the analysis. Data from 131 countries was finally available for modeling, after removing missing data. Power analysis [31] showed the power for multivariable regression under these conditions to vary between 0.86–0.96 for significance levels of 0.01–0.05, for small effects (coefficient of determination, R^2^ at 0.2). Based on AIC scores, a model with 5 variables (percent urban population, percent employed in agriculture, population density, percent population aged between 15 and 64 yrs., and temperature in March) was found to be the most parsimonious with respect to the global incidence of confirmed COVID-19 cases for May 11 data (model adjusted R^2^ = 0.68, model *p*-value < 2.20E-16) (Fig. 3a,b). In this figure, a total of 15 sub-models were generated containing between 1 and 15 regressors (excluding intercept). Variables are selected directly in relation to the frequency of their appearances in the sub-models. The population age related variable was not individually significant after adjusting for other variables for the May 11 data (Table 3). Multicollinearity among the selected 5 variables was tested via the variance inflation factor (VIF), and found to be low for all variables (VIFs< 5), requiring no further adjustments (Fig. 3c).

**Table 3:** Statistical summary of multivariable regression analysis. Statistical estimates of top variables identified after subset selection and minimization of the AIC are reported. *Col 1*, variable name; *col 2*, regression estimate for variable; *col 3*, standard error of estimate; *col 4*, t-statistic for regression; *col 5*, significance of variable association (*p*-value); *col 6*, variable association significance codes

Variable	Estimate	Std.Error	t	value	Pr(>|t|)
(Intercept)	1.25828	0.665479	1.891	0.06097	.
G_Temp_C_Mar2016	−0.02075	0.004482	−4.63	9.04E-06	***
E_Urban_pct2018	0.009165	0.002912	3.148	0.00206	**
E_Employ2018_agri_pct_tot_emp	−0.021051	0.003676	−5.726	7.22E-08	***
log_D_Popden2018	0.175727	0.087111	2.017	0.04581	*
D_Age_15_64y_2018	0.014129	0.009234	1.53	0.12854	

Significance codes: 0 ‘***’, 0.001 ‘**’, 0.01 ‘*’, 0.05 ‘.’

Residual standard error: 0.4992 on 125 degrees of freedom

Multiple R-squared: 0.6928; Adjusted R-squared: 0.6805

F-statistic: 56.39 on 5 and 125 degrees of freedom, *p*-value < 2.20E-16

### Effect of lockdown on new COVID-19 cases

**Fig. 4:**
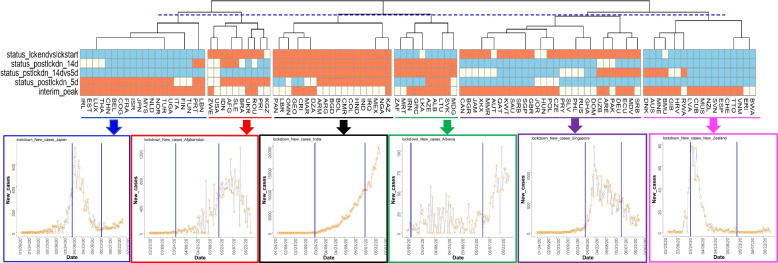
Characterization of new COVID-19 cases at the beginning and close of lockdowns. Countries were characterized on a 5-point heuristic based on new COVID-19 cases prior to, during, at the end of, and 5-days and 14-days post lockdown, and subjected to hierarchical clustering. Dendrogram and associated heatmap shows six major clusters (indicated by dashed blue line on the dendogram). Time-courses of new COVID-19 cases are shown for an exemplar country from each cluster, with the lockdown start and end days indicated by the two blue vertical bars in each plot. Heatmap is color-coded by the assigned values of the five-point criteria (− 1 = skyblue, 0 = ivory, 1 = coral)

As the majority of the countries adopted some measure of restriction (lockdown) to reduce the incidence of COVID-19 infection, and also removed such restriction (partially or entirely) after a certain period of time, we investigated the patterns by which the daily new cases of COVID-19 infections were affected due to the lockdown. Countries which had imposed and relaxed lockdowns by June 10, 2020 were considered, whereas data on total COVID-19 cases were considered until June 30, 2020 to identify post-lockdown trends. Lockdown-associated COVID-19 new case data was prepared and thresholded according to the 5-point criteria as described under Methods, and the per-country results are shown in Additional file 6. The thresholded dataset was then subjected to Euclidean hierarchical clustering and the results visualized by a dendrogram and heatmap (Fig. 4). The dendrogram was cut at the level of 6 branches (dotted blue line in Fig. 4) resulting in the countries being grouped into 6 different clusters. Representative plots for each of the six major clusters are shown alongside the dendrogram (lockdown plots for all countries are shown in Additional file 7).

## Discussion

Although the second, third and fourth waves of the pandemic are at the center of attention and discussion today, these outbreaks are primarily driven by viral mutagenesis, variable government policies and lack of social restraint, and have less predictive value for planning measures against future infection outbreaks. Pre-existing natural and human factors that engendered the pandemic in the first place are more informative for understanding the global COVID-19 experience, and for drawing important country-specific lessons that can be incorporated into future decision making. This is the motivation of the current study.

Despite a large body of research, uncertainties remain regarding the importance of environmental factors and their roles in COVID-19 transmission [33]. Results obtained from these studies have not conclusively resolved whether weather condition plays a key role in SARS-CoV-2 transmission [19]. Several factors can contribute to the observed discrepancies, including differences in outcome measures (counts of confirmed cases, new cases, or total cases or cumulative incidence rate), or weak correlations between temperature and COVID-19 propagation [16]. Compared to these published reports that focused on limited geographic regions, we examined global trends between confirmed COVID-19 cases and temperatures, by considering country-specific confirmed COVID-19 cases against their recorded monthly temperatures. Our results from both bivariate and multivariable analysis generally agree with a negative association of confirmed COVID-19 cases with temperatures, especially in the months of March and April.

In addition to the effects of temperature, our analysis also indicates a significant negative correlation of global COVID-19 incidence with markers of increased economic activity such as percent urban population and employment in industrial and service sectors, reflecting the consequences of increased congregation and socialization in the population [34]. This finding agrees with similar associations observed during the spread of other viral outbreaks with economic booms and trade expansions [35], for example an increased incidence of influenza associated with increases in employment [36]. These findings feed into the larger observation of the relationships between economic activity and population health, mediated by increased interactions between populations not otherwise exposed to each other’s disease ecologies (e.g. business and leisure visitors), and also dense permanent settlements around areas of high industrialization. Historically, both of these relationships have been found to negatively impact health of the populations exposed [37]. Overall, our analysis supports this trend. Multivariable regression modeling with variable subset selection further affirmed that a mixture of economic, demographic and meteorological variables was adequate for explaining the variation in total COVID cases at a global level.

The analysis of time course trajectories of COVID-19 incidence showed important differences among the countries examined. While the log-logistic and logistic models were adequate in modeling the COVID-19 trajectories for the majority of countries, there were nations whose SARS-Cov-2 incidence patterns were better modeled by exponential or quadratic fits. Such country-specific differences are probably the result of a combination of factors including natural elements (e.g. meteorology), socioeconomic regulators (e.g. urbanization), as well as governmental interventions (e.g. quarantines). Finally, we investigated the viral spread trajectories in additional detail by overlaying information on government-induced lockdown restrictions on the time course curves and estimating their effects on new COVID-19 case incidence. While effectively administered lockdowns are expected to successfully reduce the virus reproduction number, premature lockdown relaxation may lead to epidemic rebounding in still susceptible populations [38]. To identify possible recurring patterns in the countries’ experiences with COVID-19 incidence around lockdown, we employed hierarchical clustering, that allowed us to classify the responses into six main clusters depending on how the COVID-19 case numbers fluctuated before, during, and immediately after lockdowns. We found that countries such as Australia used lockdowns effectively to bring down the viral case-load to near zero levels well within the lockdown period, and kept it low post-lockdown, whereas another cluster represented by France for example, achieved near zero case-loads only as the lockdown was lifted. In contrast, countries such as India continued to see a steady rise in case numbers during, as well as after lockdown end, probably due to premature timing of both lockdown initiation and relaxation [38, 39]. While the current analyses do not establish causality between lockdown timings and COVID-19 incidence, it does allow for a retrospective assessment of the country clusters and identification of patterns representing variation in COVID-19 incidences around lockdowns. Based on these, the timing of a lockdown relative to the stage of the pandemic appears to be an important factor in SARS-CoV-2 transmission patterns, as also reported elsewhere [40].

Overall, our results provide empirical data on a global level that are consistent with some of the published modeling assumptions [41], and should prove useful for future policymaking. For example, our analysis of COVID-19 trends over time demonstrate country-specific trajectories following different growth models, and thereby provides important comparative information for future reference and planning in the respective countries. Similarly, our analysis of new COVID-19 cases around lockdown periods identify clusters containing countries with shared experiences, that should provide valuable comparative information regarding better planning of lockdown timing in relation to an infection’s spread. Finally, our multivariable analysis identified a small set of economic, meteorological and demographic variables that are significantly associated with global COVID-19 cases. Knowledge about such associations can allow individual countries to either take relevant mitigating steps when possible (e.g. reducing industrial economic activity sooner) or increase the pace of preventive measures if such countries have high indicators for the associated variables (e.g. countries with high urban population, high population density, lower agricultural employment, etc.).

Some limitations to the study are now discussed. First and foremost, we depended on publicly reported data on COVID-19 incidences per country, that, as has been pointed out, may have variable accuracies for different countries, especially with regard to underreporting of cases [42, 43]. Inefficient contact tracing, lack of accurate patient registration and differences in policy interventions are some of the contributing factors for inaccurate estimates of disease incidence, which could introduce some bias in the results reported in this study. Secondly, in our efforts to maximize the number of countries with complete information on candidate factors affecting COVID-19 incidence, we sometimes had to use slightly older datasets (e.g. from 2016 for temperature and rainfall) which could potentially differ from more recent estimates. This could also change the regression modeling estimates somewhat, although the lessons learned would most likely still remain the same. Finally, the results obtained in this study are at the national level and should not be extrapolated to sub-national units due to the possibility of ecological fallacy. For example, regional variation in COVID-19 incidence or differential susceptibility among different age groups in a country can, in fact, introduce ecological bias when aggregated at the national level.

In summary, our analysis contributes to the field of infectious diseases in three important aspects. First, our study models the trajectory of SARS-CoV-2 spread at a country level and specifies important differences in the time-course of virus transmission around the world. Second, we examine key economic, meteorological, geographic and health determinants of the spread of COVID-19 on a global level. Third, our study investigates virus-infection statistics around lockdown periods and identifies both similarities and differences in the countries’ experiences with new virus infections around such restrictions. These analyses provide valuable prior data on disease incidence at global and national scales, allowing for more informed decision making for future disease outbreaks.

## Supplementary Information

**Additional file 1.** Lockdown start and end dates by country (and ciies) until June 30, 2020.

**Additional file 2.** Table of Hamming distances to estimate the effect of different thresholds on country status around lockdown periods.

**Additional file 3.** Akaike Information Criterion (AIC) scores for different modeling fits of COVID-19 incidence trajectories per country.

**Additional file 4.** Analysis of the time-course of increase in COVID-19 total cases by country, using different growth-curve models.

**Additional file 5.** Association of selected demographic, economic and meteorological variables with total COVID-19 cases in May 2020, as determined by univariate linear regression.

**Additional file 6.** Characterization of COVID-19 incidence surrounding lockdown imposition and relaxation at a per country level.

**Additional file 7.** Country-specific trajectories of new COVID-19 cases around lockdown periods until June 30, 2020.

## Data Availability

The datasets supporting the conclusions of this article are included within the article and its supplementary information files. Additional source data and data analysis scripts are available from Dryad (doi:10.5061/dryad.612jm6465) and github (https://github.com/sg3451/covid-19-related).
