# Supplementary material for: Global-scale modeling of early factors and country-specific trajectories of COVID-19 incidence: a cross-sectional study of the first 6 months of the pandemic
Source: BMC Public Health. 2022 Oct 14;22:1919. doi: 10.1186/s12889-022-14336-w (PMC9568998; doi:10.1186/s12889-022-14336-w)
Supplement: Supplementary file 2 — Additional file 2. Table of Hamming distances to estimate the effect of different thresholds on country status around lockdown periods. [file 12889_2022_14336_MOESM2_ESM.pdf]

Additional File 2: Table of Hamming distances to estimate the effect of different thresholds on country status around lockdown periods. A 5-point criteria was used to annotate countries based on the COVID-19 caseloads surrounding lockdown periods in each country: (a) percent change in the number of daily cases at the beginning and end of lockdown, (b) the presence of a peak in the number of daily cases within the lockdown period, (c) percent change in the number of daily cases 5 days after lifting of lockdown (early post-lockdown effects), (d) percent change in the number of daily cases 14 days after lifting of lockdown (later post-lockdown effects), and (e) percent change between day 5 and day 14 post-lockdown. The original thresholds applied were as follows: For (a), a >20% change was indicated as 1, a <-20% change was indicated as -1 and a change between -20% to 20% was indicated as 0. For (b), the presence of a peak was ascertained by visual inspection and indicated as 1 or 0 depending on the presence or absence of a peak. For (c, d, e), changes >10% were indicated by 1, changes <-10% were indicated by -1 and changes between -10% to 10% were indicated by 0. Alternate thresholds tested were ±10% and ±30% change for (a) and ±5 and ±15% changes for (c,d,e). The number of countries showing different assignments (0/1/-1) based on a comparison of the original thresholds to the altered thresholds are listed. Hamming distance was calculated based on 106 countries. The 5 lockdown criteria are symbolized as follows in the table: **a**, status_lckendvslckstart; **b**, interim peak; **c**, status_postlckdn_5d; **d**, status_postlckdn_14d; **e**, status_pstlckdn_14dvs5d

**comp1**	**comp2**	**hamming_dist**
a_10%	a_20%	0
a_10%	a_30%	6
a_20%	a_30%	6
c_5%	c_10%	0
c_5%	c_15%	12
c_10%	c_15%	12
d_5%	d_10%	0
d_5%	d_15%	5
d_10%	d_15%	5
e_5%	e_10%	5
e_5%	e_15%	9
e_10%	e_15%	4
