# Supplementary material for: Global-scale modeling of early factors and country-specific trajectories of COVID-19 incidence: a cross-sectional study of the first 6 months of the pandemic
Source: BMC Public Health. 2022 Oct 14;22:1919. doi: 10.1186/s12889-022-14336-w (PMC9568998; doi:10.1186/s12889-022-14336-w)
Supplement: Supplementary file 3 — Additional file 3. Akaike Information Criterion (AIC) scores for different modeling fits of COVID-19 incidence trajectories per country. [file 12889_2022_14336_MOESM3_ESM.pdf]

Additional File 3: **Akaike Information Criterion (AIC) scores for different modeling fits of COVID-19 incidence trajectories per country.**

**Time-dependent increases in COVID-19 incidence in each country was modeled by 5 different fits (logistic, log-logistic, Gompertz, exponential and quadratic) and the AIC for each fit was obtained. The fit with the lowest AIC was considered the best descriptor of COVID-19 incidence trajectory for that country. E=exponential, G=Gompertz, L=logistic, LL=log logistic, Q=quadratic**

| iso_code | location | aic_exponential_aomisc | aic_quadratic_aomisc | aic_gompertz | aic_logistic | aic_loglogistic | min_aic | min_model |
|---|---|---|---|---|---|---|---|---|
| ARG | Argentina | -519.4359 | -293.1 | -399.3947 | -510.3414 | -429.7279 | -519.4359 | E |
| ARM | Armenia | -575.4335 | -281.2955 | -429.9532 | -569.1829 | -481.008 | -575.4335 | E |
| KEN | Kenya | -492.1391 | -264.4357 | -364.1499 | -483.4558 | -403.5553 | -492.1391 | E |
| MOZ | Mozambique | -347.0875 | -234.0068 | -286.808 | -341.6595 | -297.3055 | -347.0875 | E |
| OMN | Oman | -608.6447 | -266.261 | -469.5128 | -598.7713 | -514.9805 | -608.6447 | E |
| SYR | Syria | -189.9028 | -169.8439 | -177.1733 | -185.3791 | -173.7686 | -189.9028 | E |
| ABW | Aruba | -37.1124 | -179.6666 | -383.5624 | -375.8171 | -370.1277 | -383.5624 | G |
| AUS | Australia | 288.8622 | -98.56375 | -721.5018 | -630.9336 | -679.4295 | -721.5018 | G |
| BHS | Bahamas | -65.48152 | -286.1183 | -460.9797 | -436.6443 | -454.0847 | -460.9797 | G |
| BLR | Belarus | 124.3315 | -417.2244 | -719.0496 | -532.2615 | -639.1237 | -719.0496 | G |
| BEL | Belgium | 236.721 | -192.2218 | -928.5873 | -653.8464 | -813.9948 | -928.5873 | G |
| KHM | Cambodia | 293.3314 | -92.24171 | -560.7456 | -539.0694 | -553.1181 | -560.7456 | G |
| CAN | Canada | -240.6206 | -403.0425 | -1043.556 | -728.9181 | -874.5596 | -1043.556 | G |
| COM | Comoros | -65.00342 | -88.53149 | -109.4742 | -103.4227 | -107.8058 | -109.4742 | G |
| HRV | Croatia | 236.2205 | -177.6417 | -694.42 | -503.2007 | -659.0569 | -694.42 | G |
| FIN | Finland | 222.1002 | -275.2527 | -926.8893 | -716.9593 | -885.2132 | -926.8893 | G |
| FRA | France | -91.54441 | -188.9969 | -810.3587 | -629.3746 | -735.6116 | -810.3587 | G |
| DEU | Germany | -71.38762 | -169.7339 | -834.1931 | -607.9412 | -724.5686 | -834.1931 | G |
| GIN | Guinea | 131.4185 | -336.2047 | -560.8072 | -471.5141 | -554.1017 | -560.8072 | G |
| GNB | Guinea-Bissau | -89.86203 | -146.9462 | -359.1439 | -303.4059 | -334.8317 | -359.1439 | G |
| ISR | Israel | 224.026 | -164.021 | -598.7116 | -502.0449 | -586.4122 | -598.7116 | G |
| ITA | Italy | 260.8388 | -210.9075 | -934.4075 | -631.0801 | -808.6129 | -934.4075 | G |
| MYS | Malaysia | 230.397 | -275.1989 | -607.1743 | -504.8873 | -576.6348 | -607.1743 | G |
| MDV | Maldives | -203.4072 | -322.667 | -441.595 | -390.2536 | -419.5333 | -441.595 | G |
| MRT | Mauritania | -368.8885 | -156.8842 | -449.6888 | -447.4561 | -407.1135 | -449.6888 | G |
| NZL | New_Zealand | 240.2938 | -124.1334 | -623.8233 | -474.6146 | -590.2486 | -623.8233 | G |
| NIC | Nicaragua | -182.5548 | -112.206 | -284.0722 | -269.6554 | -273.2338 | -284.0722 | G |
| NGA | Nigeria | 93.12115 | -373.688 | -588.4661 | -506.709 | -564.1186 | -588.4661 | G |
| PSE | Palestine | -106.5762 | -242.5927 | -337.1454 | -335.8945 | -336.1737 | -337.1454 | G |
| ROU | Romania | 179.5701 | -286.4403 | -694.1917 | -514.8765 | -676.8608 | -694.1917 | G |
| RUS | Russia | 120.7514 | -423.8718 | -1019.606 | -787.6958 | -896.9339 | -1019.606 | G |
| SGP | Singapore | -282.7371 | -412.8369 | -835.0261 | -680.2723 | -742.8103 | -835.0261 | G |
| SVN | Slovenia | -36.25767 | -330.946 | -594.9116 | -515.0559 | -524.3848 | -594.9116 | G |
| SOM | Somalia | 115.8344 | -336.8206 | -448.8508 | -382.0417 | -441.0927 | -448.8508 | G |
| KOR | South Korea | 317.3303 | -217.9089 | -547.1069 | -468.4442 | -527.6811 | -547.1069 | G |
| ESP | Spain | -51.42859 | -159.2056 | -788.6015 | -583.0118 | -702.1987 | -788.6015 | G |
| SUR | Suriname | -210.2892 | -92.87623 | -426.363 | -401.3942 | -294.6721 | -426.363 | G |
| SWZ | Swaziland | -204.057 | -304.9156 | -393.6947 | -357.9433 | -383.835 | -393.6947 | G |
| SWE | Sweden | 152.1216 | -599.7472 | -701.6013 | -608.5926 | -684.5854 | -701.6013 | G |
| THA | Thailand | 288.6975 | -121.0405 | -842.2996 | -688.2195 | -752.1801 | -842.2996 | G |
| TTO | Trinidad_and_Tobago | -13.16185 | -153.3113 | -377.3256 | -362.5323 | -375.8425 | -377.3256 | G |
| UKR | Ukraine | 134.5356 | -352.3908 | -521.3244 | -448.8891 | -514.2782 | -521.3244 | G |
| ARE | United_Arab_Emirates | -391.183 | -518.4 | -884.4118 | -764.0728 | -846.7397 | -884.4118 | G |
| GBR | United_Kingdom | -196.4518 | -330.2519 | -994.419 | -690.1365 | -830.009 | -994.419 | G |
| USA | United_States | -276.3895 | -457.1768 | -831.8523 | -647.3242 | -739.3606 | -831.8523 | G |
| VNM | Vietnam | -83.79162 | -198.6398 | -453.4304 | -421.9267 | -449.511 | -453.4304 | G |
| YEM | Yemen | -192.2829 | -278.6972 | -304.3151 | -276.8409 | -302.5162 | -304.3151 | G |
| AFG | Afghanistan | 75.20697 | -267.3883 | -659.0859 | -769.6437 | -684.1644 | -769.6437 | L |
| DZA | Algeria | 147.8677 | -465.995 | -591.062 | -604.5712 | -598.3841 | -604.5712 | L |
| AZE | Azerbaijan | -433.3739 | -325.4042 | -385.118 | -438.5728 | -381.3475 | -438.5728 | L |
| BOL | Bolivia | -441.4922 | -270.6955 | -532.805 | -606.7738 | -553.4904 | -606.7738 | L |
| BRA | Brazil | -533.6372 | -324.3367 | -783.4708 | -819.0281 | -799.8339 | -819.0281 | L |
| BRN | Brunei | -58.41021 | -170.1948 | -475.4641 | -486.5877 | -431.1208 | -486.5877 | L |
| BGR | Bulgaria | -175.6322 | -324.4719 | -451.9472 | -483.2497 | -450.2278 | -483.2497 | L |
| CMR | Cameroon | -422.8009 | -341.5096 | -418.6513 | -463.6158 | -415.0359 | -463.6158 | L |
| CHL | Chile | 73.63594 | -291.1997 | -541.8297 | -623.6542 | -562.9857 | -623.6542 | L |
| COL | Colombia | -531.4528 | -339.0274 | -575.0414 | -717.6664 | -556.521 | -717.6664 | L |
| COD | Democratic Republic of Congo | -423.7926 | -328.8589 | -514.3976 | -585.9027 | -527.1454 | -585.9027 | L |
| DJI | Djibouti | 96.86081 | -222.5696 | -245.858 | -261.3541 | -240.2804 | -261.3541 | L |
| EGY | Egypt | -715.9543 | -330.8873 | -620.4612 | -792.0326 | -676.7329 | -792.0326 | L |
| SLV | El Salvador | -284.1813 | -393.5157 | -492.6497 | -538.6895 | -518.8214 | -538.6895 | L |

| | | | | | | | | |
|---|---|---|---|---|---|---|---|---|
| GNQ | Equatorial Guinea | -184.848 | -268.3965 | -295.3989 | -308.0626 | -300.4626 | -308.0626 | L |
| ETH | Ethiopia | -444.8667 | -179.1884 | -394.9899 | -478.6834 | -457.0415 | -478.6834 | L |
| FRO | Faeroe Islands | -134.314 | -202.9913 | -533.1052 | -536.7499 | -484.6154 | -536.7499 | L |
| GAB | Gabon | -249.4319 | -306.1942 | -463.4074 | -525.2767 | -510.8885 | -525.2767 | L |
| GTM | Guatemala | -415.1563 | -256.3496 | -493.8615 | -527.1234 | -503.6336 | -527.1234 | L |
| HND | Honduras | -339.1489 | -349.3233 | -459.1551 | -504.7013 | -483.6571 | -504.7013 | L |
| ISL | Iceland | 5.018274 | -203.7473 | -521.6432 | -712.5129 | -569.628 | -712.5129 | L |
| IRQ | Iraq | -358.9644 | -240.274 | -303.2333 | -378.603 | -345.369 | -378.603 | L |
| JPN | Japan | 249.7093 | -211.0231 | -754.037 | -857.9649 | -851.9757 | -857.9649 | L |
| KWT | Kuwait | -330.3434 | -325.5369 | -574.0966 | -708.6893 | -665.4664 | -708.6893 | L |
| LBY | Libya | -182.2106 | -144.7013 | -208.2521 | -222.5981 | -213.555 | -222.5981 | L |
| MDG | Madagascar | -321.841 | -211.1728 | -340.4128 | -367.9508 | -354.516 | -367.9508 | L |
| MWI | Malawi | -179.9557 | -118.7264 | -214.8729 | -227.7988 | -224.5619 | -227.7988 | L |
| MUS | Mauritius | -13.44839 | -148.1111 | -349.7391 | -394.3464 | -334.7457 | -394.3464 | L |
| MNG | Mongolia | -160.9919 | -173.3632 | -207.5932 | -216.8627 | -213.2572 | -216.8627 | L |
| MNE | Montenegro | -9.9015 | -194.5633 | -417.6422 | -439.3091 | -411.5469 | -439.3091 | L |
| NPL | Nepal | -772.5703 | -186.6951 | -736.8629 | -812.4892 | -694.3476 | -812.4892 | L |
| PAK | Pakistan | 72.11925 | -357.1864 | -569.8906 | -597.6492 | -589.1561 | -597.6492 | L |
| PRY | Paraguay | -203.8209 | -278.1733 | -306.3096 | -326.5595 | -321.2364 | -326.5595 | L |
| PRI | Puerto_Rico | -296.0552 | -320.6779 | -325.3588 | -344.4649 | -303.7455 | -344.4649 | L |
| SMR | San Marino | -107.0072 | -322.8579 | -454.8554 | -485.0689 | -433.3266 | -485.0689 | L |
| SLE | Sierra_Leone | -161.5772 | -268.5225 | -390.1726 | -429.0014 | -394.2264 | -429.0014 | L |
| SVK | Slovakia | -40.40369 | -204.0995 | -353.5257 | -444.3017 | -382.7082 | -444.3017 | L |
| ZAF | South_Africa | -713.1142 | -277.9243 | -489.2998 | -714.8869 | -563.1096 | -714.8869 | L |
| TJK | Tajikistan | -69.97804 | -133.1642 | -197.9593 | -212.6947 | -197.3153 | -212.6947 | L |
| TZA | Tanzania | -0.7878212 | -70.468 | -294.4116 | -306.5018 | -300.2692 | -306.5018 | L |
| TGO | Togo | -241.3733 | -267.9437 | -280.5283 | -312.2453 | -298.6332 | -312.2453 | L |
| UGA | Uganda | -260.0813 | -184.8116 | -221.1829 | -275.4897 | -267.0315 | -275.4897 | L |
| VEN | Venezuela | -360.9993 | -213.7441 | -378.6897 | -404.4584 | -381.59 | -404.4584 | L |
| ZWE | Zimbabwe | -240.4124 | -138.4916 | -271.1943 | -282.4253 | -279.3634 | -282.4253 | L |
| ALB | Albania | -180.2771 | -414.5921 | -403.9503 | -340.6403 | -449.2842 | -449.2842 | LL |
| AND | Andorra | -48.54891 | -205.7091 | -372.12 | -333.0738 | -378.6237 | -378.6237 | LL |
| AUT | Austria | 245.5047 | -205.2538 | -517.5523 | -420.187 | -534.191 | -534.191 | LL |
| BHR | Bahrain | -536.9529 | -420.3046 | -672.5993 | -660.6068 | -680.0634 | -680.0634 | LL |
| BGD | Bangladesh | -418.8996 | -325.7193 | -626.1149 | -579.3237 | -696.6032 | -696.6032 | LL |
| BMU | Bermuda | -75.12337 | -274.5523 | -312.1753 | -308.354 | -313.7703 | -313.7703 | LL |
| BIH | Bosnia and Herzegovina | -121.5209 | -308.9959 | -485.7773 | -375.8362 | -509.2348 | -509.2348 | LL |
| BFA | Burkina Faso | -90.7018 | -339.3211 | -376.577 | -331.3556 | -426.0207 | -426.0207 | LL |
| CPV | Cape Verde | -205.46 | -322.098 | -351.4381 | -329.7493 | -352.0379 | -352.0379 | LL |
| CAF | Central African Republic | -492.5666 | -213.6687 | -520.2174 | -537.1035 | -552.0606 | -552.0606 | LL |
| TCD | Chad | -138.017 | -215.6217 | -425.884 | -429.5412 | -449.8989 | -449.8989 | LL |
| CHN | China | 409.8893 | -211.8449 | -801.3443 | -842.9381 | -854.0821 | -854.0821 | LL |
| COG | Congo | -251.8946 | -398.8223 | -390.7207 | -372.2798 | -399.5034 | -399.5034 | LL |
| CUB | Cuba | -84.31355 | -228.5059 | -472.0589 | -387.2562 | -487.1679 | -487.1679 | LL |
| CYP | Cyprus | -43.82064 | -260.1209 | -489.6963 | -410.2633 | -527.0066 | -527.0066 | LL |
| CZE | Czech_Repub lic | 211.1751 | -281.4305 | -438.3096 | -361.793 | -472.3774 | -472.3774 | LL |
| DNK | Denmark | 212.0067 | -276.6672 | -602.8864 | -515.6257 | -610.8224 | -610.8224 | LL |
| DOM | Dominican_R epublic | 129.3501 | -542.6278 | -593.0407 | -494.751 | -646.8393 | -646.8393 | LL |
| ECU | Ecuador | 161.8695 | -203.0704 | -311.348 | -306.7964 | -314.4104 | -314.4104 | LL |
| EST | Estonia | 220.672 | -246.7971 | -504.6721 | -424.5989 | -527.6343 | -527.6343 | LL |
| GEO | Georgia | -134.246 | -262.075 | -547.201 | -499.7239 | -571.5538 | -571.5538 | LL |
| GHA | Ghana | 110.745 | -335.9963 | -396.8478 | -381.6732 | -398.8628 | -398.8628 | LL |
| GIB | Gibraltar | -79.91624 | -180.4305 | -256.819 | -255.7615 | -281.8402 | -281.8402 | LL |
| GRC | Greece | -65.8449 | -284.4489 | -548.4726 | -459.9016 | -584.0823 | -584.0823 | LL |
| GUM | Guam | -83.86717 | -207.4634 | -266.7225 | -269.0415 | -297.0752 | -297.0752 | LL |
| GGY | Guernsey | -5.304221 | -172.6126 | -415.9911 | -425.0924 | -432.2642 | -432.2642 | LL |
| GUY | Guyana | -161.4976 | -334.9942 | -372.5732 | -366.4181 | -379.8538 | -379.8538 | LL |
| HTI | Haiti | -366.388 | -174.0277 | -489.0297 | -507.2827 | -512.5395 | -512.5395 | LL |
| HUN | Hungary | 191.8024 | -239.8258 | -621.2835 | -475.6198 | -638.5135 | -638.5135 | LL |
| IND | India | 60.53107 | -345.8236 | -1076.049 | -1025.569 | -1217.242 | -1217.242 | LL |
| IDN | Indonesia | 116.4559 | -629.9846 | -566.1467 | -483.9269 | -630.076 | -630.076 | LL |
| IRN | Iran | 172.1402 | -449.9572 | -418.7691 | -408.6297 | -465.8453 | -465.8453 | LL |
| IRL | Ireland | 209.4387 | -165.0268 | -559.2162 | -586.6975 | -625.698 | -625.698 | LL |
| IMN | Isle of Man | -13.90298 | -209.3267 | -398.8593 | -400.2791 | -408.1989 | -408.1989 | LL |
| JAM | Jamaica | -71.39612 | -152.2579 | -394.2924 | -382.9004 | -404.4485 | -404.4485 | LL |
| JEY | Jersey | -37.8474 | -264.8204 | -399.0455 | -378.7026 | -408.5039 | -408.5039 | LL |
| KAZ | Kazakhstan | -306.3074 | -471.5966 | -479.7348 | -446.8391 | -486.9395 | -486.9395 | LL |
| OWID_ KOS | Kosovo | -107.5783 | -243.9919 | -339.6378 | -306.9429 | -349.6144 | -349.6144 | LL |
| LVA | Latvia | -87.24654 | -346.6816 | -439.3656 | -406.3895 | -453.592 | -453.592 | LL |
| LBR | Liberia | -195.854 | -331.8112 | -345.9563 | -291.569 | -363.7065 | -363.7065 | LL |
| LTU | Lithuania | 209.201 | -197.1854 | -381.921 | -317.9006 | -393.7108 | -393.7108 | LL |

| | | | | | | | | |
|---|---|---|---|---|---|---|---|---|
| LUX | Luxembourg | 232.1051 | -212.3696 | -510.8381 | -374.8697 | -536.564 | -536.564 | LL |
| MKD | Macedonia | 124.5843 | -300.5874 | -287.241 | -265.1456 | -303.7303 | -303.7303 | LL |
| MLI | Mali | -219.4592 | -400.0941 | -362.9061 | -308.5409 | -406.9345 | -406.9345 | LL |
| MLT | Malta | -101.5559 | -319.1074 | -338.9974 | -291.6384 | -362.5572 | -362.5572 | LL |
| MEX | Mexico | 87.73245 | -377.0196 | -840.3216 | -672.1857 | -852.9599 | -852.9599 | LL |
| MDA | Moldova | 129.9677 | -482.7673 | -463.9427 | -405.7322 | -516.3397 | -516.3397 | LL |
| MAR | Morocco | 166.2563 | -252.4766 | -580.4489 | -550.9611 | -591.5384 | -591.5384 | LL |
| MMR | Myanmar | -91.58642 | -227.3966 | -256.931 | -244.4117 | -268.6348 | -268.6348 | LL |
| NLD | Netherlands | 216.2215 | -230.5749 | -696.7019 | -553.8551 | -721.2155 | -721.2155 | LL |
| NER | Niger | -46.6877 | -220.3004 | -253.8218 | -238.7944 | -267.3848 | -267.3848 | LL |
| NOR | Norway | 239.7677 | -285.6923 | -599.3417 | -496.5518 | -652.9174 | -652.9174 | LL |
| PER | Peru | 100.6404 | -475.6739 | -626.6595 | -566.5708 | -629.8398 | -629.8398 | LL |
| POL | Poland | 149.0126 | -434.1856 | -514.8712 | -476.8072 | -557.1157 | -557.1157 | LL |
| PRT | Portugal | 191.5806 | -279.8206 | -454.4319 | -391.1689 | -477.3903 | -477.3903 | LL |
| QAT | Qatar | 100.4169 | -433.8739 | -732.6619 | -702.281 | -766.49 | -766.49 | LL |
| STP | Sao Tome and Principe | -89.14566 | -129.8387 | -140.3291 | -137.3855 | -140.3943 | -140.3943 | LL |
| SAU | Saudi_Arabia | 106.6419 | -484.318 | -650.6945 | -610.679 | -656.5145 | -656.5145 | LL |
| SEN | Senegal | -275.2635 | -397.5647 | -496.4703 | -514.174 | -531.8181 | -531.8181 | LL |
| SRB | Serbia | 189.3387 | -191.3527 | -558.3205 | -464.2723 | -573.9561 | -573.9561 | LL |
| SSD | South Sudan | -209.9274 | -204.6344 | -250.9443 | -251.4258 | -252.0148 | -252.0148 | LL |
| LKA | Sri_Lanka | 128.8065 | -449.3042 | -528.0057 | -511.0591 | -528.0899 | -528.0899 | LL |
| SDN | Sudan | 91.9268 | -321.7591 | -560.5731 | -551.1181 | -580.8621 | -580.8621 | LL |
| CHE | Switzerland | 248.5261 | -211.5366 | -822.3088 | -569.1514 | -863.091 | -863.091 | LL |
| TWN | Taiwan | -29.62892 | -137.7052 | -648.1754 | -627.018 | -652.3505 | -652.3505 | LL |
| TUN | Tunisia | -31.88137 | -225.999 | -554.8591 | -460.5878 | -555.6231 | -555.6231 | LL |
| TUR | Turkey | 180.8283 | -232.4939 | -508.1436 | -406.3355 | -532.9472 | -532.9472 | LL |
| URY | Uruguay | -129.8379 | -419.8751 | -400.9915 | -370.1214 | -517.4208 | -517.4208 | LL |
| UZB | Uzbekistan | -186.3442 | -322.1371 | -298.8413 | -257.6641 | -328.785 | -328.785 | LL |
| ZMB | Zambia | -130.4609 | -176.5468 | -302.1519 | -334.6131 | -340.0396 | -340.0396 | LL |
| CYM | Cayman Islands | -221.3734 | -259.0723 | -249.8907 | -242.2448 | -248.4093 | -259.0723 | Q |
| CRI | Costa_Rica | -191.1178 | -301.1858 | -261.0247 | -232.6146 | -295.076 | -301.1858 | Q |
| CIV | Cote d'Ivoire | -292.9623 | -413.3115 | -377.5052 | -339.006 | -407.4024 | -413.3115 | Q |
| JOR | Jordan | -188.6798 | -292.6486 | -257.1936 | -235.6517 | -281.7566 | -292.6486 | Q |
| KGZ | Kyrgyzstan | -255.8855 | -404.4822 | -354.5095 | -320.6407 | -385.5423 | -404.4822 | Q |
| LBN | Lebanon | 175.1033 | -346.7072 | -304.0947 | -272.5269 | -344.154 | -346.7072 | Q |
| PAN | Panama | -281.5108 | -430.3588 | -381.2639 | -342.1064 | -423.7495 | -430.3588 | Q |
| PHL | Philippines | -423.8084 | -573.5642 | -539.9027 | -511.7262 | -557.7275 | -573.5642 | Q |
| RWA | Rwanda | -219.716 | -371.5937 | -339.9174 | -317.9441 | -367.7955 | -371.5937 | Q |
