# Supplementary material for: Global-scale modeling of early factors and country-specific trajectories of COVID-19 incidence: a cross-sectional study of the first 6 months of the pandemic
Source: BMC Public Health. 2022 Oct 14;22:1919. doi: 10.1186/s12889-022-14336-w (PMC9568998; doi:10.1186/s12889-022-14336-w)
Supplement: Supplementary file 4 — Additional file 4. Analysis of the time-course of increase in COVID-19 total cases by country, using different growth-curve models. [file 12889_2022_14336_MOESM4_ESM.pdf]

Additional File 4. Analysis of the time-course of increase in COVID-19 total cases by country, using different growth-curve models. For each plot, the actual number of COVID-19 cases are shown as open circles and the fitted curve is shown in red. The y-axis refers to the proportion of daily total cases to the maximum total cases recorded in the time interval studied (0-1 scaling), and the x-axis refers to the time-course in day-month-year format. The best growth-curve model for each country was determined by minimization of the AIC.

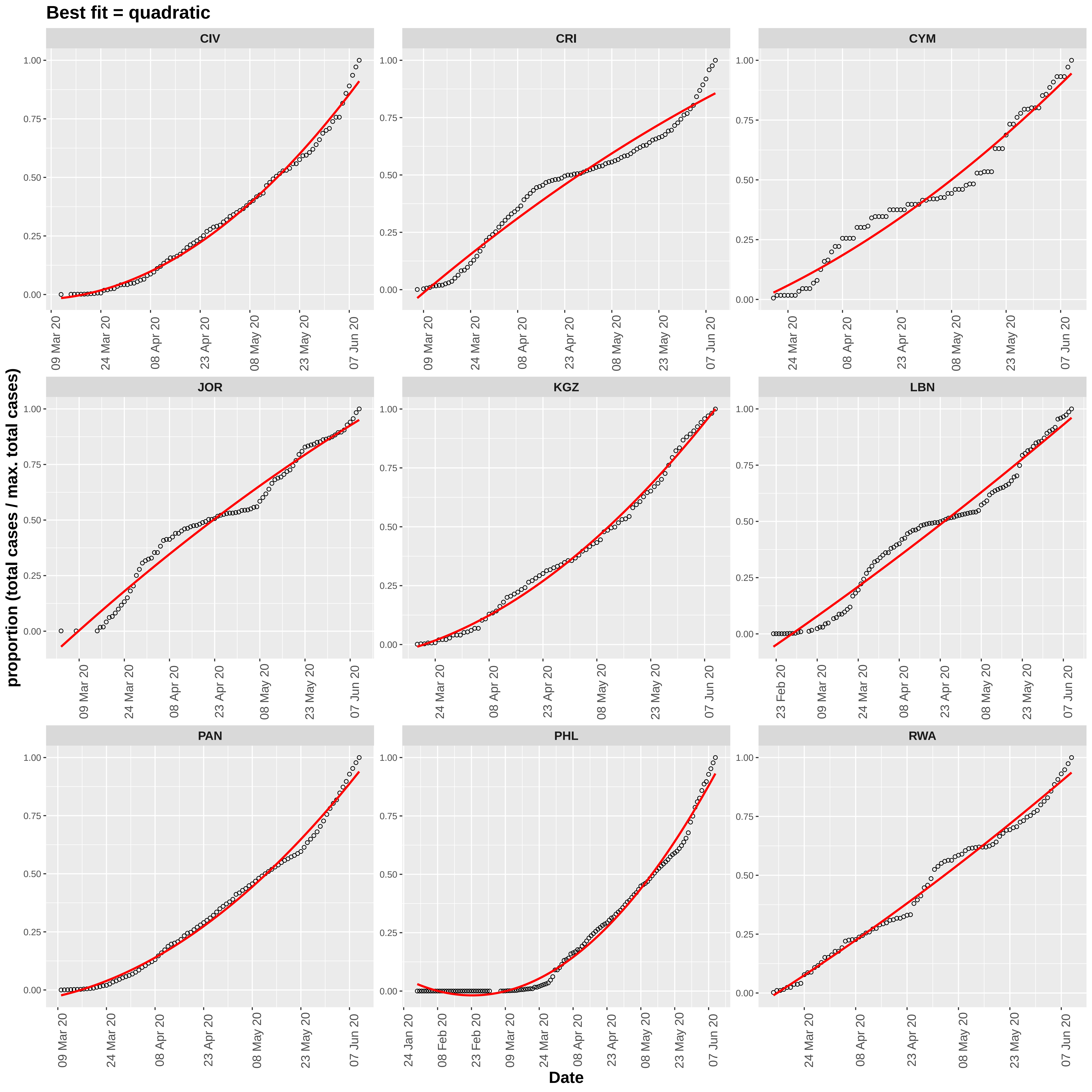
Best fit = quadratic
CIV
CRI
CYM
JOR
KGZ
LBN
PAN
PHL
RWA
proportion (total cases / max. total cases)
Date
0.00
0.25
0.50
0.75
1.00
09 Mar 20
24 Mar 20
08 Apr 20
23 Apr 20
08 May 20
23 May 20
07 Jun 20
23 Feb 20
24 Jan 20
08 Feb 20

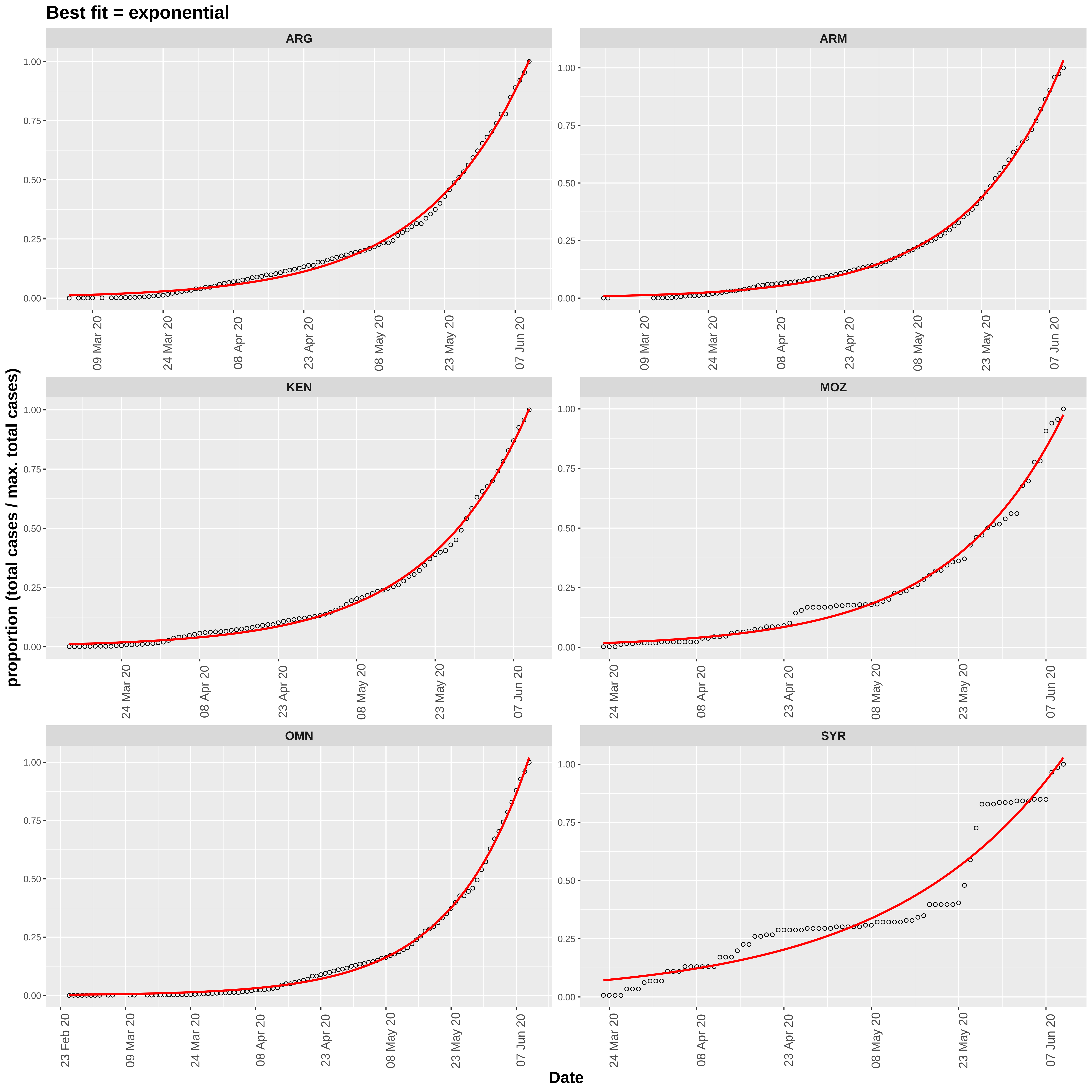

Best fit = exponential
ARG
ARM
KEN
MOZ
OMN
SYR
proportion (total cases / max. total cases)
Date
0.00
0.25
0.50
0.75
1.00
23 Feb 20
09 Mar 20
24 Mar 20
08 Apr 20
23 Apr 20
08 May 20
23 May 20
07 Jun 20

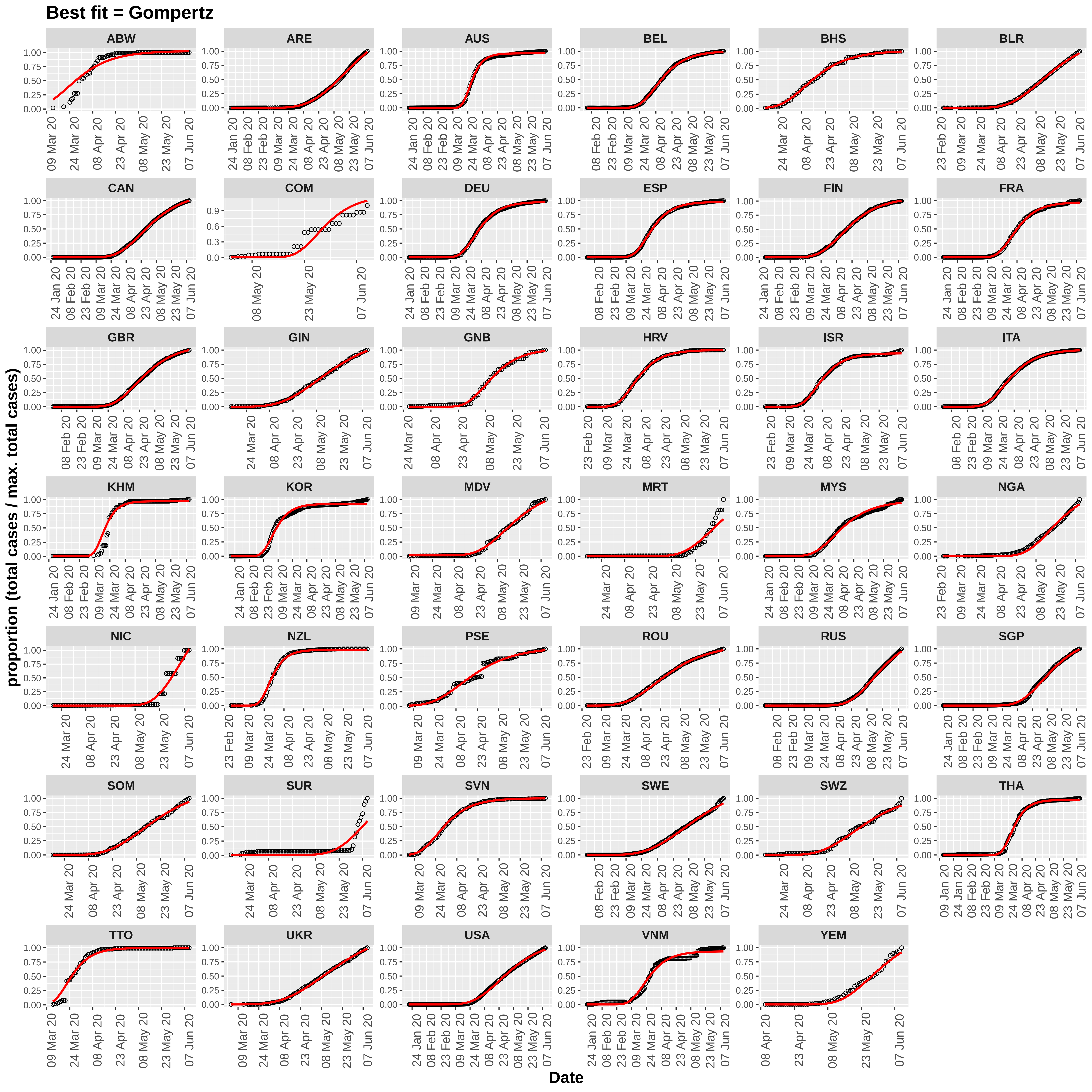

Best fit = Gompertz
ABW
ARE
AUS
BEL
BHS
BLR
CAN
COM
DEU
ESP
FIN
FRA
GBR
GIN
GNB
HRV
ISR
ITA
KHM
KOR
MDV
MRT
MYS
NGA
NIC
NZL
PSE
ROU
RUS
SGP
SOM
SUR
SVN
SWE
SWZ
THA
TTO
UKR
USA
VNM
YEM
proportion (total cases / max. total cases)
Date

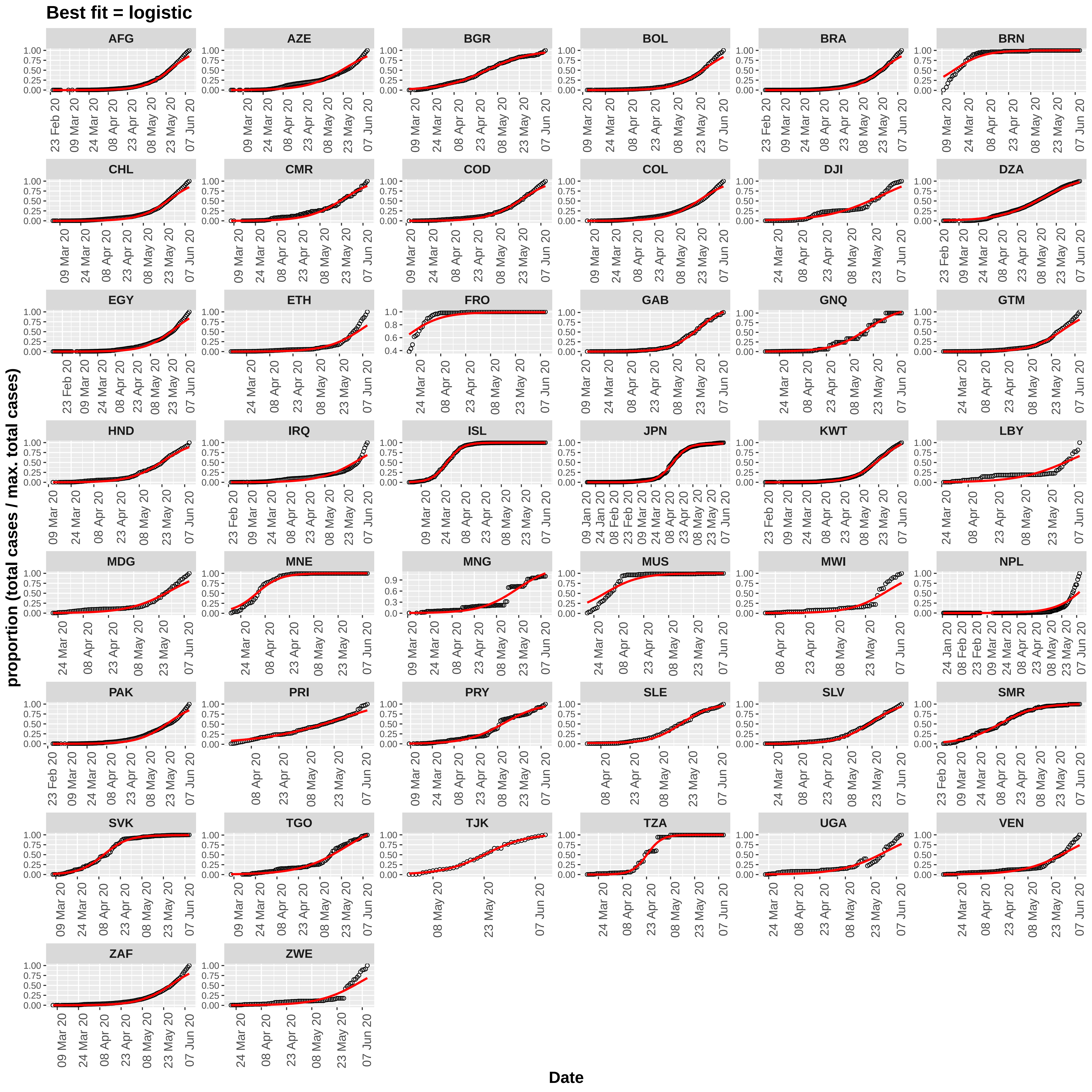

Best fit = logistic
AFG
AZE
BGR
BOL
BRA
BRN
CHL
CMR
COD
COL
DJI
DZA
EGY
ETH
FRO
GAB
GNQ
GTM
HND
IRQ
ISL
JPN
KWT
LBY
MDG
MNE
MNG
MUS
MWI
NPL
PAK
PRI
PRY
SLE
SLV
SMR
SVK
TGO
TJK
TZA
UGA
VEN
ZAF
ZWE
proportion (total cases / max. total cases)
Date

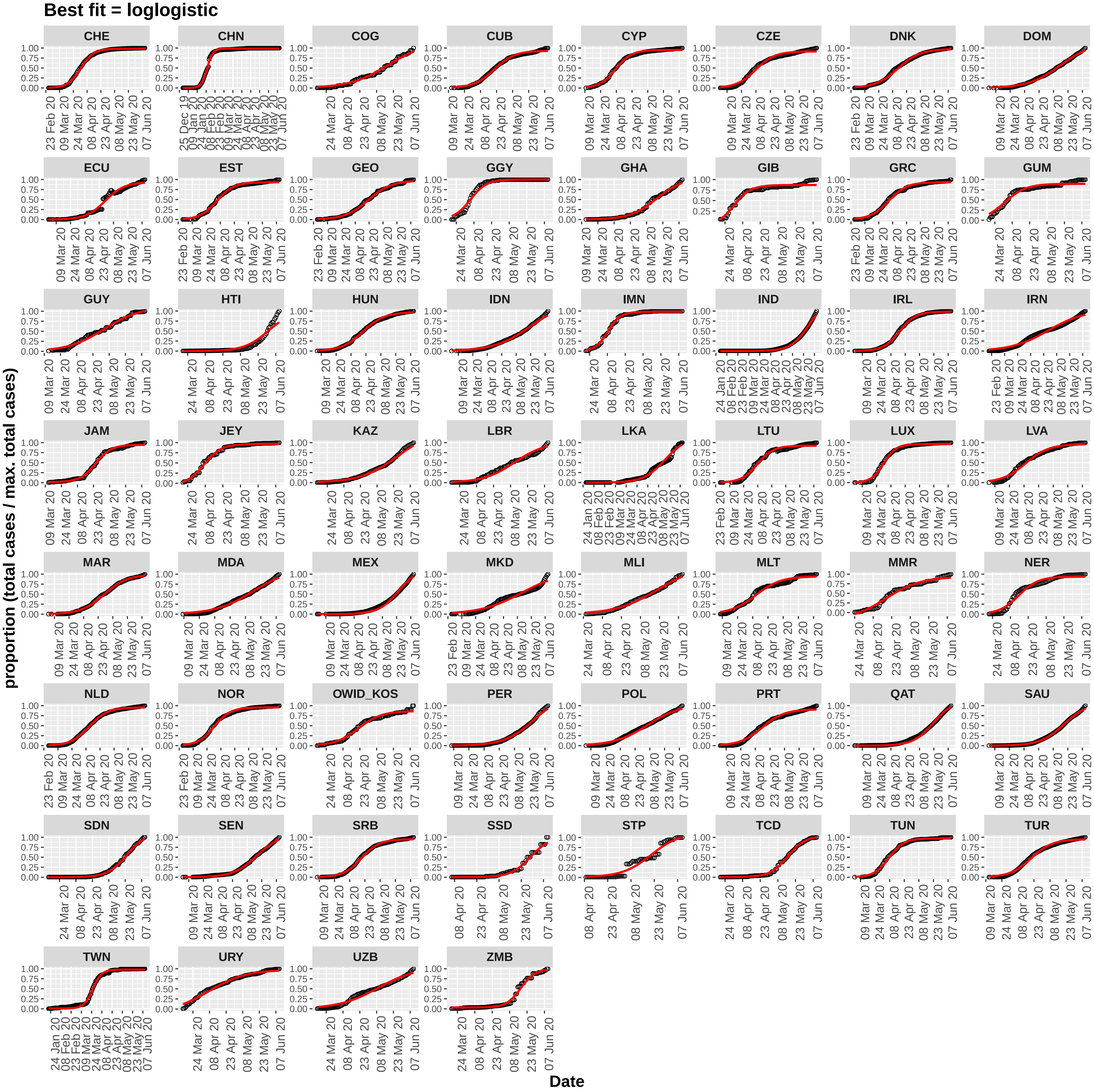

Best fit = loglogistic
CHE
CHN
COG
CUB
CYP
CZE
DNK
DOM
ECU
EST
GEO
GGY
GHA
GIB
GRC
GUM
GUY
HTI
HUN
IDN
IMN
IND
IRL
IRN
JAM
JEY
KAZ
LBR
LKA
LTU
LUX
LVA
MAR
MDA
MEX
MKD
MLI
MLT
MMR
NER
NLD
NOR
OWID_KOS
PER
POL
PRT
QAT
SAU
SDN
SEN
SRB
SSD
STP
TCD
TUN
TUR
TWN
URY
UZB
ZMB
proportion (total cases / max. total cases)
Date
