# Supplementary material for: Global-scale modeling of early factors and country-specific trajectories of COVID-19 incidence: a cross-sectional study of the first 6 months of the pandemic
Source: BMC Public Health. 2022 Oct 14;22:1919. doi: 10.1186/s12889-022-14336-w (PMC9568998; doi:10.1186/s12889-022-14336-w)
Supplement: Supplementary file 5 — Additional file 5. Association of selected demographic, economic and meteorological variables with total COVID-19 cases in May 2020, as determined by univariate linear regression. [file 12889_2022_14336_MOESM5_ESM.pdf]

Additional File 5: Association of selected demographic, economic and meteorological variables with total COVID-19 cases in May 2020, as determined by univariate linear regression. For each plot, the X-axis shows the analysed variable and the Y-axis represents the logarithm of the country-level total COVID-19 cases per million people in May 2020. The regression equation and the associated R2 and p-value are also shown. The variables analysed are described as below:

**Variable**	**Description**
D_Age_15_64y_2018	Total population between the ages 15 to 64 as a percentage of the total population in 2018.
D_Pop_over65_2018	Population ages 65 and above as a percentage of the total population in 2018.
D_Popden2018	Population density in 2018.
E_Employ_agri_%totemp_2018	Employment in agriculture, male (% total male employment)
E_Employ_ind_%totemp_2018	Employment in agriculture, male (% total male employment)
E_Employ_serv_%totemp_2018	Employment in agriculture, male (% total male employment)
E_Total_visitors2018	Number of total registered visitors in 2018.
E_Urban_pct2018	Percentage of urban living population.
G_Land_area_sqkm	Land area in square kilometers.
G_Rain_mm_Apr2016	Average rainfall in millimeters in April 2016
G_Rain_mm_Dec2016	Average rainfall in millimeters in December 2016
G_Rain_mm_Feb2016	Average rainfall in millimeters in February 2016
G_Rain_mm_Jan2016	Average rainfall in millimeters in January 2016
G_Rain_mm_Mar2016	Average rainfall in millimeters in March 2016
G_Temp_C_Apr2016	Average temperature in degrees Celsius in April 2016
G_Temp_C_Feb2016	Average temperature in degrees Celsius in February 2016
G_Temp_C_Jan2016	Average temperature in degrees Celsius in January 2016
G_Temp_C_Mar2016	Average temperature in degrees Celsius in March 2016
H_covid_duration_9Apr	A calculated estimate between April 9, 2020 and the first day of reported COVID-19 case(s) in a country
H_DALY_CVD_70yrs	Disability Adjusted Life Year estimates calculated per country by cardiovascular disease incidence for people aged 70 yrs or more
H_DALY_CVD_all	Disability Adjusted Life Year estimates calculated per country by cardiovascular disease incidence across all age groups
H_DALY_resp_70yrs	Disability Adjusted Life Year estimates calculated per country by respiratory symptoms for people aged 70 yrs or more
H_DALY_resp_all	Disability Adjusted Life Year estimates calculated per country by respiratory symptoms for all age groups
H_Diabetes2019	Diabetes prevalence refers to the percentage of people ages 20-79 who have type 1 or type 2 diabetes.
H_Total_COVID_cases	Number of total reported COVID-19 cases per day per country

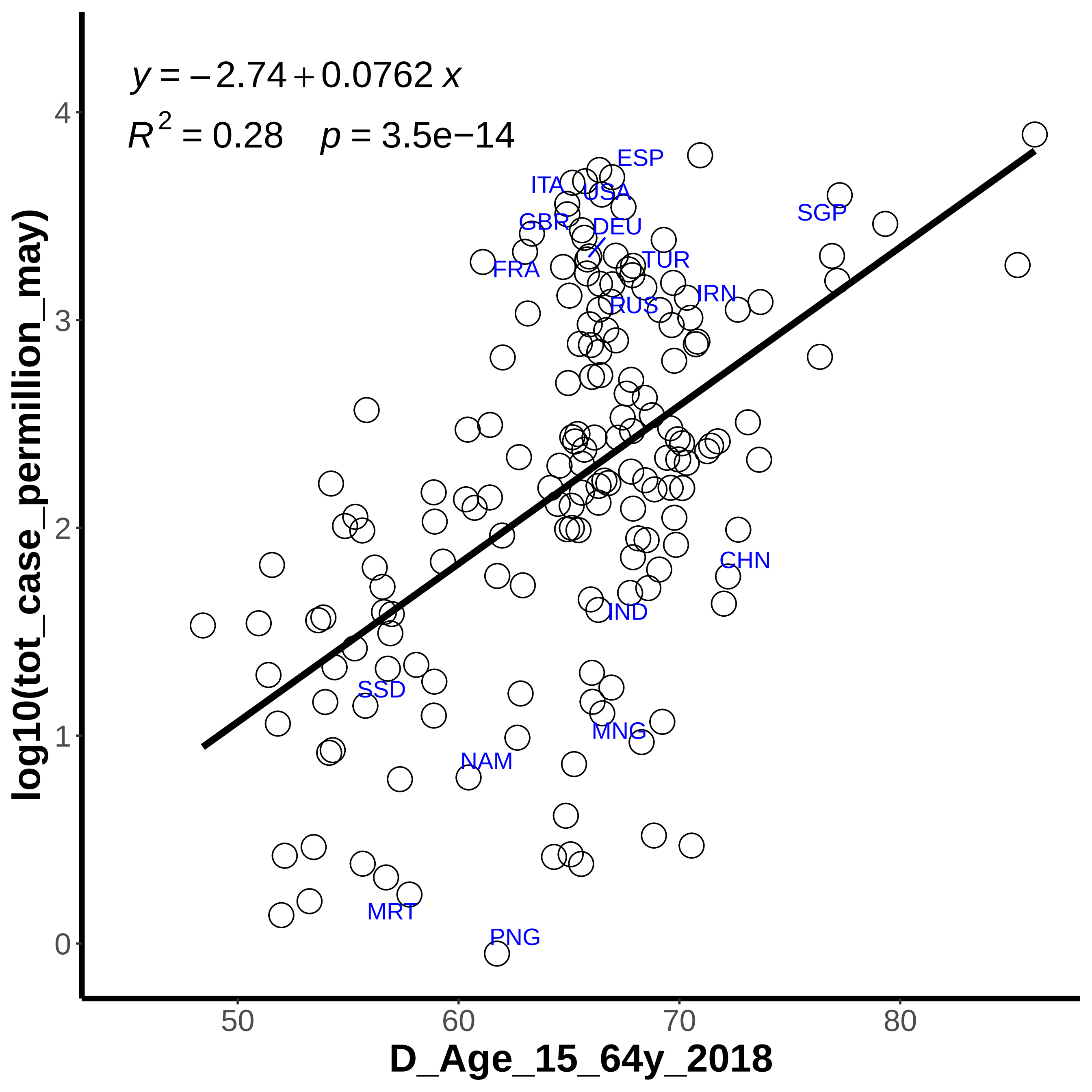

$y = -2.74 + 0.0762\,x$
$R^2 = 0.28 \quad p = 3.5e-14$
ESP
ITA
USA
GBR
DEU
FRA
TUR
IRN
RUS
SGP
CHN
IND
SSD
MNG
NAM
MRT
PNG
0
1
2
3
4
50
60
70
80
log10(tot_case_permillion_may)
D_Age_15_64y_2018

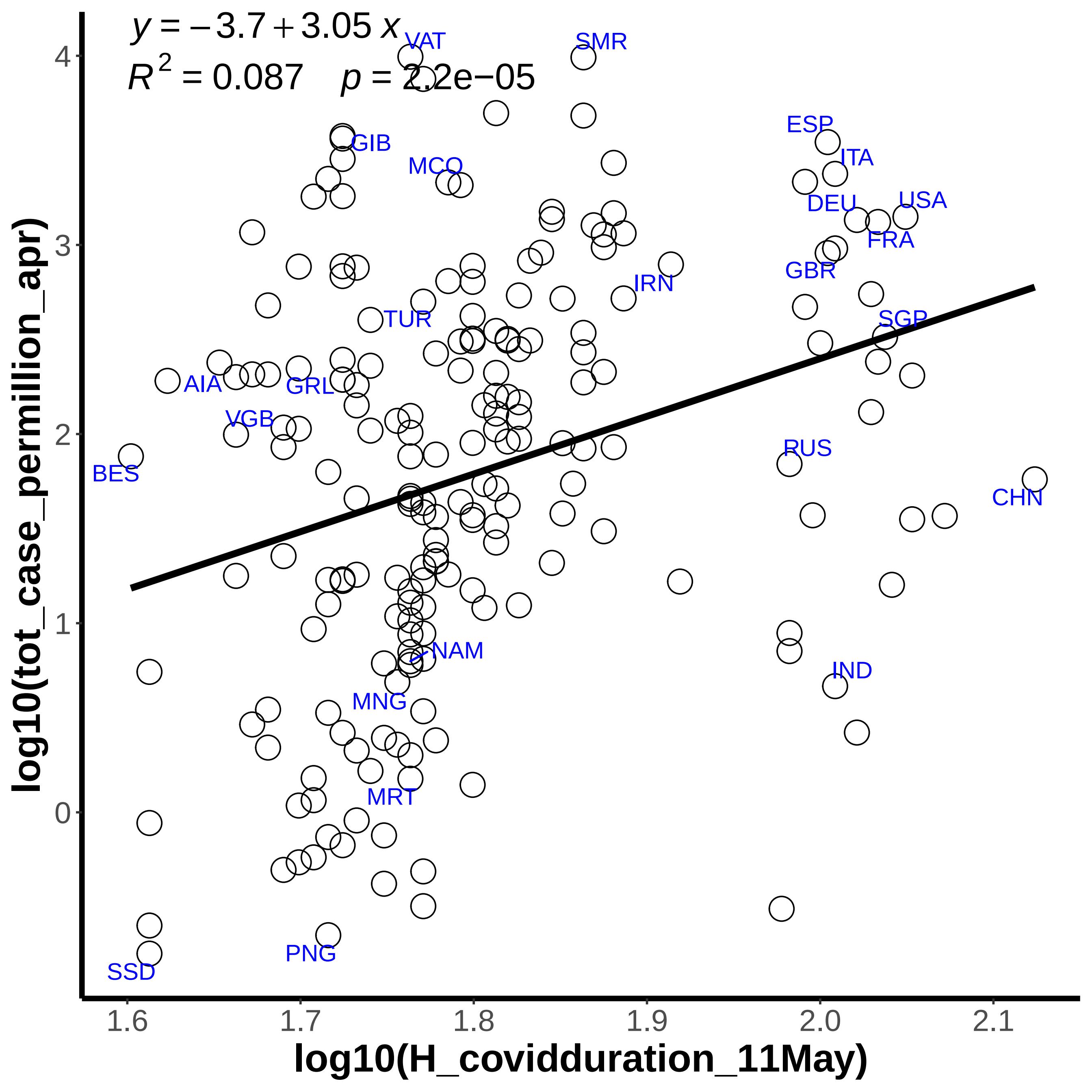

$y = -3.7 + 3.05\,x$
$R^2 = 0.087 \quad p = 2.2e{-05}$
log10(tot_case_permillion_apr)
log10(H_coviddduration_11May)
0
1
2
3
4
1.6
1.7
1.8
1.9
2.0
2.1
VAT
SMR
ESP
ITA
GIB
MCO
DEU
USA
FRA
GBR
IRN
TUR
SGP
AIA
GRL
VGB
BES
RUS
CHN
NAM
IND
MNG
MRT
PNG
SSD

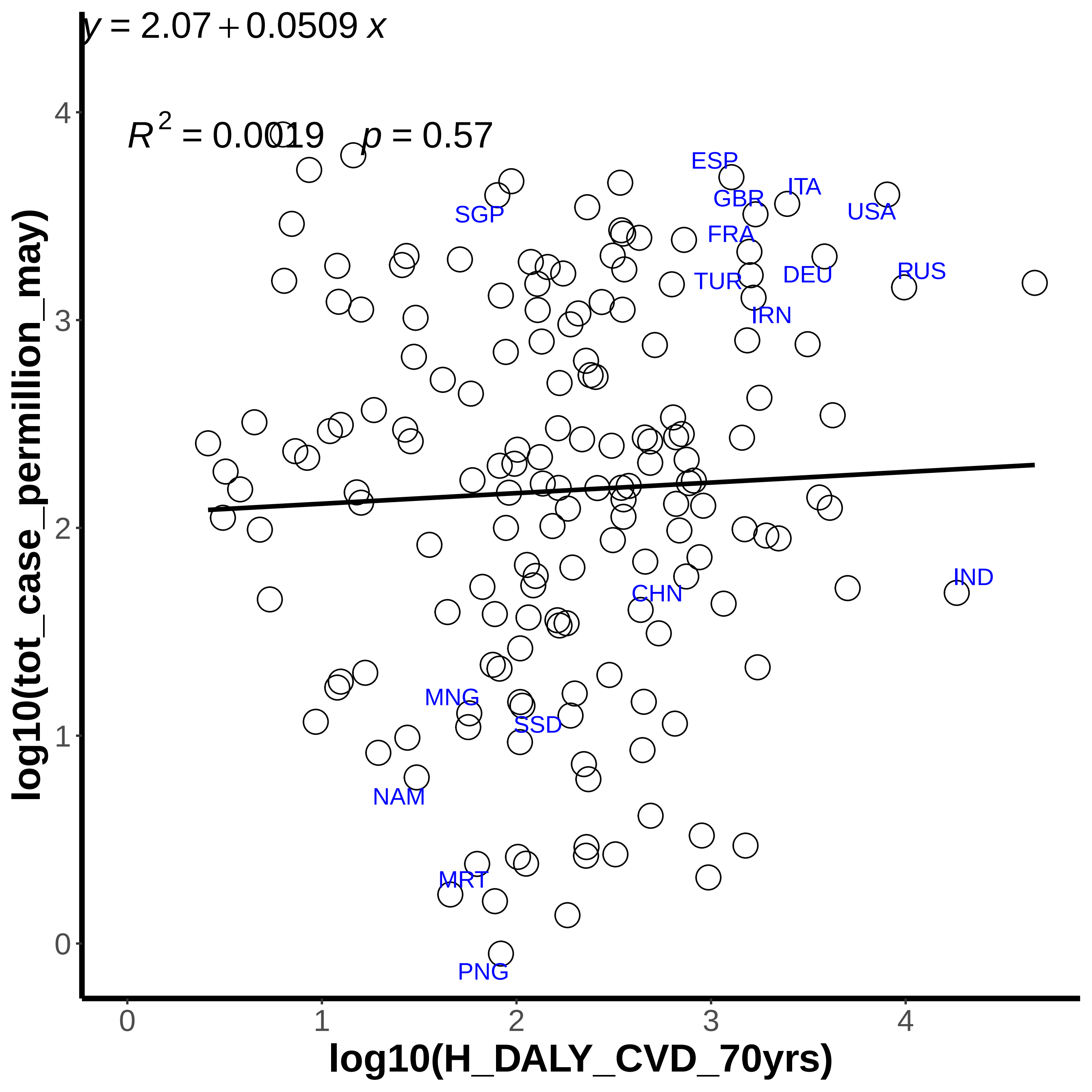

$y = 2.07 + 0.0509\,x$
$R^2 = 0.0019 \quad p = 0.57$
ESP
ITA
GBR
USA
SGP
FRA
TUR
DEU
RUS
IRN
IND
CHN
MNG
SSD
NAM
MRT
PNG
log10(tot_case_permillion_may)
log10(H_DALY_CVD_70yrs)

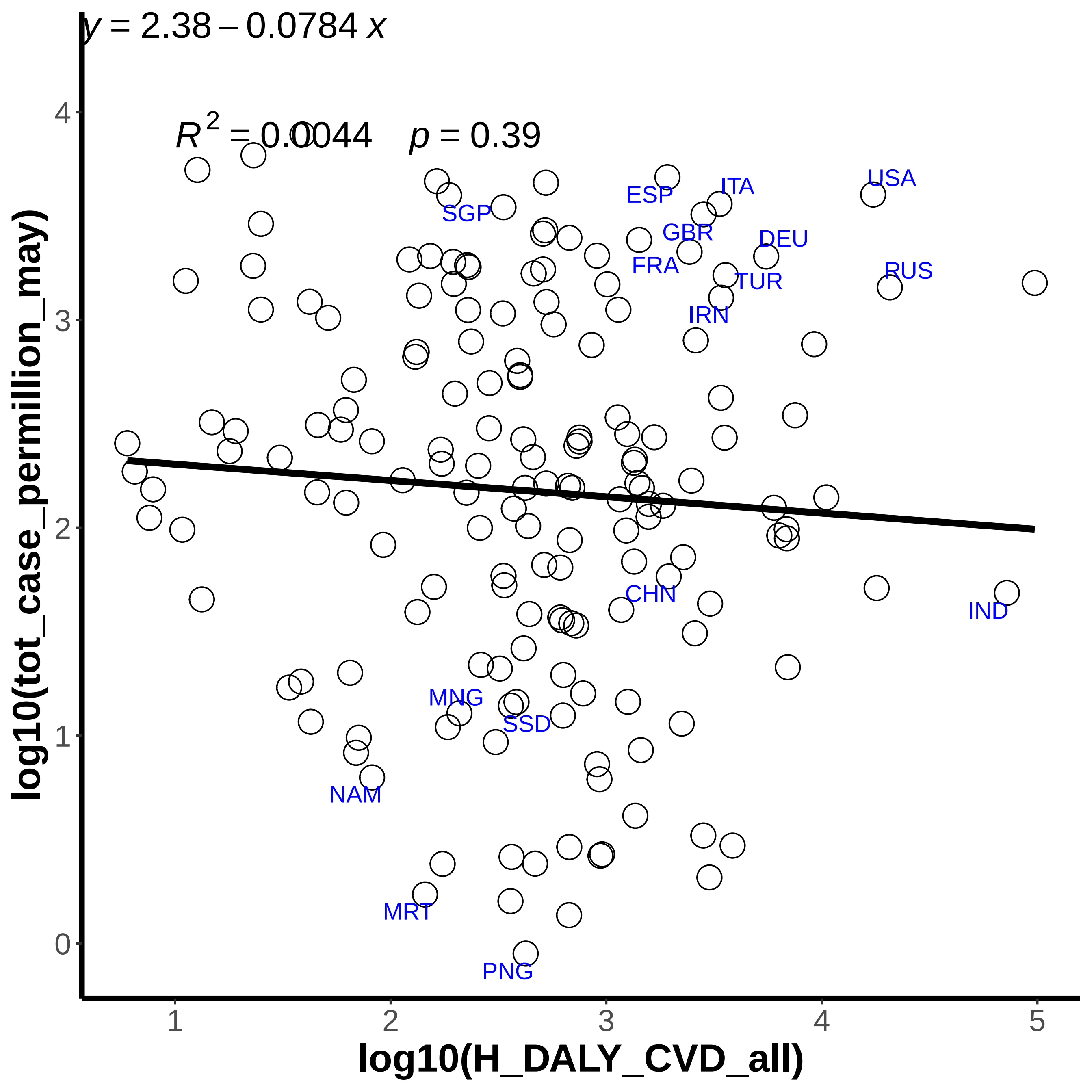

y = 2.38 – 0.0784 x
R^2 = 0.0044   p = 0.39
USA
ITA
ESP
SGP
GBR
DEU
FRA
RUS
TUR
IRN
CHN
IND
MNG
SSD
NAM
MRT
PNG
4
3
2
1
0
1
2
3
4
5
log10(tot_case_permillion_may)
log10(H_DALY_CVD_all)

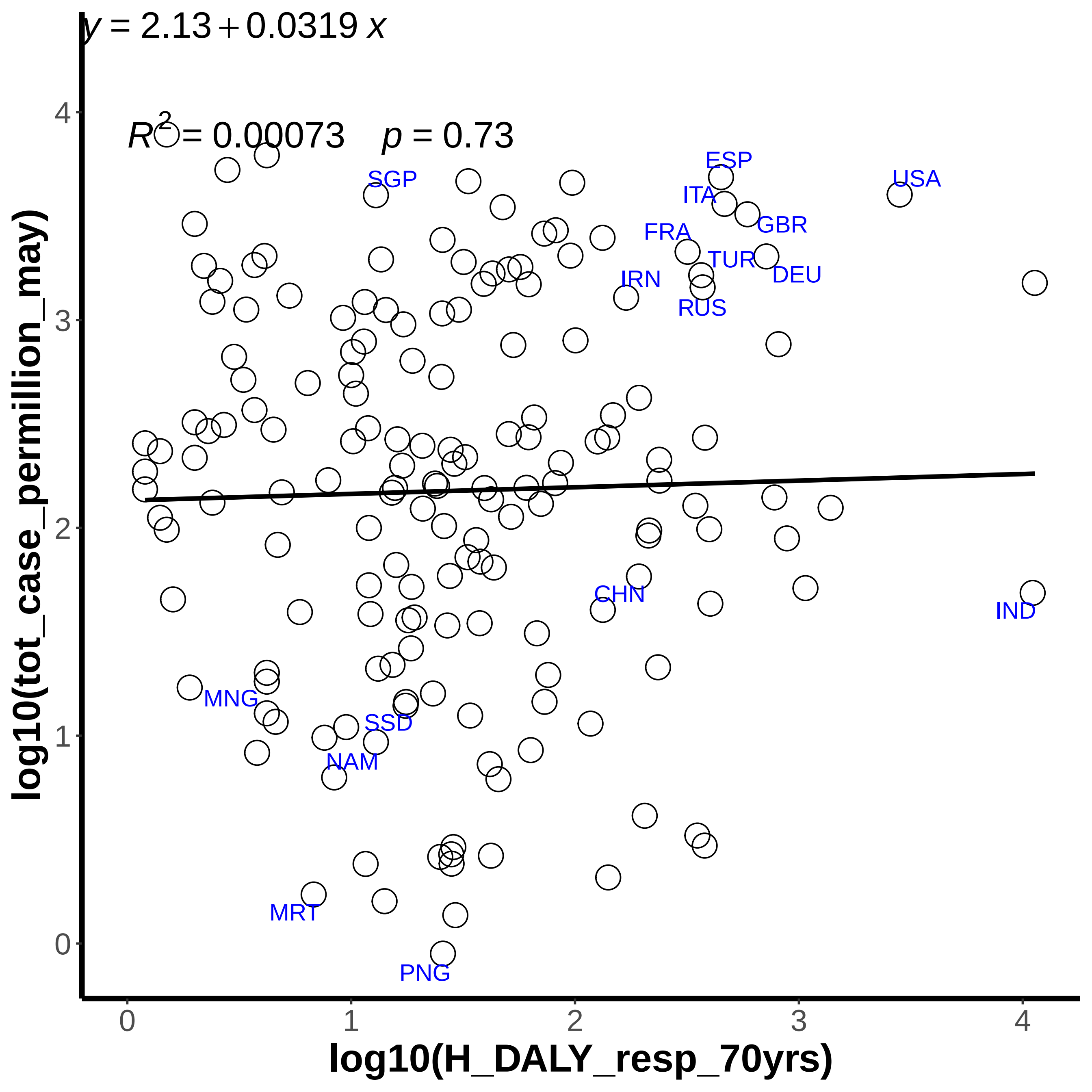

y = 2.13 + 0.0319 x
R2 = 0.00073   p = 0.73
SGP
ESP
USA
ITA
GBR
FRA
TUR
DEU
IRN
RUS
CHN
IND
MNG
SSD
NAM
MRT
PNG
log10(tot_case_permillion_may)
log10(H_DALY_resp_70yrs)
0
1
2
3
4

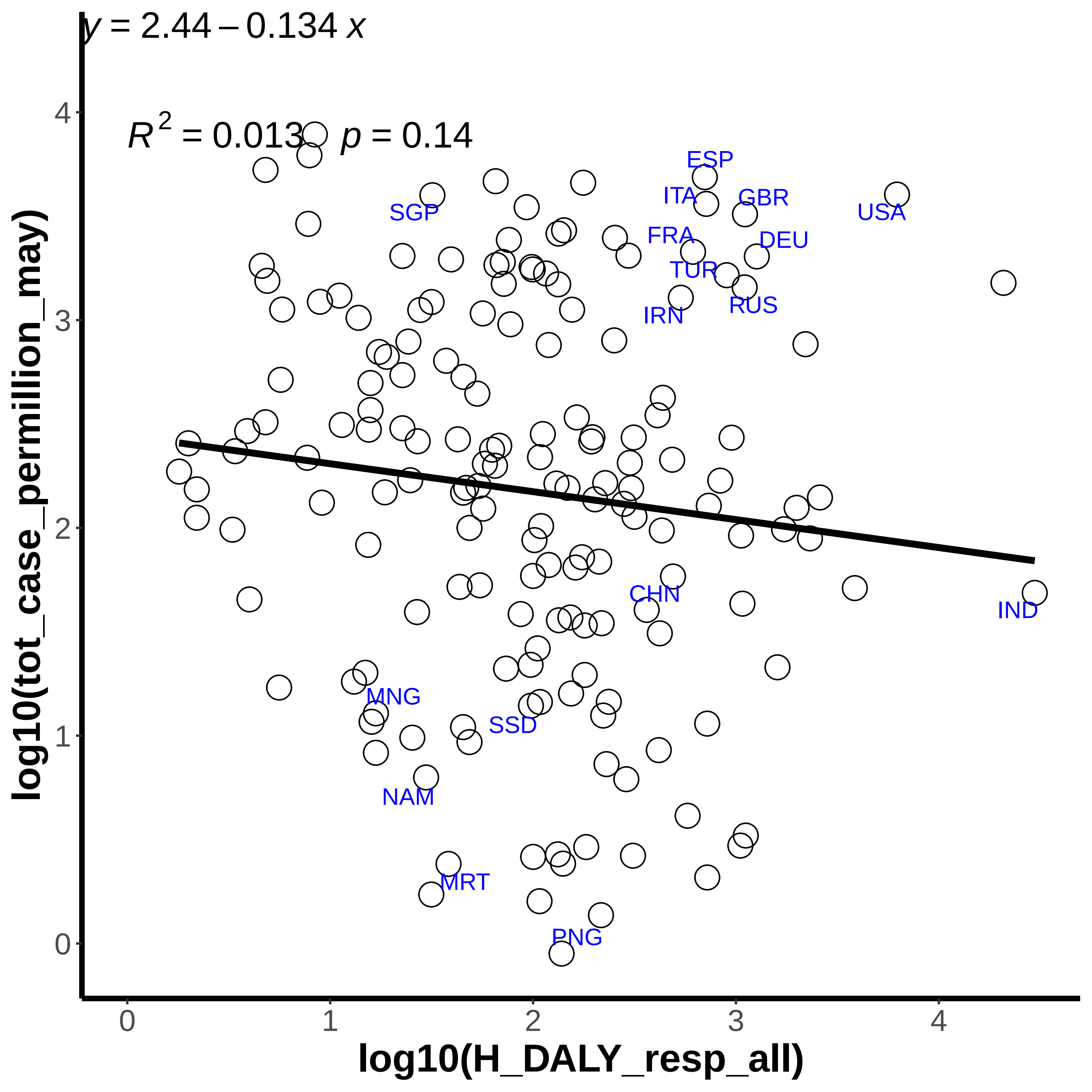
$y = 2.44 - 0.134\,x$
$R^2 = 0.013 \quad p = 0.14$
ESP
ITA
GBR
USA
SGP
FRA
DEU
TUR
RUS
IRN
CHN
IND
MNG
SSD
NAM
MRT
PNG
0
1
2
3
4
log10(tot_case_permillion_may)
log10(H_DALY_resp_all)

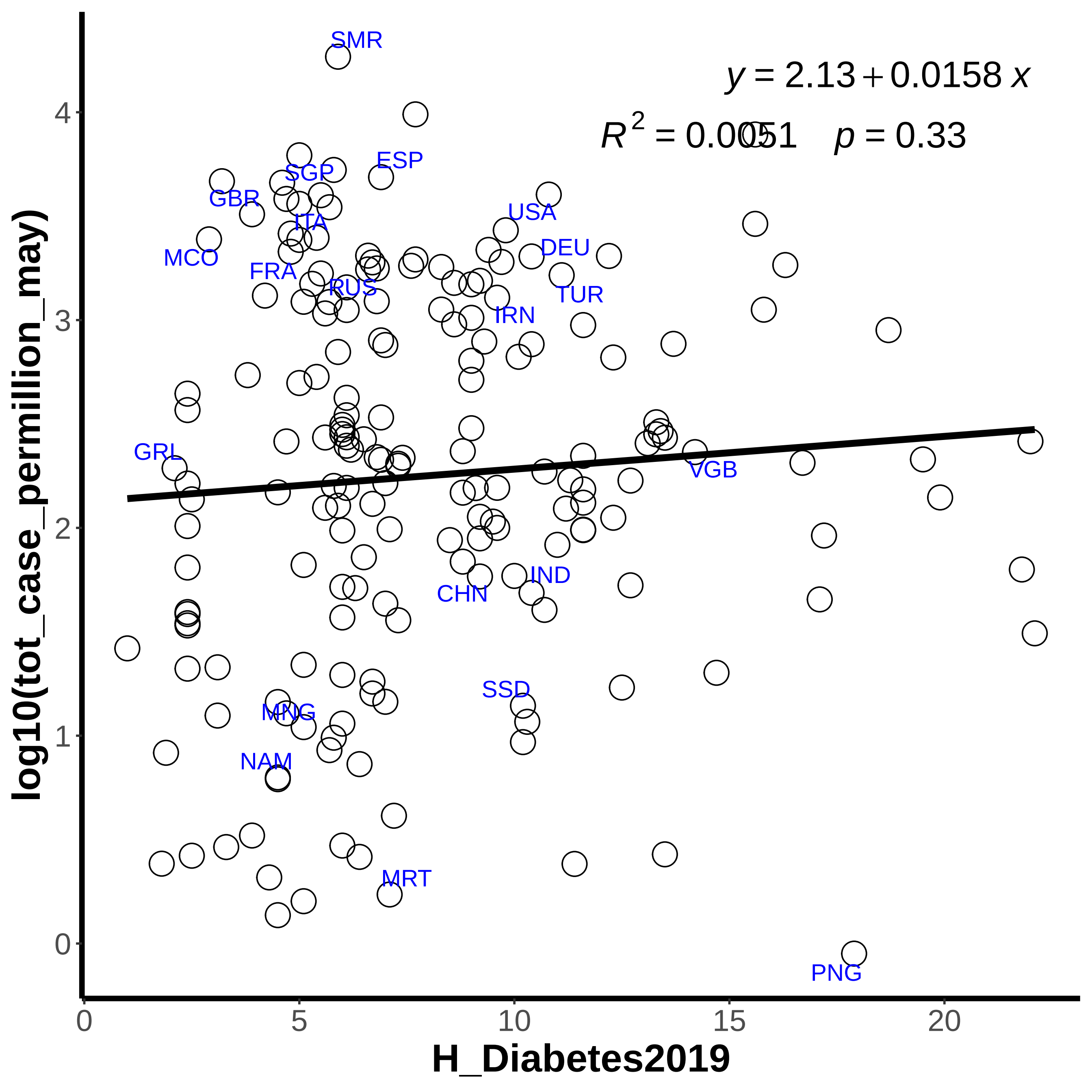

$y = 2.13 + 0.0158\,x$
$R^2 = 0.0051 \quad p = 0.33$
SMR
ESP
SGP
GBR
ITA
USA
MCO
DEU
FRA
RUS
TUR
IRN
GRL
VGB
IND
CHN
SSD
MNG
NAM
MRT
PNG
log10(tot_case_permillion_may)
H_Diabetes2019
0
1
2
3
4
0
5
10
15
20

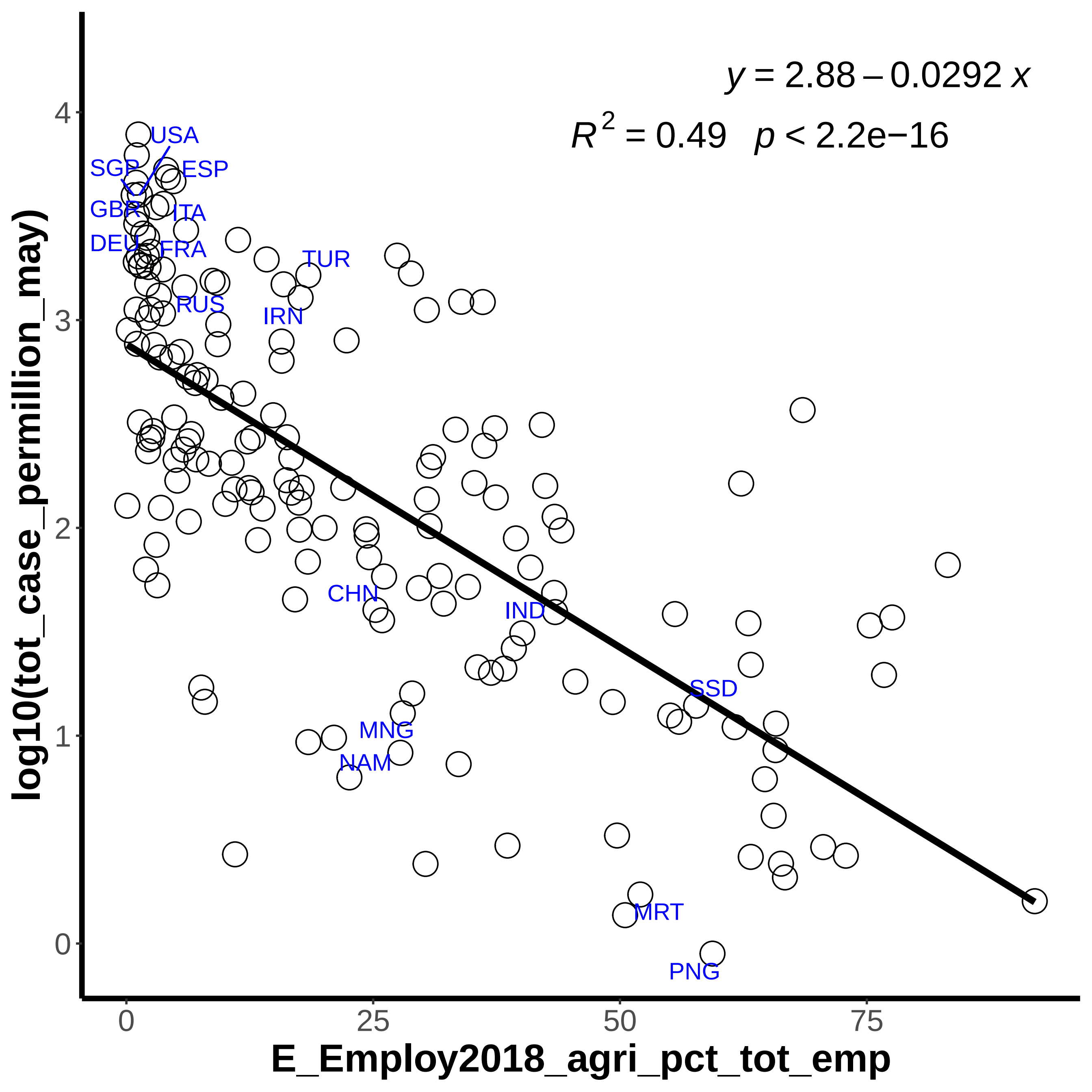

$y = 2.88 - 0.0292\,x$
$R^2 = 0.49 \quad p < 2.2e-16$
log10(tot_case_permillion_may)
E_Employ2018_agri_pct_tot_emp
0
1
2
3
4
0
25
50
75
USA
SGP
ESP
GBR
ITA
DEU
FRA
TUR
RUS
IRN
CHN
IND
SSD
MNG
NAM
MRT
PNG

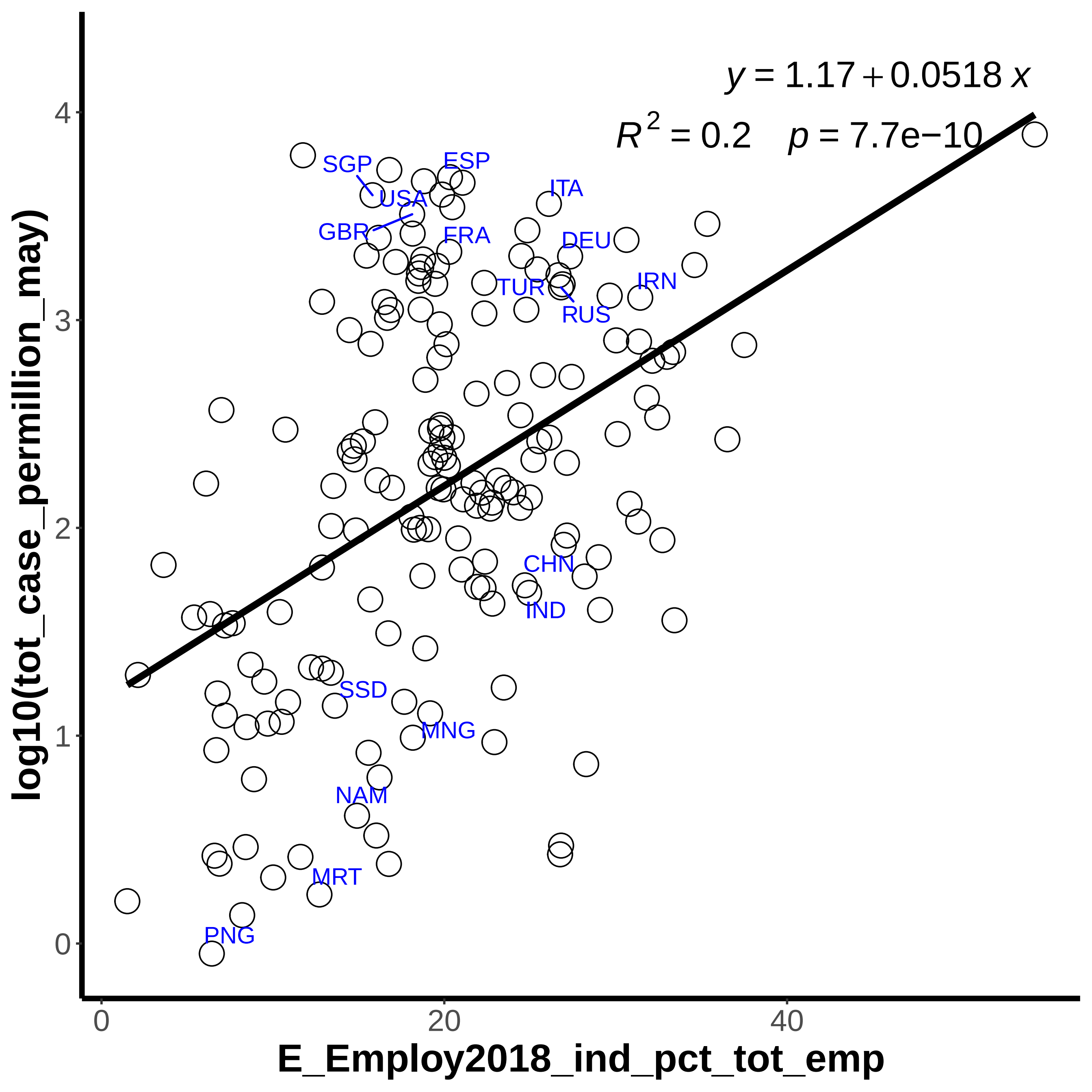

$y = 1.17 + 0.0518\,x$
$R^2 = 0.2 \quad p = 7.7e{-}10$
log10(tot_case_permillion_may)
E_Employ2018_ind_pct_tot_emp
0
1
2
3
4
0
20
40
SGP
ESP
USA
ITA
GBR
FRA
DEU
IRN
TUR
RUS
CHN
IND
SSD
MNG
NAM
MRT
PNG

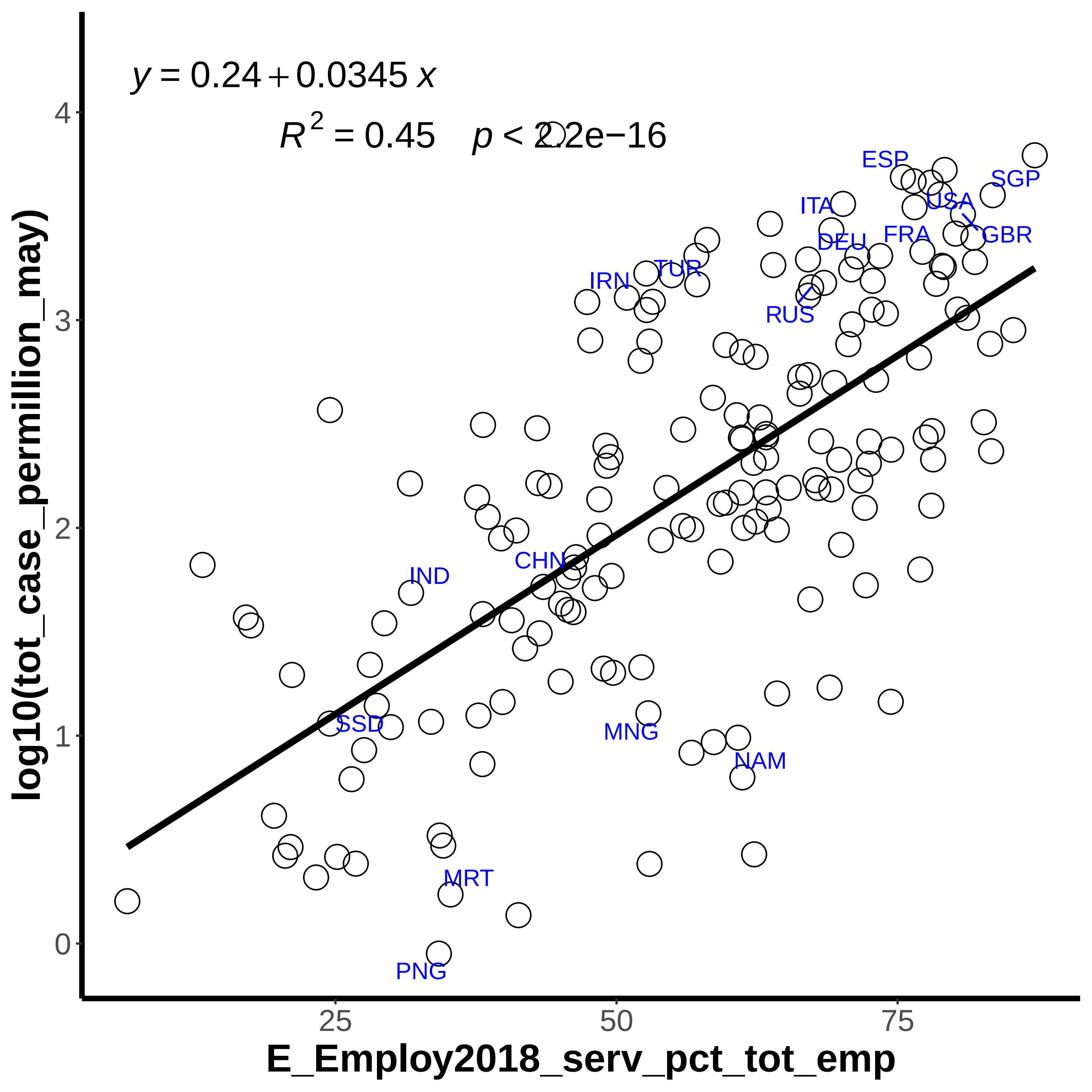

$y = 0.24 + 0.0345\,x$
$R^2 = 0.45 \quad p < 2.2e{-16}$
ESP
SGP
ITA
USA
GBR
FRA
DEU
TUR
IRN
RUS
CHN
IND
SSD
MNG
NAM
MRT
PNG
4
3
2
1
0
25
50
75
log10(tot_case_permillion_may)
E_Employ2018_serv_pct_tot_emp

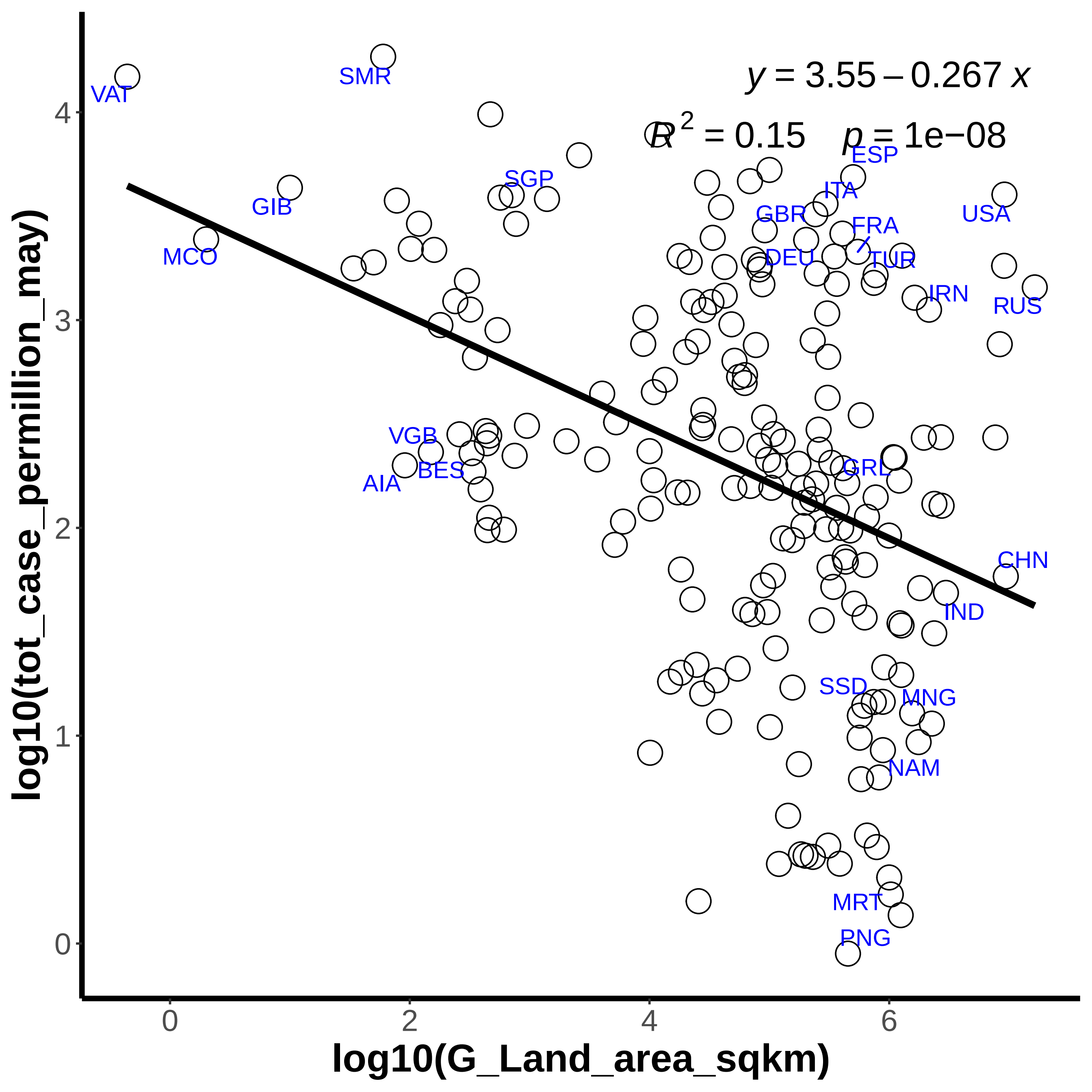

$y = 3.55 - 0.267\,x$
$R^2 = 0.15 \quad p = 1e{-}08$
VAT
SMR
SGP
GIB
MCO
ESP
ITA
GBR
FRA
USA
DEU
TUR
IRN
RUS
VGB
BES
AIA
GRL
CHN
IND
SSD
MNG
NAM
MRT
PNG
4
3
2
1
0
0
2
4
6
log10(tot_case_permillion_may)
log10(G_Land_area_sqkm)

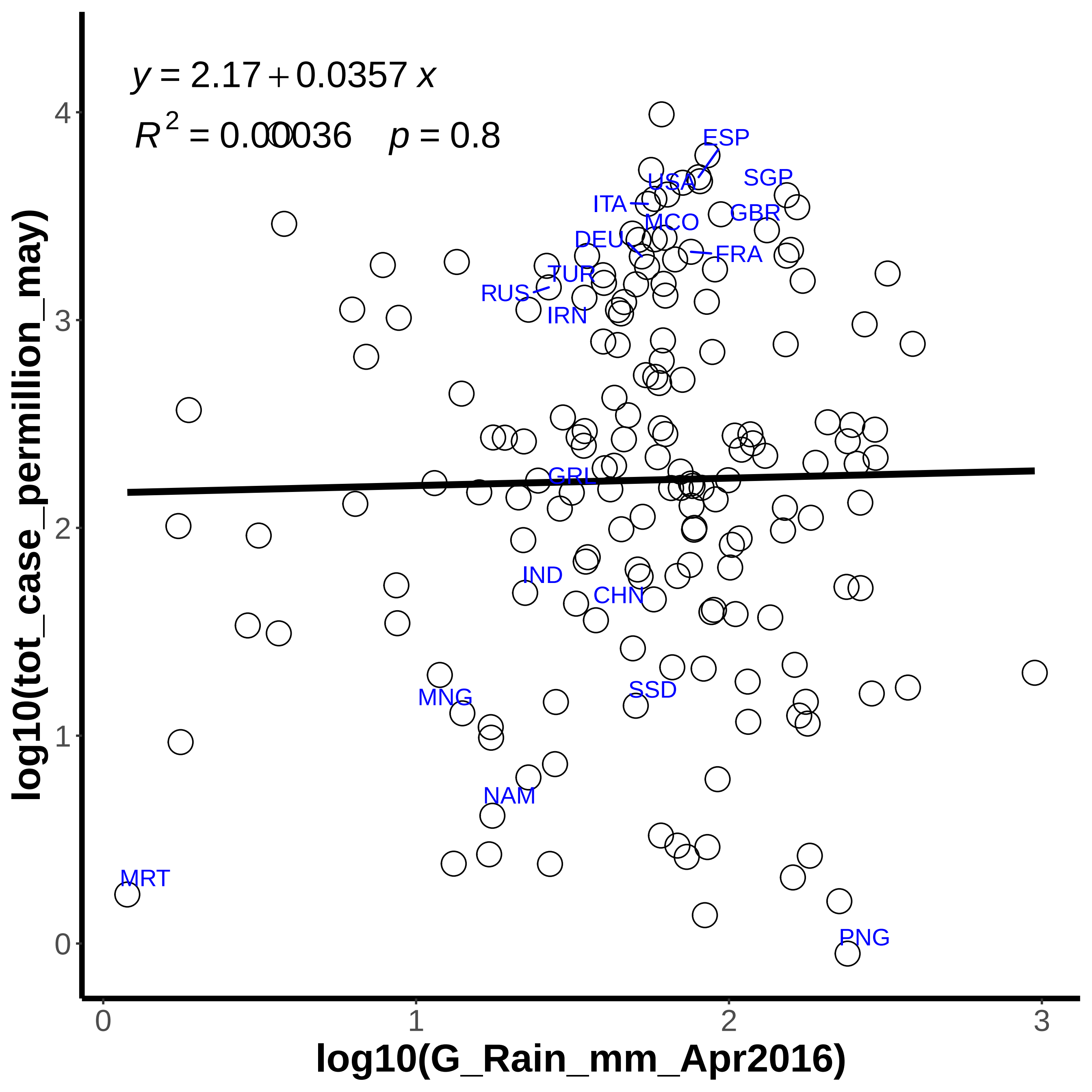

$y = 2.17 + 0.0357\ x$
$R^2 = 0.00036 \quad p = 0.8$
ESP
USA
SGP
ITA
GBR
MCO
DEU
FRA
TUR
RUS
IRN
GRL
IND
CHN
SSD
MNG
NAM
MRT
PNG
0
1
2
3
4
log10(tot_case_permillion_may)
log10(G_Rain_mm_Apr2016)

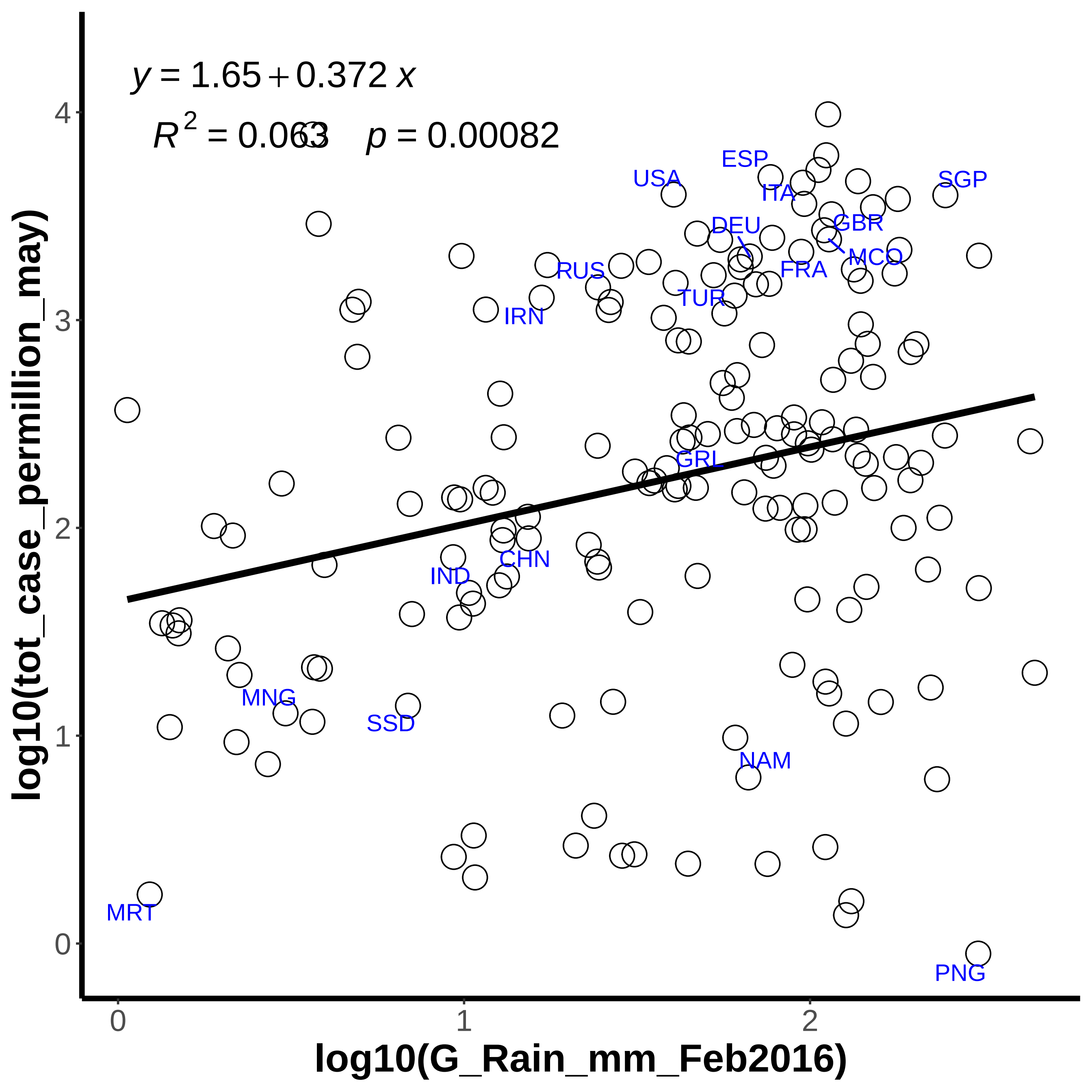

$y = 1.65 + 0.372\,x$
$R^2 = 0.063$ $p = 0.00082$
log10(tot_case_permillion_may)
log10(G_Rain_mm_Feb2016)
0
1
2
3
4
ESP
USA
ITA
SGP
DEU
GBR
MCO
RUS
FRA
TUR
IRN
GRL
CHN
IND
MNG
SSD
NAM
MRT
PNG

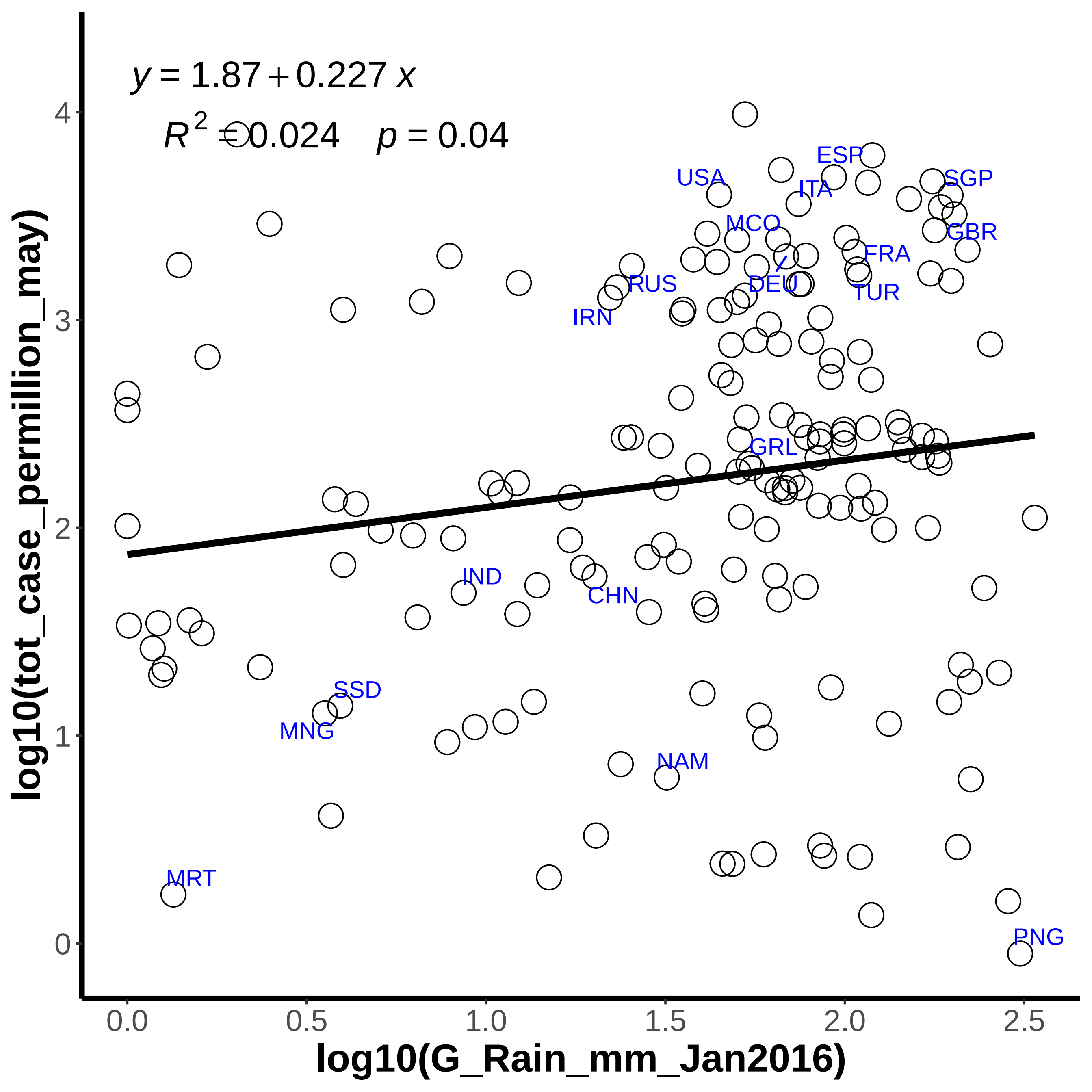

$y = 1.87 + 0.227\,x$
$R^2 = 0.024 \quad p = 0.04$
USA
ESP
ITA
SGP
MCO
GBR
FRA
RUS
DEU
TUR
IRN
GRL
IND
CHN
SSD
MNG
NAM
MRT
PNG
0
1
2
3
4
0.0
0.5
1.0
1.5
2.0
2.5
log10(G_Rain_mm_Jan2016)
log10(tot_case_permillion_may)

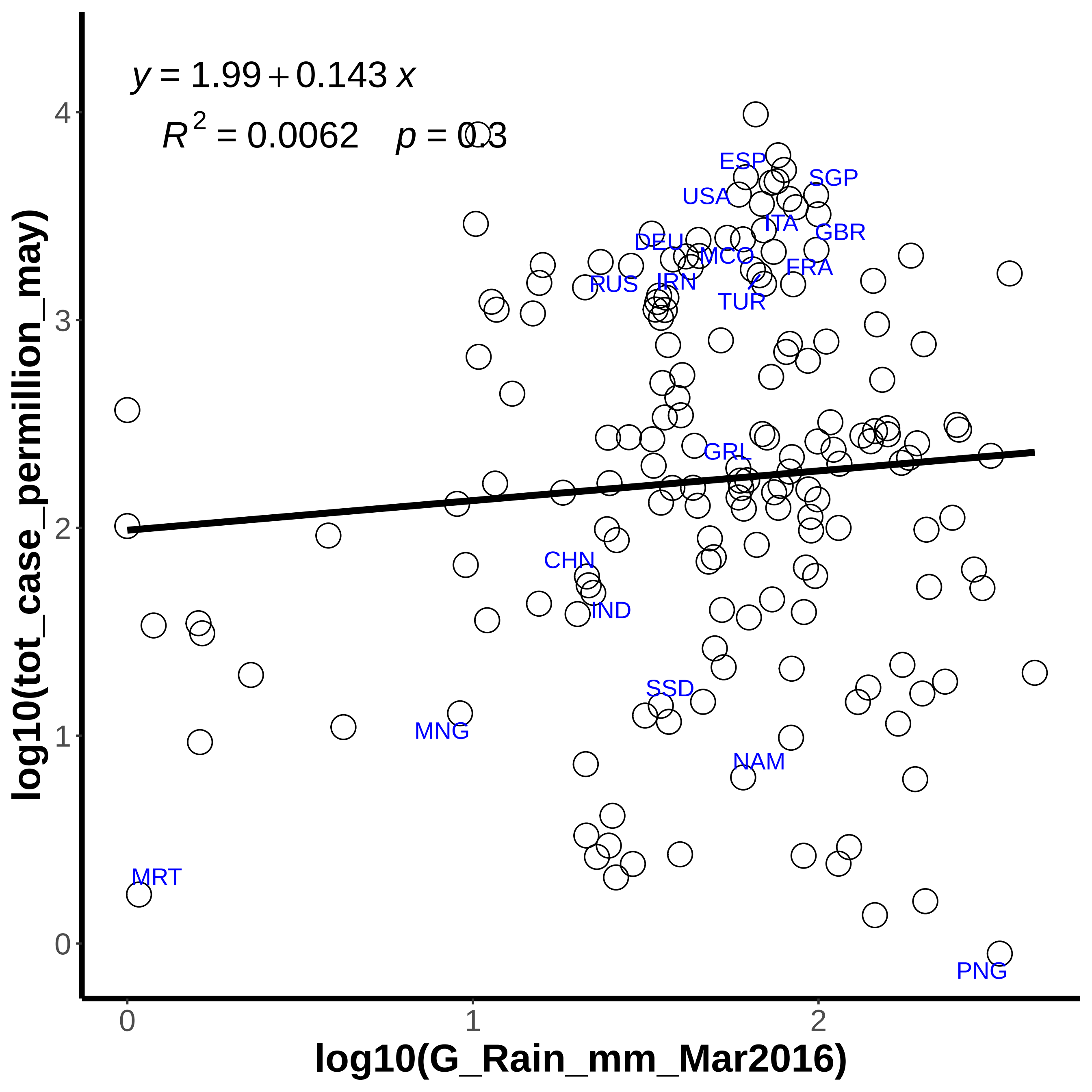

$y = 1.99 + 0.143\,x$
$R^2 = 0.0062 \quad p = 0.3$
ESP
SGP
USA
ITA
GBR
DEU
MCO
FRA
RUS
IRN
TUR
GRL
CHN
IND
SSD
MNG
NAM
MRT
PNG
log10(tot_case_permillion_may)
log10(G_Rain_mm_Mar2016)

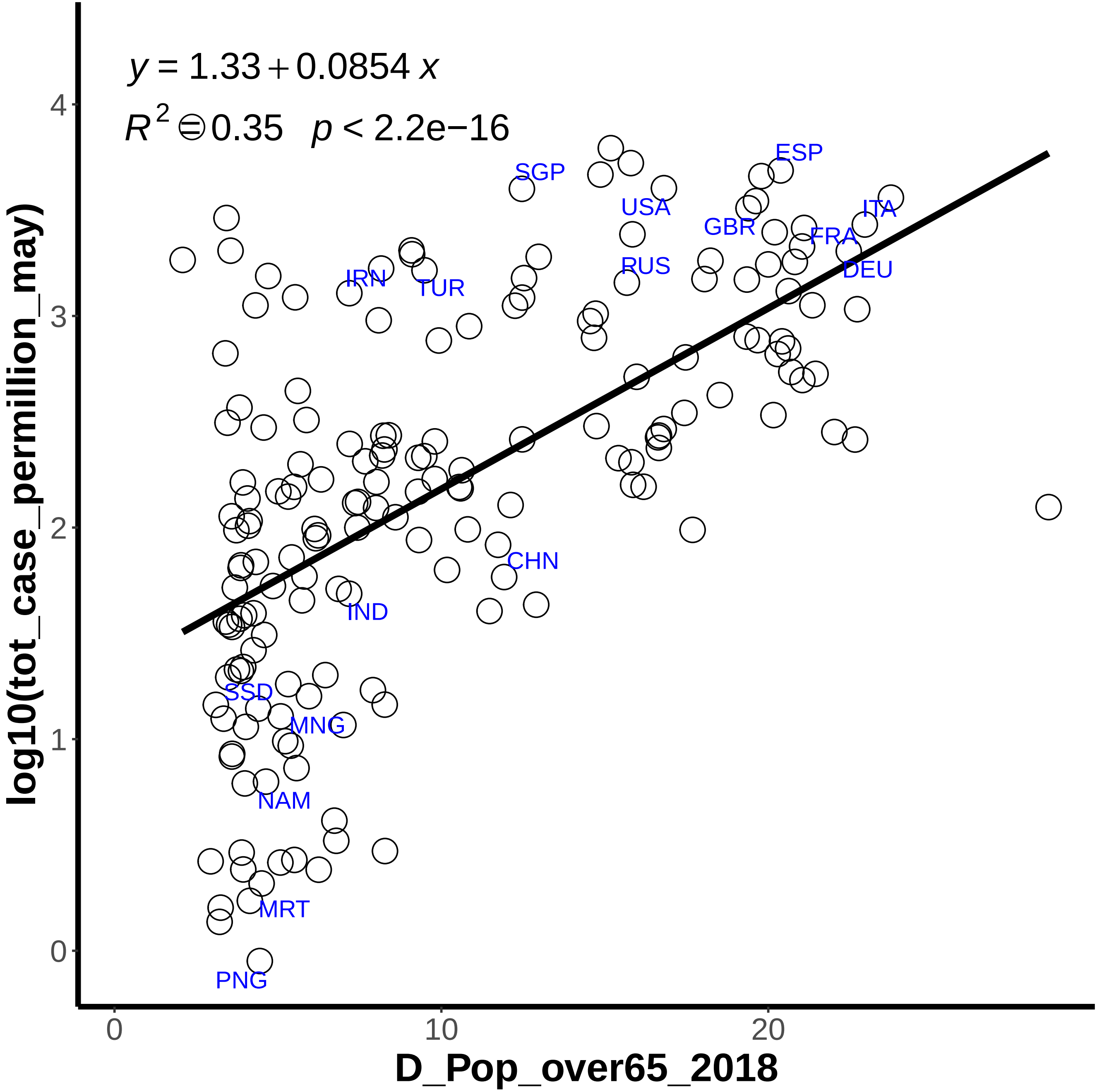

$y = 1.33 + 0.0854\,x$
$R^2 = 0.35 \quad p < 2.2e-16$
log10(tot_case_permillion_may)
D_Pop_over65_2018
0
1
2
3
4
0
10
20
SGP
ESP
USA
ITA
GBR
FRA
IRN
TUR
RUS
DEU
CHN
IND
SSD
MNG
NAM
MRT
PNG

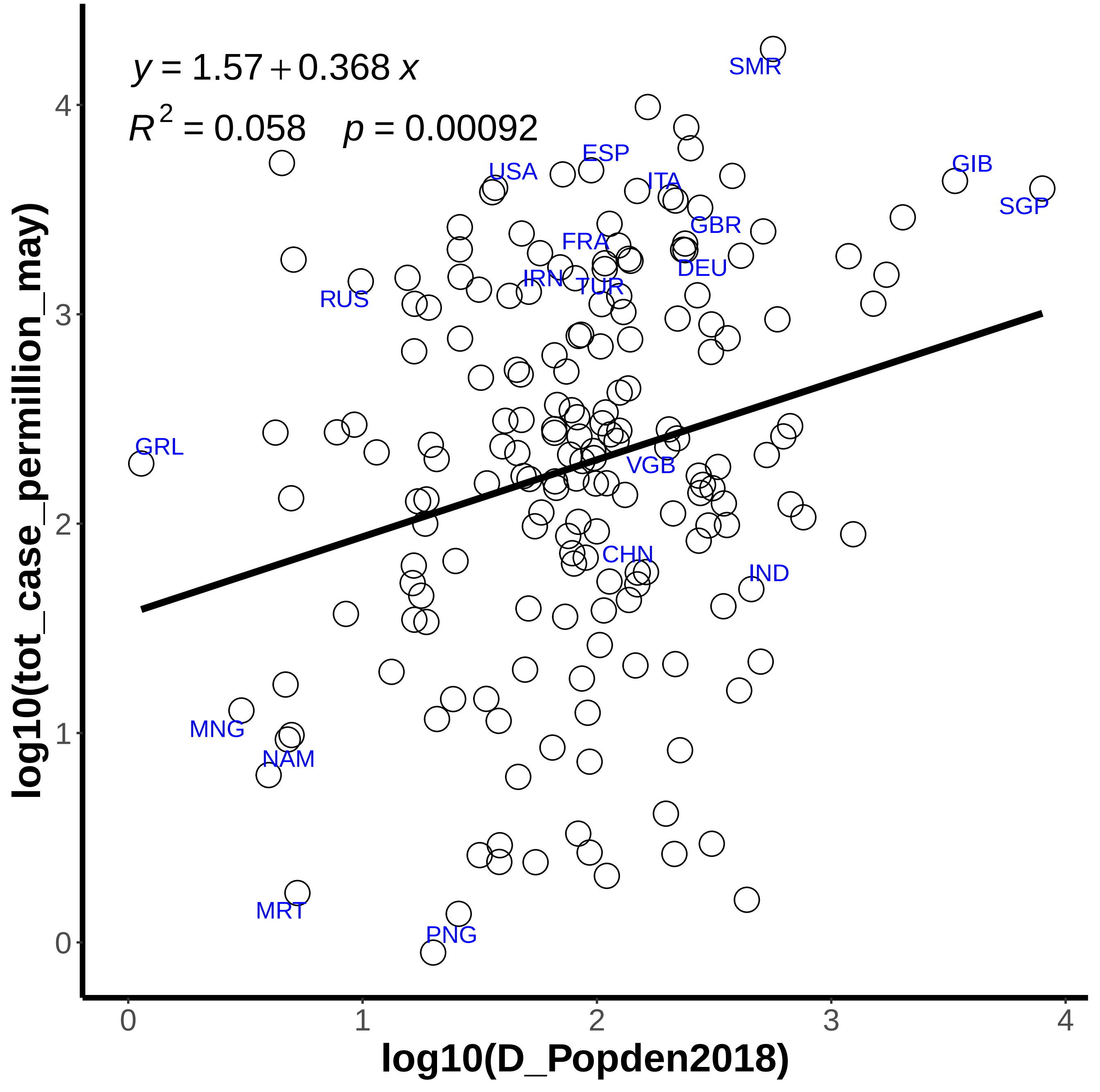

$y = 1.57 + 0.368\,x$
$R^2 = 0.058 \quad p = 0.00092$
SMR
ESP
USA
ITA
GIB
SGP
GBR
FRA
IRN
DEU
TUR
RUS
GRL
VGB
CHN
IND
MNG
NAM
MRT
PNG
log10(tot_case_permillion_may)
log10(D_Popden2018)
0
1
2
3
4

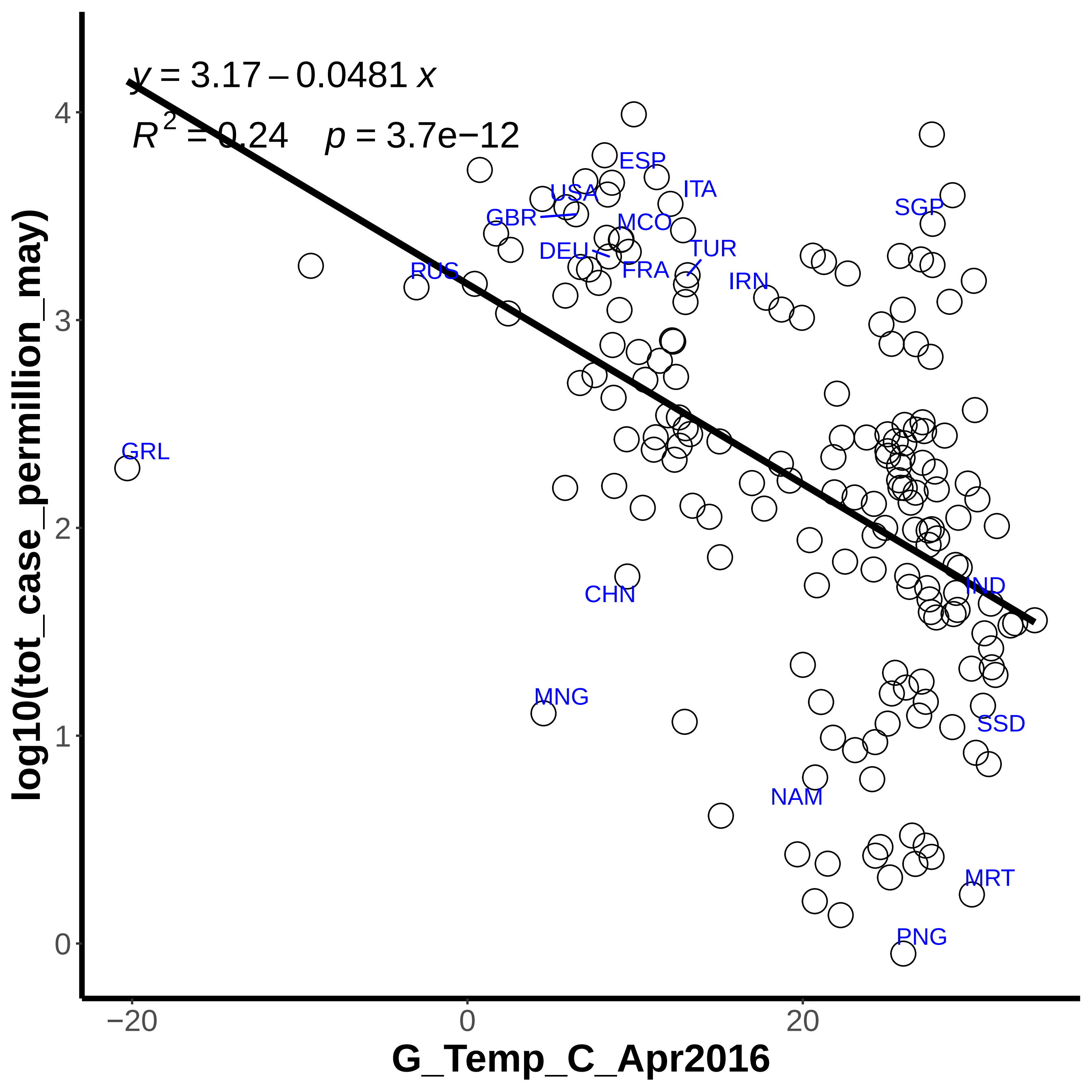

$y = 3.17 - 0.0481\,x$
$R^2 = 0.24 \quad p = 3.7e{-}12$
log10(tot_case_permillion_may)
G_Temp_C_Apr2016
0
1
2
3
4
−20
0
20
GRL
RUS
GBR
USA
DEU
ESP
ITA
MCO
FRA
TUR
IRN
SGP
CHN
MNG
IND
SSD
NAM
MRT
PNG

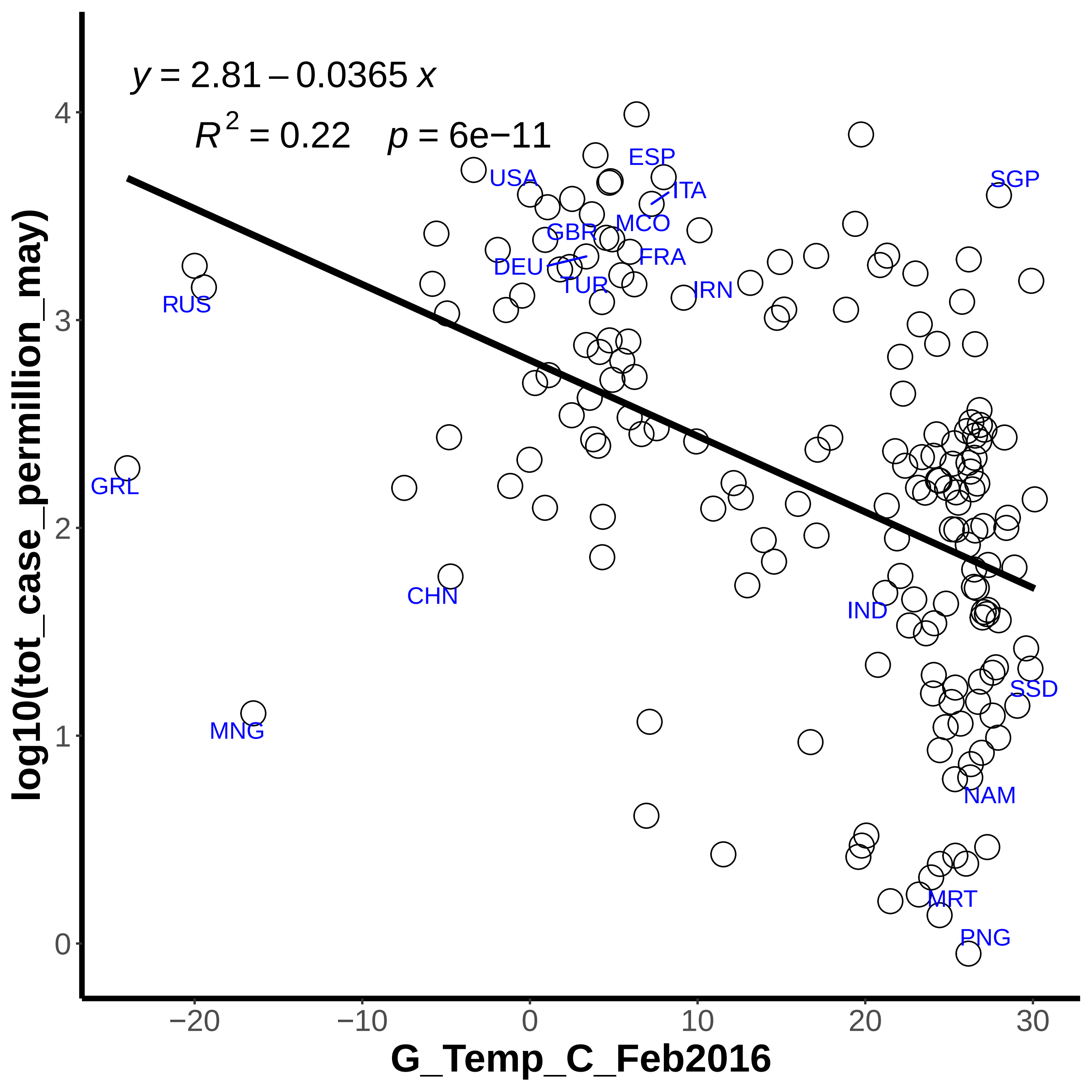

$y = 2.81 - 0.0365\,x$
$R^2 = 0.22 \quad p = 6e{-}11$
USA
ESP
ITA
MCO
GBR
DEU
FRA
TUR
IRN
SGP
RUS
GRL
CHN
IND
MNG
SSD
NAM
MRT
PNG
log10(tot_case_permillion_may)
G_Temp_C_Feb2016
−20
−10
0
10
20
30
0
1
2
3
4

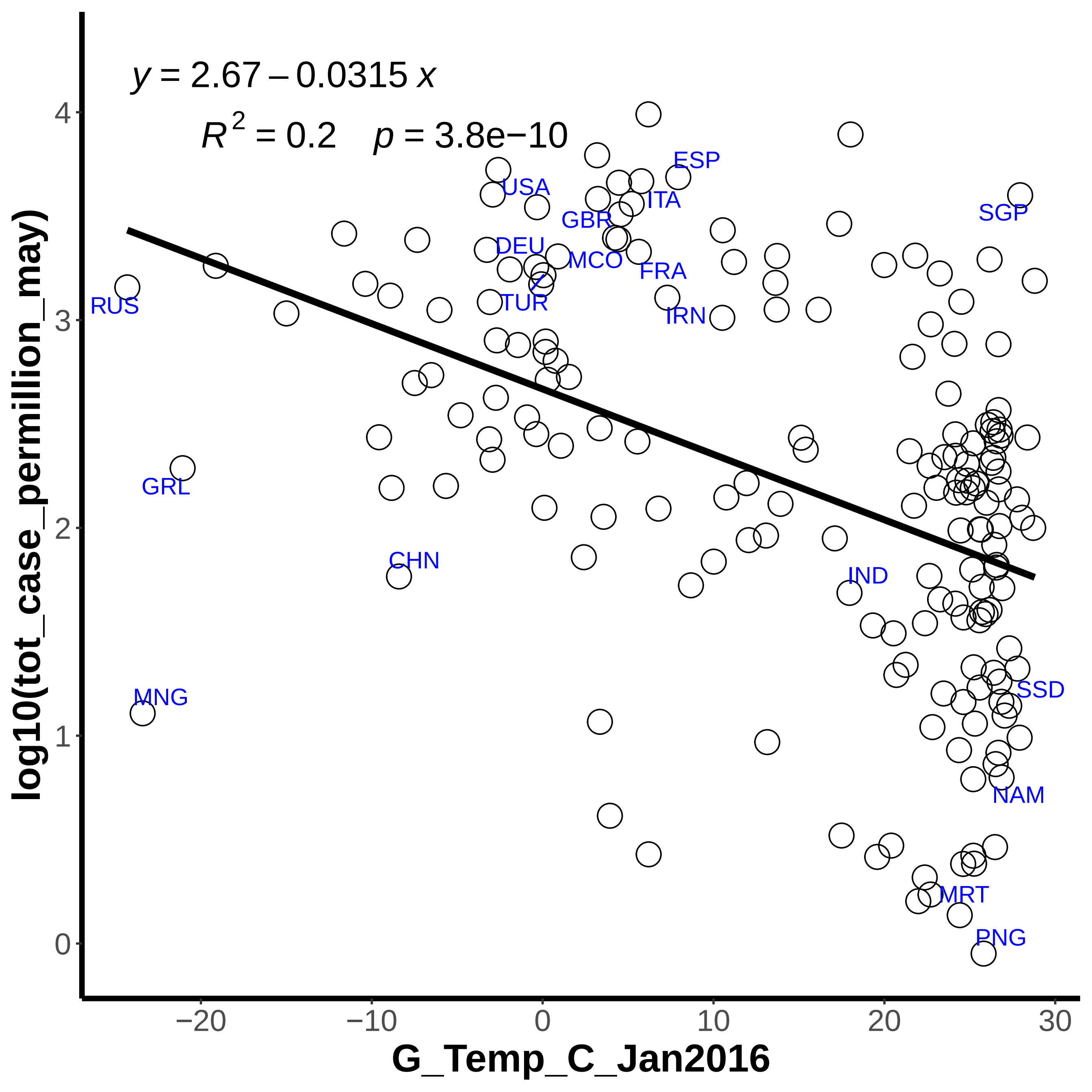
$y = 2.67 - 0.0315\,x$
$R^2 = 0.2 \quad p = 3.8e{-}10$
log10(tot_case_permillion_may)
G_Temp_C_Jan2016
4
3
2
1
0
−20
−10
0
10
20
30
RUS
USA
ESP
ITA
GBR
SGP
DEU
MCO
FRA
TUR
IRN
GRL
CHN
IND
MNG
SSD
NAM
MRT
PNG

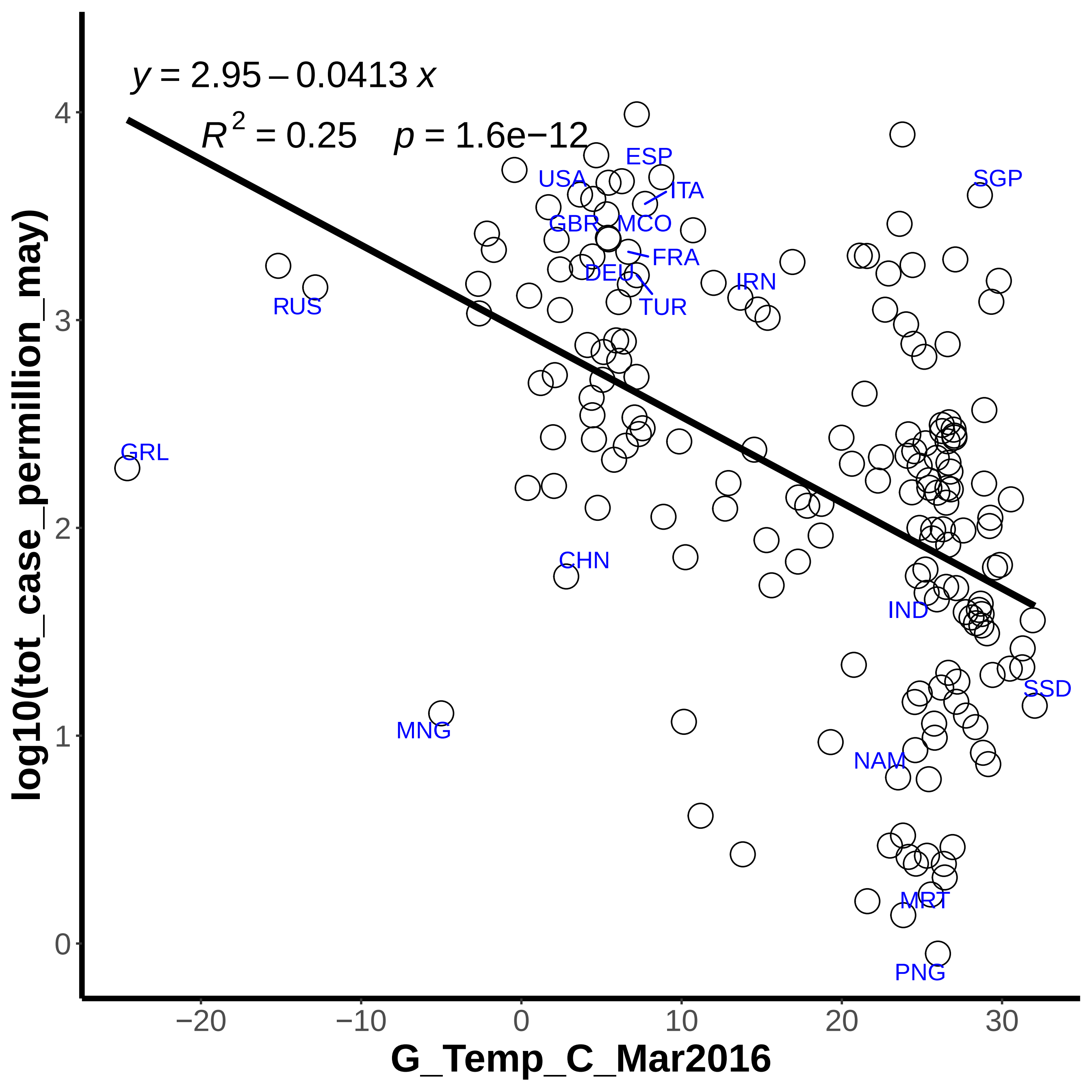

$y = 2.95 - 0.0413\,x$
$R^2 = 0.25 \quad p = 1.6e-12$
ESP
USA
ITA
SGP
GBR
MCO
FRA
DEU
IRN
TUR
RUS
GRL
CHN
IND
SSD
MNG
NAM
MRT
PNG
4
3
2
1
0
−20
−10
0
10
20
30
log10(tot_case_permillion_may)
G_Temp_C_Mar2016

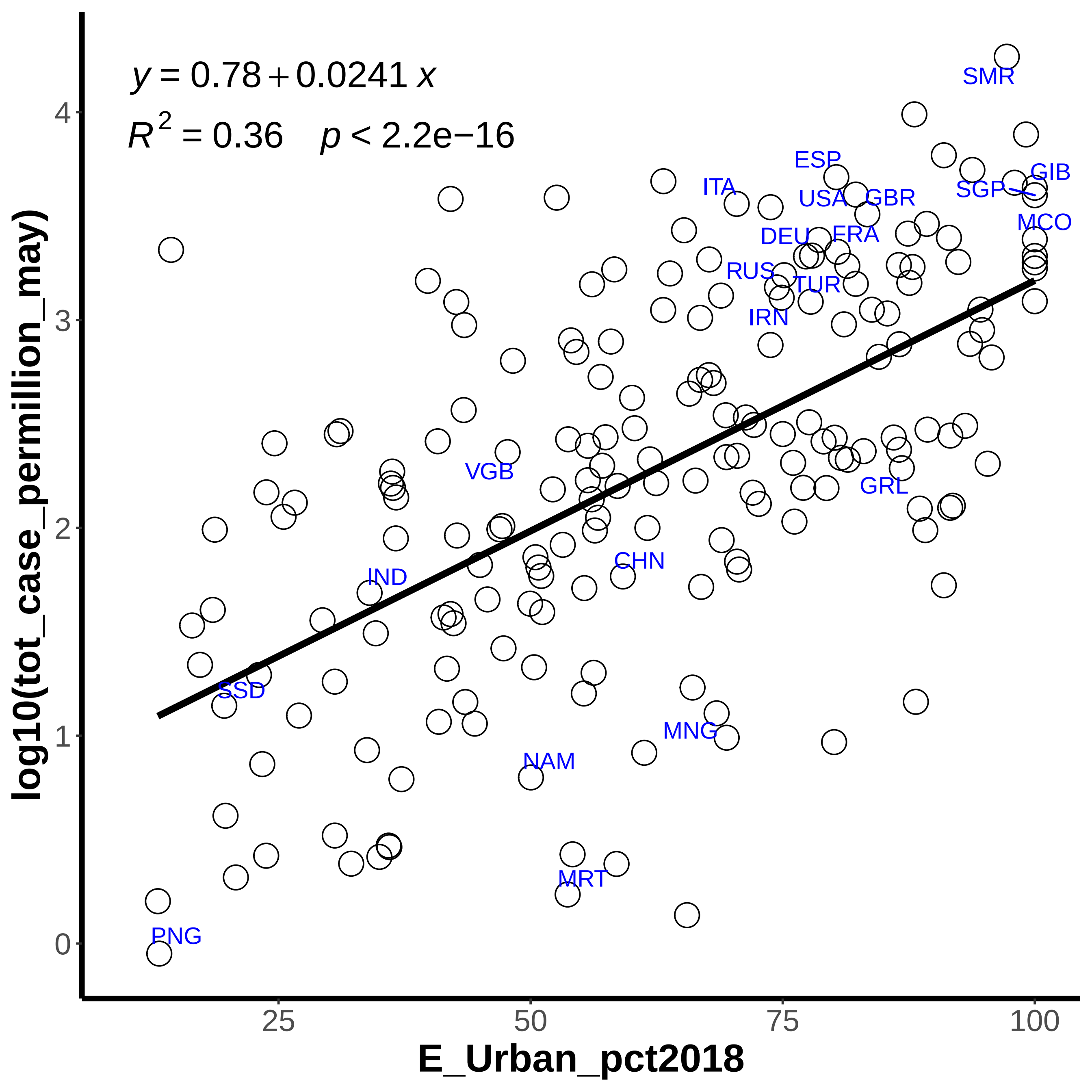

$y = 0.78 + 0.0241\,x$
$R^2 = 0.36 \quad p < 2.2e{-16}$
SMR
GIB
SGP
MCO
ESP
ITA
USA
GBR
DEU
FRA
RUS
TUR
IRN
VGB
GRL
CHN
IND
SSD
MNG
NAM
MRT
PNG
log10(tot_case_permillion_may)
E_Urban_pct2018
0
1
2
3
4
25
50
75
100

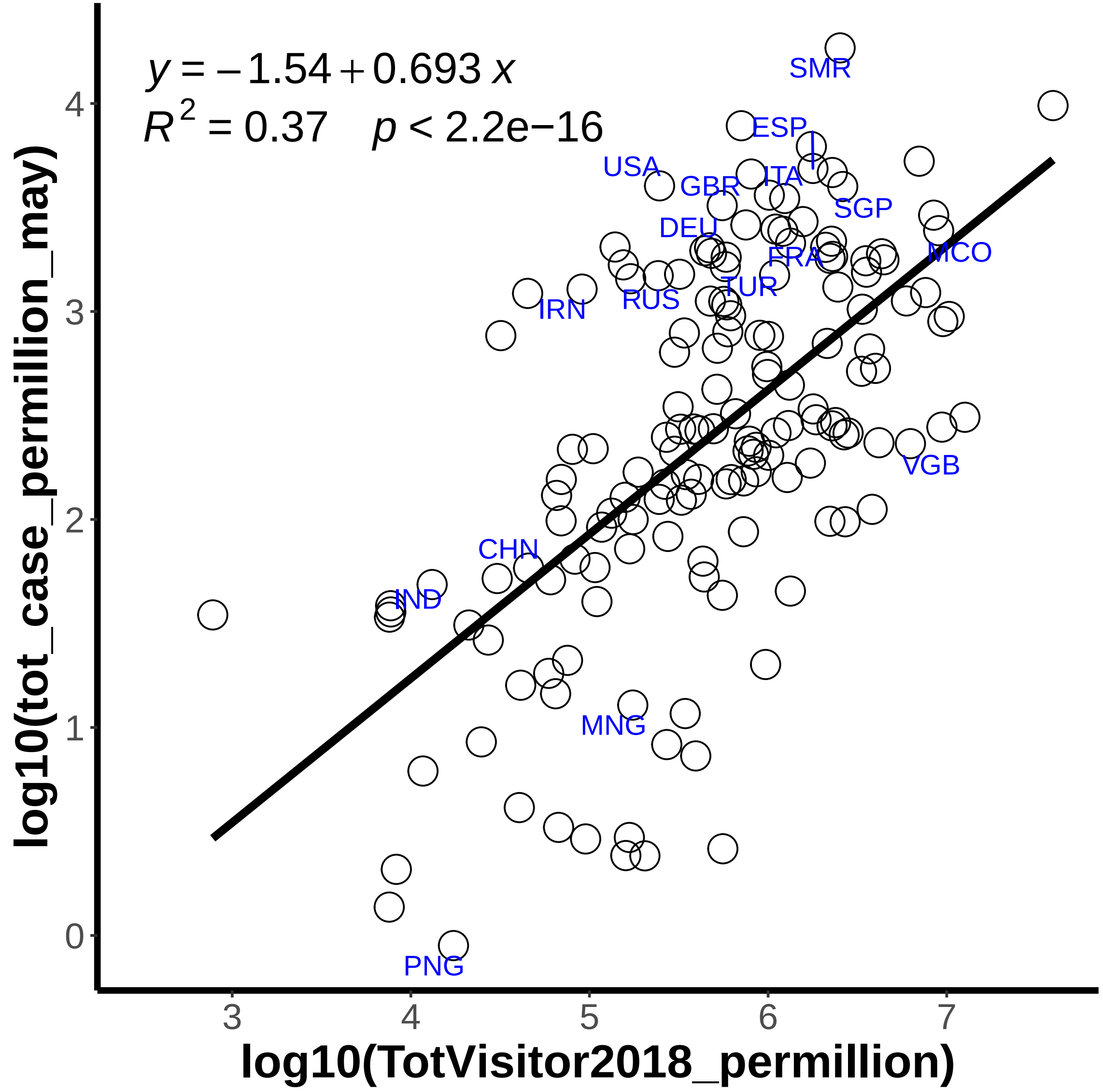

$y = -1.54 + 0.693\,x$
$R^2 = 0.37 \quad p < 2.2e{-16}$
log10(tot_case_permillion_may)
log10(TotVisitor2018_permillion)
0
1
2
3
4
3
4
5
6
7
SMR
ESP
USA
GBR
ITA
SGP
DEU
FRA
MCO
TUR
RUS
IRN
VGB
CHN
IND
MNG
PNG
