# Supplementary material for: Global-scale modeling of early factors and country-specific trajectories of COVID-19 incidence: a cross-sectional study of the first 6 months of the pandemic
Source: BMC Public Health. 2022 Oct 14;22:1919. doi: 10.1186/s12889-022-14336-w (PMC9568998; doi:10.1186/s12889-022-14336-w)
Supplement: Supplementary file 7 — Additional file 7. Country-specific trajectories of new COVID-19 cases around lockdown periods until June 30, 2020. [file 12889_2022_14336_MOESM7_ESM.pdf]

Additional File 7. Country-specific trajectories of new COVID-19 cases around lockdown periods until June 30, 2020. In each graph, the line joining the points is simply to make the order of the points more evident, and does not relate to any fitting of the datapoints. The days are plotted on the x-axis (as dates) and the number of reported new cases are plotted on the y-axis. The start and end dates of the lockdown are indicated by blue vertical lines.

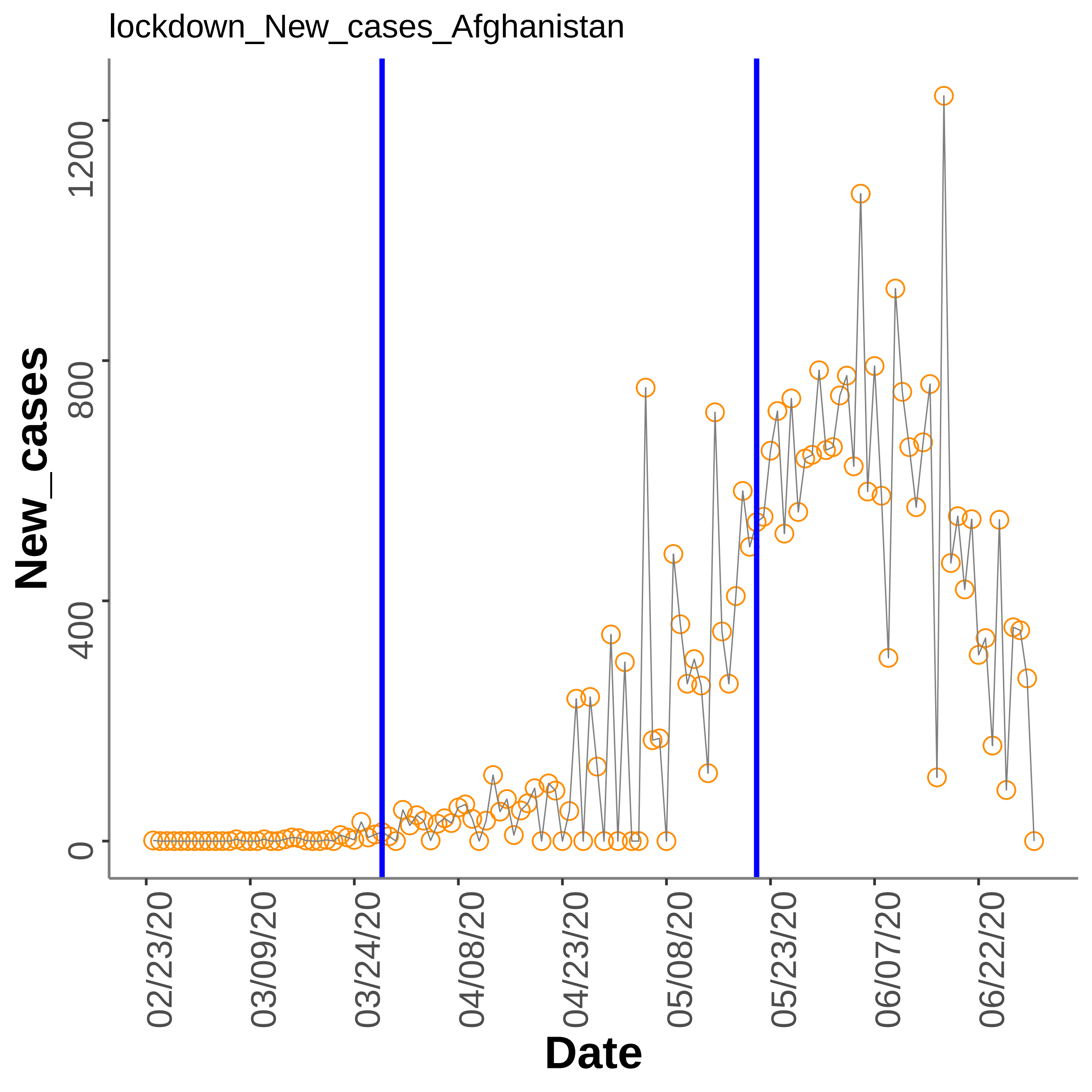

lockdown_New_cases_Afghanistan
New_cases
0
400
800
1200
Date
02/23/20
03/09/20
03/24/20
04/08/20
04/23/20
05/08/20
05/23/20
06/07/20
06/22/20

lockdown_New_cases_Albania

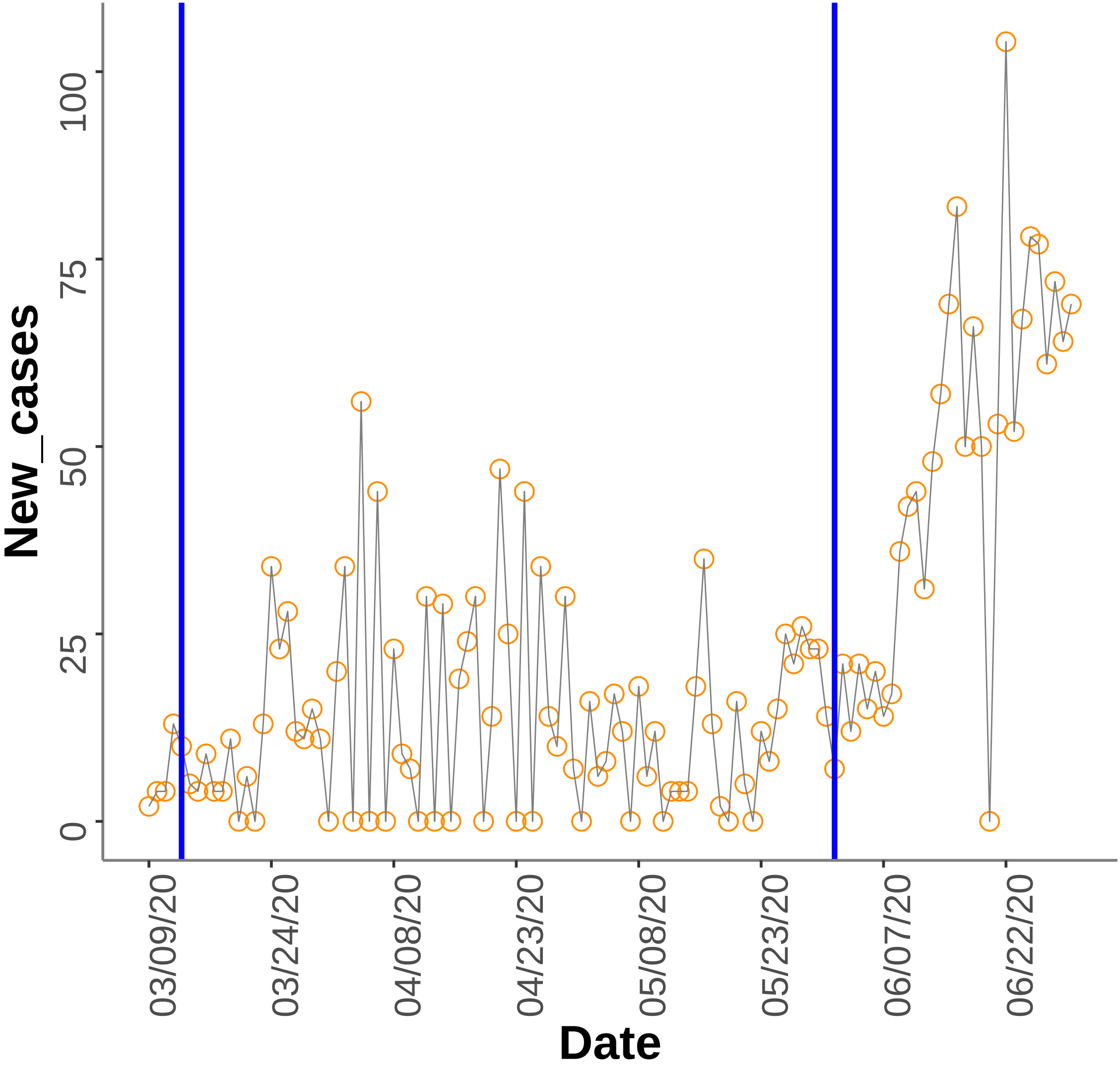

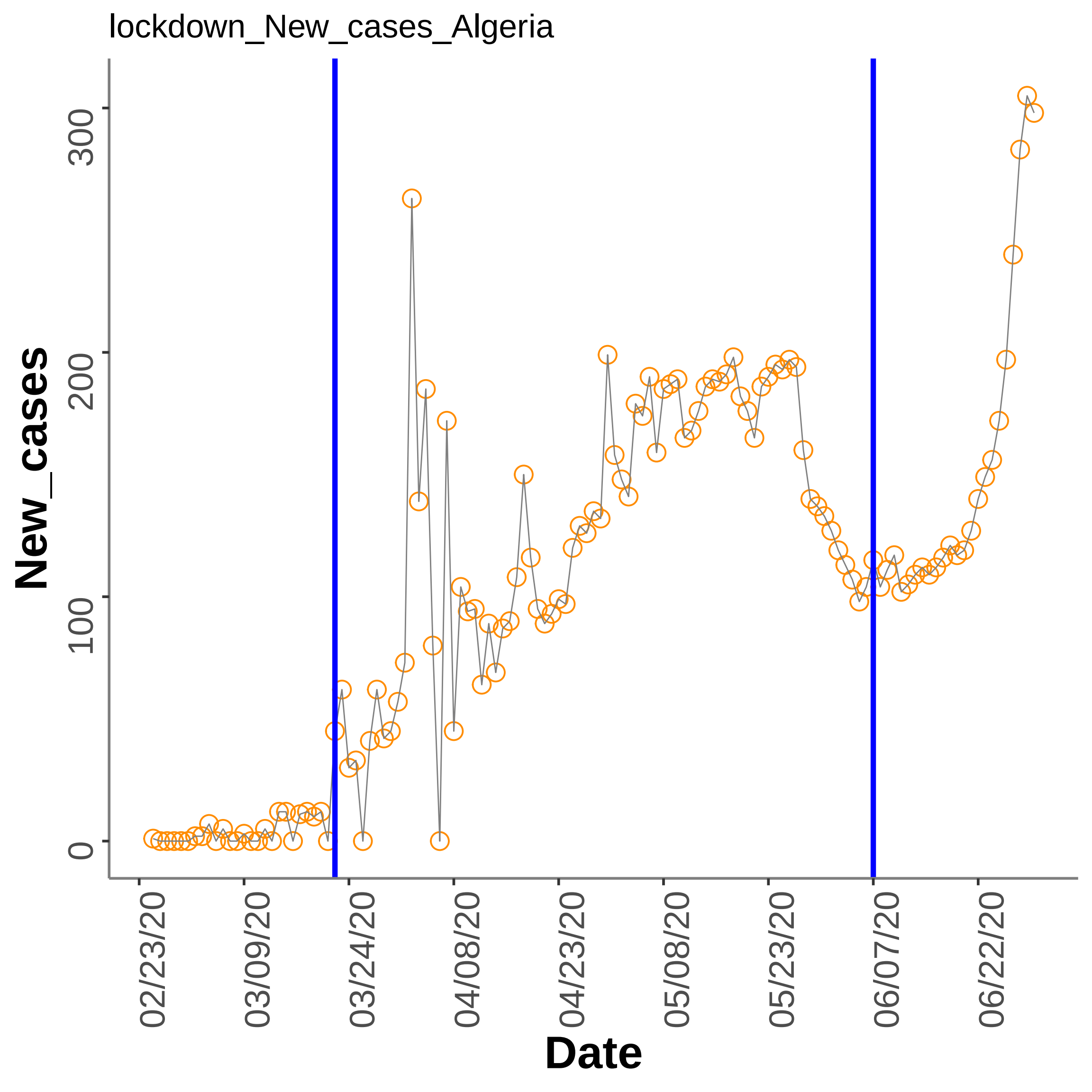
lockdown_New_cases_Algeria
New_cases
300
200
100
0
02/23/20
03/09/20
03/24/20
04/08/20
04/23/20
05/08/20
05/23/20
06/07/20
06/22/20
Date

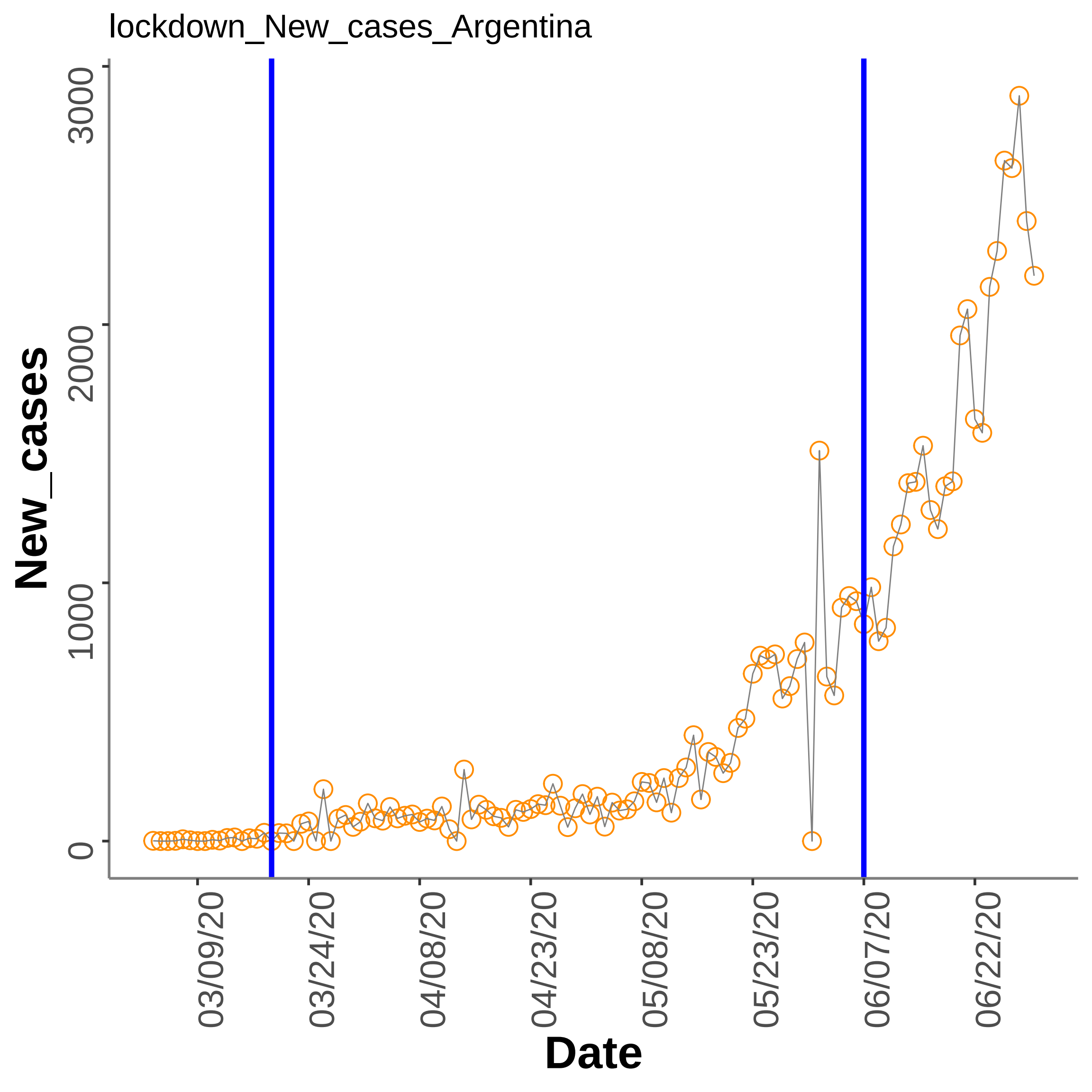

lockdown_New_cases_Argentina
New_cases
3000
2000
1000
0
03/09/20
03/24/20
04/08/20
04/23/20
05/08/20
05/23/20
06/07/20
06/22/20
Date

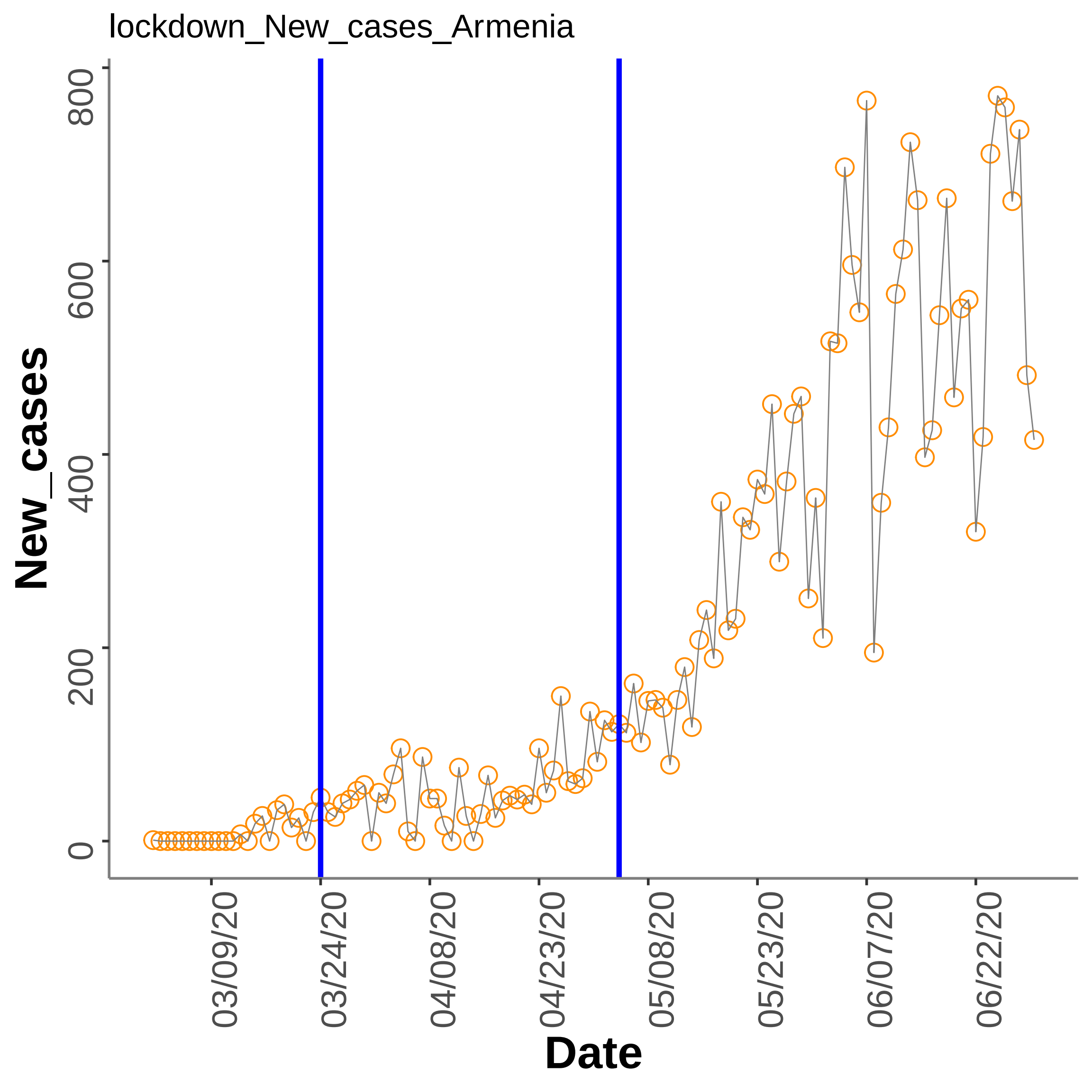
lockdown_New_cases_Armenia
New_cases
800
600
400
200
0
Date
03/09/20
03/24/20
04/08/20
04/23/20
05/08/20
05/23/20
06/07/20
06/22/20

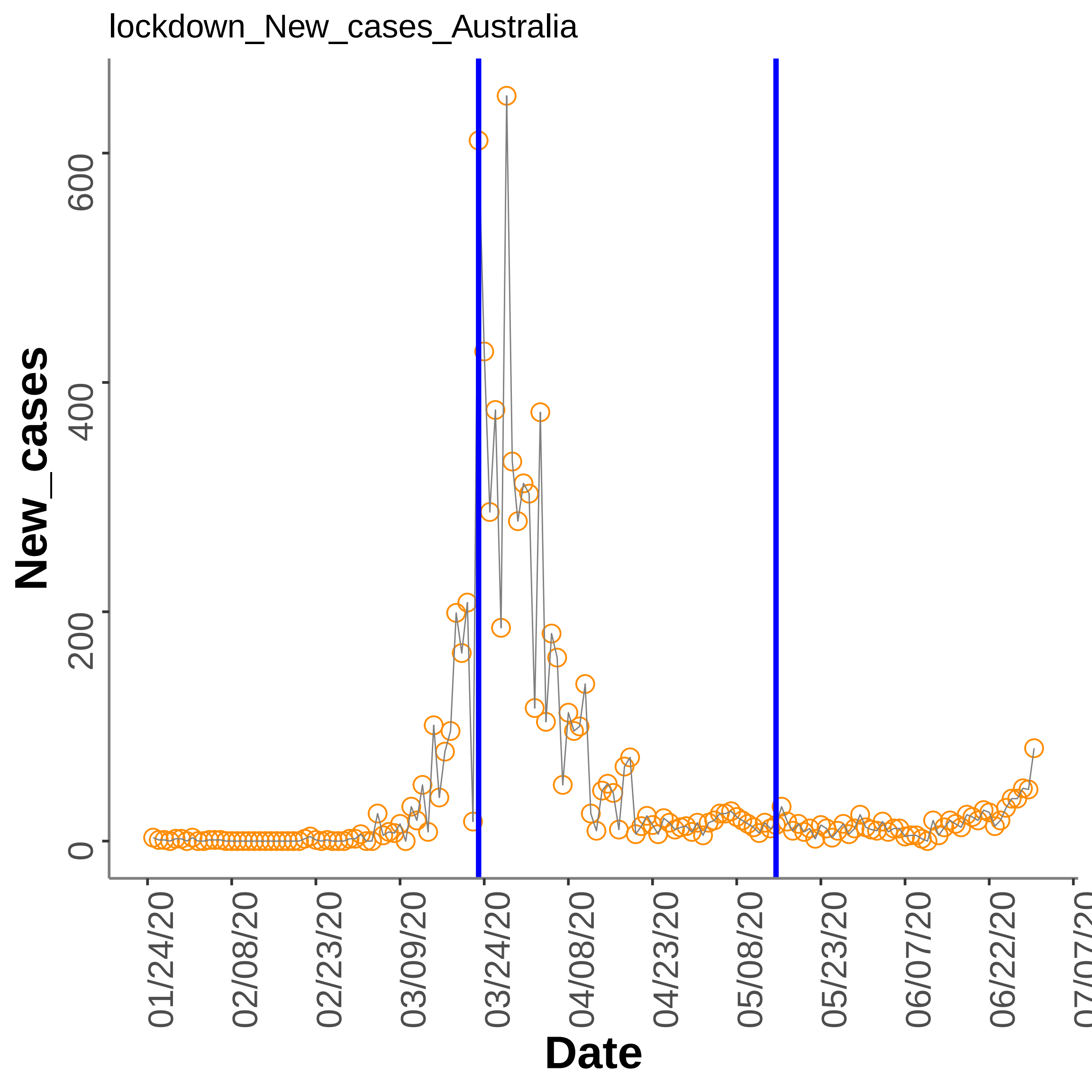

lockdown_New_cases_Australia
New_cases
0
200
400
600
Date
01/24/20
02/08/20
02/23/20
03/09/20
03/24/20
04/08/20
04/23/20
05/08/20
05/23/20
06/07/20
06/22/20
07/07/20

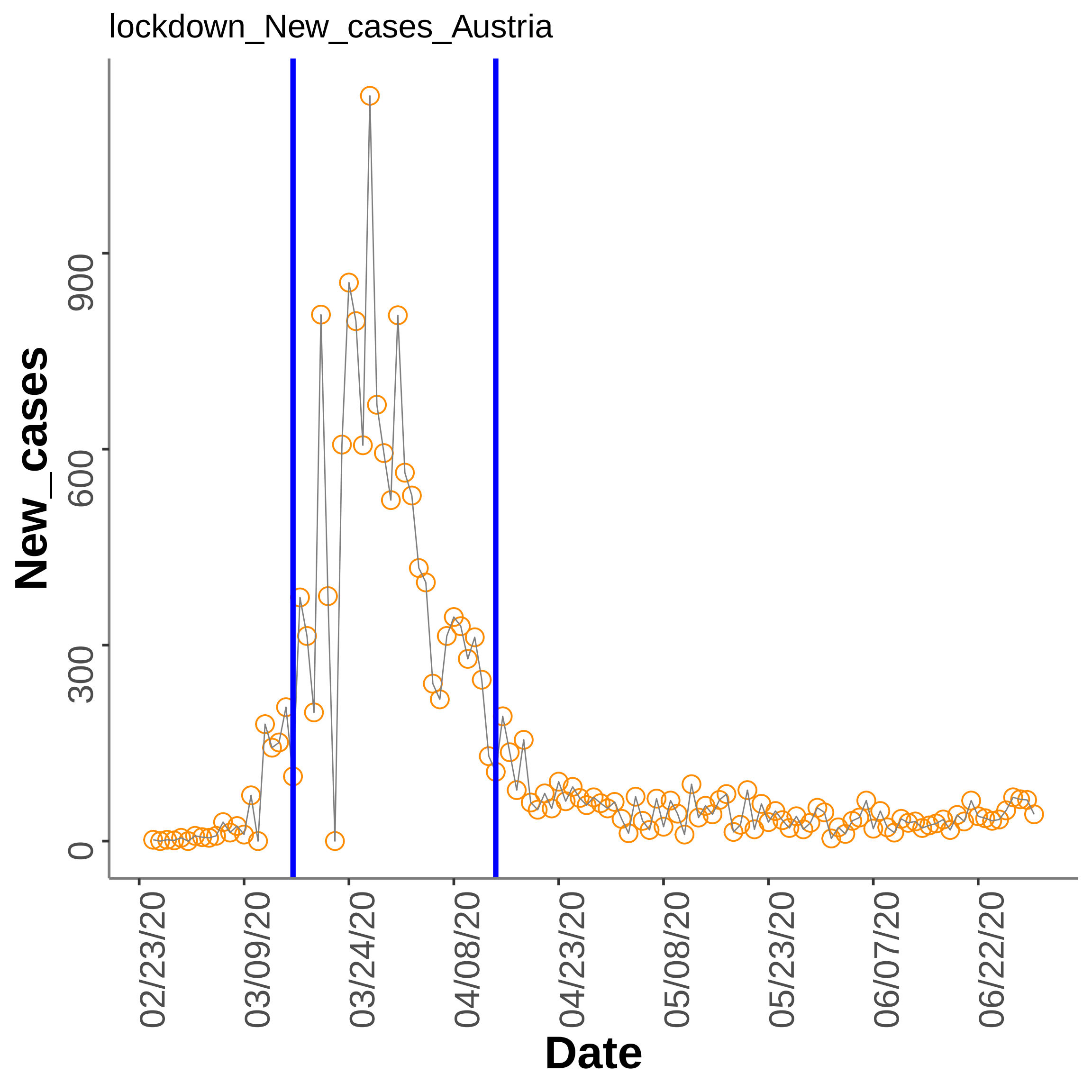
lockdown_New_cases_Austria
New_cases
0
300
600
900
02/23/20
03/09/20
03/24/20
04/08/20
04/23/20
05/08/20
05/23/20
06/07/20
06/22/20
Date

lockdown_New_cases_Azerbaijan

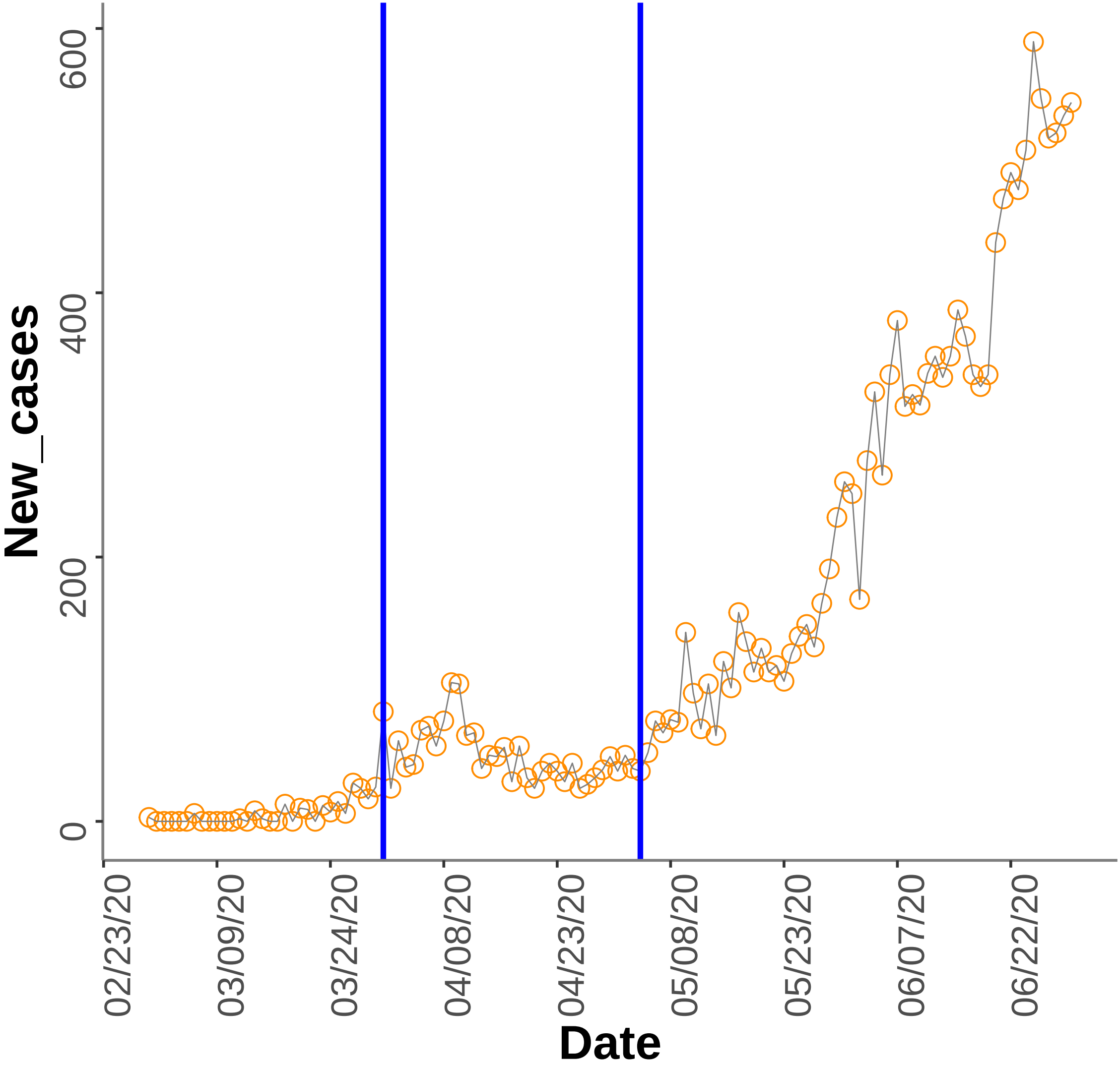

lockdown_New_cases_Bangladesh

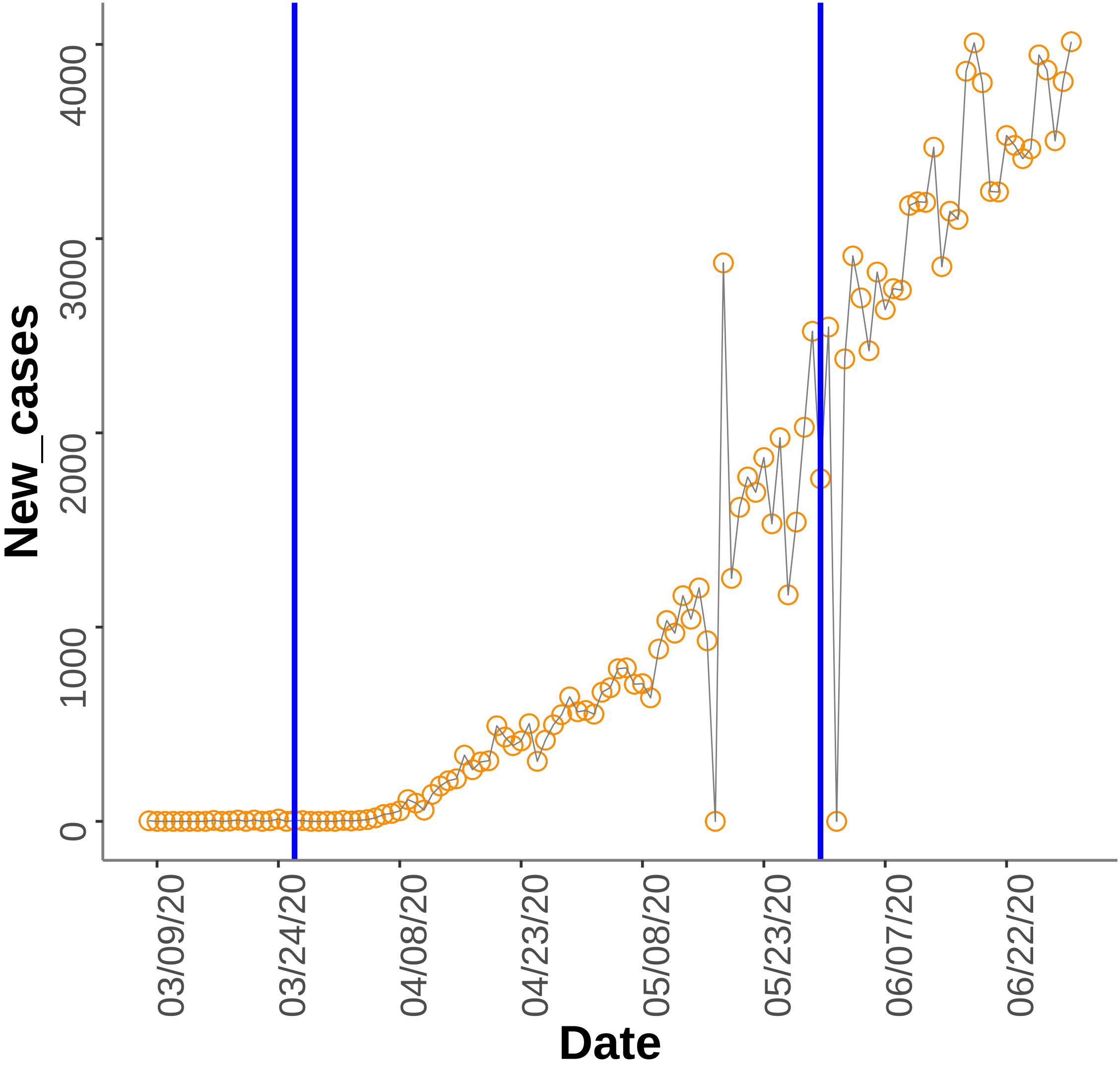

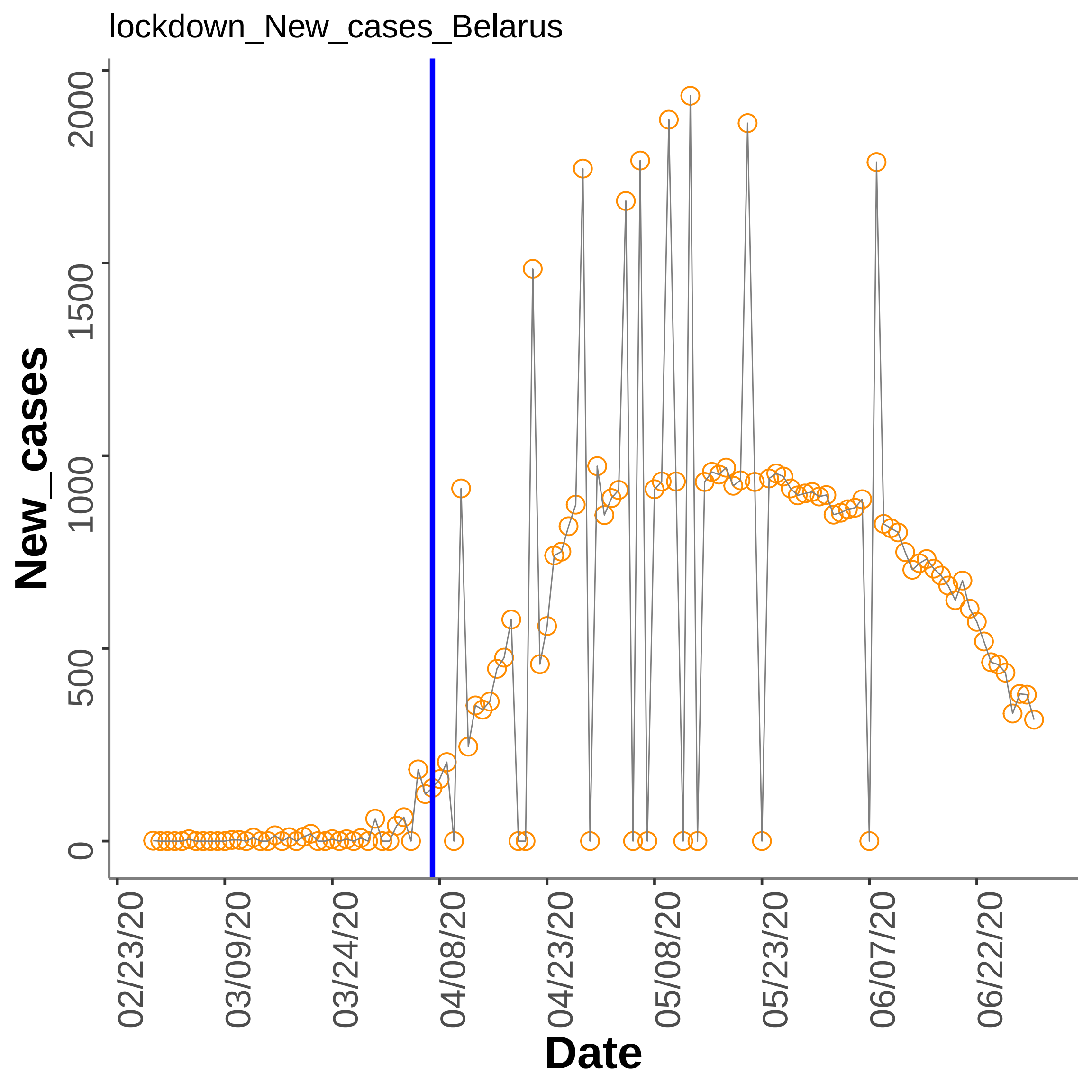

lockdown_New_cases_Belarus
New_cases
0
500
1000
1500
2000
Date
02/23/20
03/09/20
03/24/20
04/08/20
04/23/20
05/08/20
05/23/20
06/07/20
06/22/20

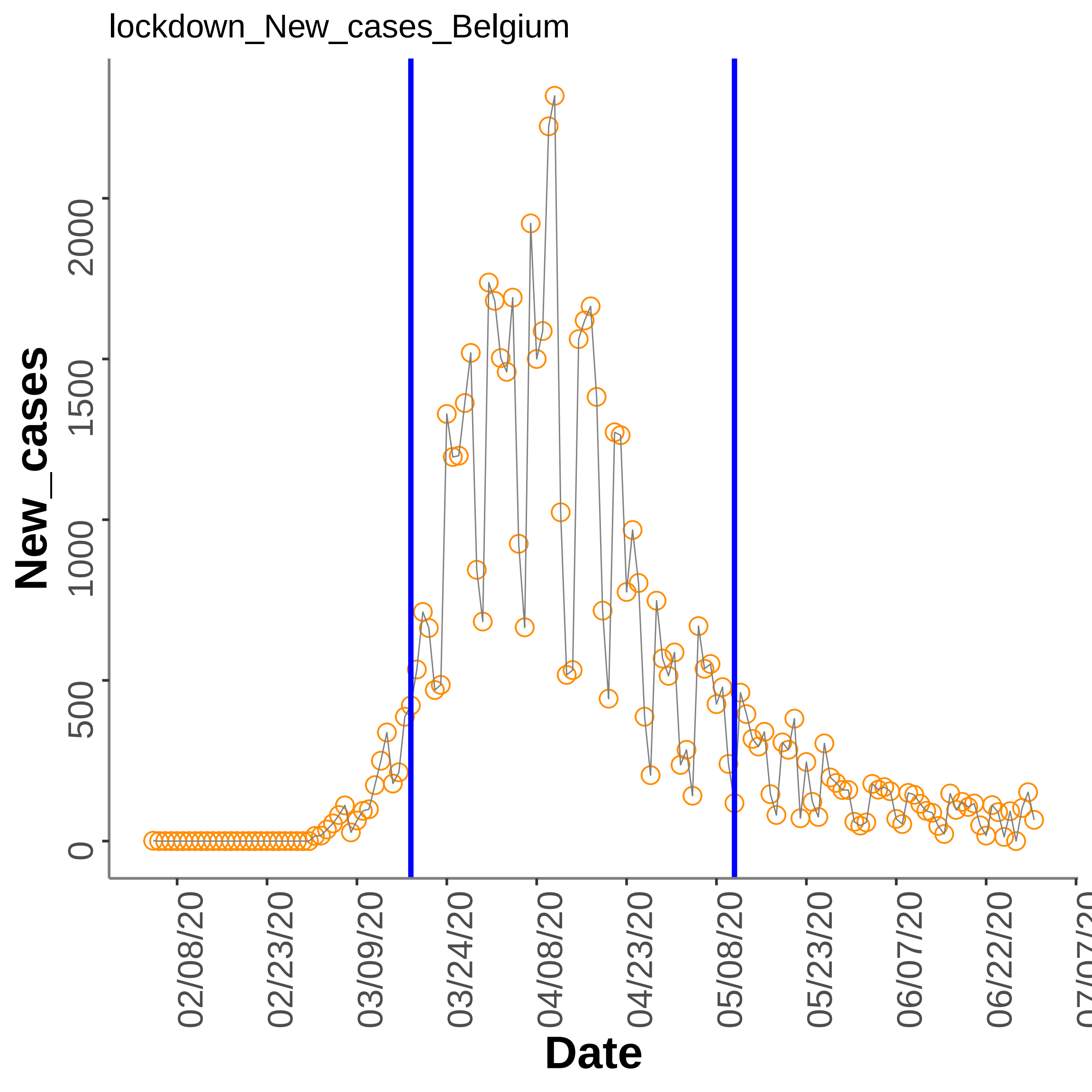

lockdown_New_cases_Belgium
New_cases
0
500
1000
1500
2000
Date
02/08/20
02/23/20
03/09/20
03/24/20
04/08/20
04/23/20
05/08/20
05/23/20
06/07/20
06/22/20

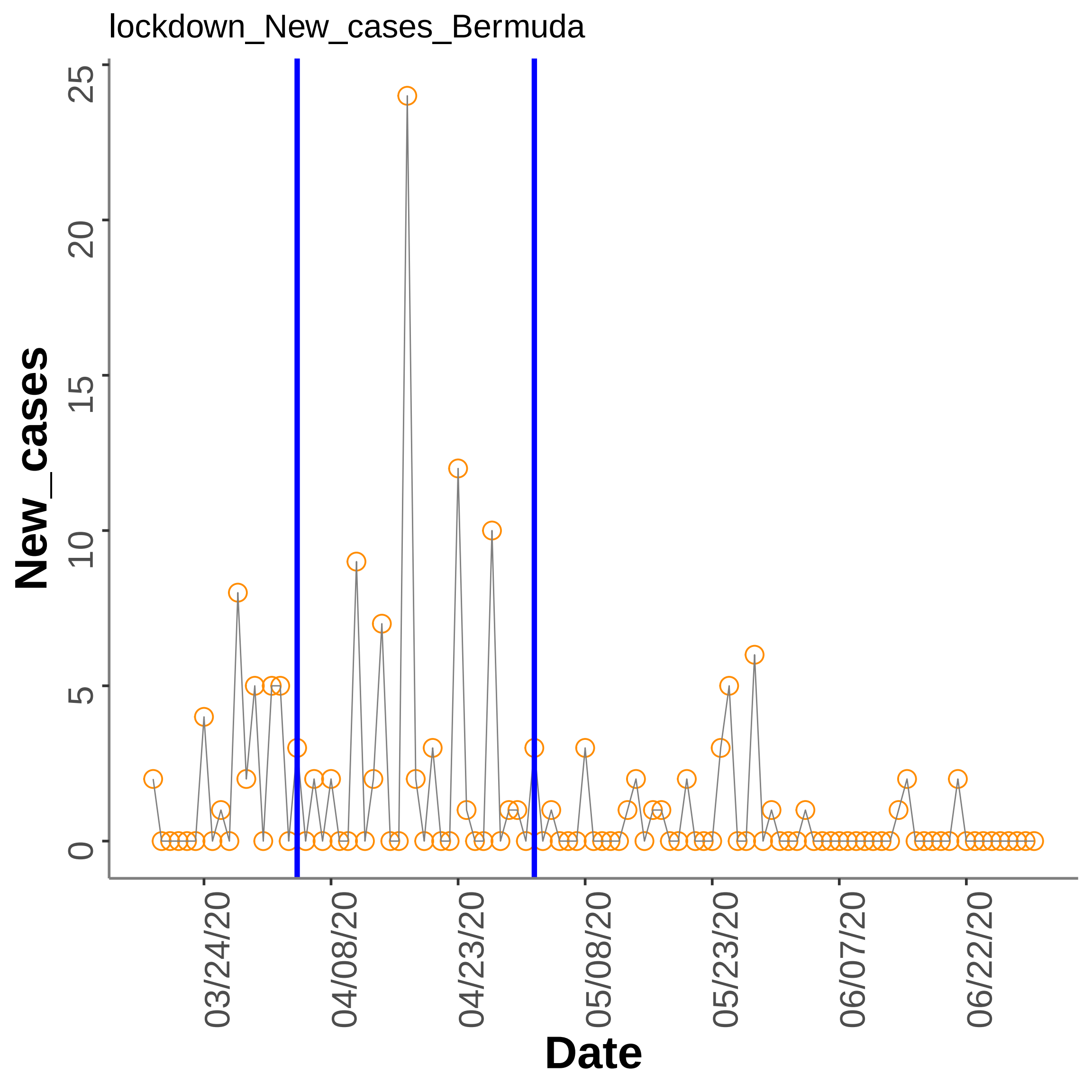

lockdown_New_cases_Bermuda
New_cases
0
5
10
15
20
25
03/24/20
04/08/20
04/23/20
05/08/20
05/23/20
06/07/20
06/22/20
Date

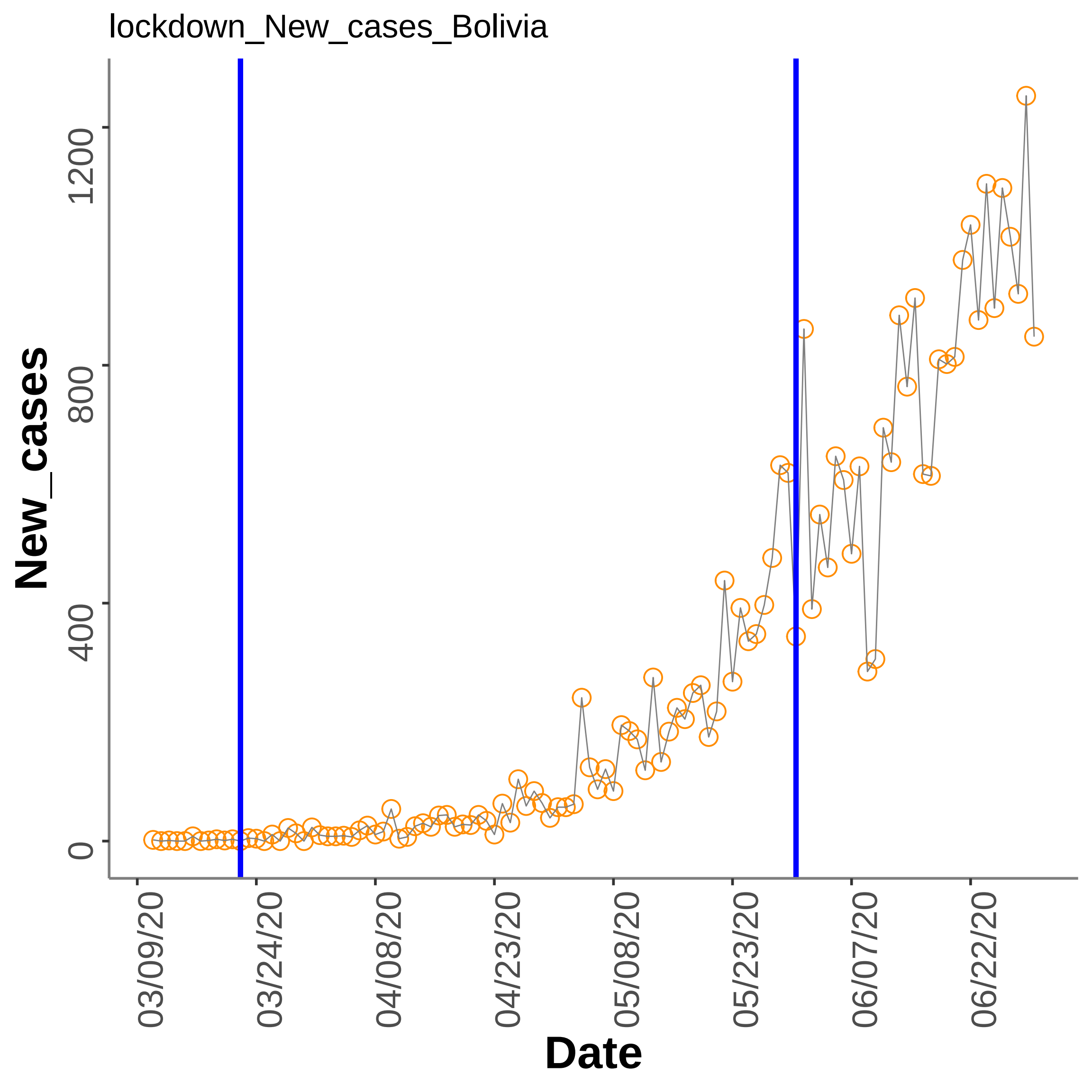

lockdown_New_cases_Bolivia
New_cases
0
400
800
1200
Date
03/09/20
03/24/20
04/08/20
04/23/20
05/08/20
05/23/20
06/07/20
06/22/20

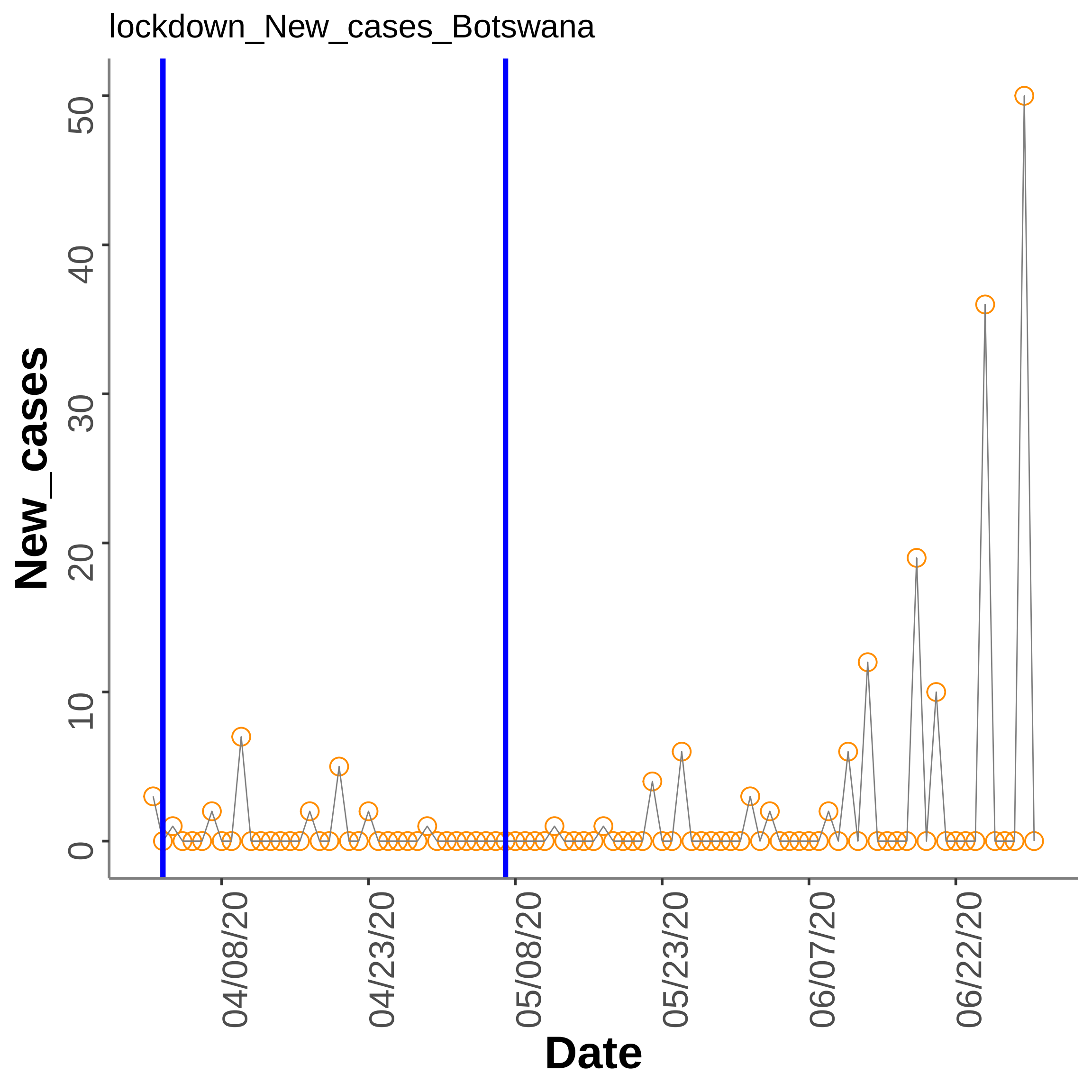

lockdown_New_cases_Botswana
New_cases
0
10
20
30
40
50
04/08/20
04/23/20
05/08/20
05/23/20
06/07/20
06/22/20
Date

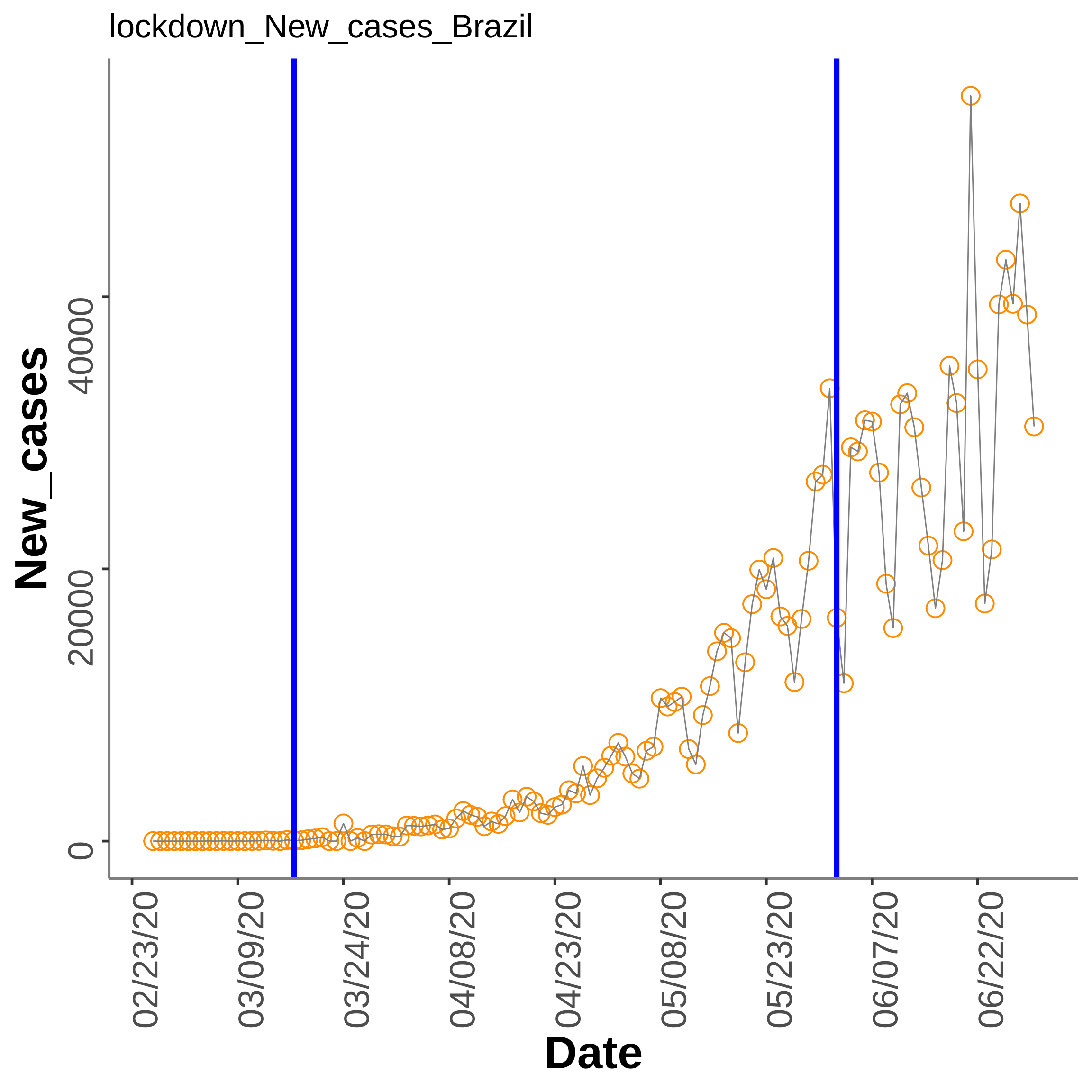
lockdown_New_cases_Brazil
New_cases
0
20000
40000
Date
02/23/20
03/09/20
03/24/20
04/08/20
04/23/20
05/08/20
05/23/20
06/07/20
06/22/20

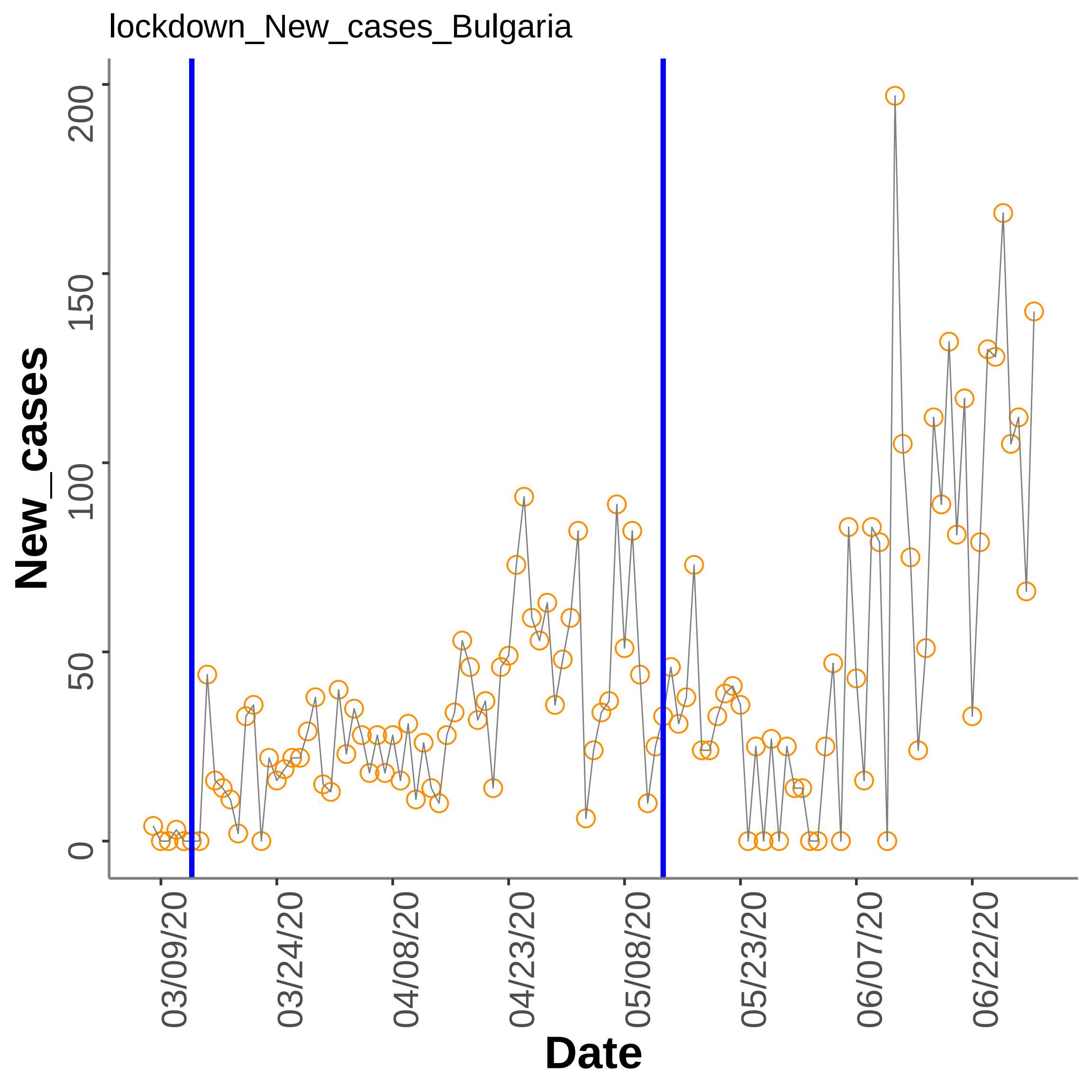

lockdown_New_cases_Bulgaria
New_cases
0
50
100
150
200
03/09/20
03/24/20
04/08/20
04/23/20
05/08/20
05/23/20
06/07/20
06/22/20
Date

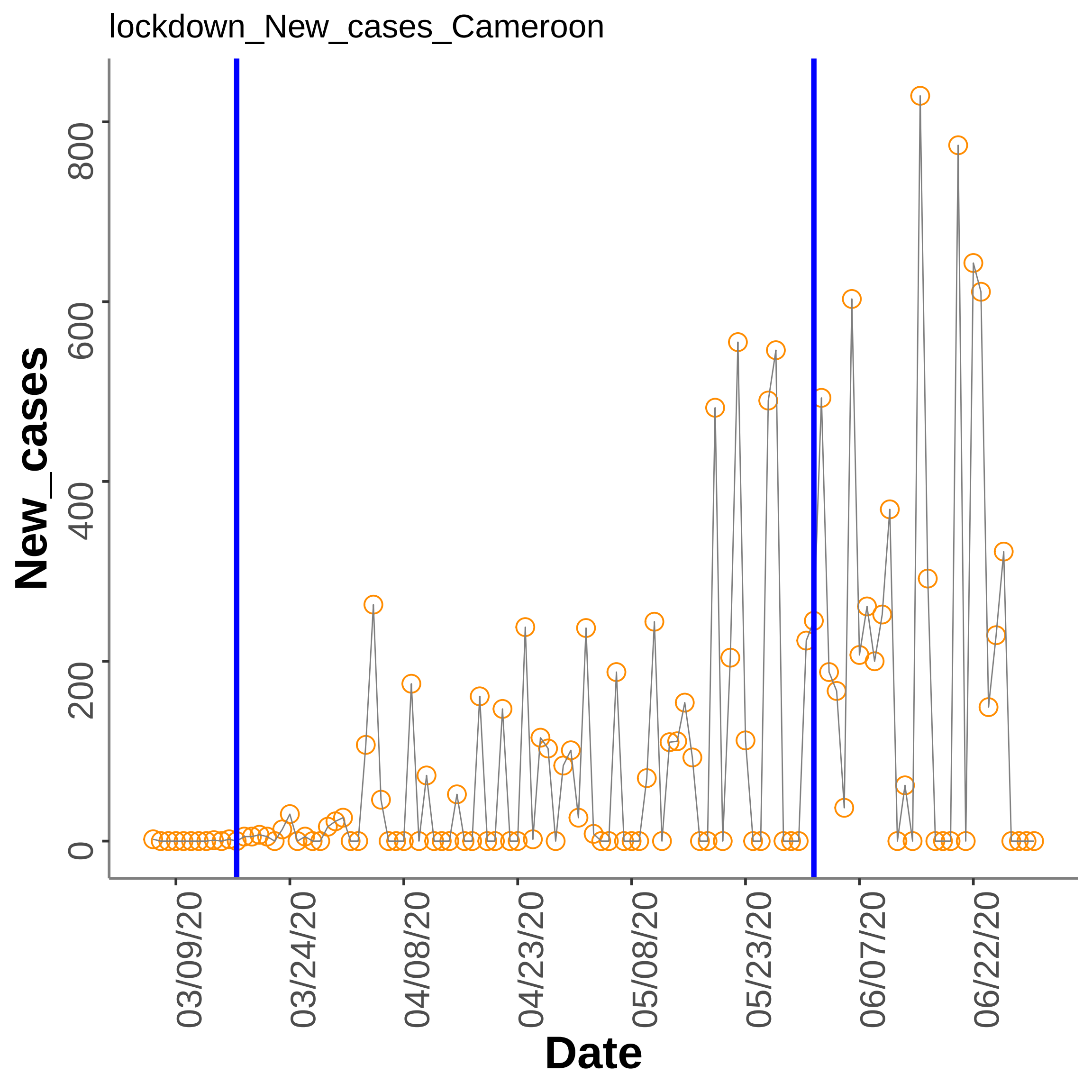
lockdown_New_cases_Cameroon
New_cases
0
200
400
600
800
03/09/20
03/24/20
04/08/20
04/23/20
05/08/20
05/23/20
06/07/20
06/22/20
Date

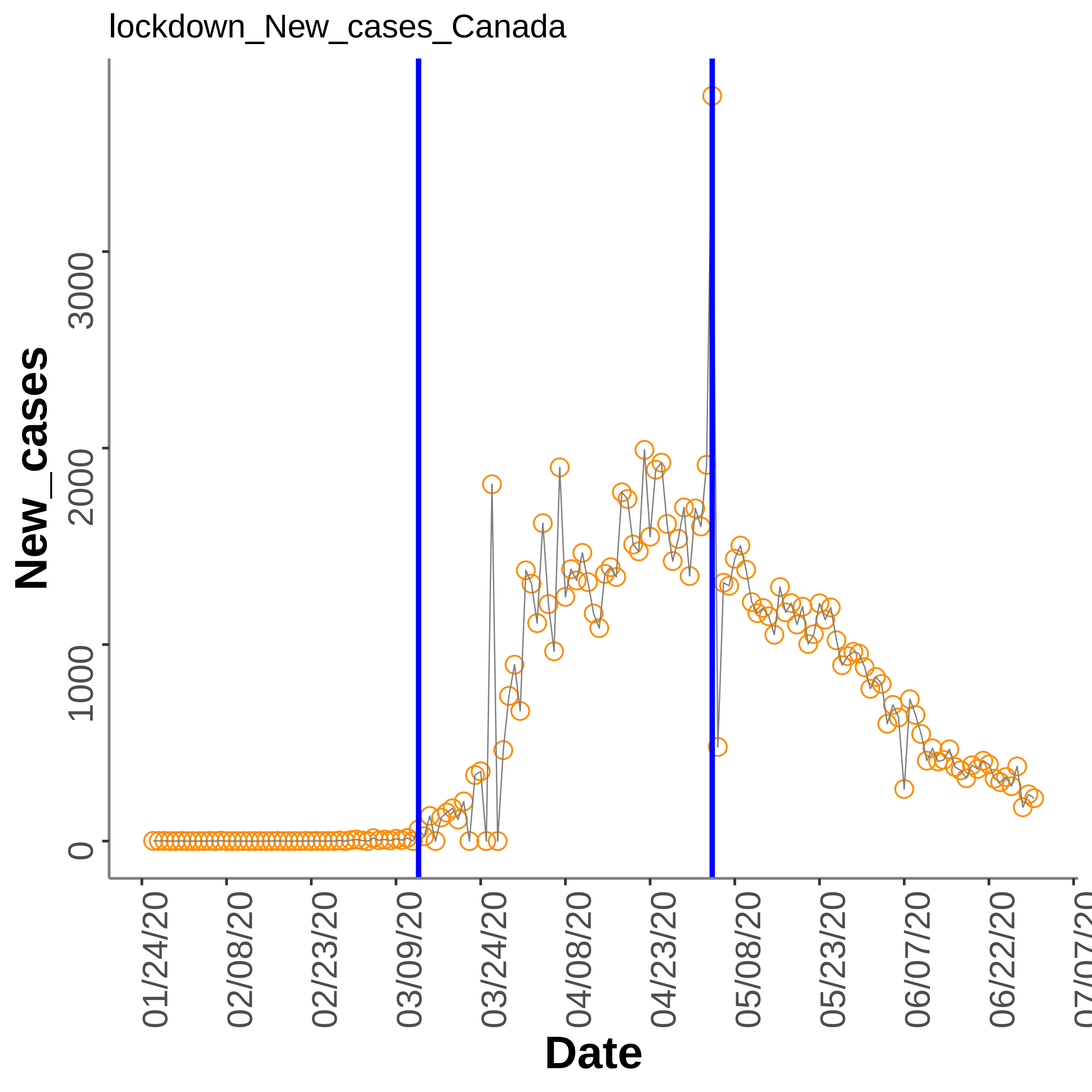

lockdown_New_cases_Canada
New_cases
0
1000
2000
3000
Date
01/24/20
02/08/20
02/23/20
03/09/20
03/24/20
04/08/20
04/23/20
05/08/20
05/23/20
06/07/20
06/22/20
07/07/20

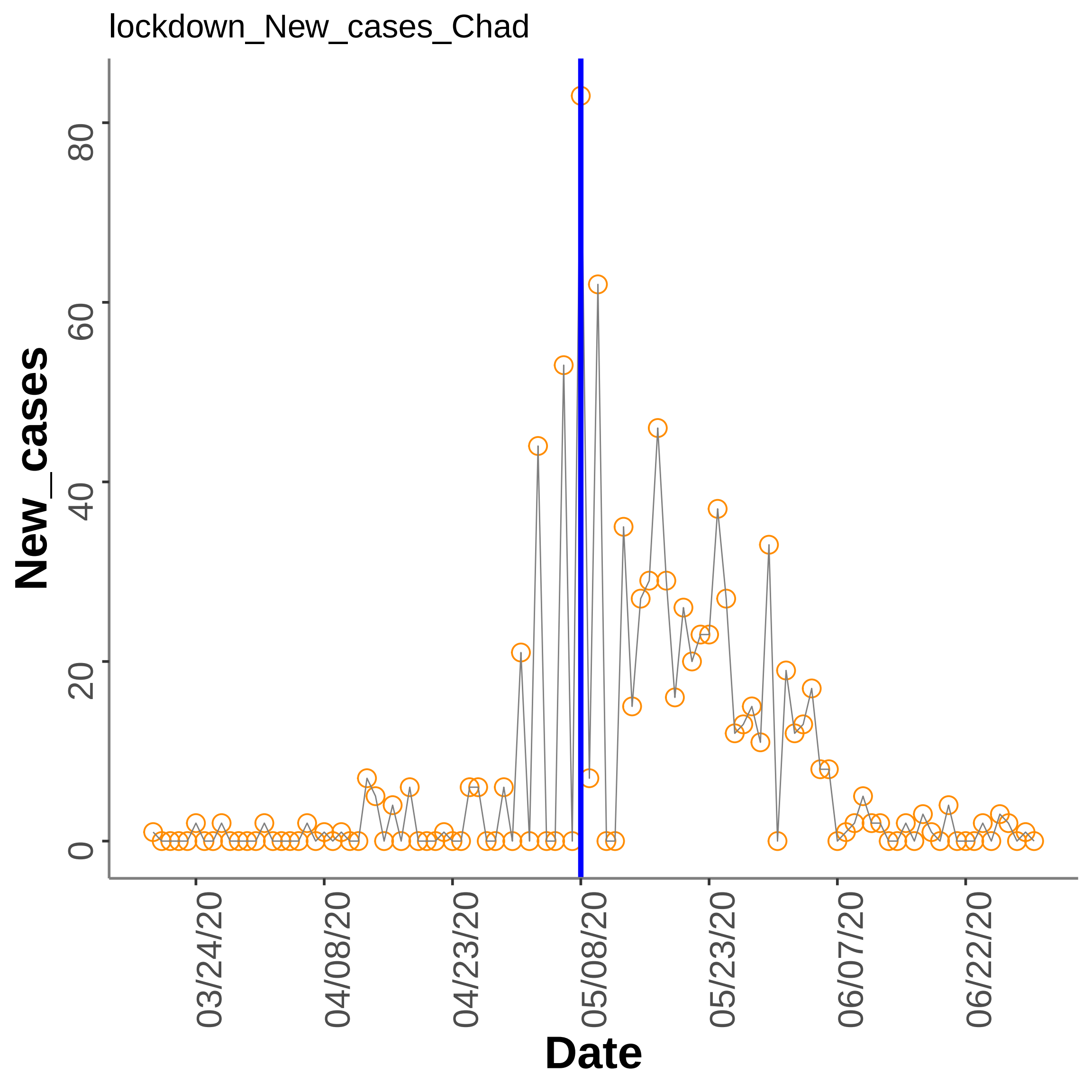
lockdown_New_cases_Chad
New_cases
0
20
40
60
80
03/24/20
04/08/20
04/23/20
05/08/20
05/23/20
06/07/20
06/22/20
Date

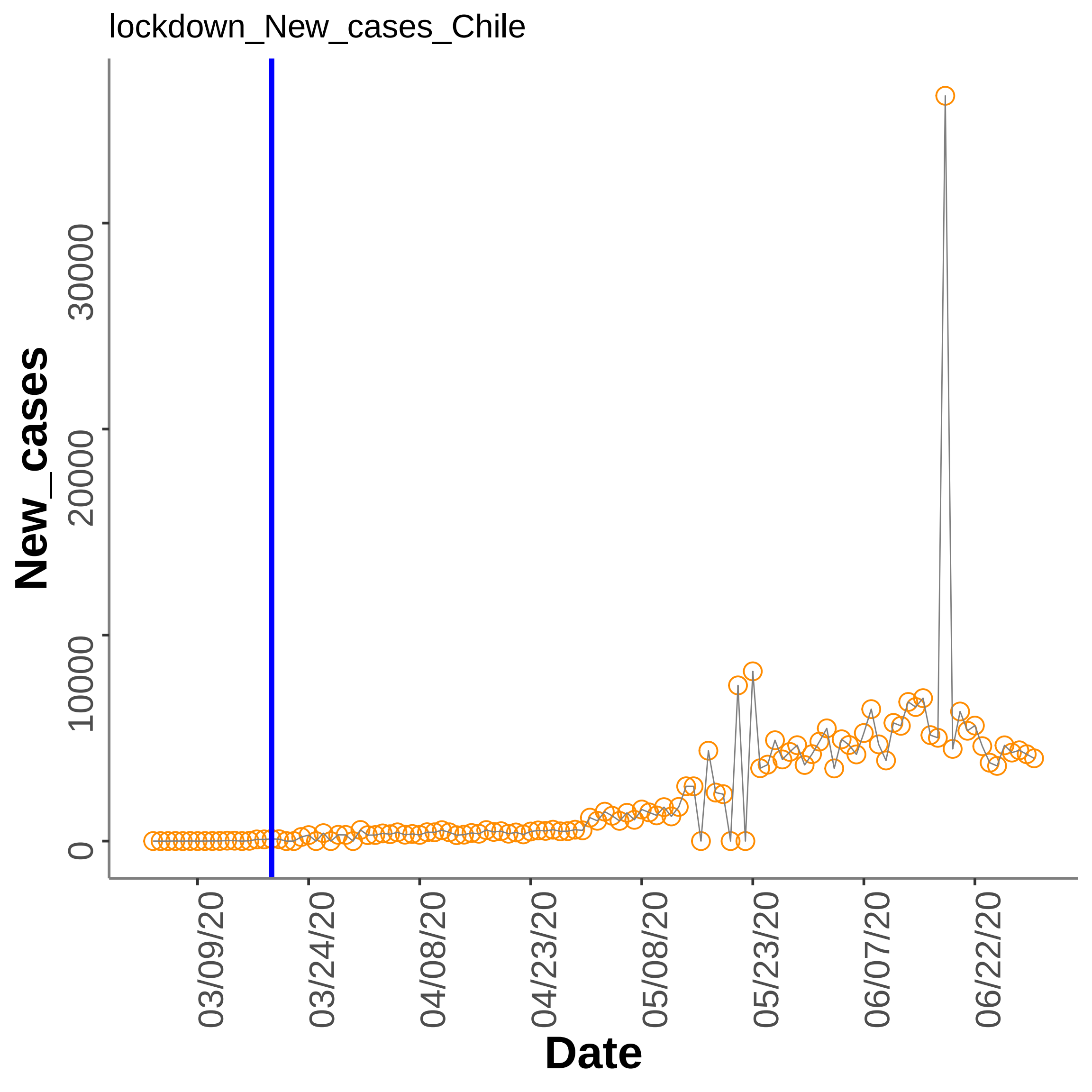

lockdown_New_cases_Chile
New_cases
0
10000
20000
30000
Date
03/09/20
03/24/20
04/08/20
04/23/20
05/08/20
05/23/20
06/07/20
06/22/20

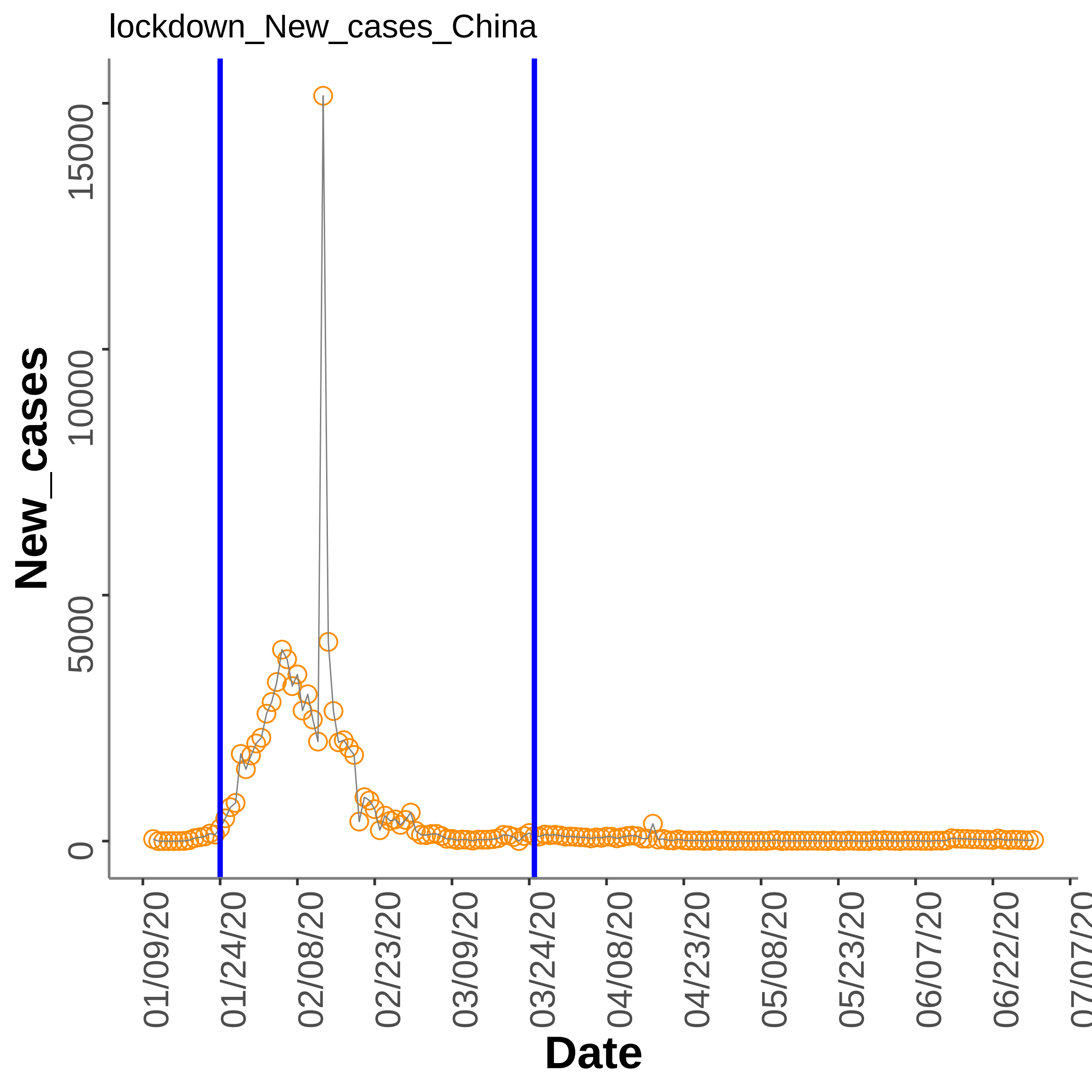
lockdown_New_cases_China
New_cases
0
5000
10000
15000
Date
01/09/20
01/24/20
02/08/20
02/23/20
03/09/20
03/24/20
04/08/20
04/23/20
05/08/20
05/23/20
06/07/20
06/22/20
07/07/20

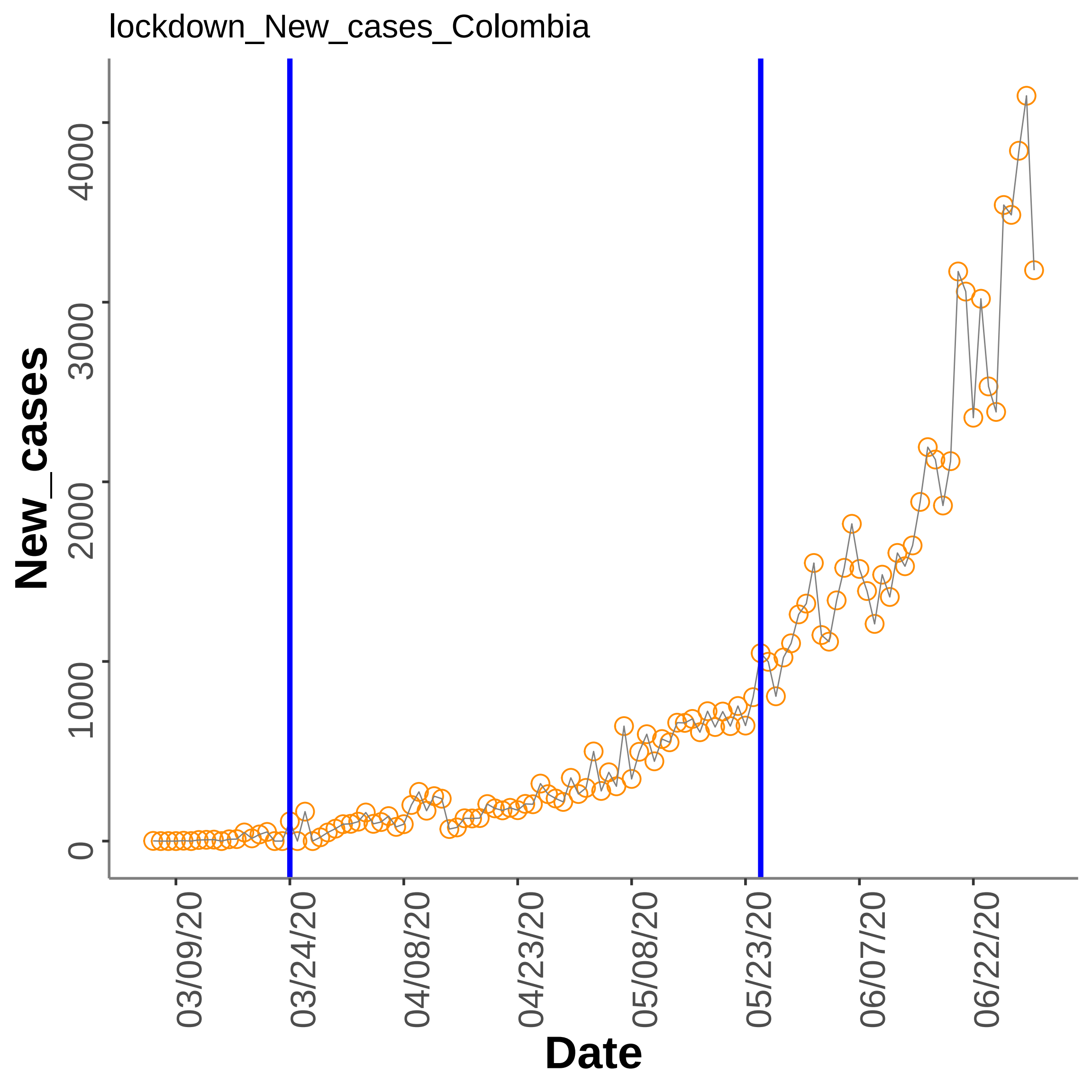

lockdown_New_cases_Colombia
New_cases
0
1000
2000
3000
4000
03/09/20
03/24/20
04/08/20
04/23/20
05/08/20
05/23/20
06/07/20
06/22/20
Date

lockdown_New_cases_Congo

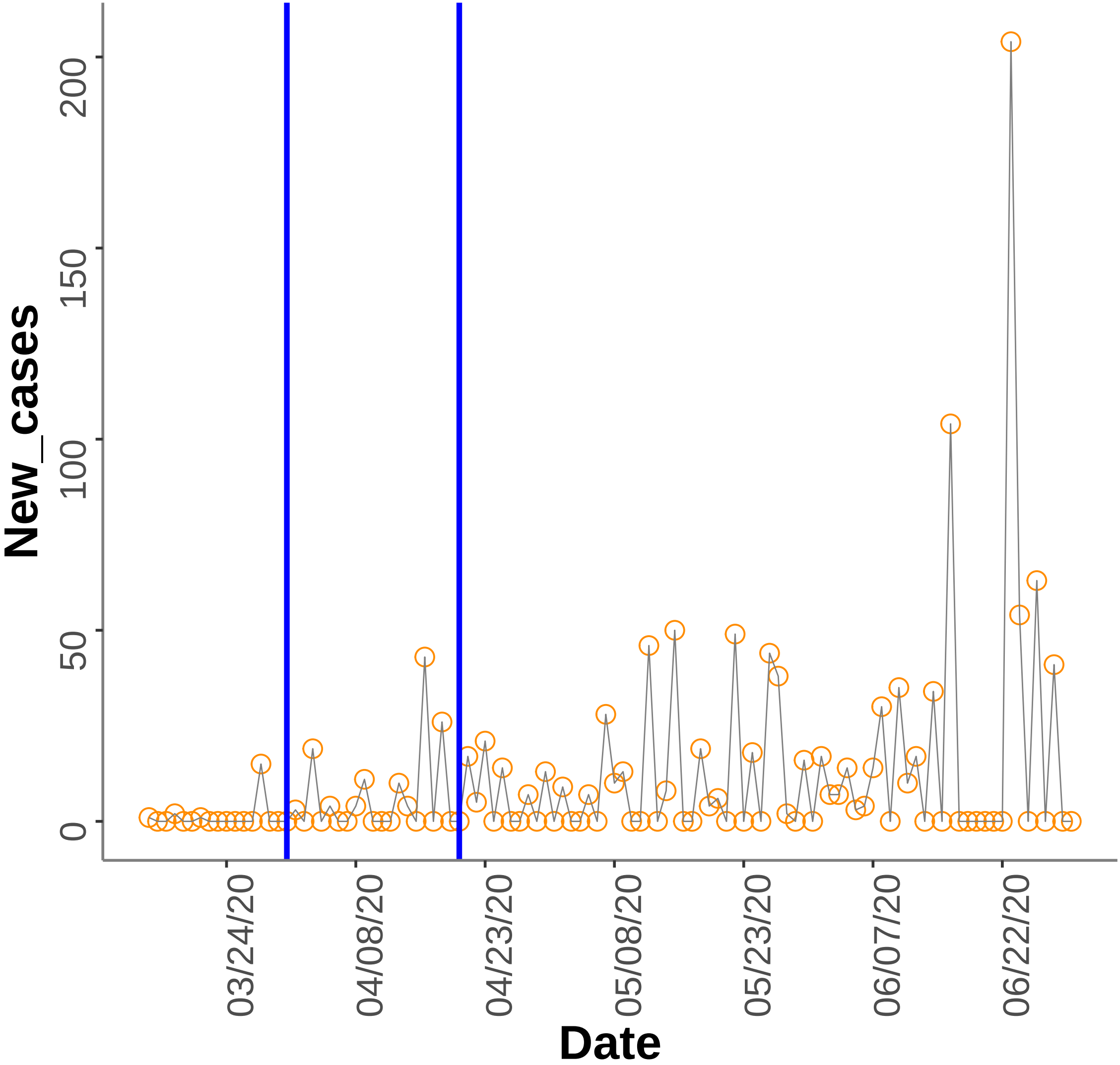

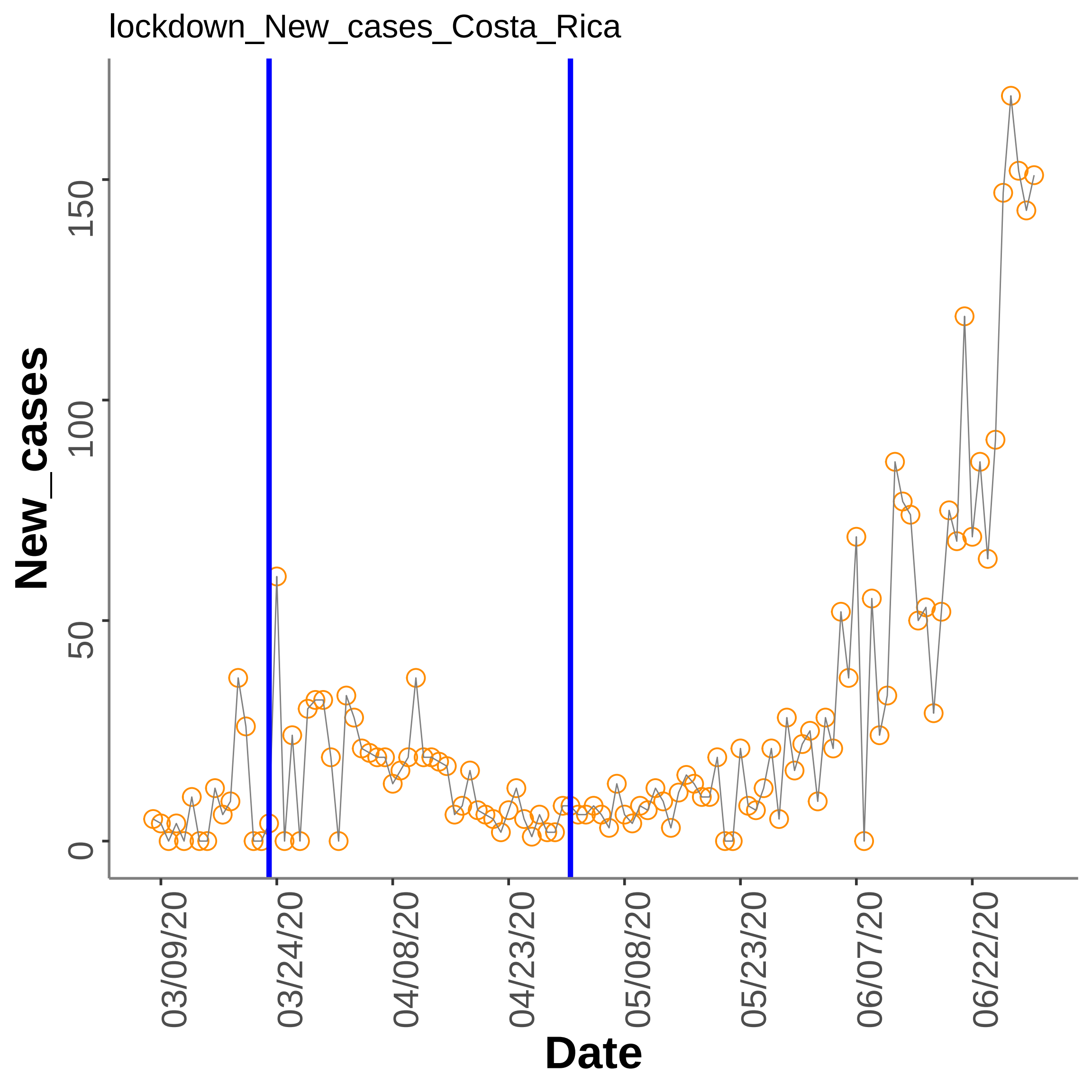

lockdown_New_cases_Costa_Rica
New_cases
0
50
100
150
Date
03/09/20
03/24/20
04/08/20
04/23/20
05/08/20
05/23/20
06/07/20
06/22/20

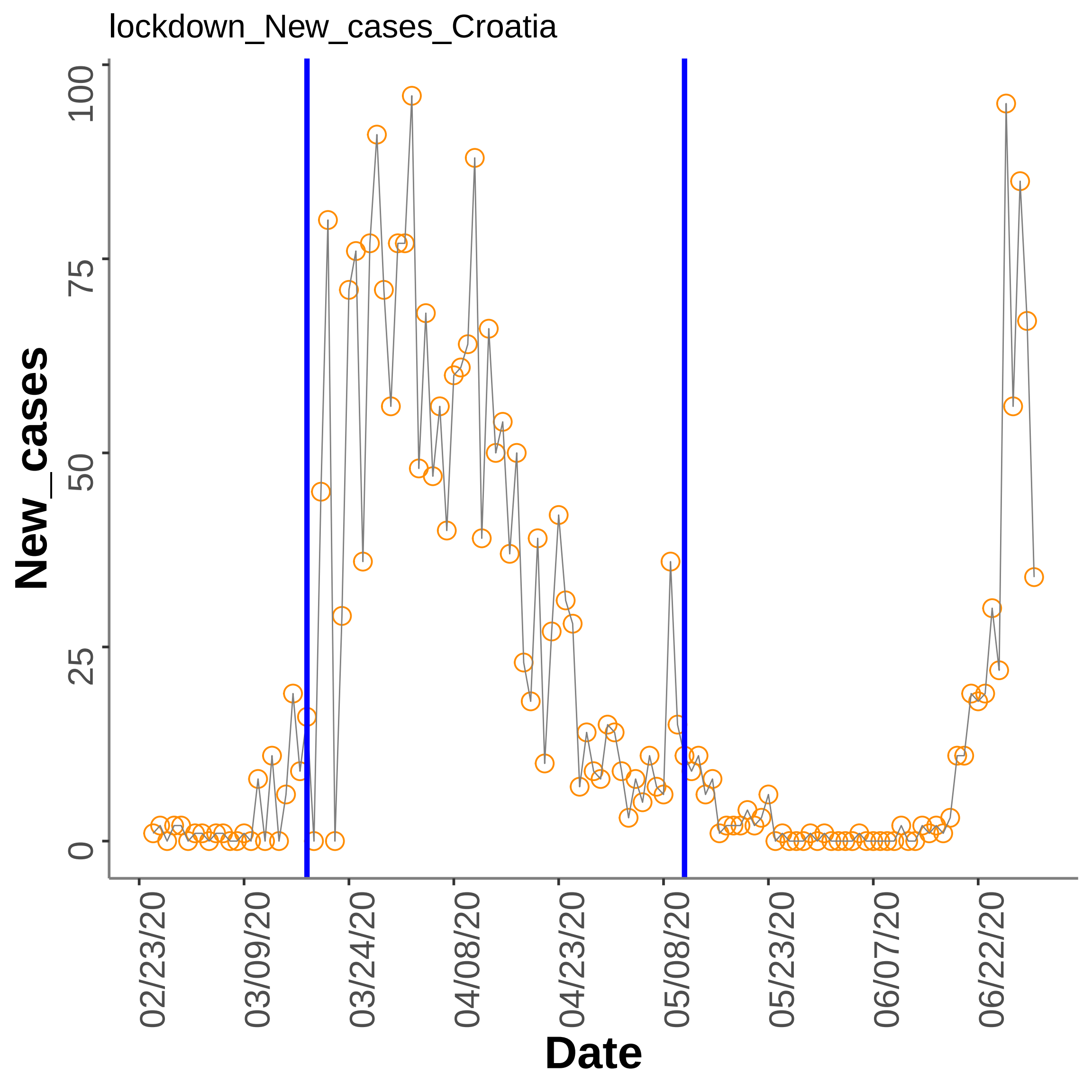

lockdown_New_cases_Croatia
New_cases
100
75
50
25
0
02/23/20
03/09/20
03/24/20
04/08/20
04/23/20
05/08/20
05/23/20
06/07/20
06/22/20
Date

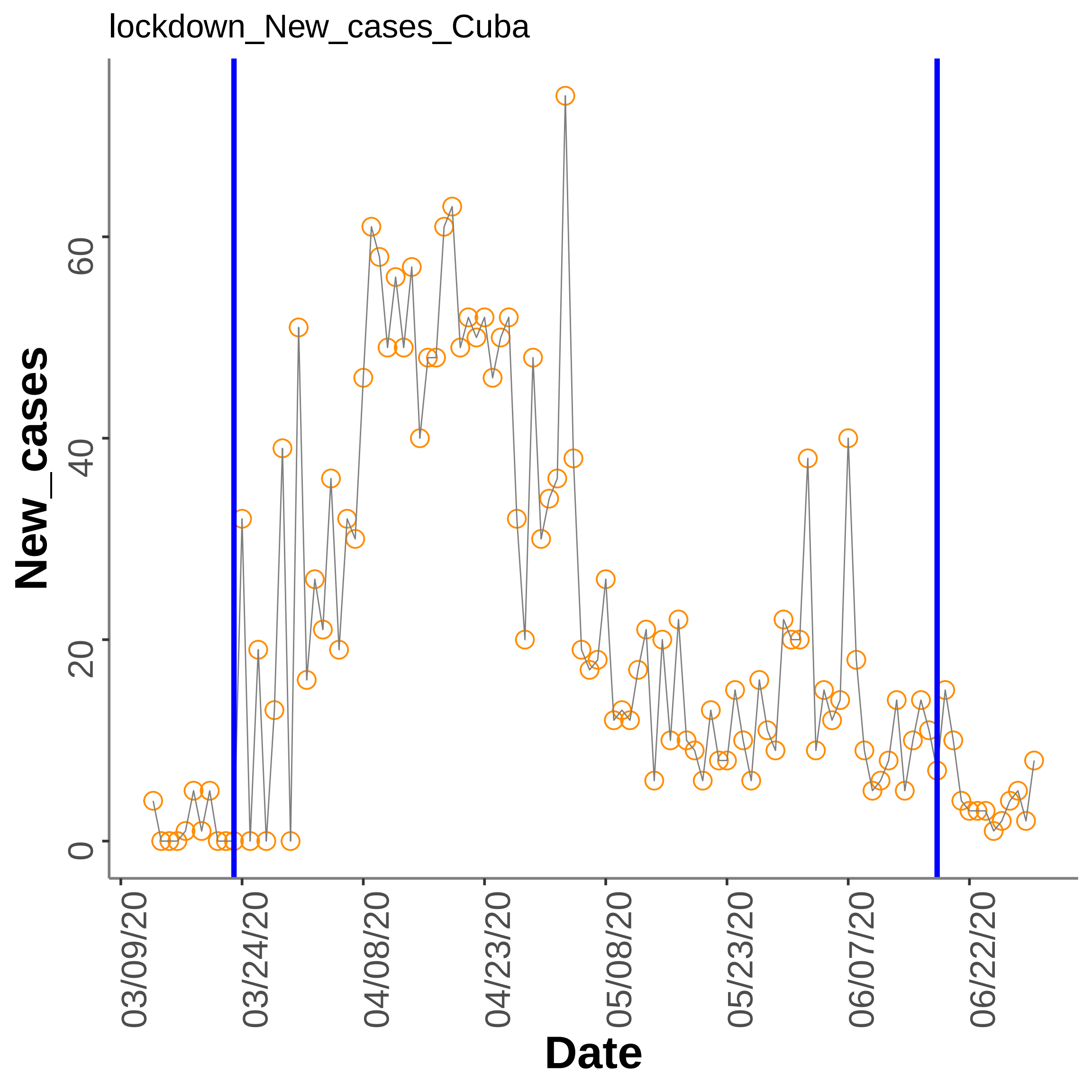
lockdown_New_cases_Cuba
New_cases
0
20
40
60
Date
03/09/20
03/24/20
04/08/20
04/23/20
05/08/20
05/23/20
06/07/20
06/22/20

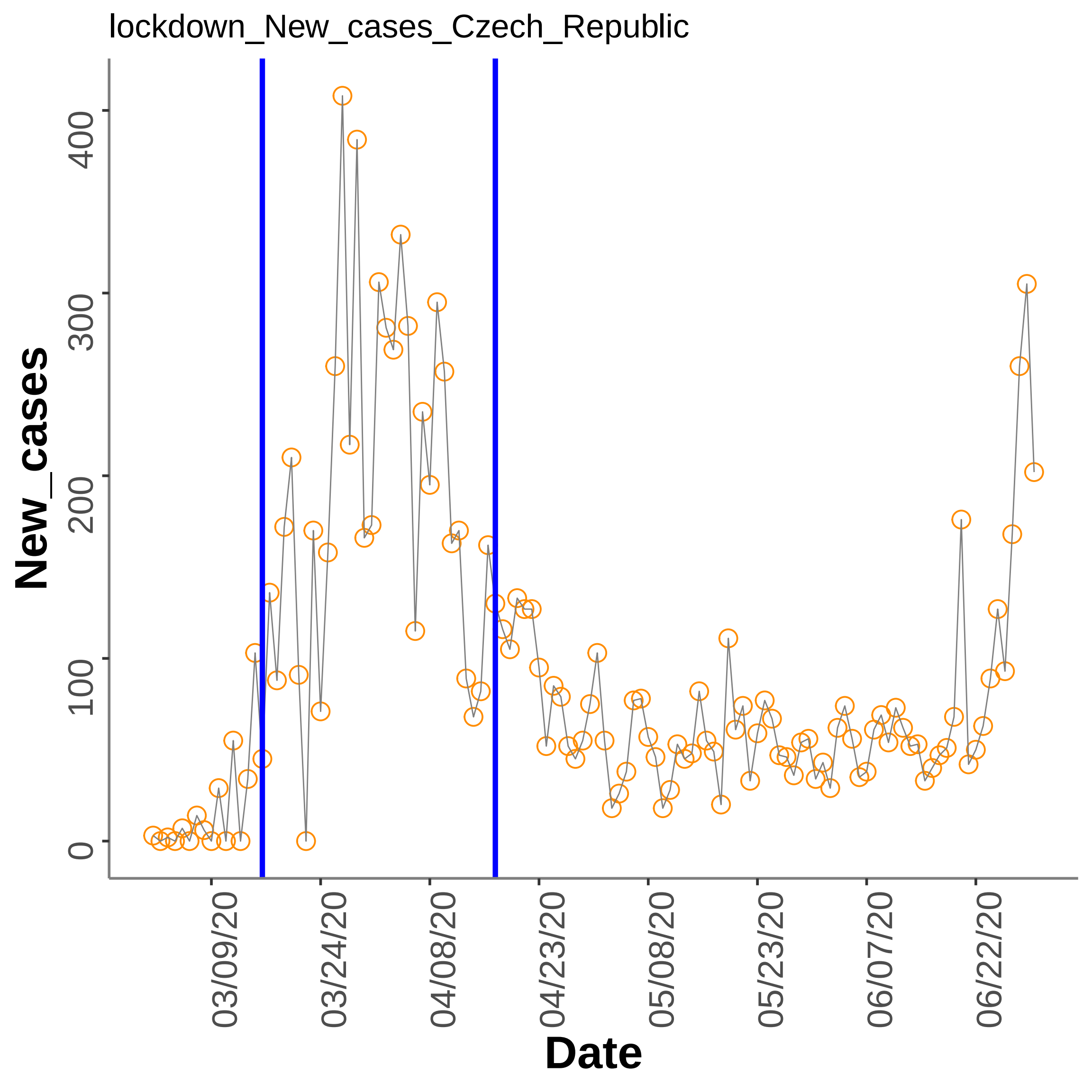
lockdown_New_cases_Czech_Republic
New_cases
0
100
200
300
400
Date
03/09/20
03/24/20
04/08/20
04/23/20
05/08/20
05/23/20
06/07/20
06/22/20

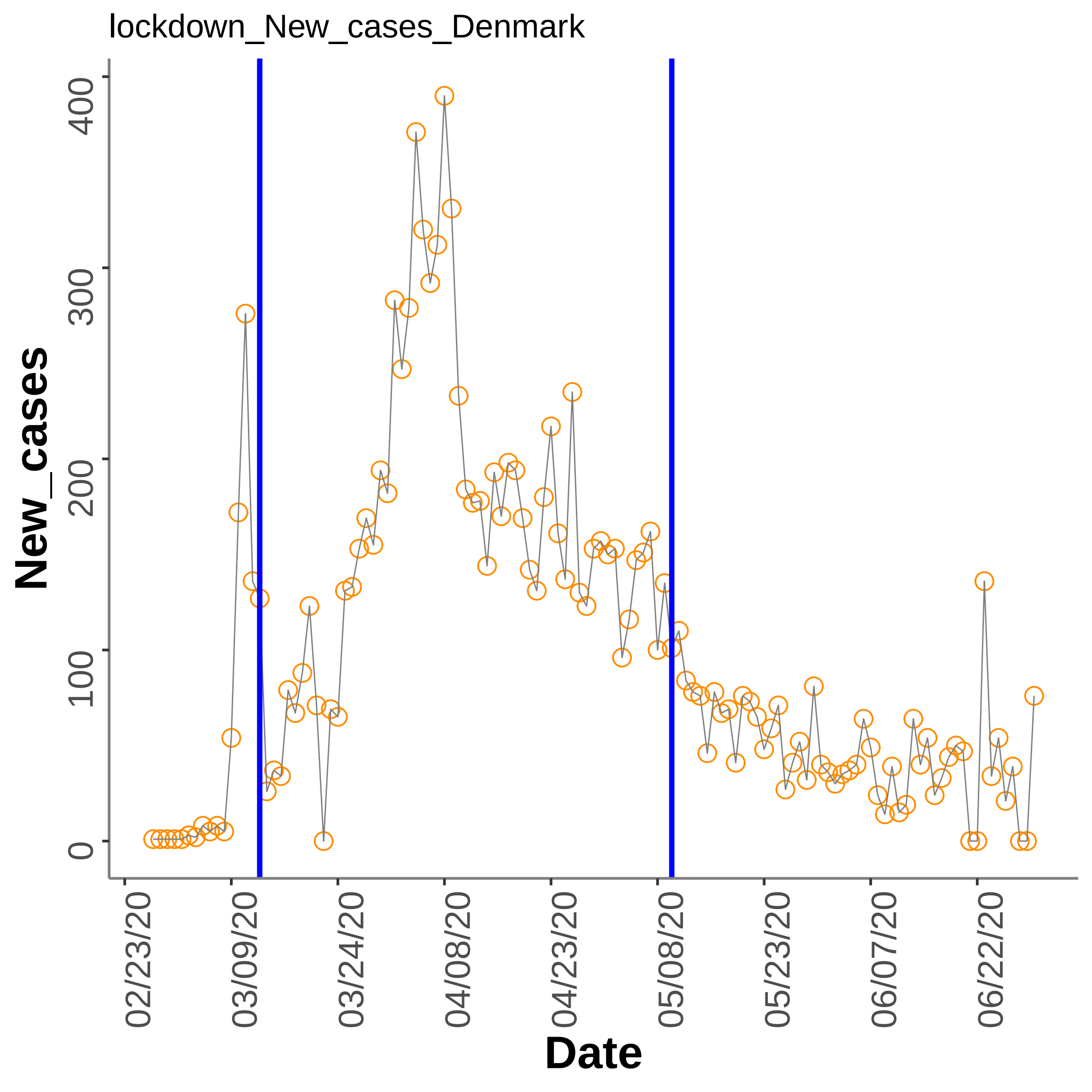
lockdown_New_cases_Denmark
New_cases
0
100
200
300
400
Date
02/23/20
03/09/20
03/24/20
04/08/20
04/23/20
05/08/20
05/23/20
06/07/20
06/22/20

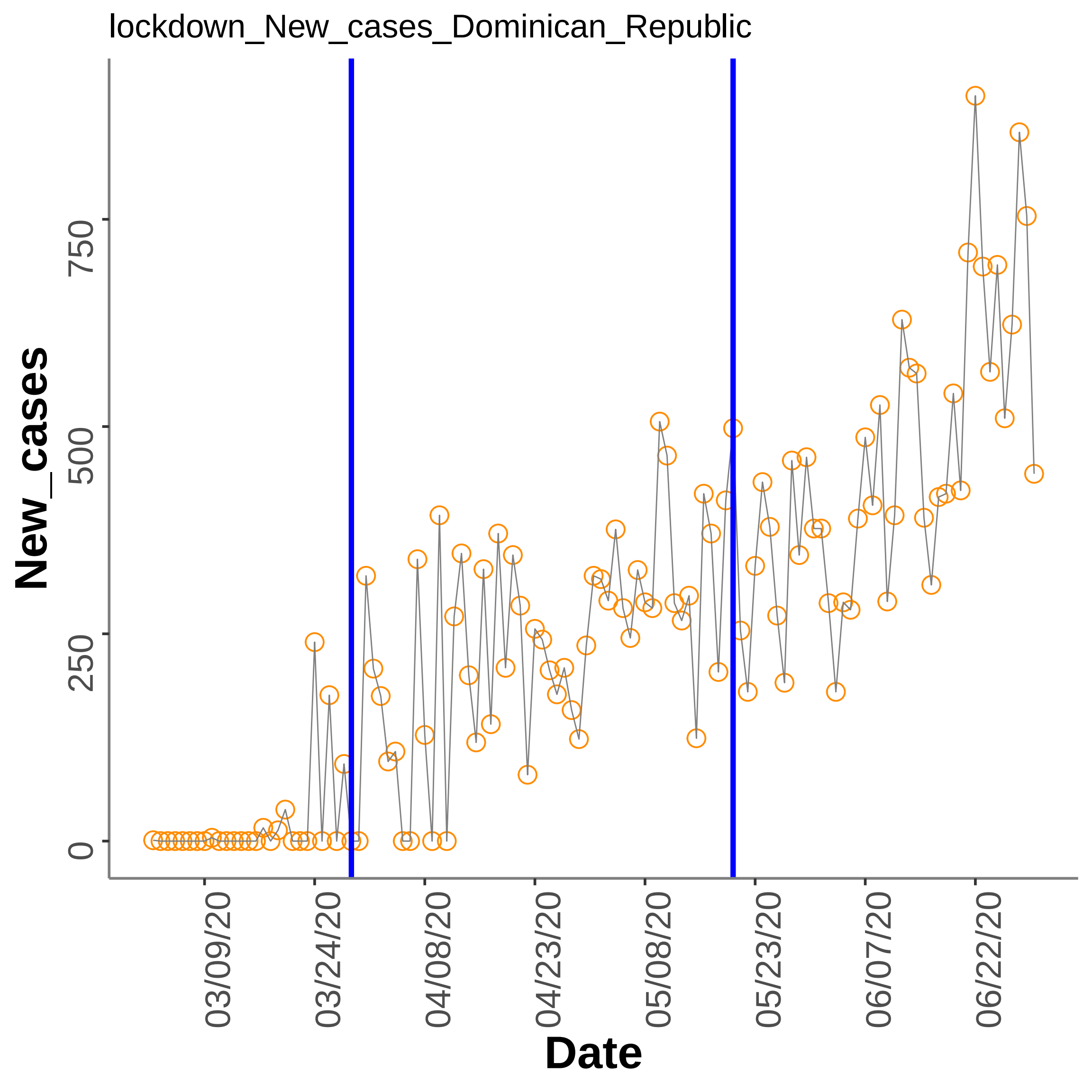

lockdown_New_cases_Dominican_Republic
New_cases
0
250
500
750
Date
03/09/20
03/24/20
04/08/20
04/23/20
05/08/20
05/23/20
06/07/20
06/22/20

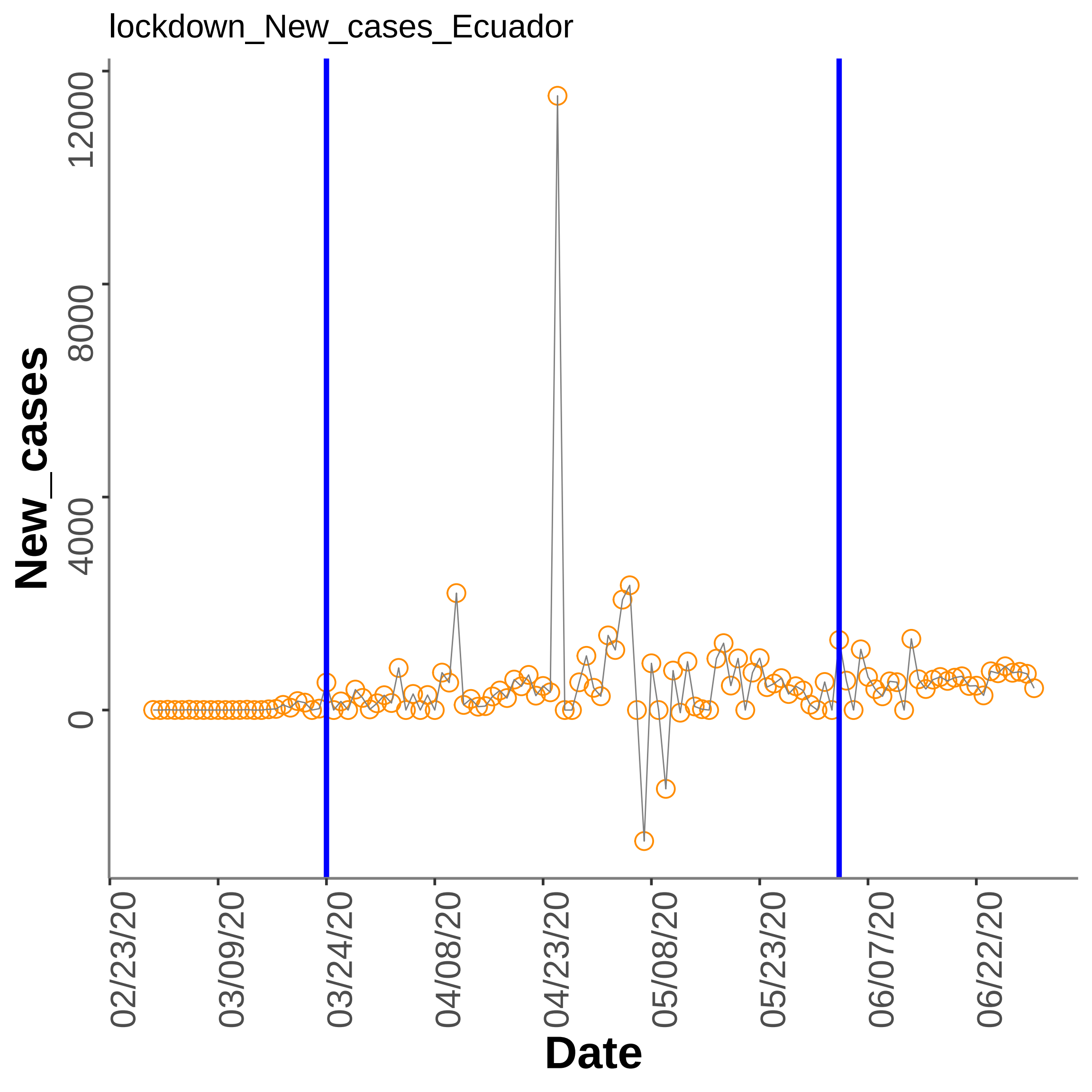

lockdown_New_cases_Ecuador
New_cases
0
4000
8000
12000
Date
02/23/20
03/09/20
03/24/20
04/08/20
04/23/20
05/08/20
05/23/20
06/07/20
06/22/20

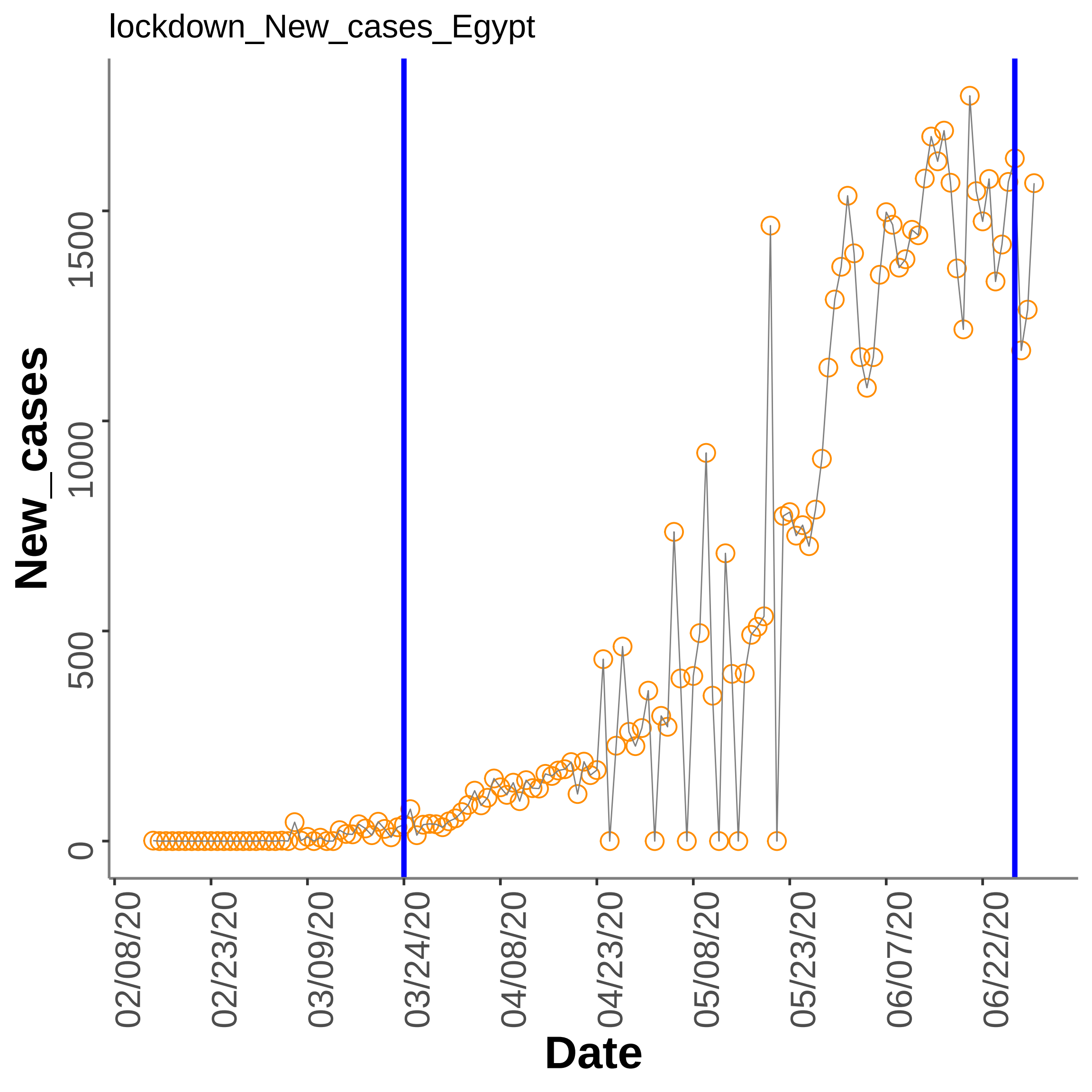
lockdown_New_cases_Egypt
New_cases
0
500
1000
1500
02/08/20
02/23/20
03/09/20
03/24/20
04/08/20
04/23/20
05/08/20
05/23/20
06/07/20
06/22/20
Date

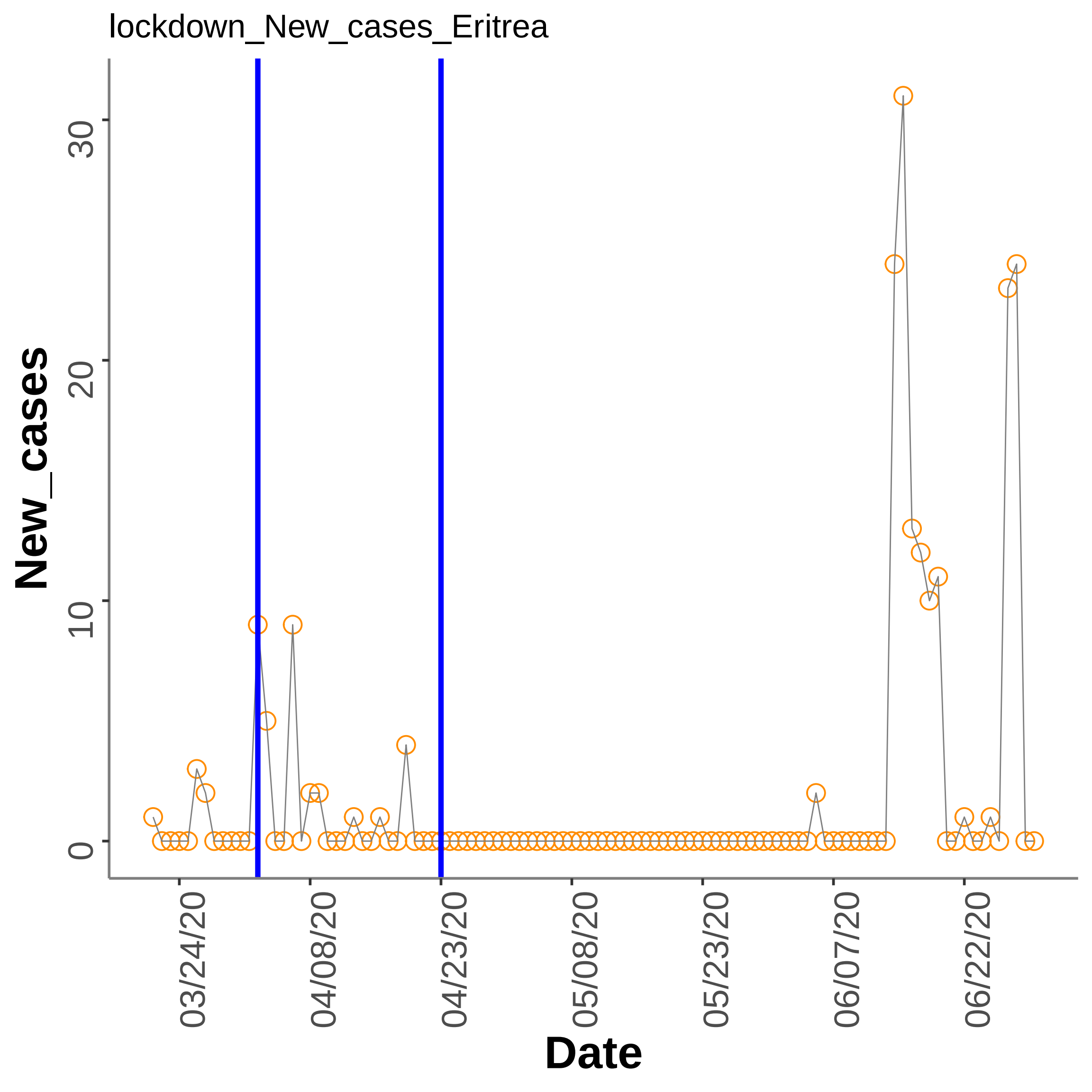
lockdown_New_cases_Eritrea
New_cases
0
10
20
30
Date
03/24/20
04/08/20
04/23/20
05/08/20
05/23/20
06/07/20
06/22/20

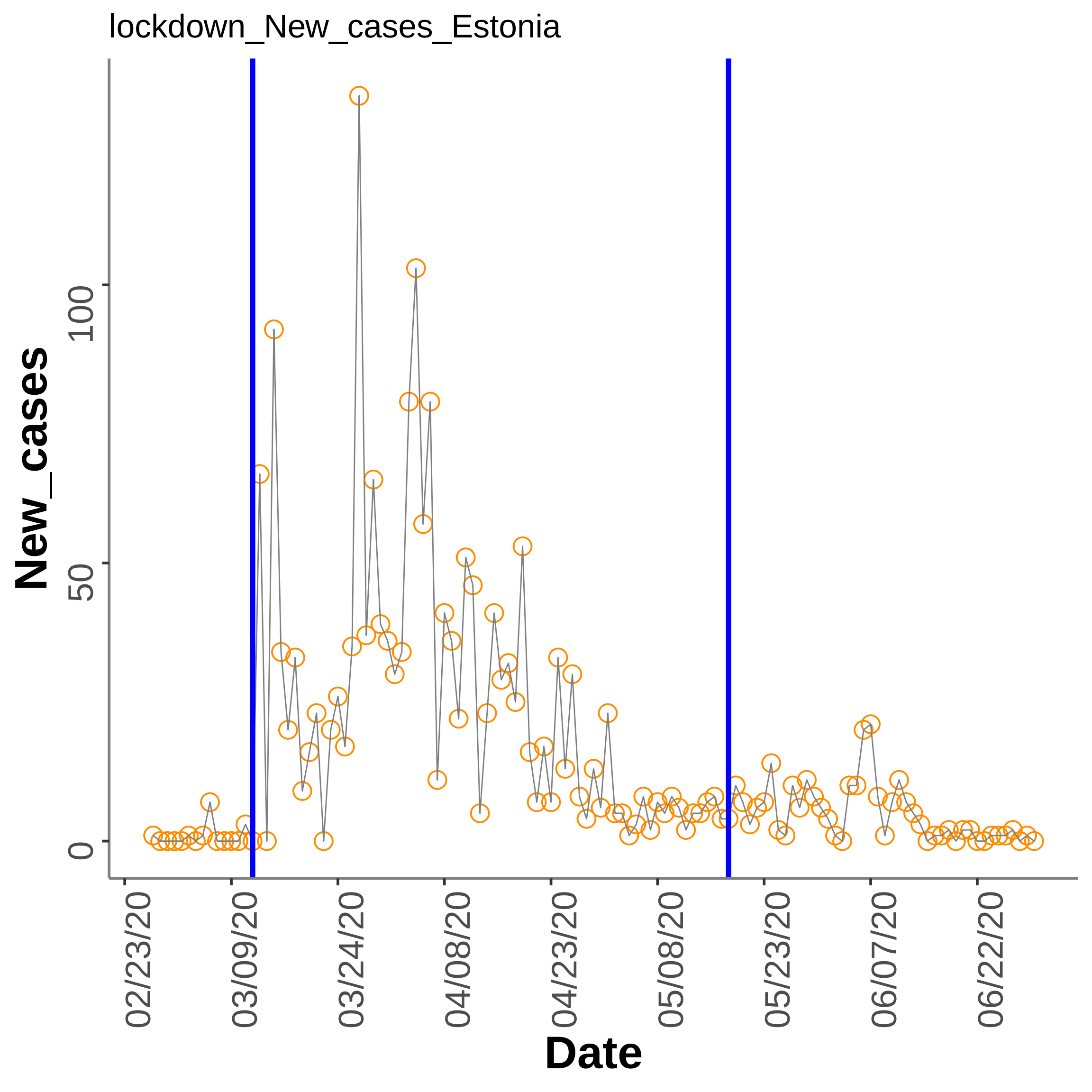

lockdown_New_cases_Estonia
New_cases
0
50
100
Date
02/23/20
03/09/20
03/24/20
04/08/20
04/23/20
05/08/20
05/23/20
06/07/20
06/22/20

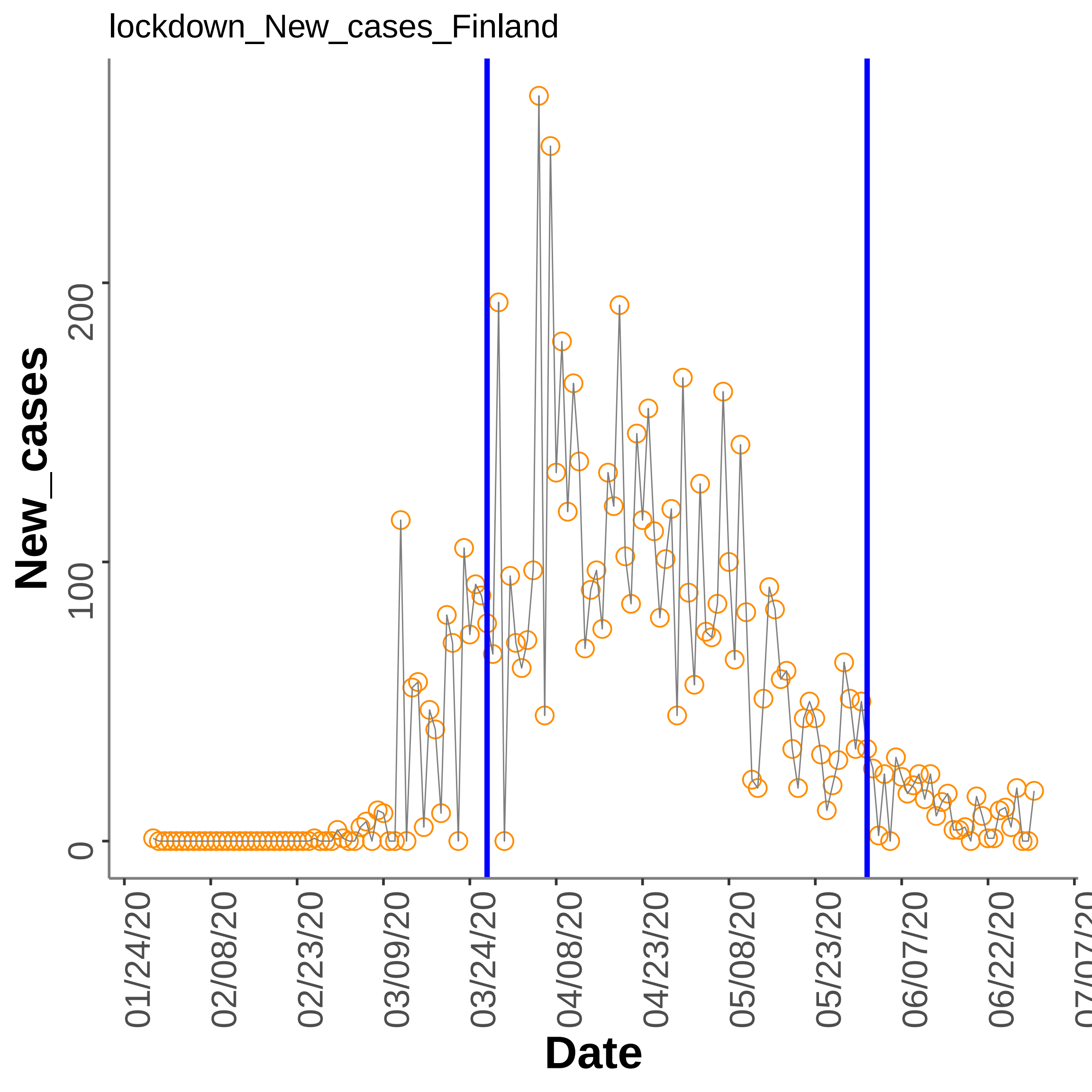
lockdown_New_cases_Finland
New_cases
0
100
200
Date
01/24/20
02/08/20
02/23/20
03/09/20
03/24/20
04/08/20
04/23/20
05/08/20
05/23/20
06/07/20
06/22/20

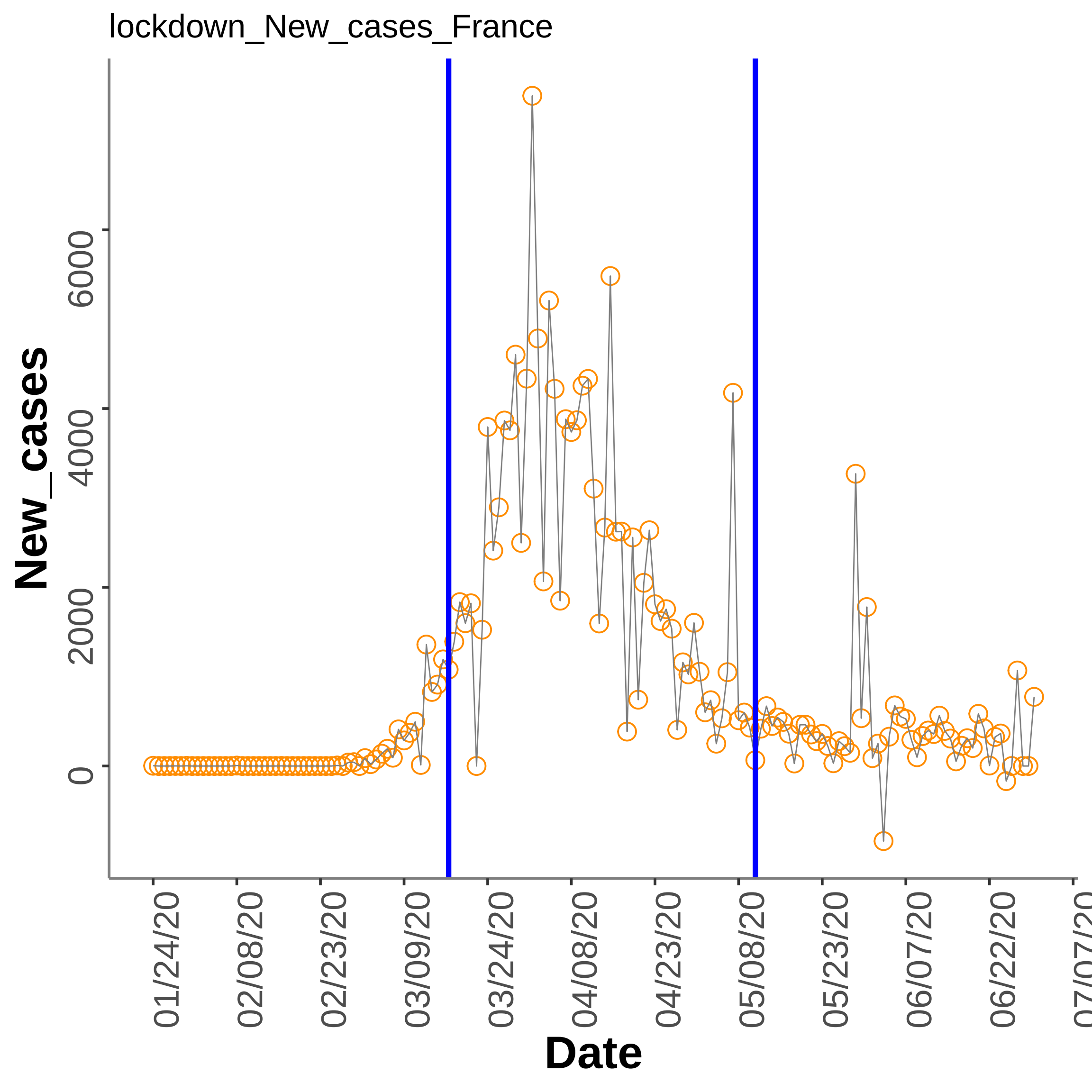

lockdown_New_cases_France
New_cases
0
2000
4000
6000
Date
01/24/20
02/08/20
02/23/20
03/09/20
03/24/20
04/08/20
04/23/20
05/08/20
05/23/20
06/07/20
06/22/20
07/07/20

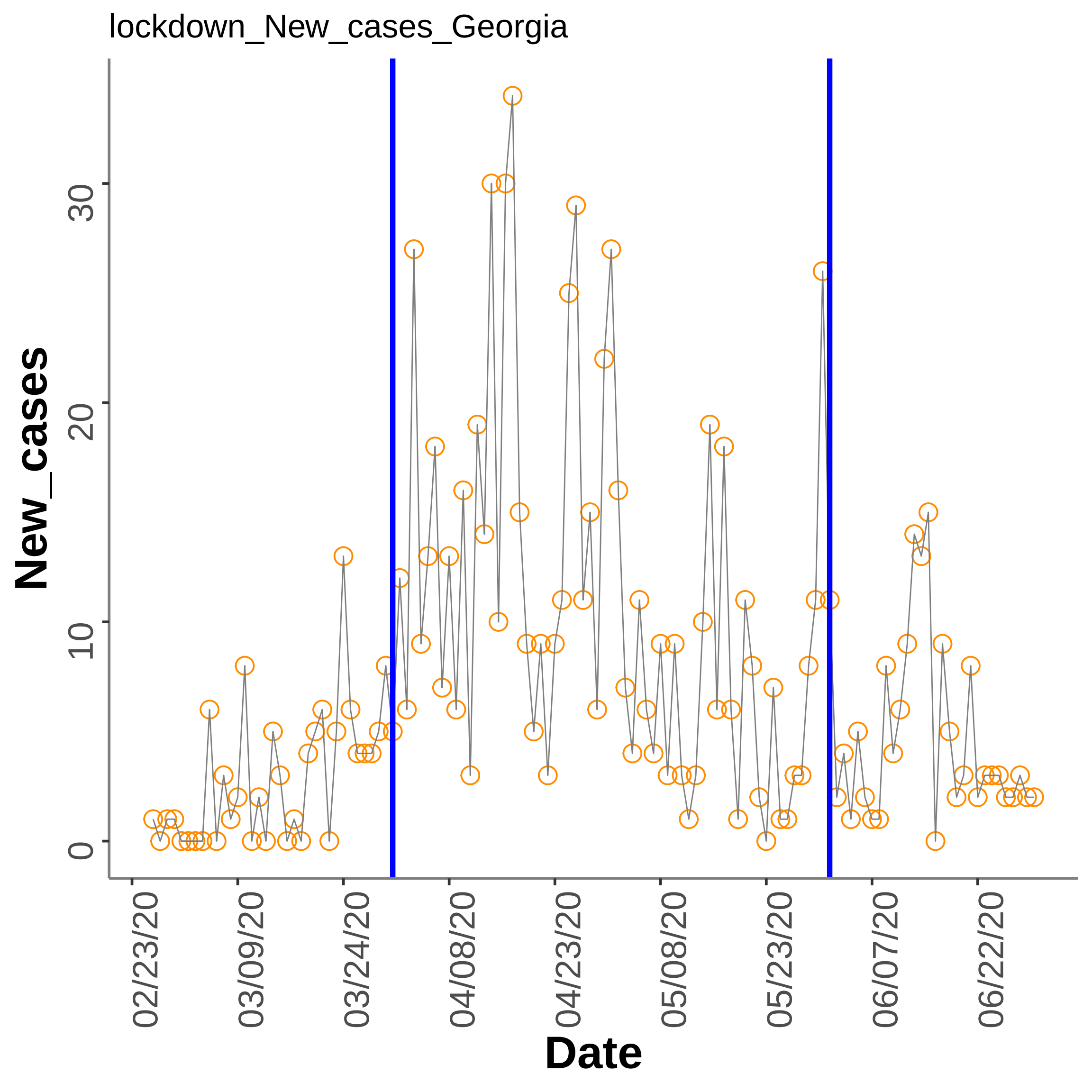

lockdown_New_cases_Georgia
New_cases
0
10
20
30
Date
02/23/20
03/09/20
03/24/20
04/08/20
04/23/20
05/08/20
05/23/20
06/07/20
06/22/20

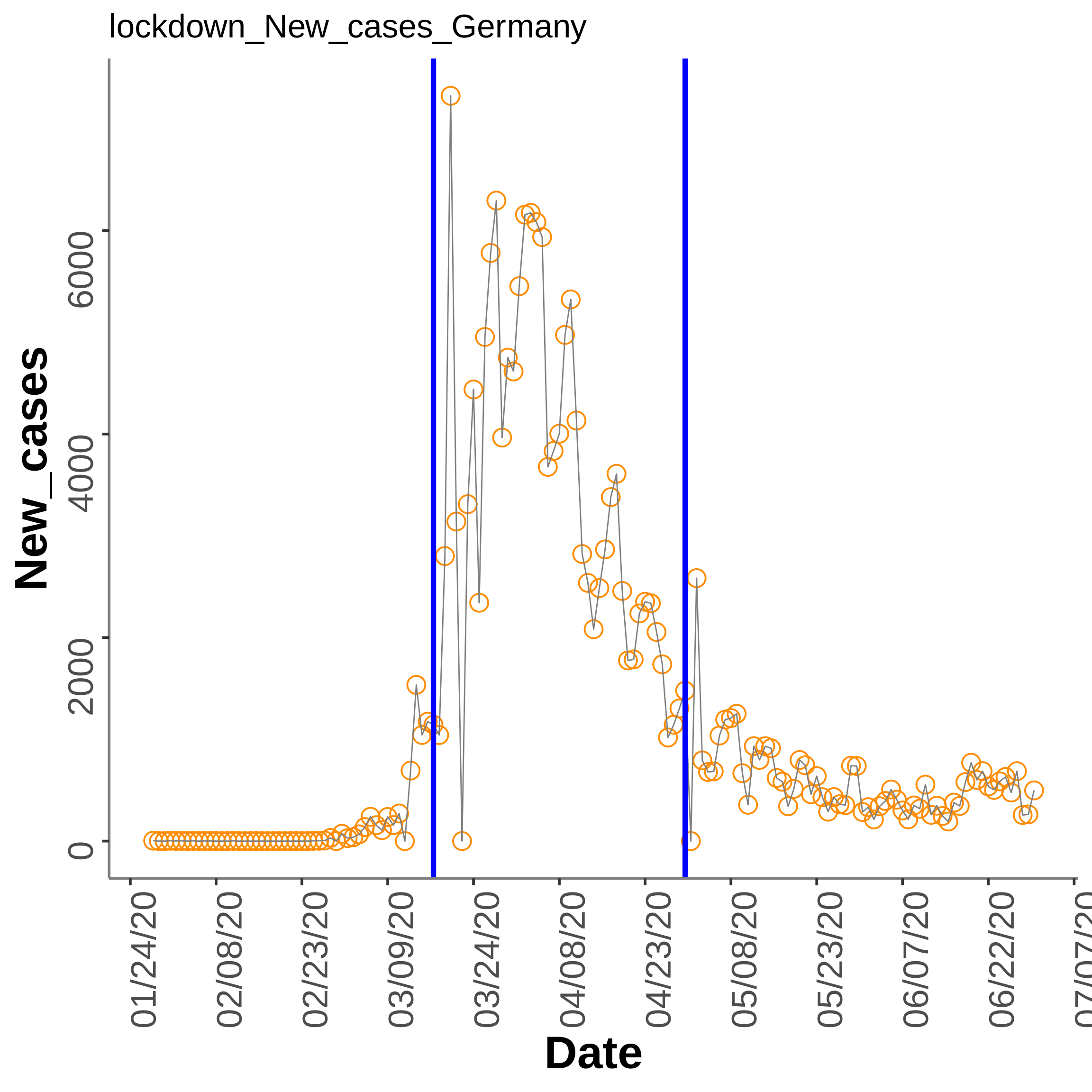
lockdown_New_cases_Germany
New_cases
0
2000
4000
6000
Date
01/24/20
02/08/20
02/23/20
03/09/20
03/24/20
04/08/20
04/23/20
05/08/20
05/23/20
06/07/20
06/22/20
07/07/20

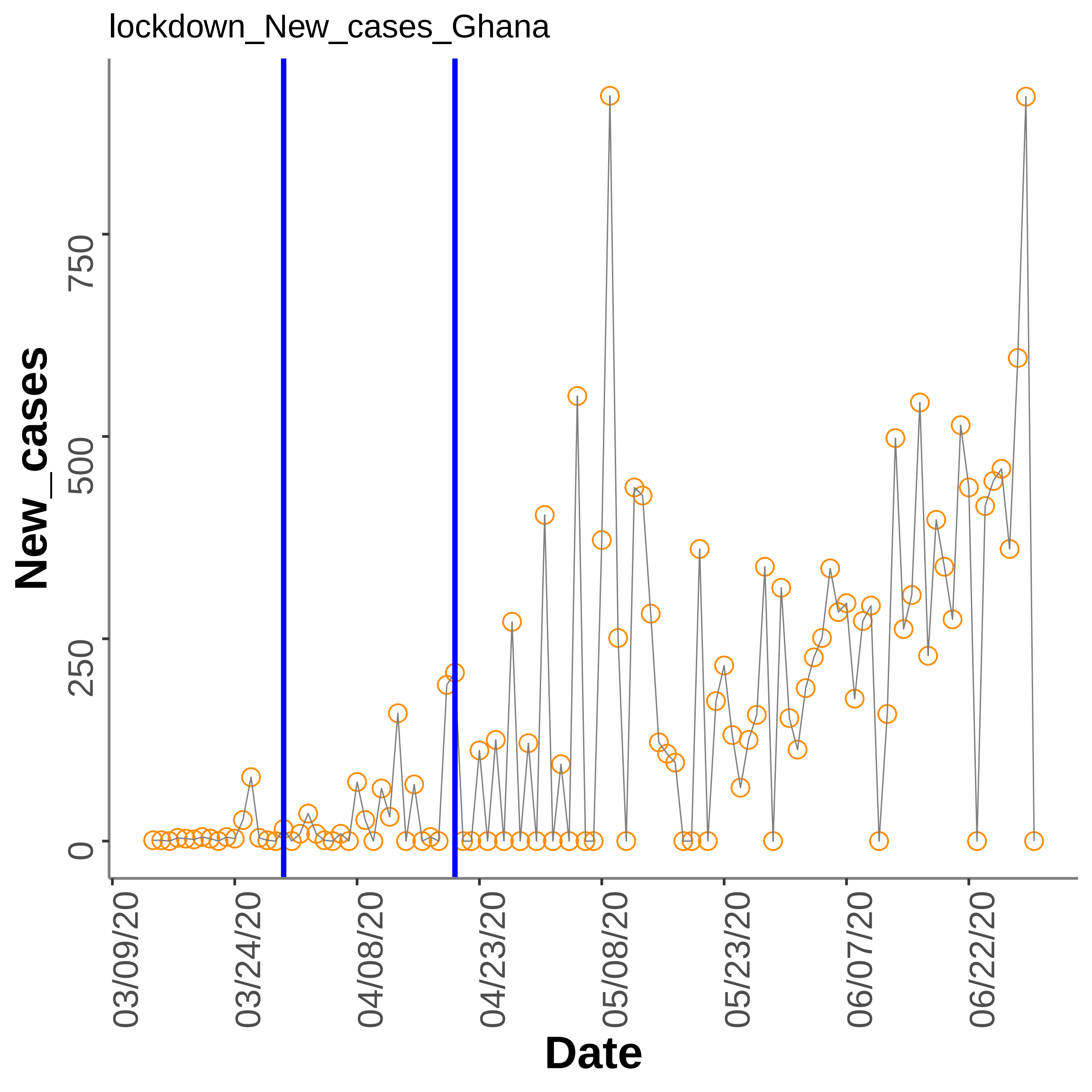
lockdown_New_cases_Ghana
New_cases
0
250
500
750
Date
03/09/20
03/24/20
04/08/20
04/23/20
05/08/20
05/23/20
06/07/20
06/22/20

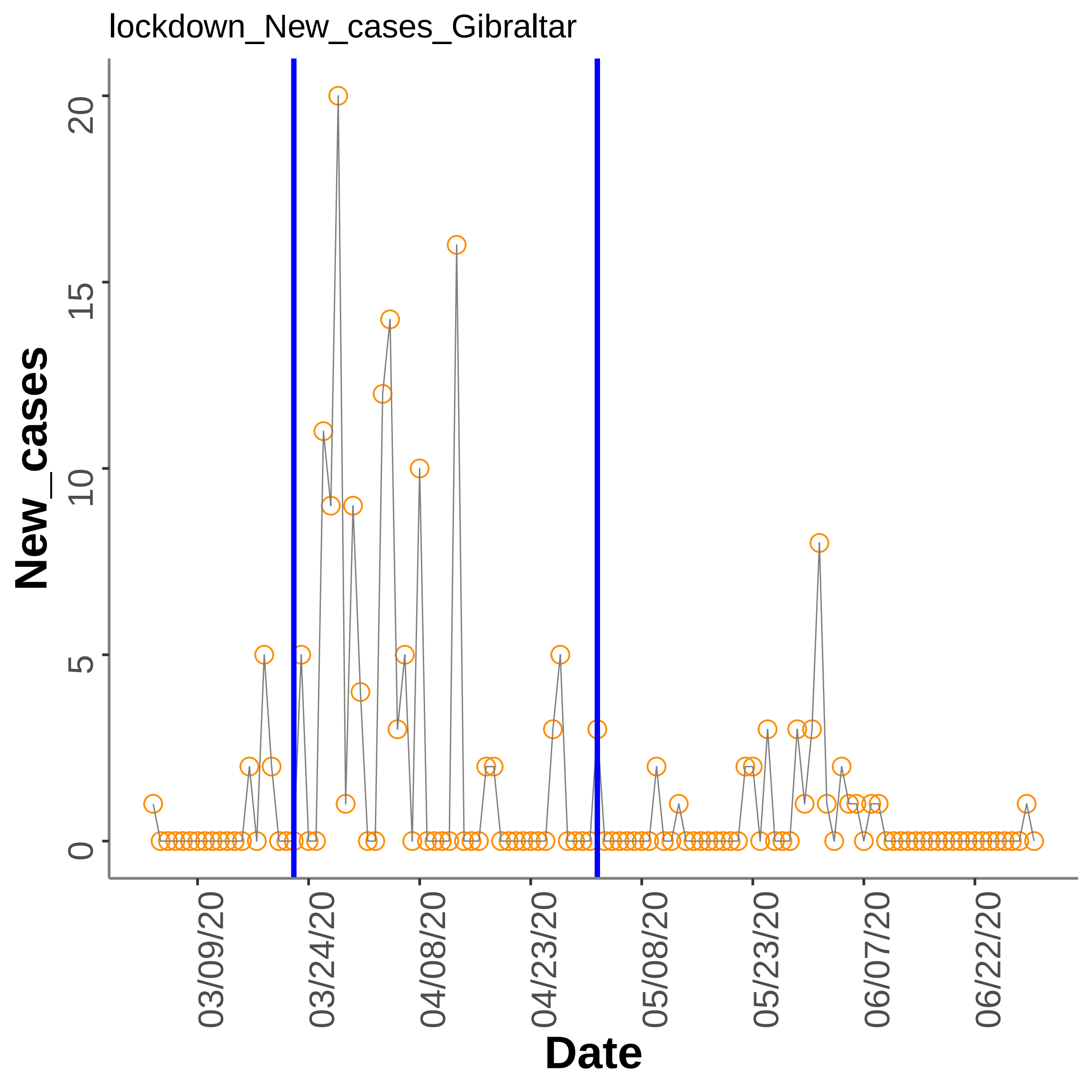
lockdown_New_cases_Gibraltar
New_cases
0
5
10
15
20
03/09/20
03/24/20
04/08/20
04/23/20
05/08/20
05/23/20
06/07/20
06/22/20
Date

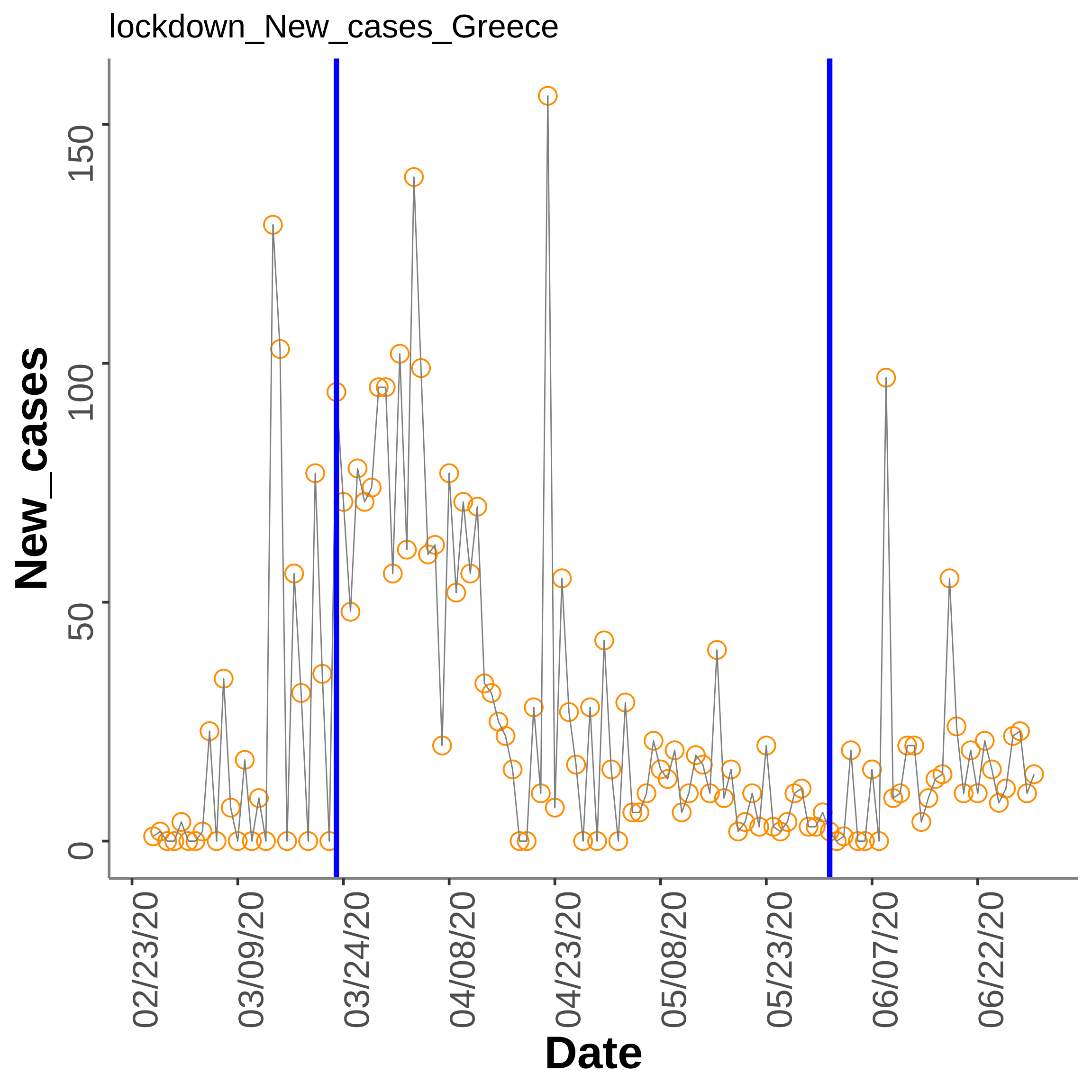

lockdown_New_cases_Greece
New_cases
0
50
100
150
Date
02/23/20
03/09/20
03/24/20
04/08/20
04/23/20
05/08/20
05/23/20
06/07/20
06/22/20

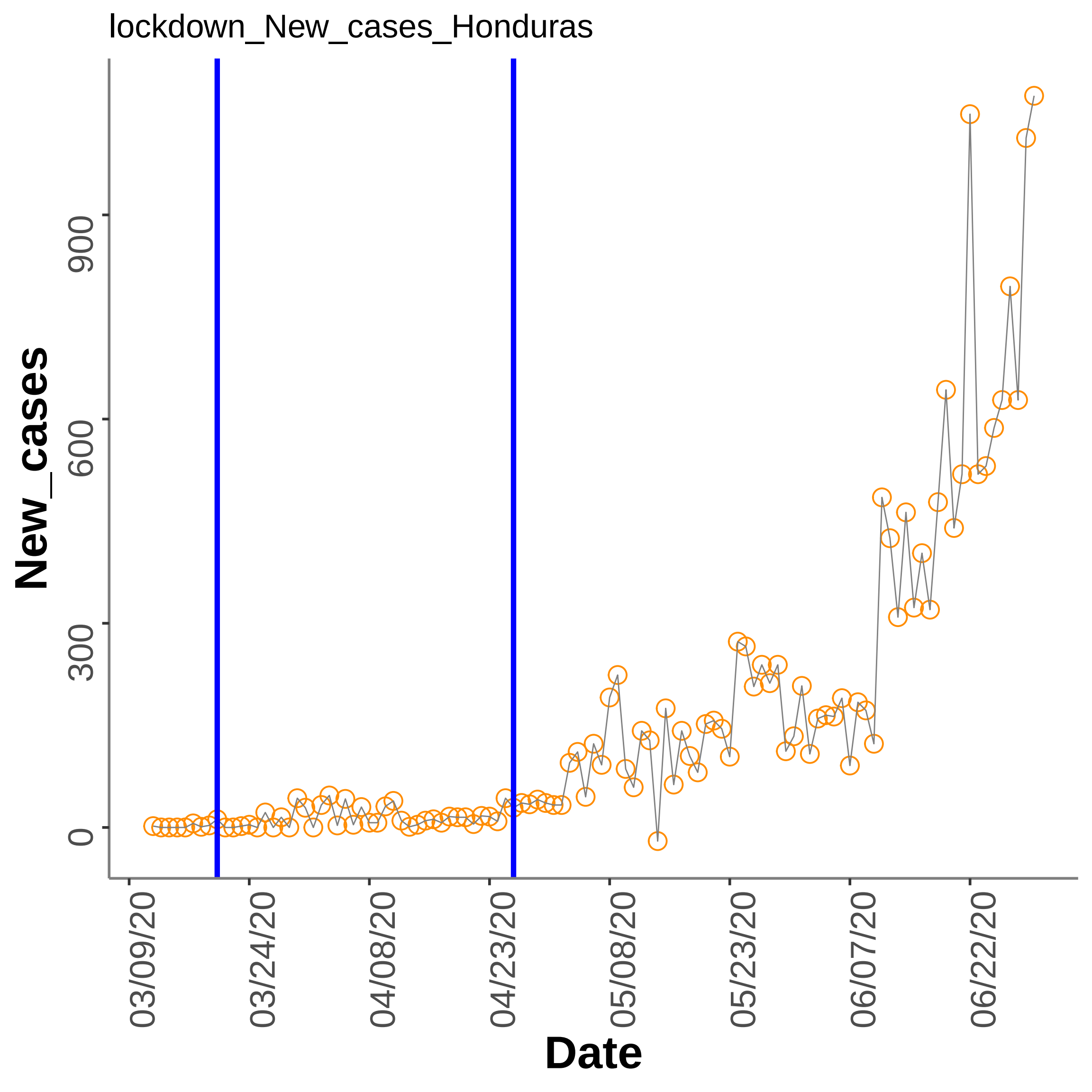
lockdown_New_cases_Honduras
New_cases
0
300
600
900
Date
03/09/20
03/24/20
04/08/20
04/23/20
05/08/20
05/23/20
06/07/20
06/22/20

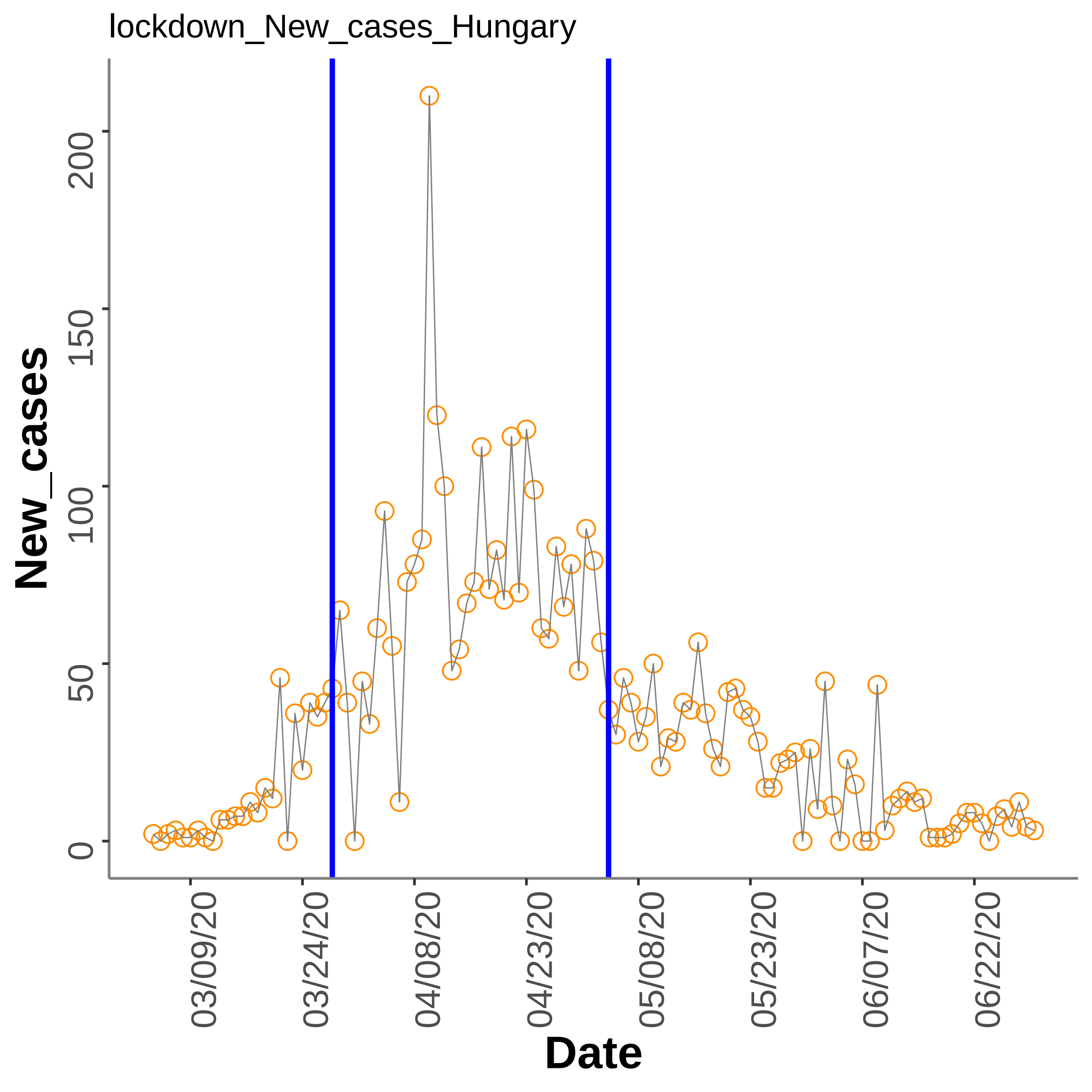

lockdown_New_cases_Hungary
New_cases
0
50
100
150
200
03/09/20
03/24/20
04/08/20
04/23/20
05/08/20
05/23/20
06/07/20
06/22/20
Date

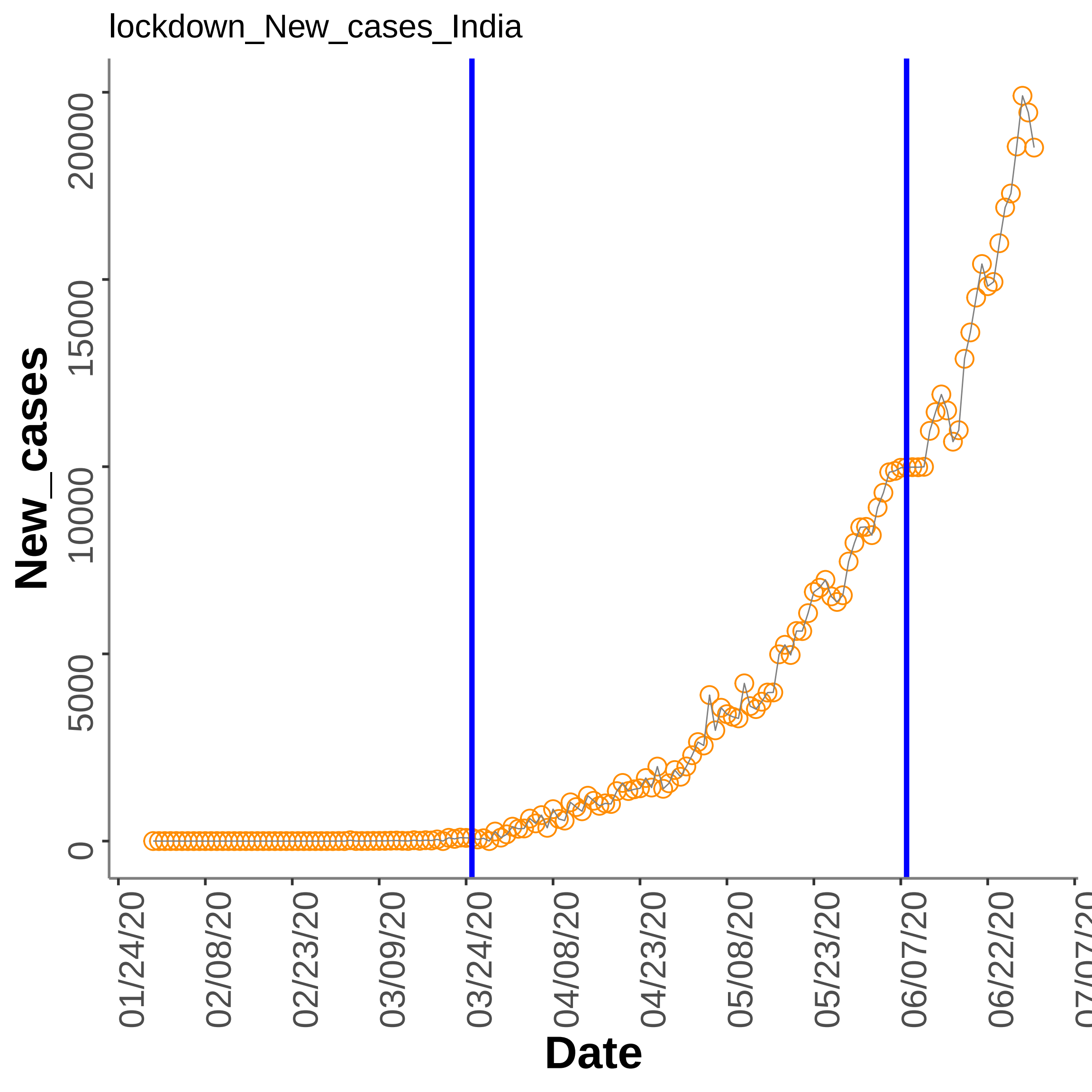
lockdown_New_cases_India
New_cases
20000
15000
10000
5000
0
01/24/20
02/08/20
02/23/20
03/09/20
03/24/20
04/08/20
04/23/20
05/08/20
05/23/20
06/07/20
06/22/20
07/07/20
Date

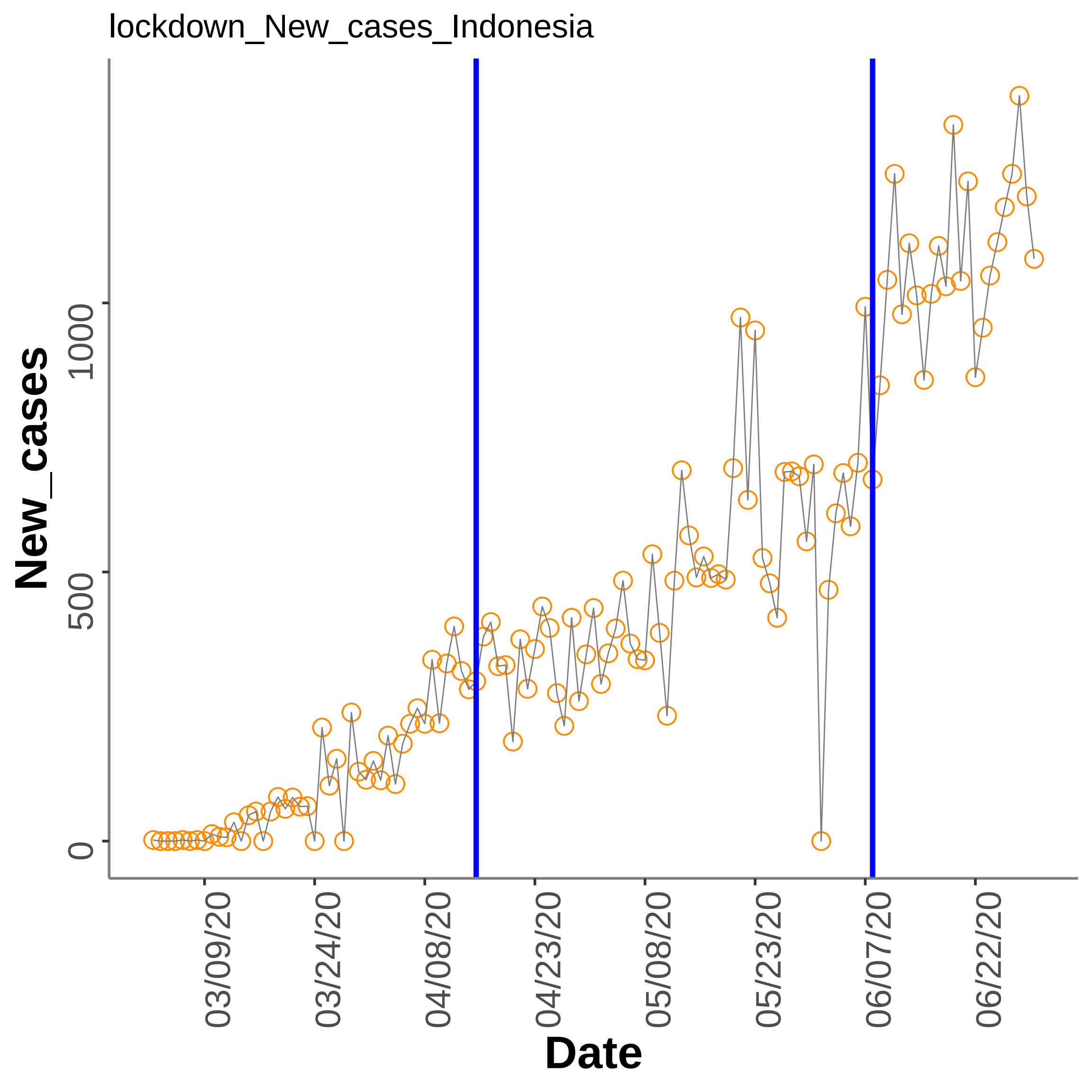

lockdown_New_cases_Indonesia
New_cases
0
500
1000
03/09/20
03/24/20
04/08/20
04/23/20
05/08/20
05/23/20
06/07/20
06/22/20
Date

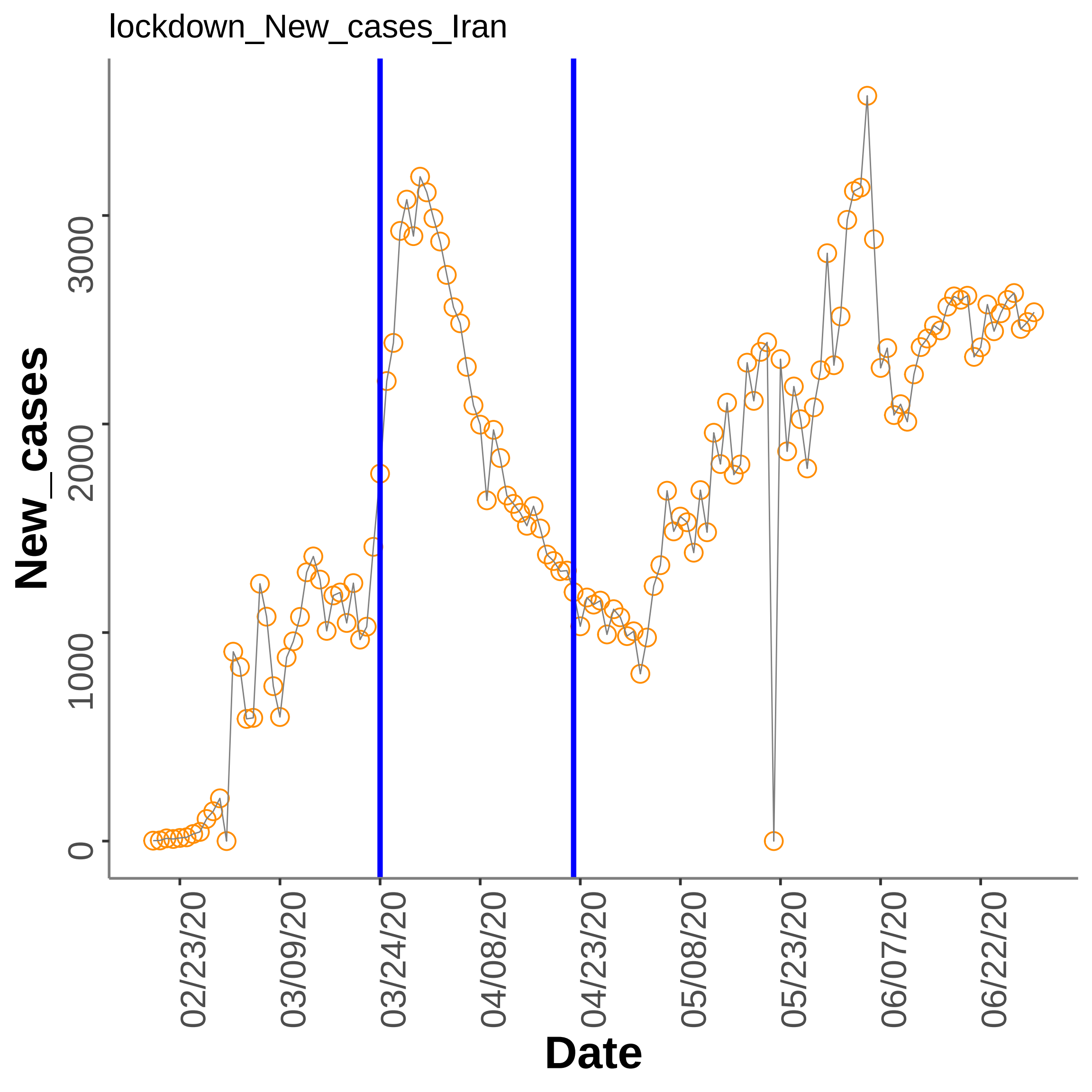
lockdown_New_cases_Iran
New_cases
0
1000
2000
3000
Date
02/23/20
03/09/20
03/24/20
04/08/20
04/23/20
05/08/20
05/23/20
06/07/20
06/22/20

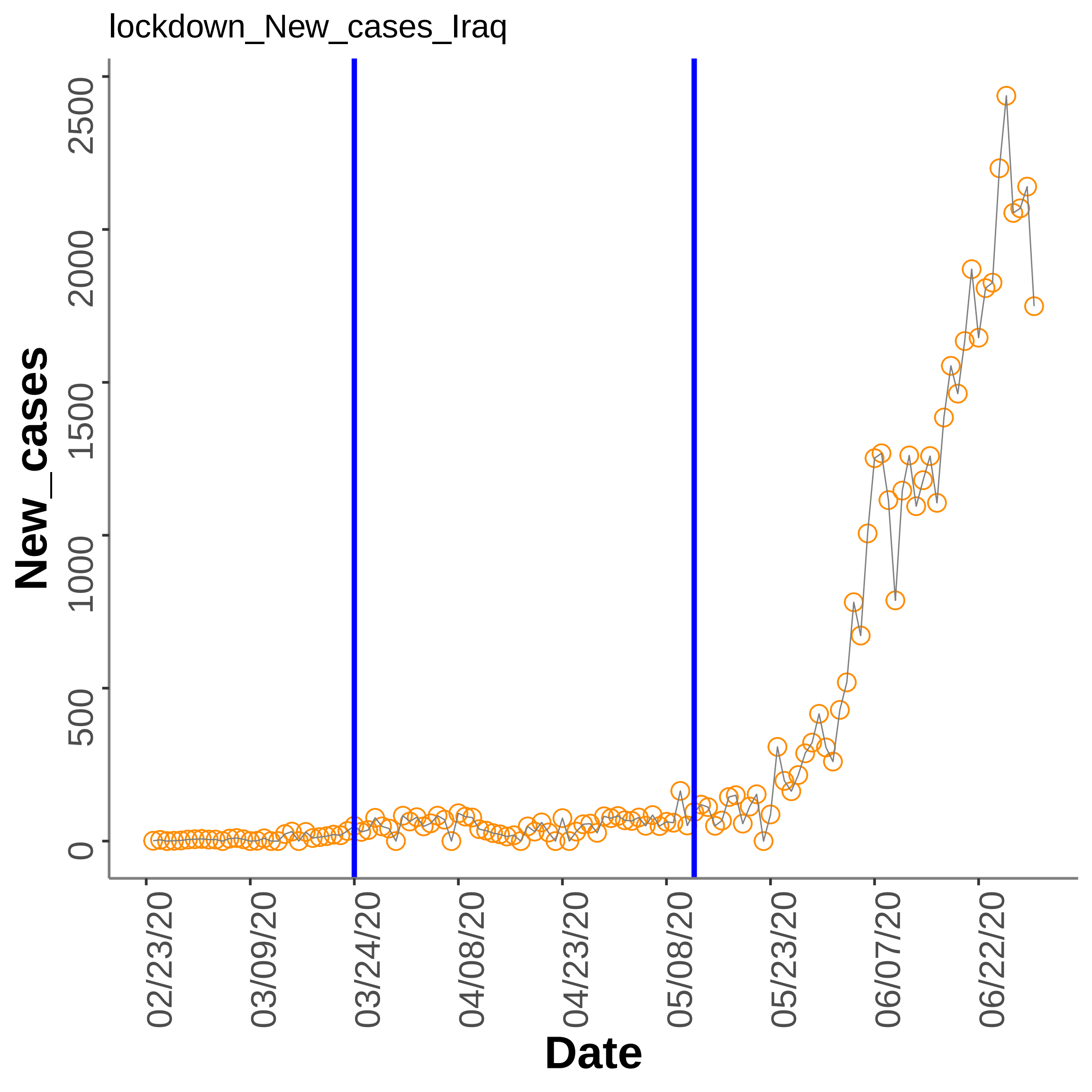

lockdown_New_cases_Iraq
New_cases
0
500
1000
1500
2000
2500
Date
02/23/20
03/09/20
03/24/20
04/08/20
04/23/20
05/08/20
05/23/20
06/07/20
06/22/20

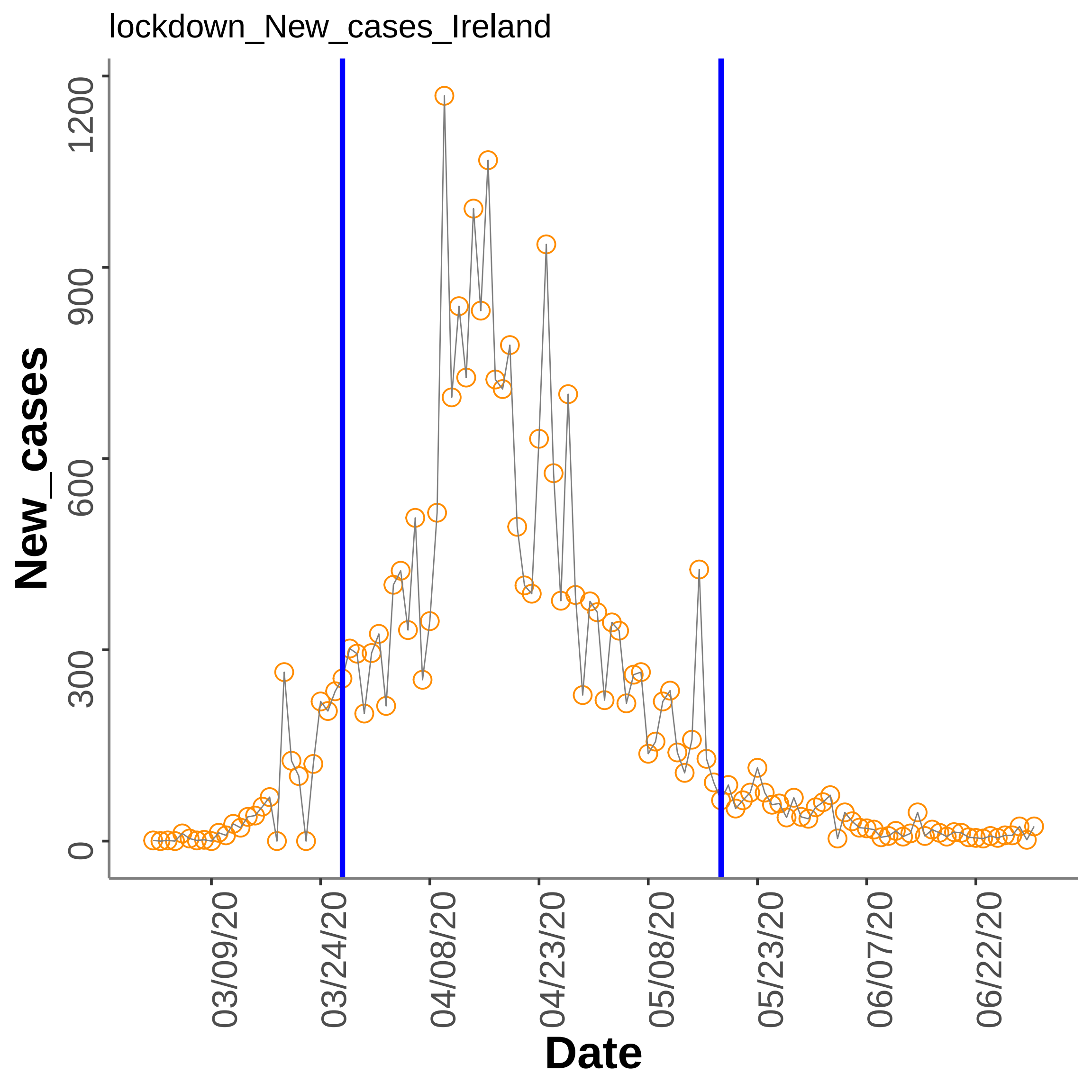
lockdown_New_cases_Ireland
New_cases
0
300
600
900
1200
Date
03/09/20
03/24/20
04/08/20
04/23/20
05/08/20
05/23/20
06/07/20
06/22/20

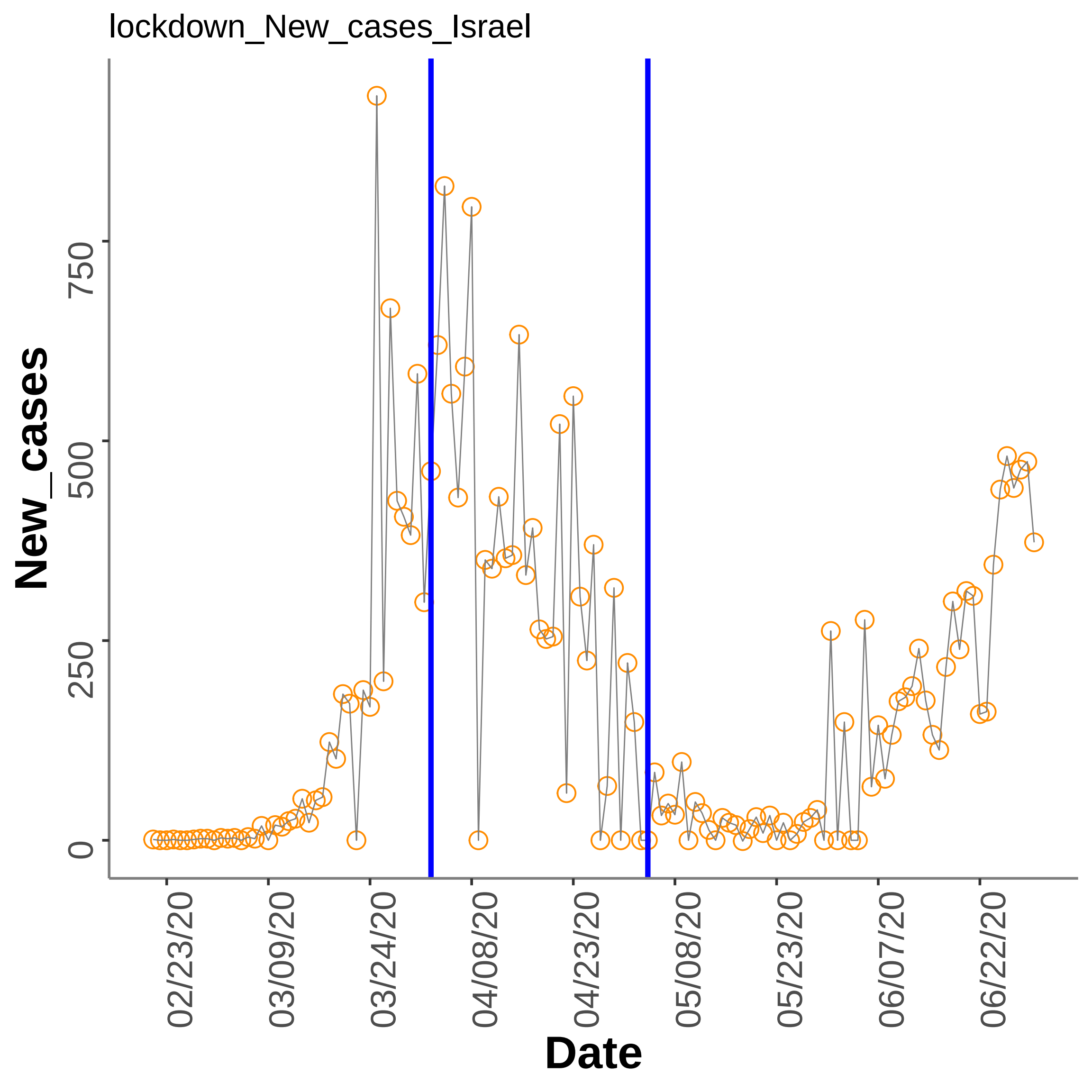

lockdown_New_cases_Israel
New_cases
0
250
500
750
Date
02/23/20
03/09/20
03/24/20
04/08/20
04/23/20
05/08/20
05/23/20
06/07/20
06/22/20

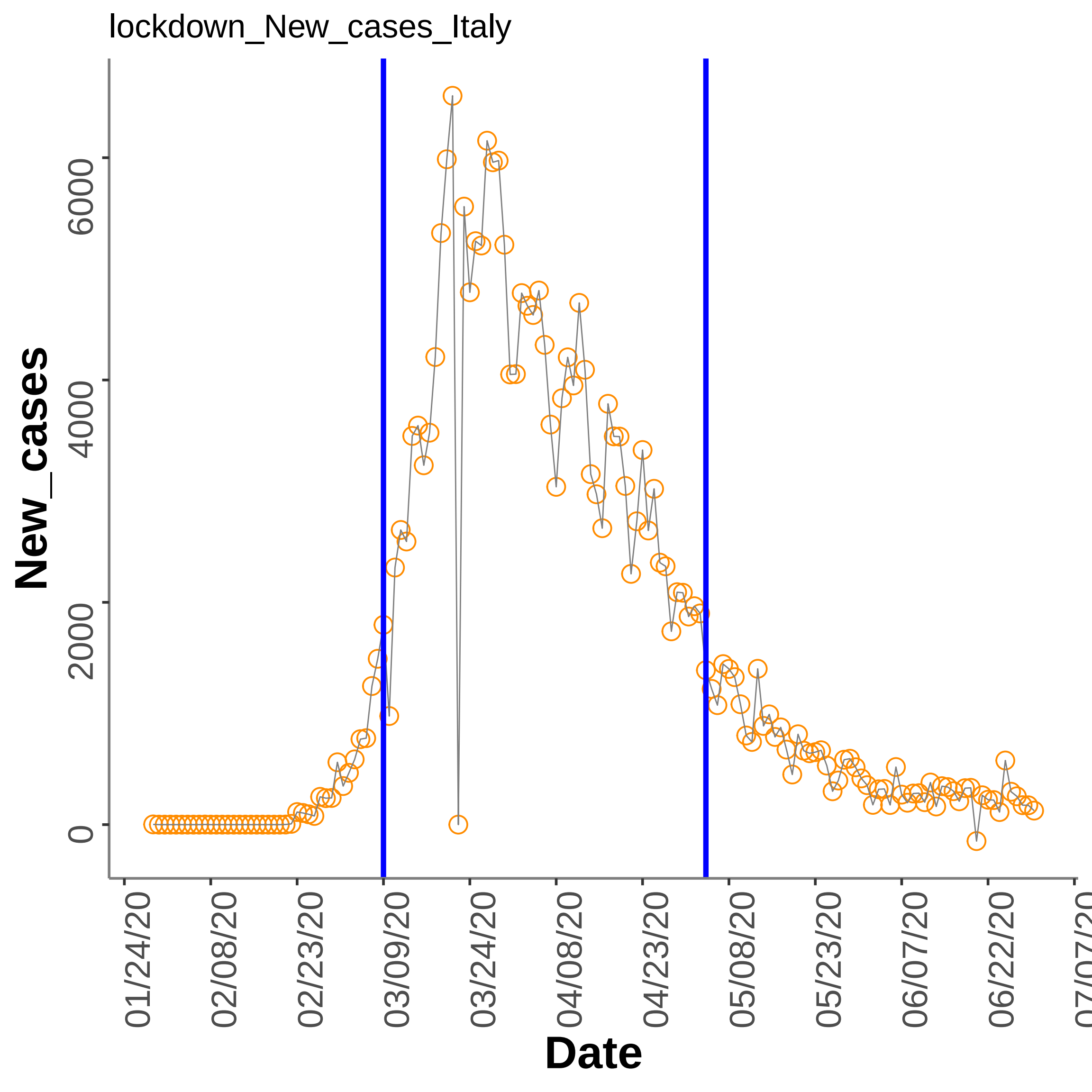

lockdown_New_cases_Italy
New_cases
0
2000
4000
6000
Date
01/24/20
02/08/20
02/23/20
03/09/20
03/24/20
04/08/20
04/23/20
05/08/20
05/23/20
06/07/20
06/22/20
07/07/20

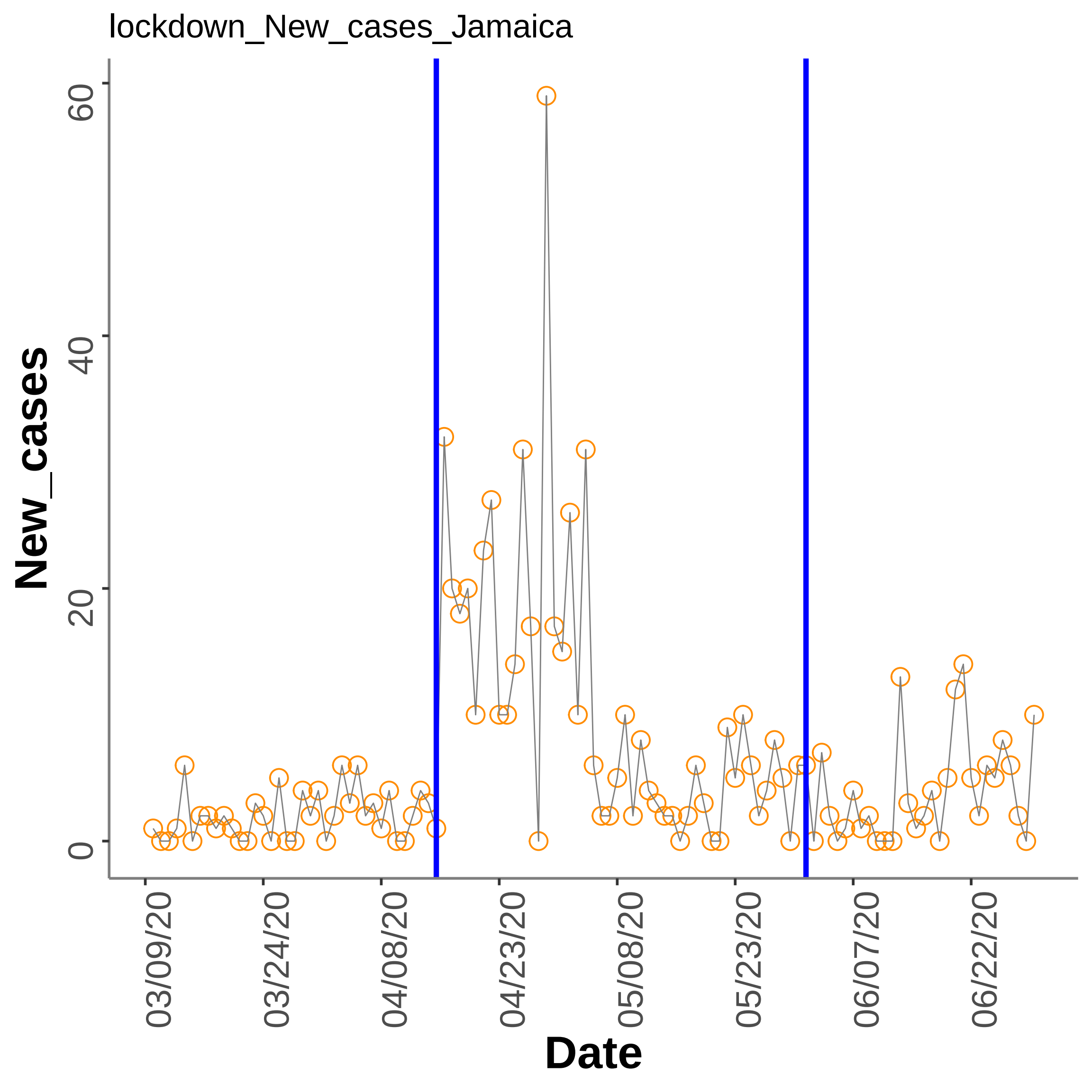

lockdown_New_cases_Jamaica
New_cases
60
40
20
0
03/09/20
03/24/20
04/08/20
04/23/20
05/08/20
05/23/20
06/07/20
06/22/20
Date

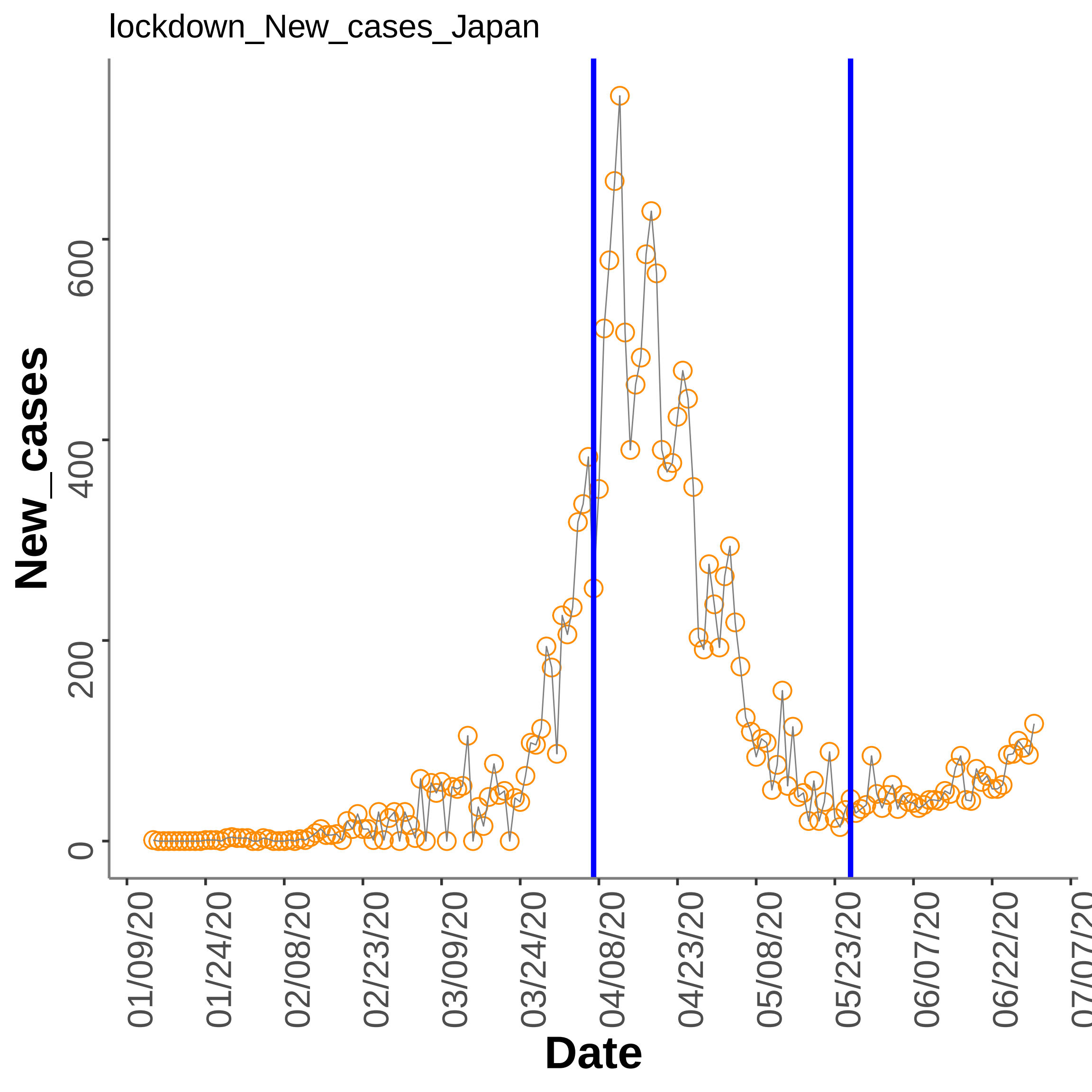
lockdown_New_cases_Japan
New_cases
0
200
400
600
Date
01/09/20
01/24/20
02/08/20
02/23/20
03/09/20
03/24/20
04/08/20
04/23/20
05/08/20
05/23/20
06/07/20
06/22/20
07/07/20

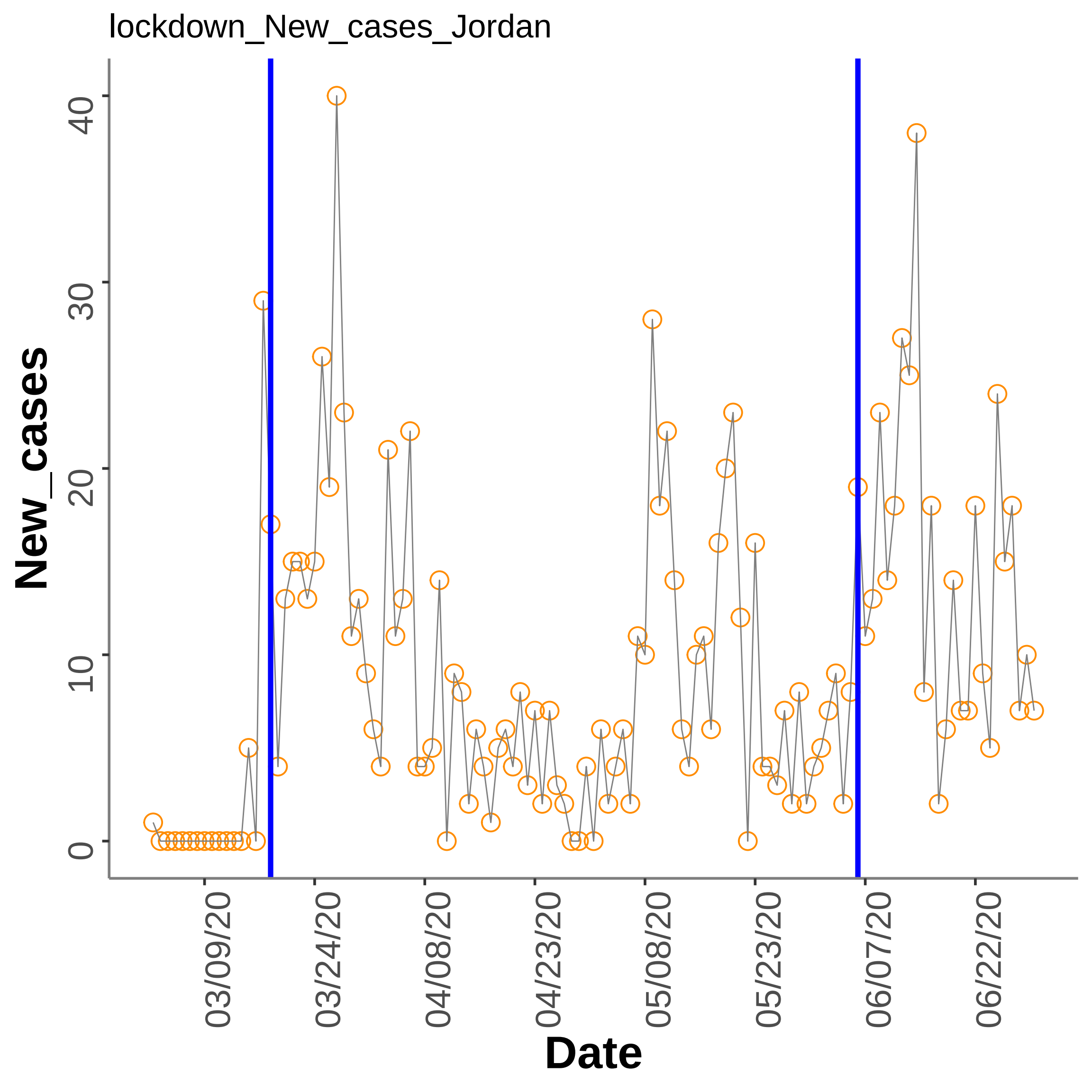
lockdown_New_cases_Jordan
New_cases
Date
0
10
20
30
40
03/09/20
03/24/20
04/08/20
04/23/20
05/08/20
05/23/20
06/07/20
06/22/20

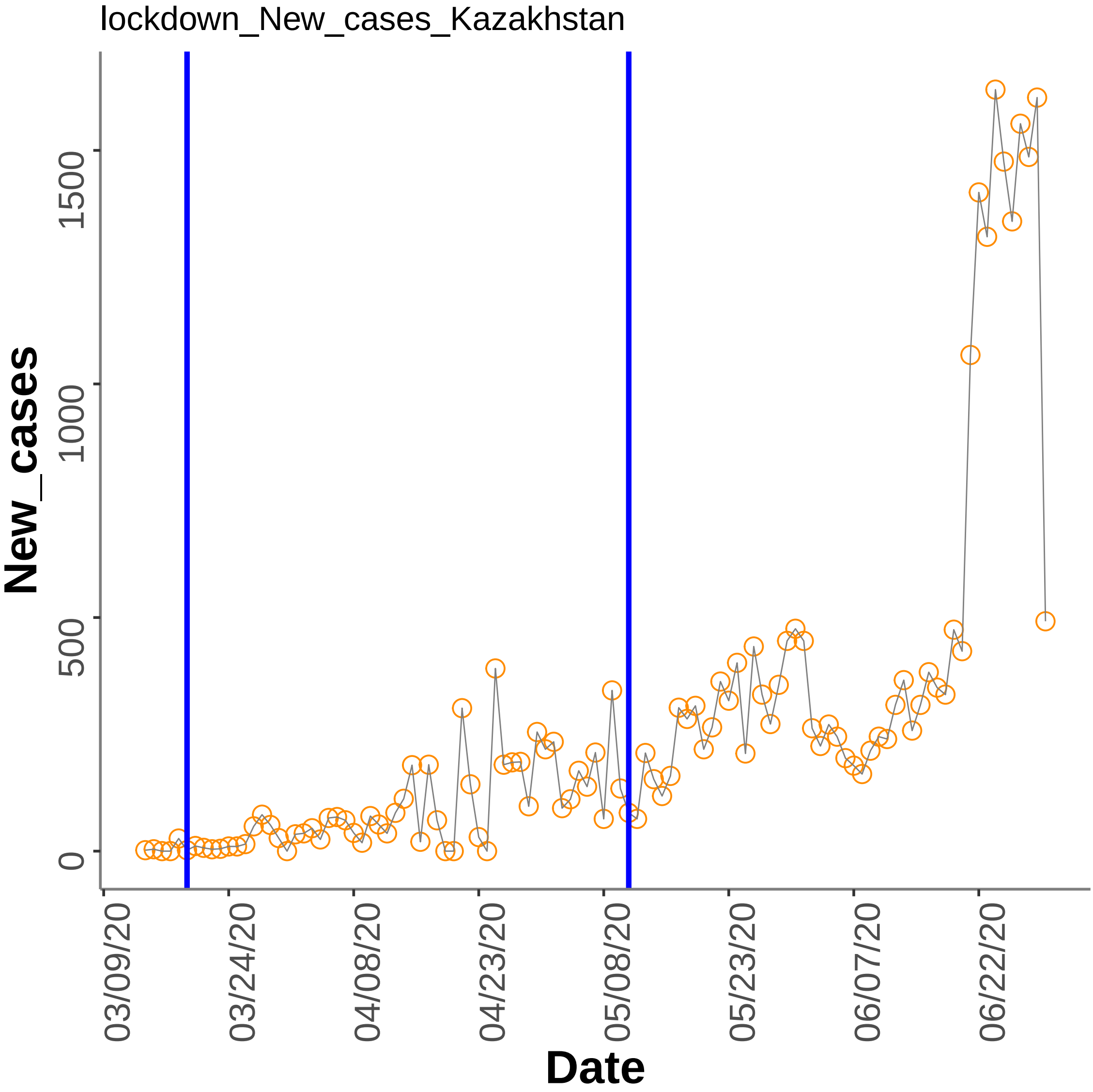

lockdown_New_cases_Kazakhstan
New_cases
0
500
1000
1500
Date
03/09/20
03/24/20
04/08/20
04/23/20
05/08/20
05/23/20
06/07/20
06/22/20

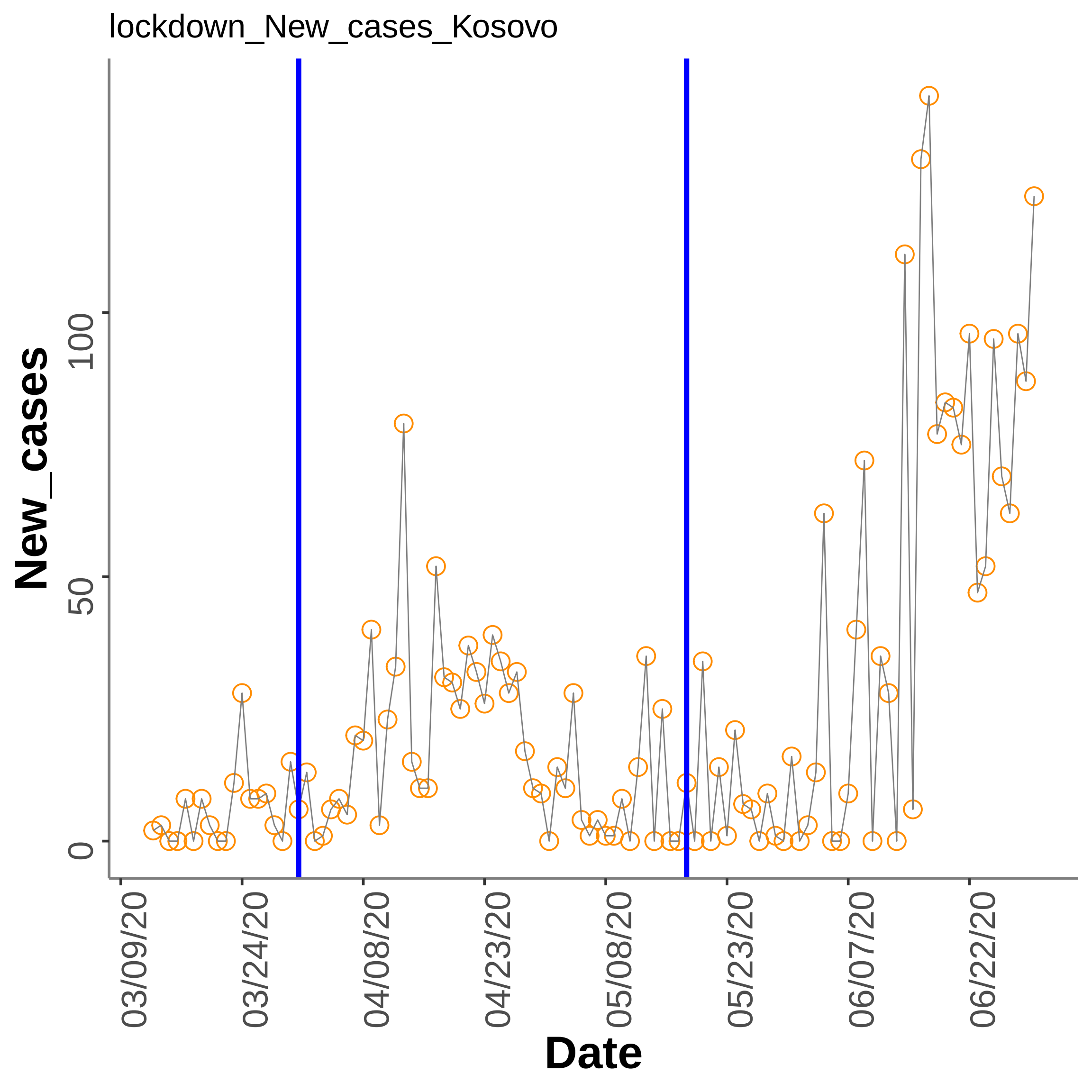
lockdown_New_cases_Kosovo
New_cases
0
50
100
Date
03/09/20
03/24/20
04/08/20
04/23/20
05/08/20
05/23/20
06/07/20
06/22/20

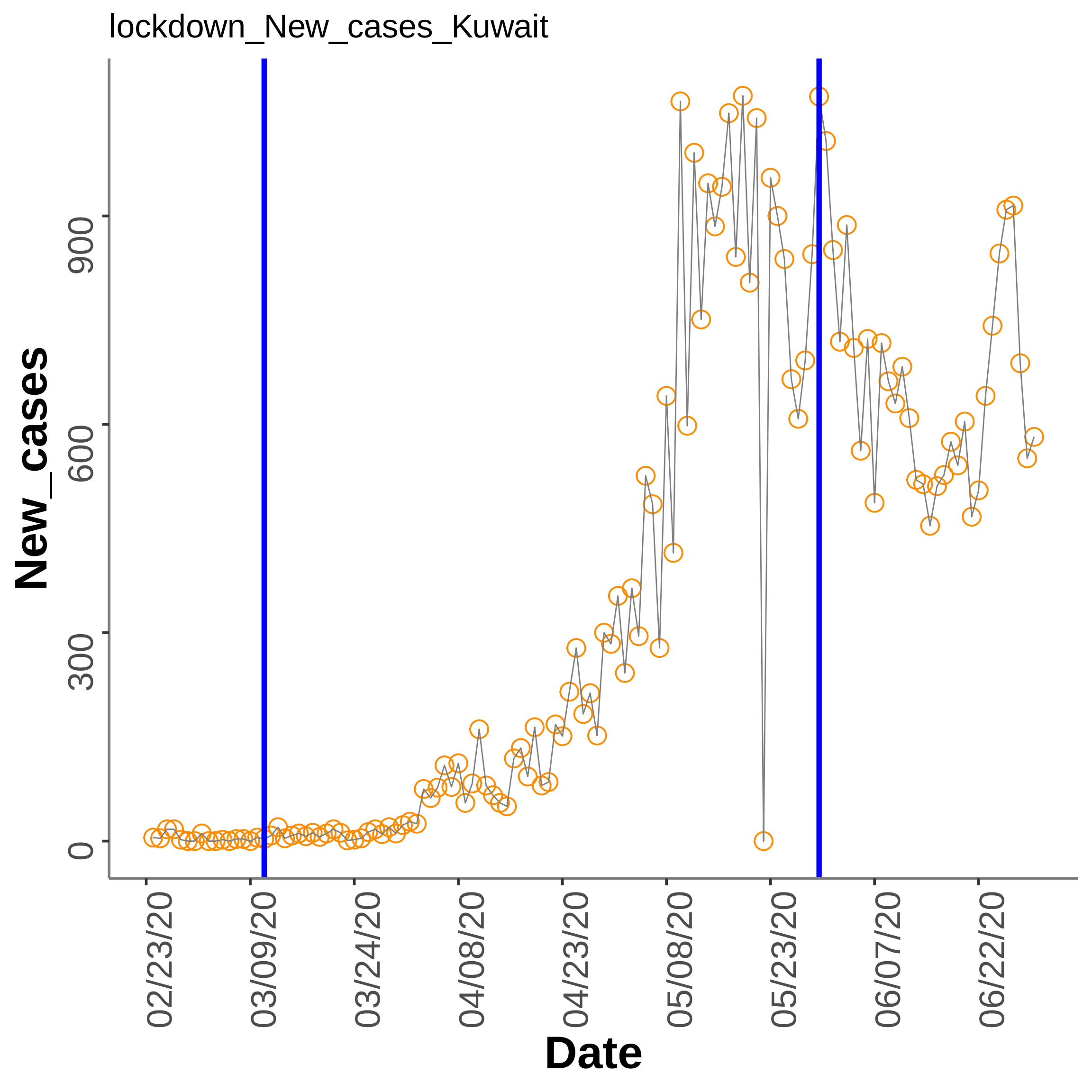

lockdown_New_cases_Kuwait
New_cases
0
300
600
900
Date
02/23/20
03/09/20
03/24/20
04/08/20
04/23/20
05/08/20
05/23/20
06/07/20
06/22/20

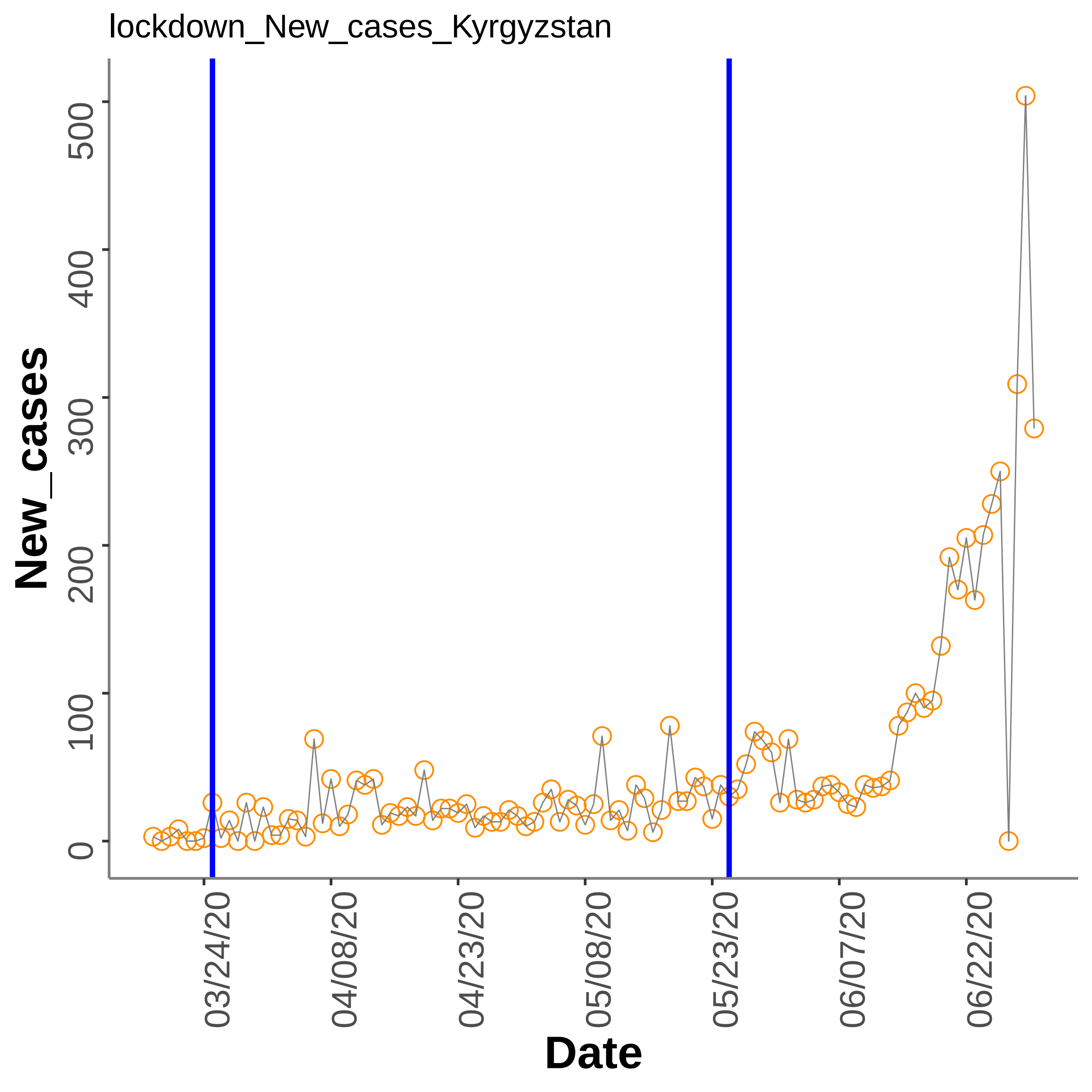
lockdown_New_cases_Kyrgyzstan
New_cases
0
100
200
300
400
500
03/24/20
04/08/20
04/23/20
05/08/20
05/23/20
06/07/20
06/22/20
Date

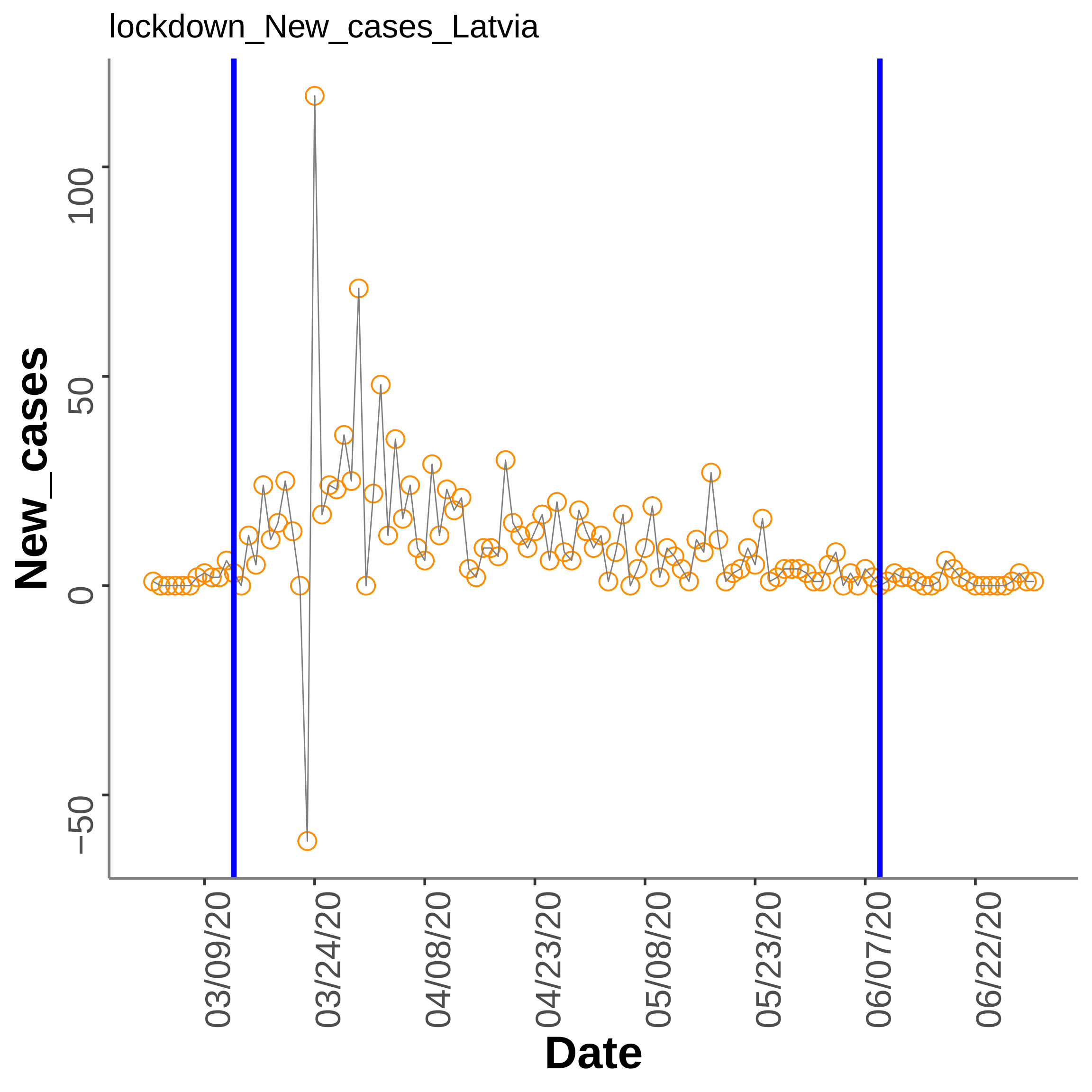

lockdown_New_cases_Latvia
New_cases
100
50
0
−50
03/09/20
03/24/20
04/08/20
04/23/20
05/08/20
05/23/20
06/07/20
06/22/20
Date

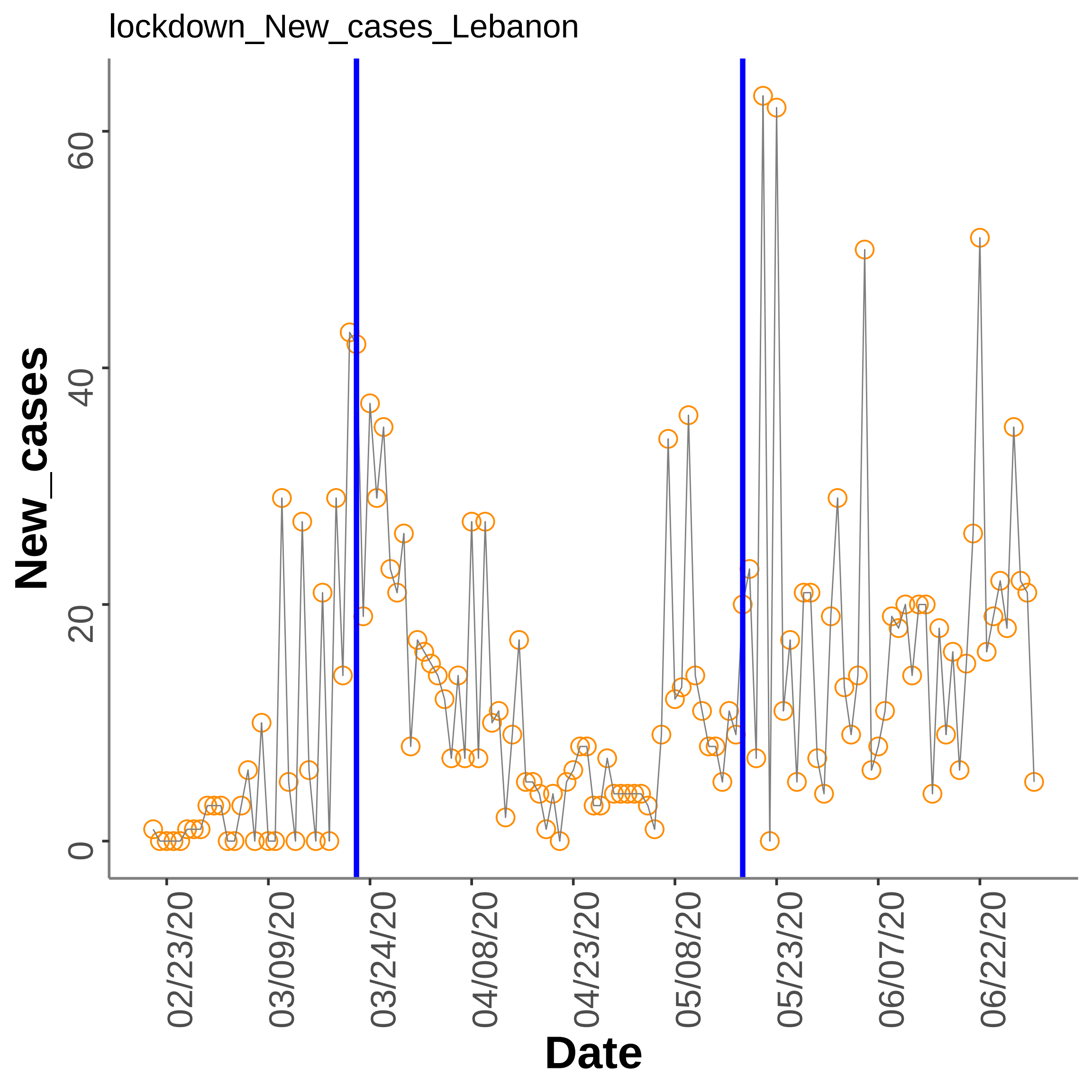

lockdown_New_cases_Lebanon
New_cases
60
40
20
0
02/23/20
03/09/20
03/24/20
04/08/20
04/23/20
05/08/20
05/23/20
06/07/20
06/22/20
Date

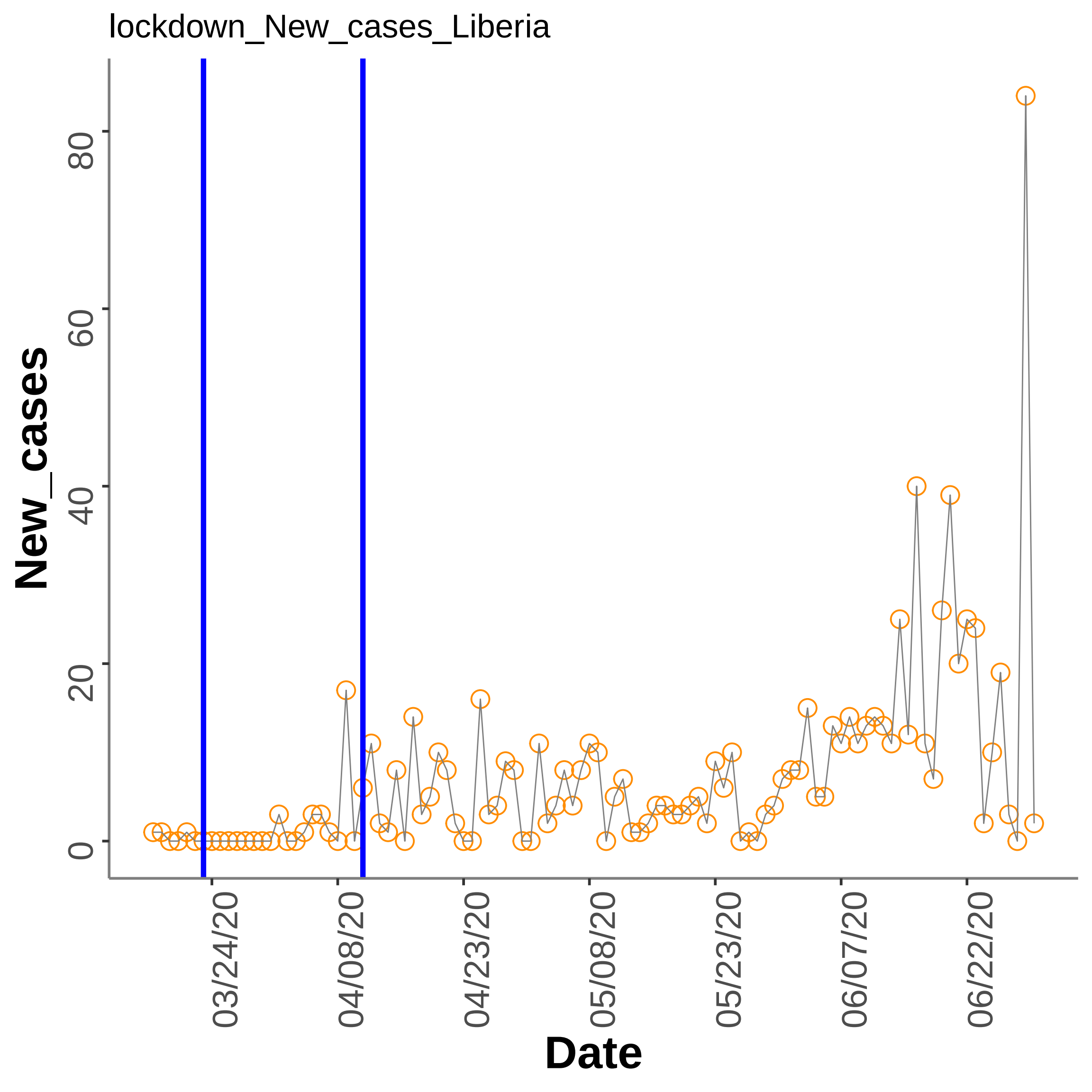

lockdown_New_cases_Liberia
New_cases
0
20
40
60
80
03/24/20
04/08/20
04/23/20
05/08/20
05/23/20
06/07/20
06/22/20
Date

lockdown_New_cases_Lithuania

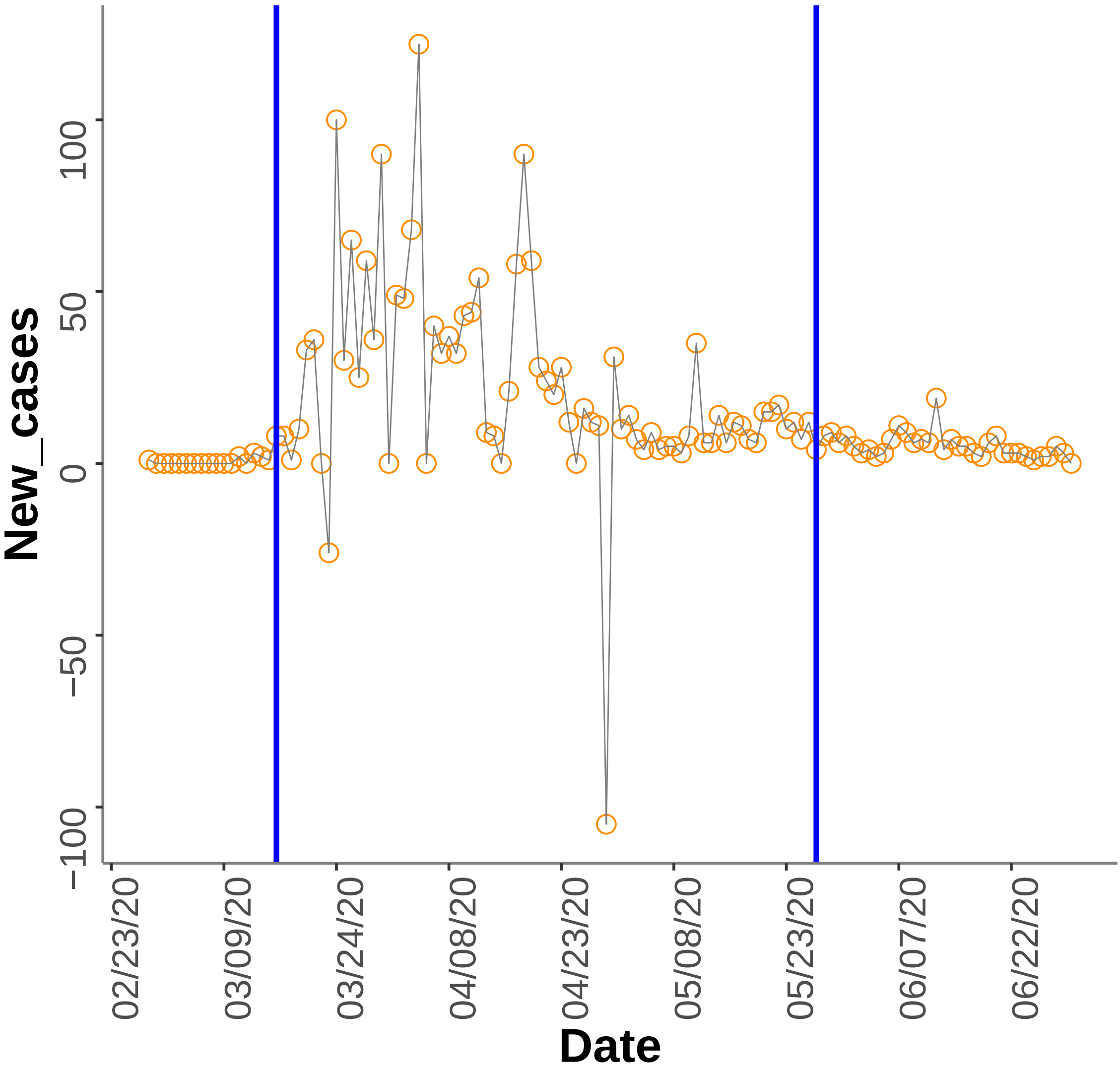

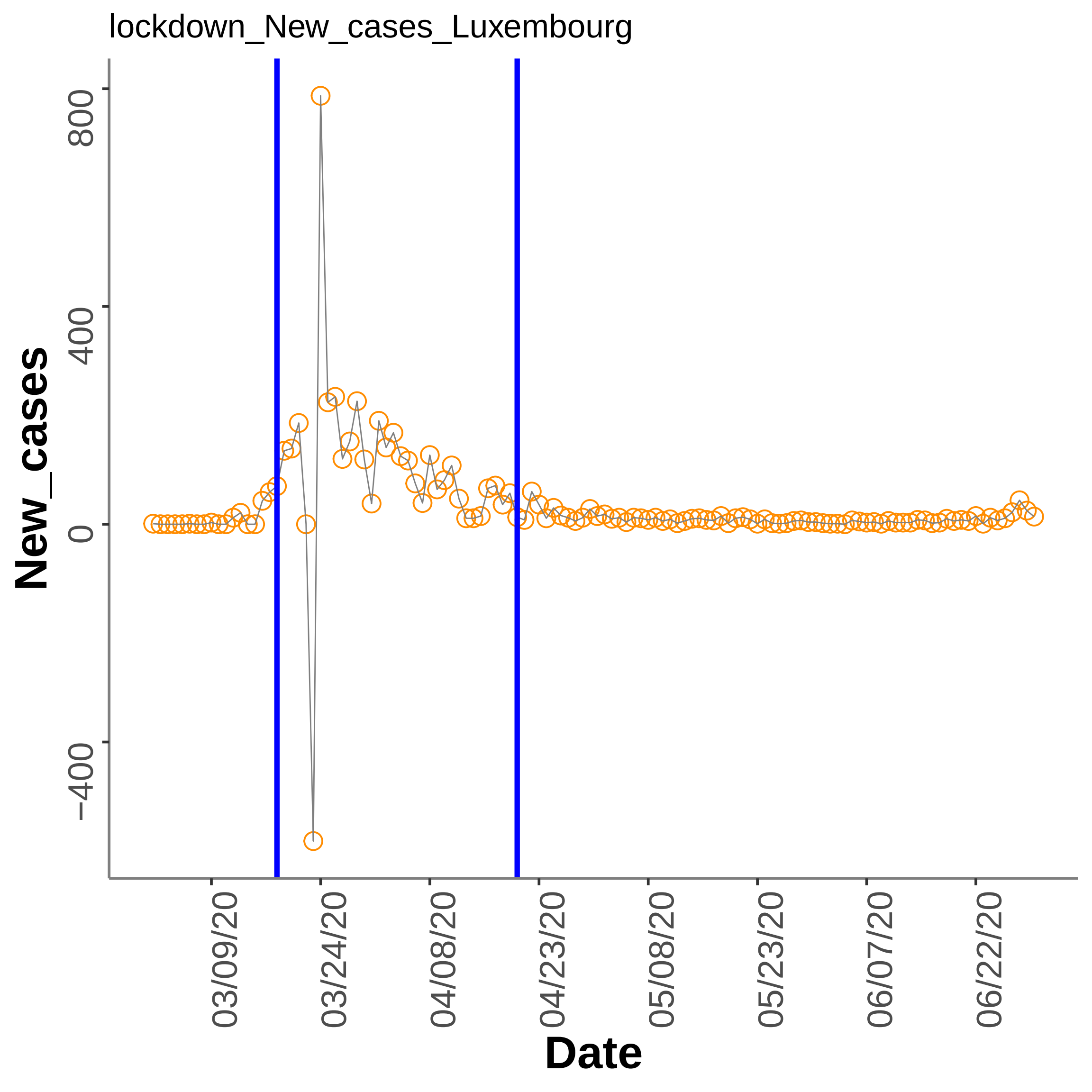
lockdown_New_cases_Luxembourg
New_cases
800
400
0
−400
Date
03/09/20
03/24/20
04/08/20
04/23/20
05/08/20
05/23/20
06/07/20
06/22/20

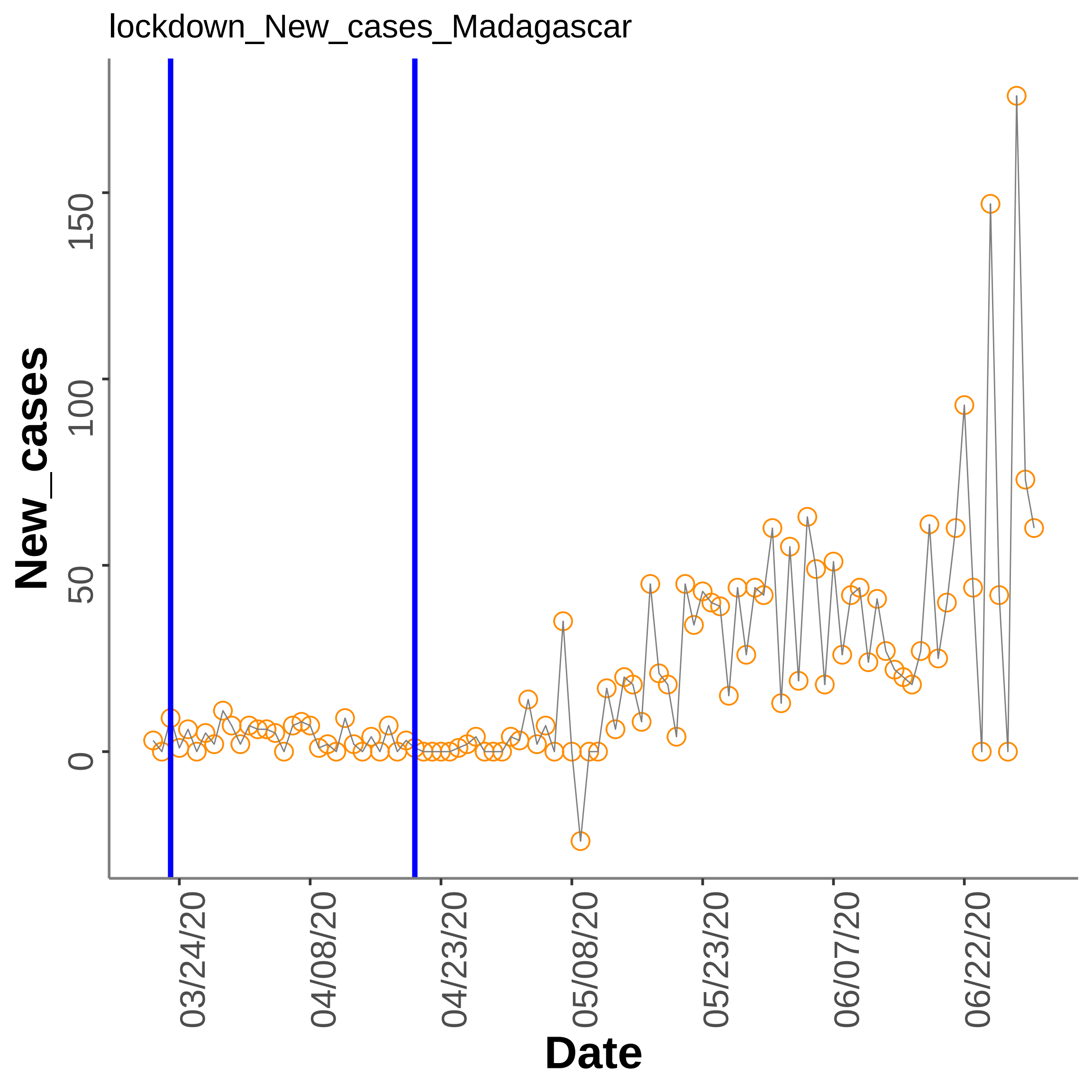
lockdown_New_cases_Madagascar
New_cases
Date
0
50
100
150
03/24/20
04/08/20
04/23/20
05/08/20
05/23/20
06/07/20
06/22/20

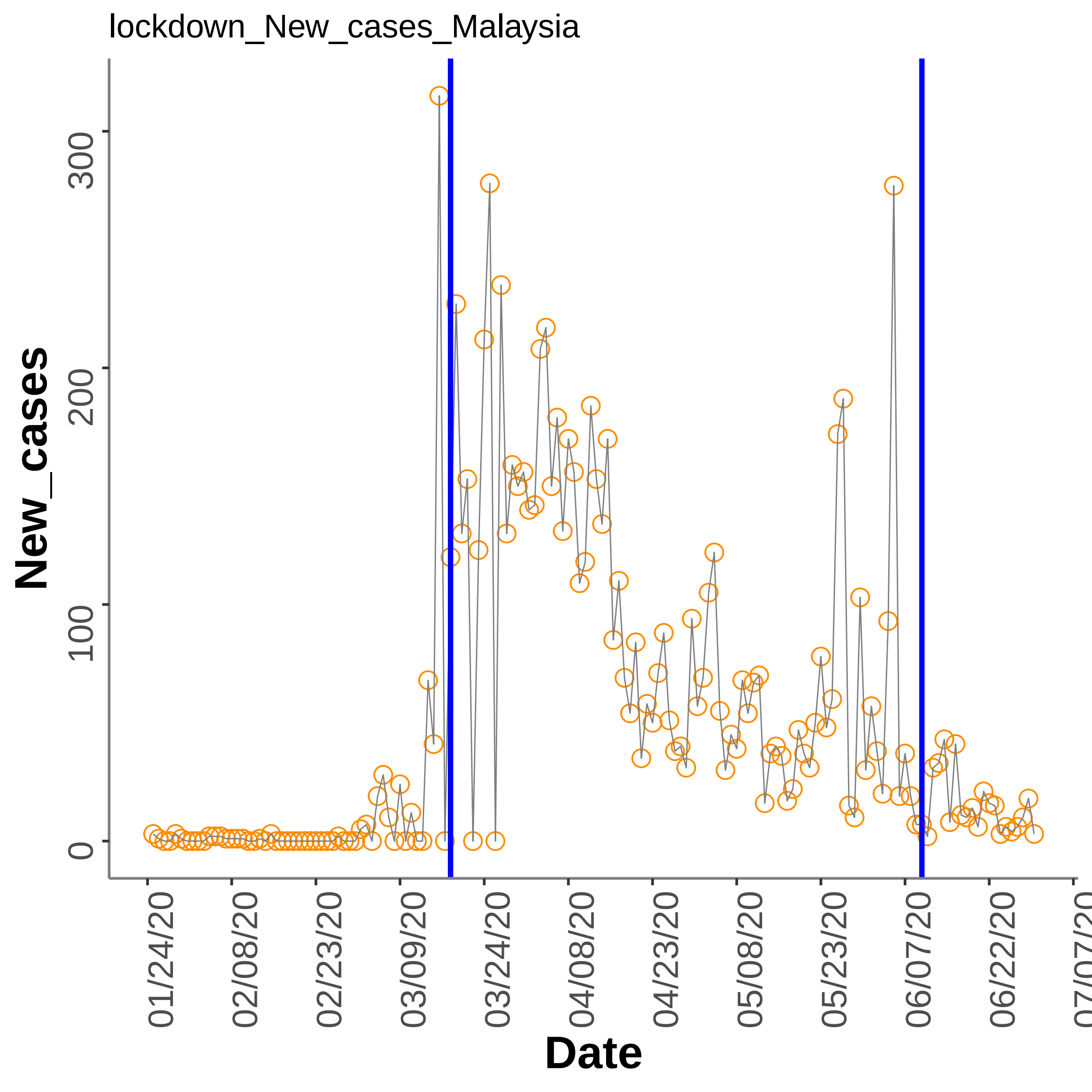
lockdown_New_cases_Malaysia
New_cases
300
200
100
0
01/24/20
02/08/20
02/23/20
03/09/20
03/24/20
04/08/20
04/23/20
05/08/20
05/23/20
06/07/20
06/22/20
07/07/20
Date

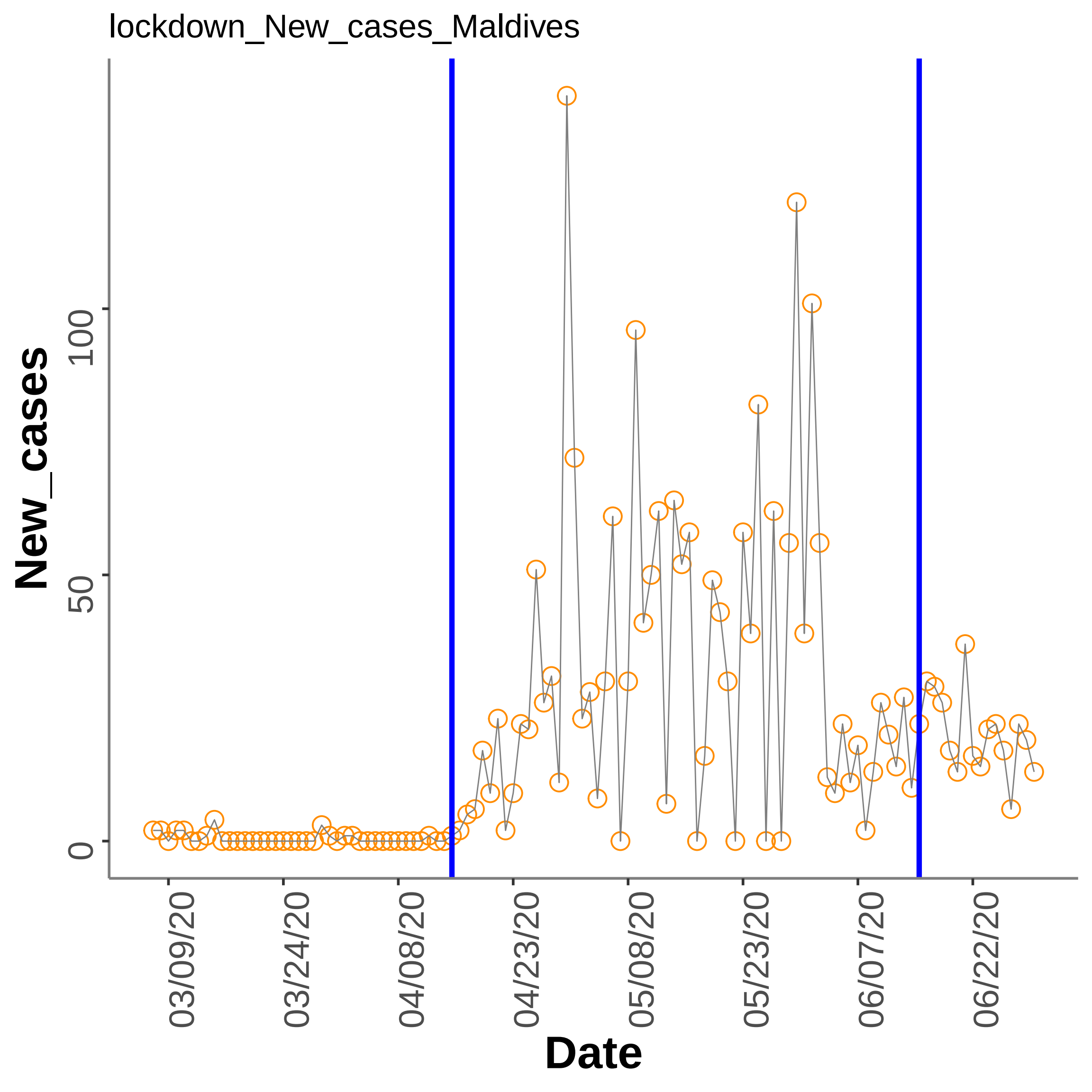

lockdown_New_cases_Maldives
New_cases
0
50
100
Date
03/09/20
03/24/20
04/08/20
04/23/20
05/08/20
05/23/20
06/07/20
06/22/20

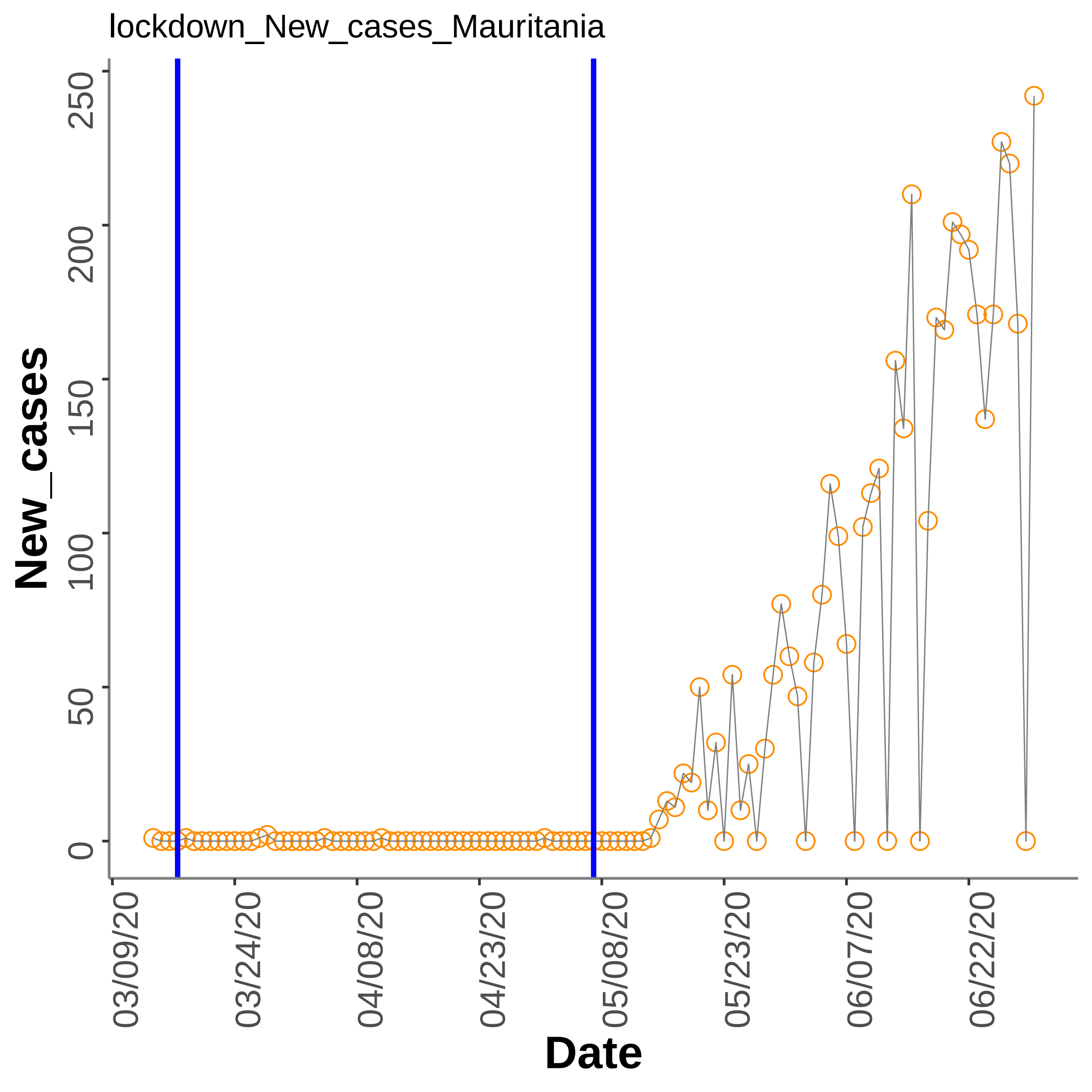

lockdown_New_cases_Mauritania
New_cases
0
50
100
150
200
250
Date
03/09/20
03/24/20
04/08/20
04/23/20
05/08/20
05/23/20
06/07/20
06/22/20

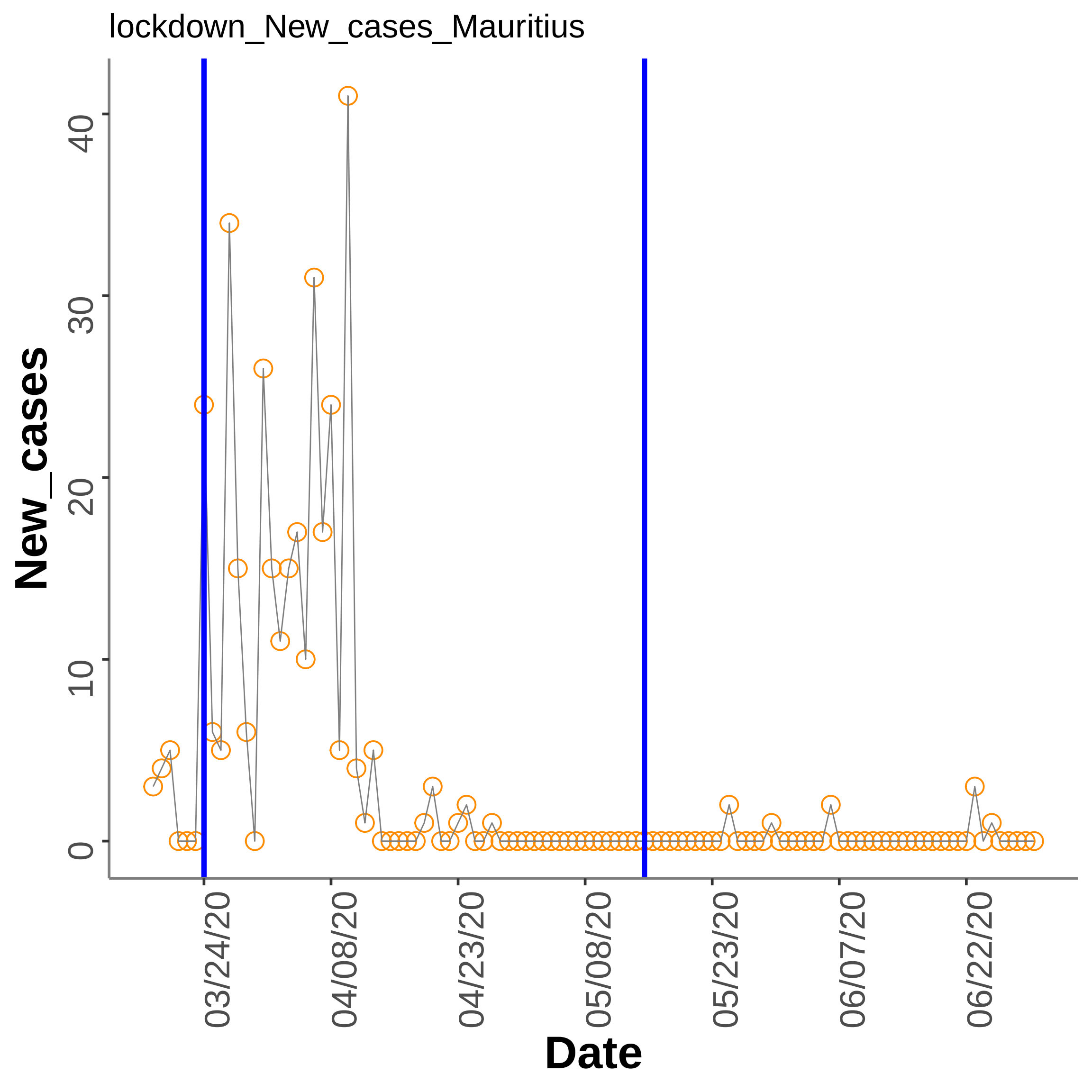
lockdown_New_cases_Mauritius
New_cases
0
10
20
30
40
Date
03/24/20
04/08/20
04/23/20
05/08/20
05/23/20
06/07/20
06/22/20

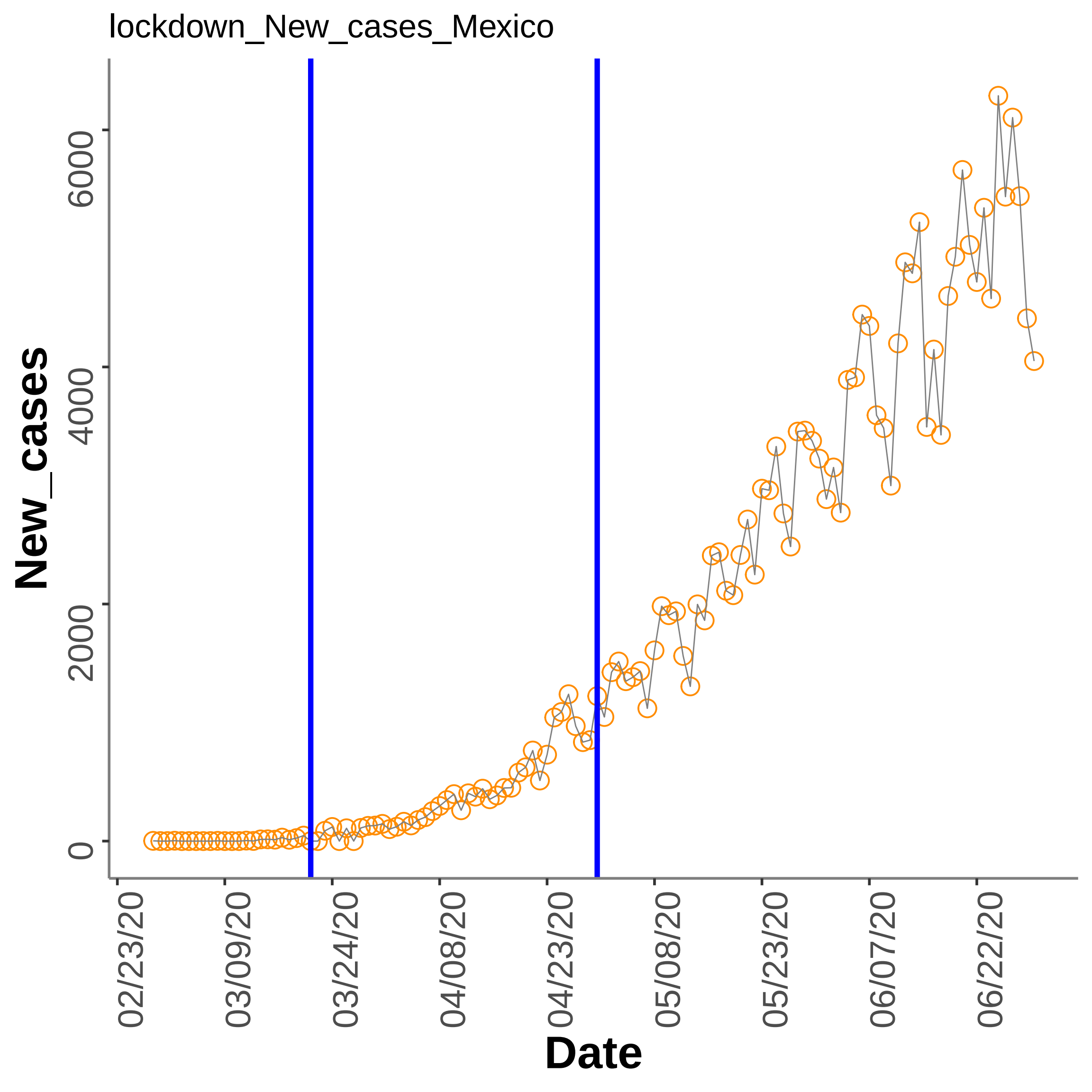

lockdown_New_cases_Mexico
New_cases
0
2000
4000
6000
Date
02/23/20
03/09/20
03/24/20
04/08/20
04/23/20
05/08/20
05/23/20
06/07/20
06/22/20

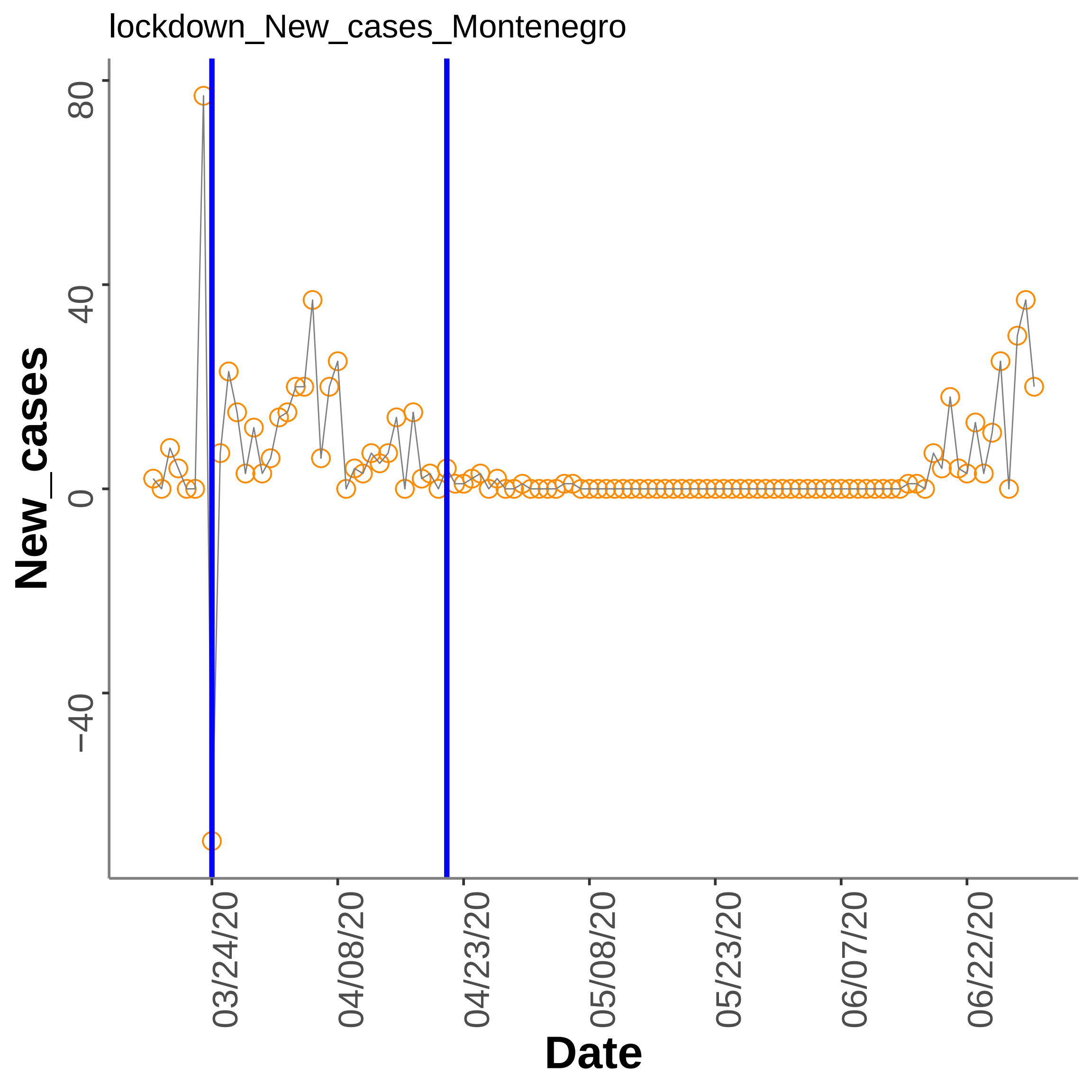
lockdown_New_cases_Montenegro
New_cases
80
40
0
−40
03/24/20
04/08/20
04/23/20
05/08/20
05/23/20
06/07/20
06/22/20
Date

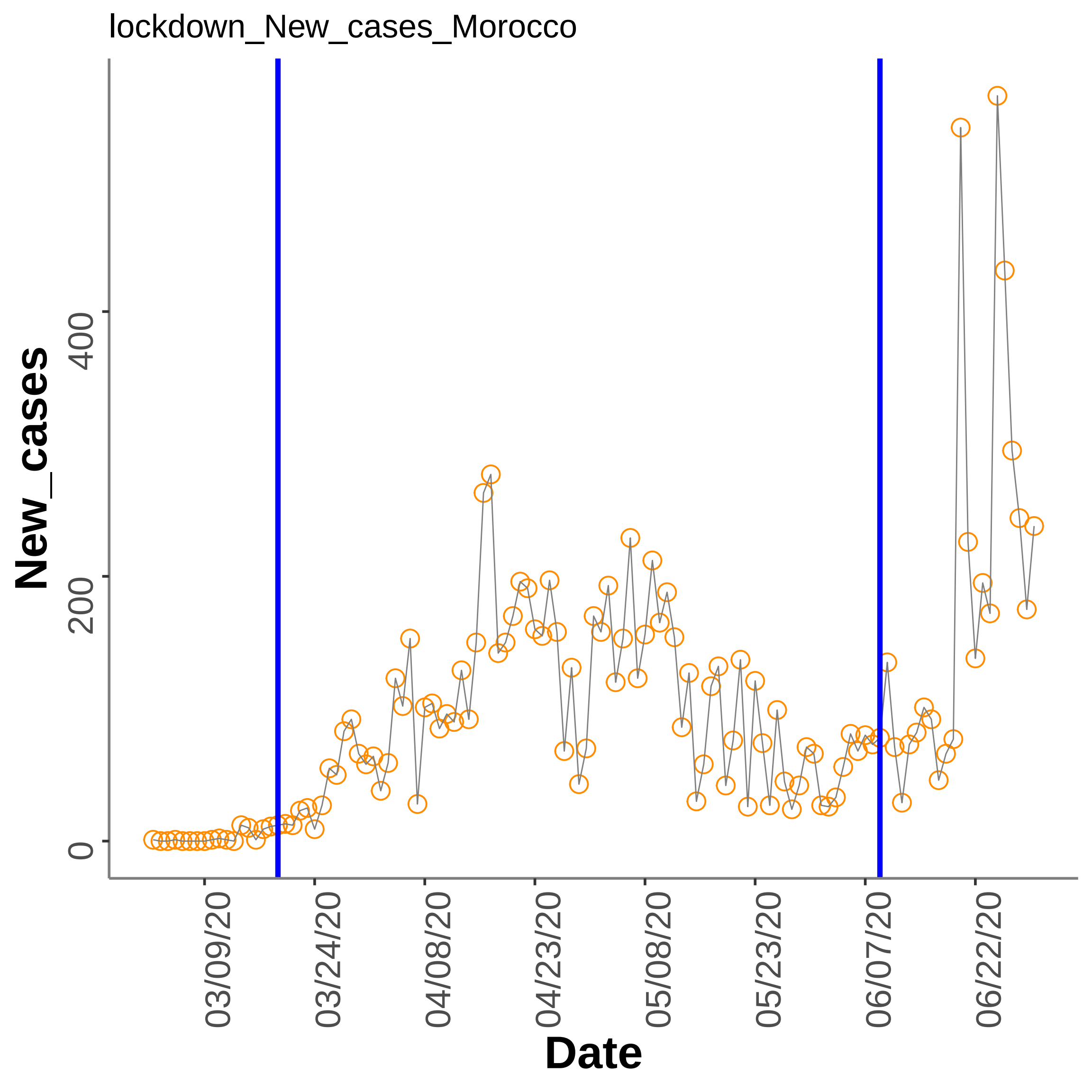

lockdown_New_cases_Morocco
New_cases
400
200
0
03/09/20
03/24/20
04/08/20
04/23/20
05/08/20
05/23/20
06/07/20
06/22/20
Date

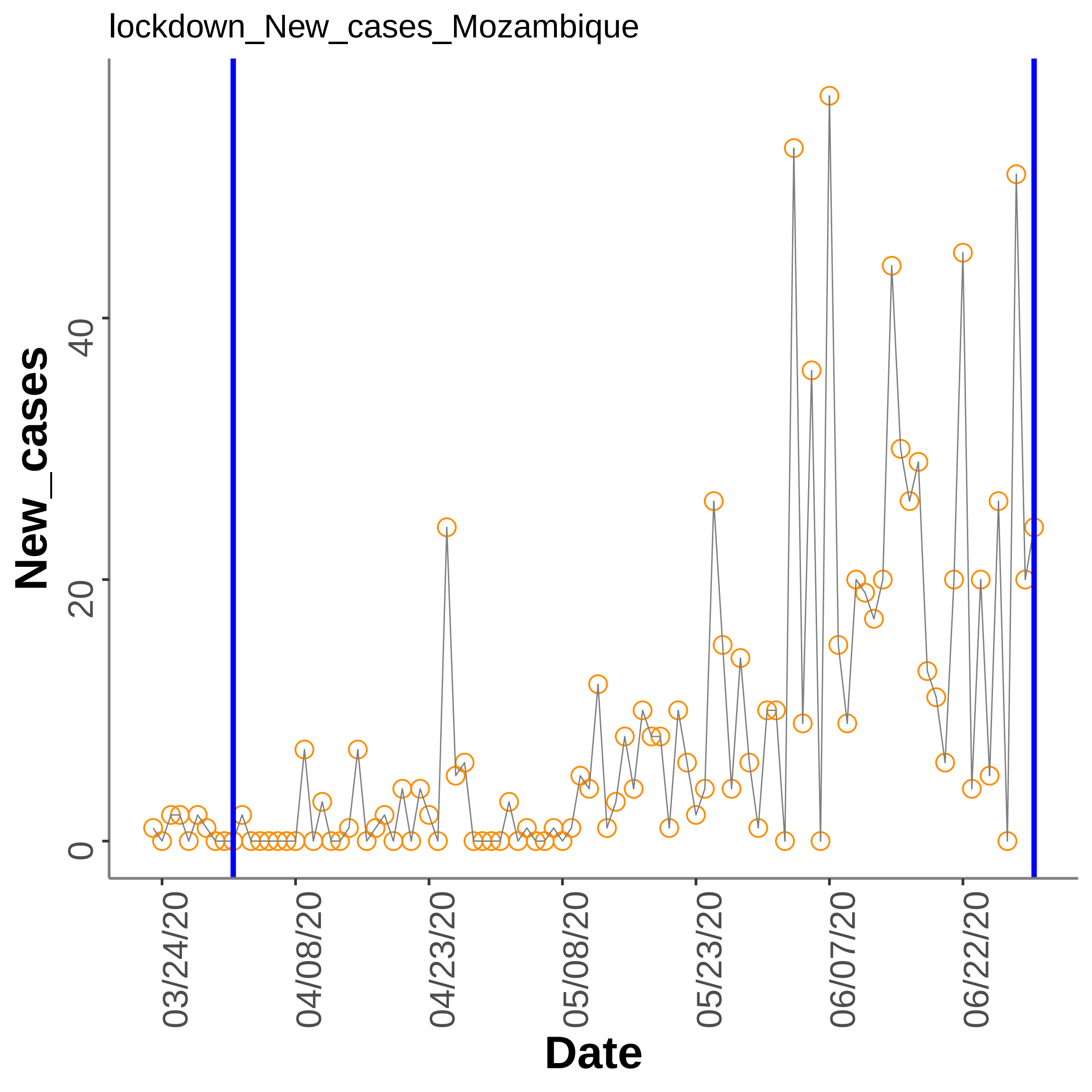
lockdown_New_cases_Mozambique
New_cases
0
20
40
Date
03/24/20
04/08/20
04/23/20
05/08/20
05/23/20
06/07/20
06/22/20

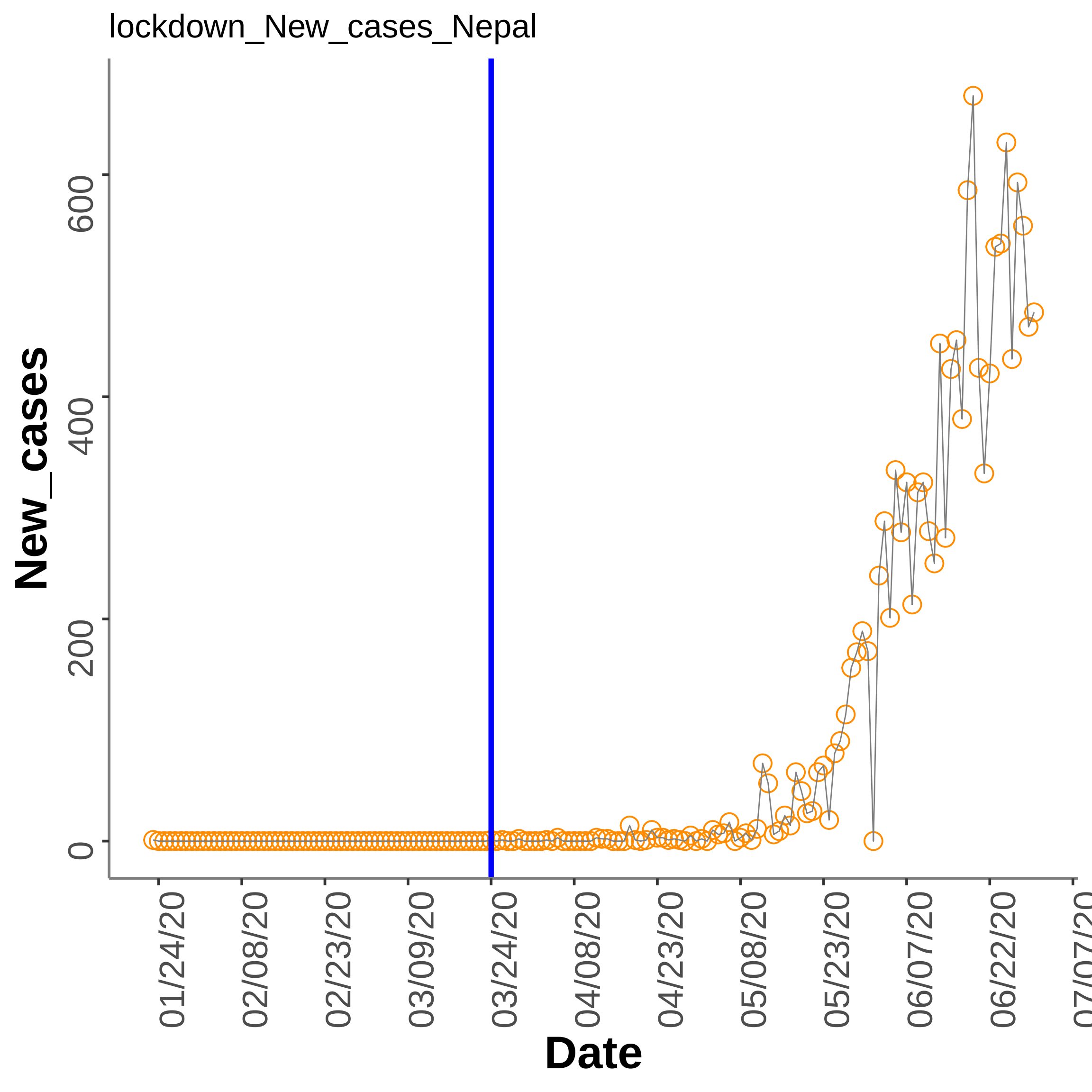

lockdown_New_cases_Nepal
New_cases
0
200
400
600
Date
01/24/20
02/08/20
02/23/20
03/09/20
03/24/20
04/08/20
04/23/20
05/08/20
05/23/20
06/07/20
06/22/20
07/07/20

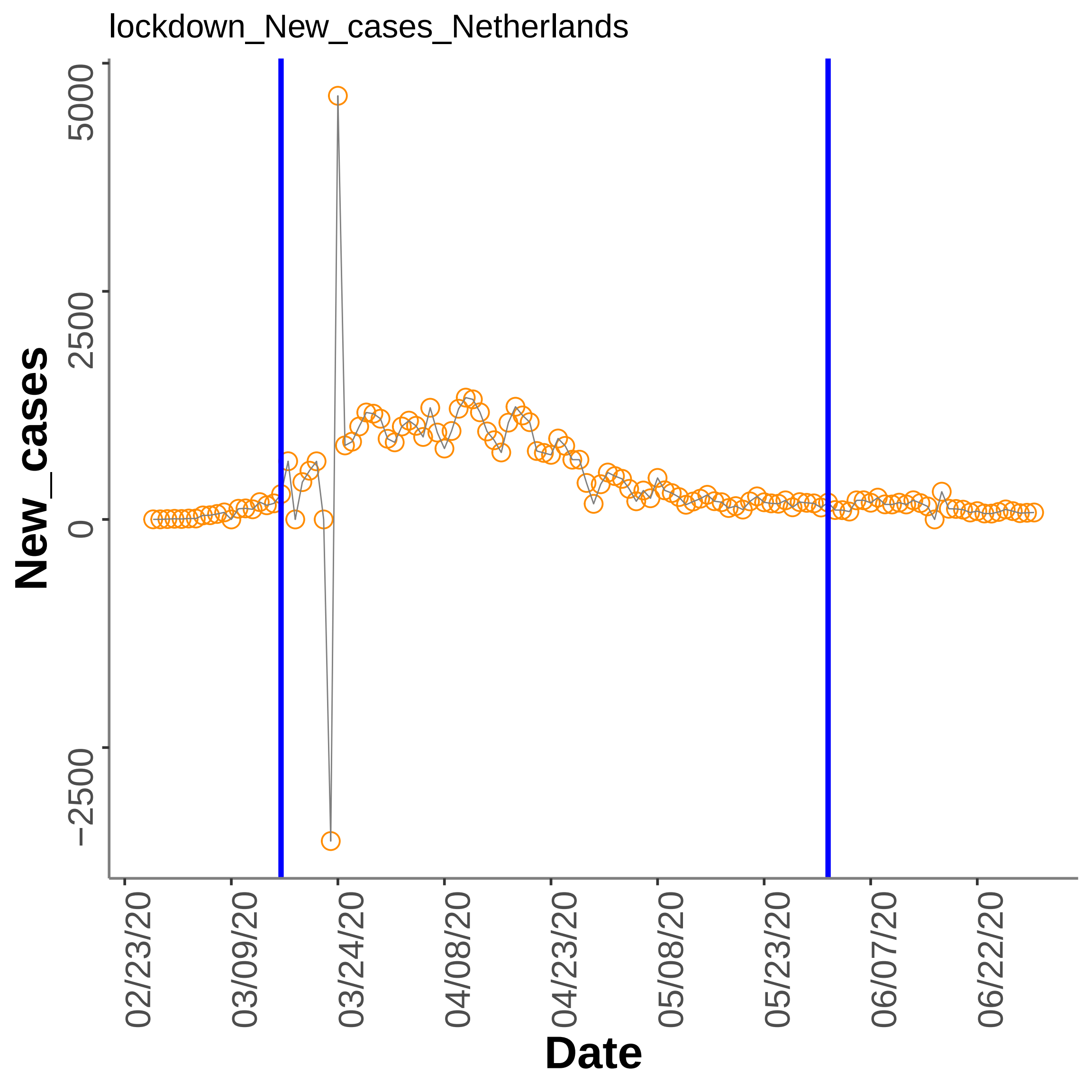
lockdown_New_cases_Netherlands
New_cases
5000
2500
0
−2500
Date
02/23/20
03/09/20
03/24/20
04/08/20
04/23/20
05/08/20
05/23/20
06/07/20
06/22/20

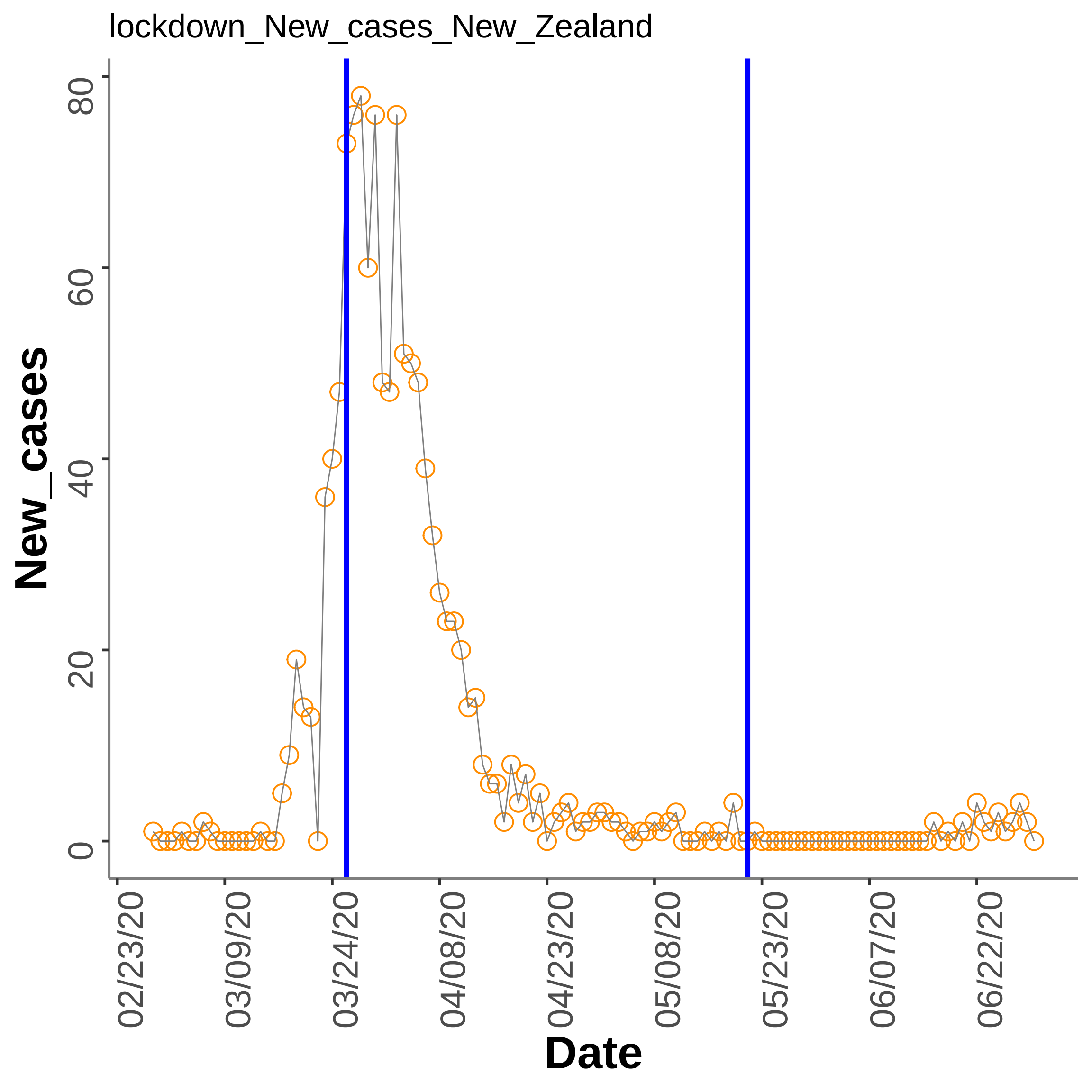

lockdown_New_cases_New_Zealand
New_cases
0
20
40
60
80
Date
02/23/20
03/09/20
03/24/20
04/08/20
04/23/20
05/08/20
05/23/20
06/07/20
06/22/20

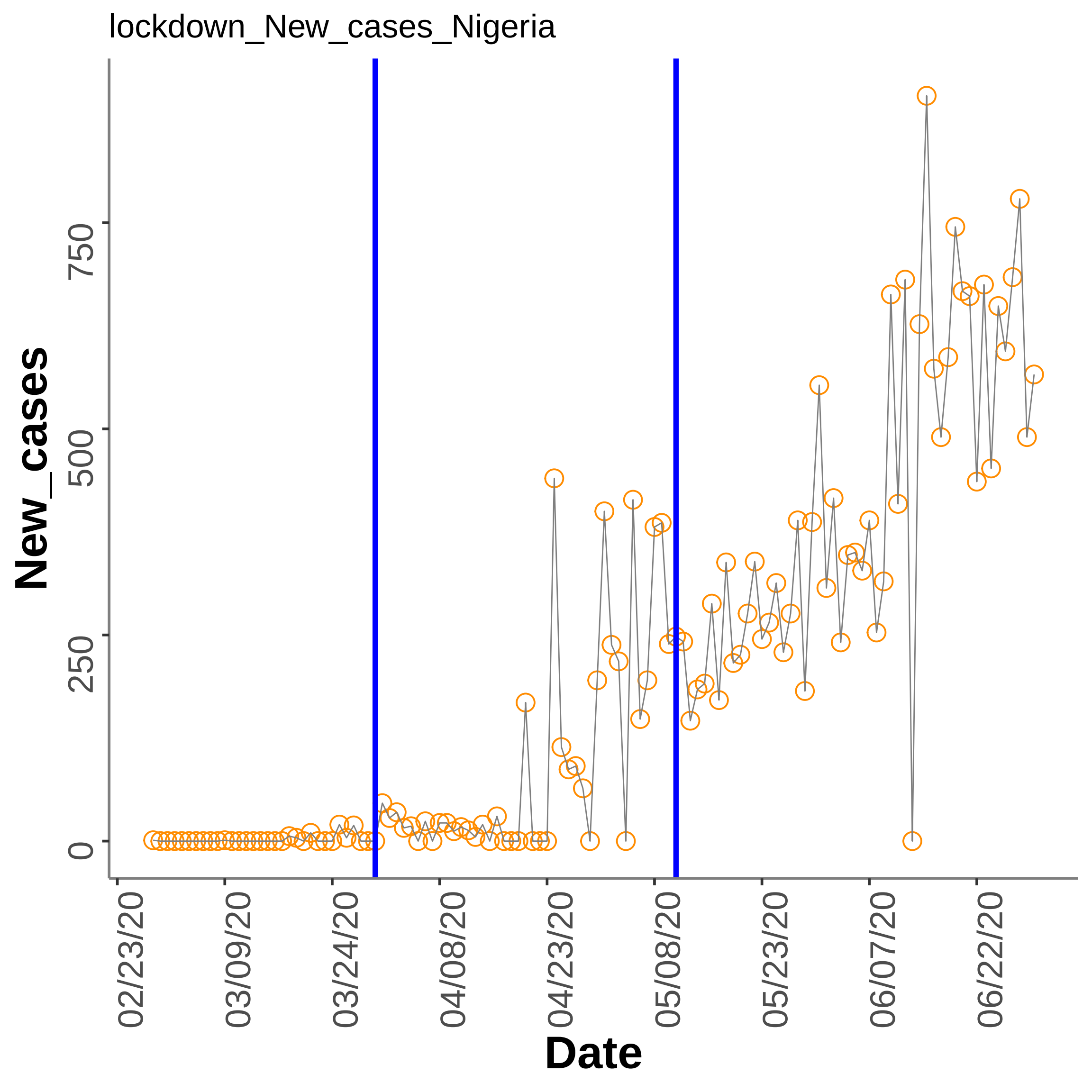

lockdown_New_cases_Nigeria
New_cases
0
250
500
750
02/23/20
03/09/20
03/24/20
04/08/20
04/23/20
05/08/20
05/23/20
06/07/20
06/22/20
Date

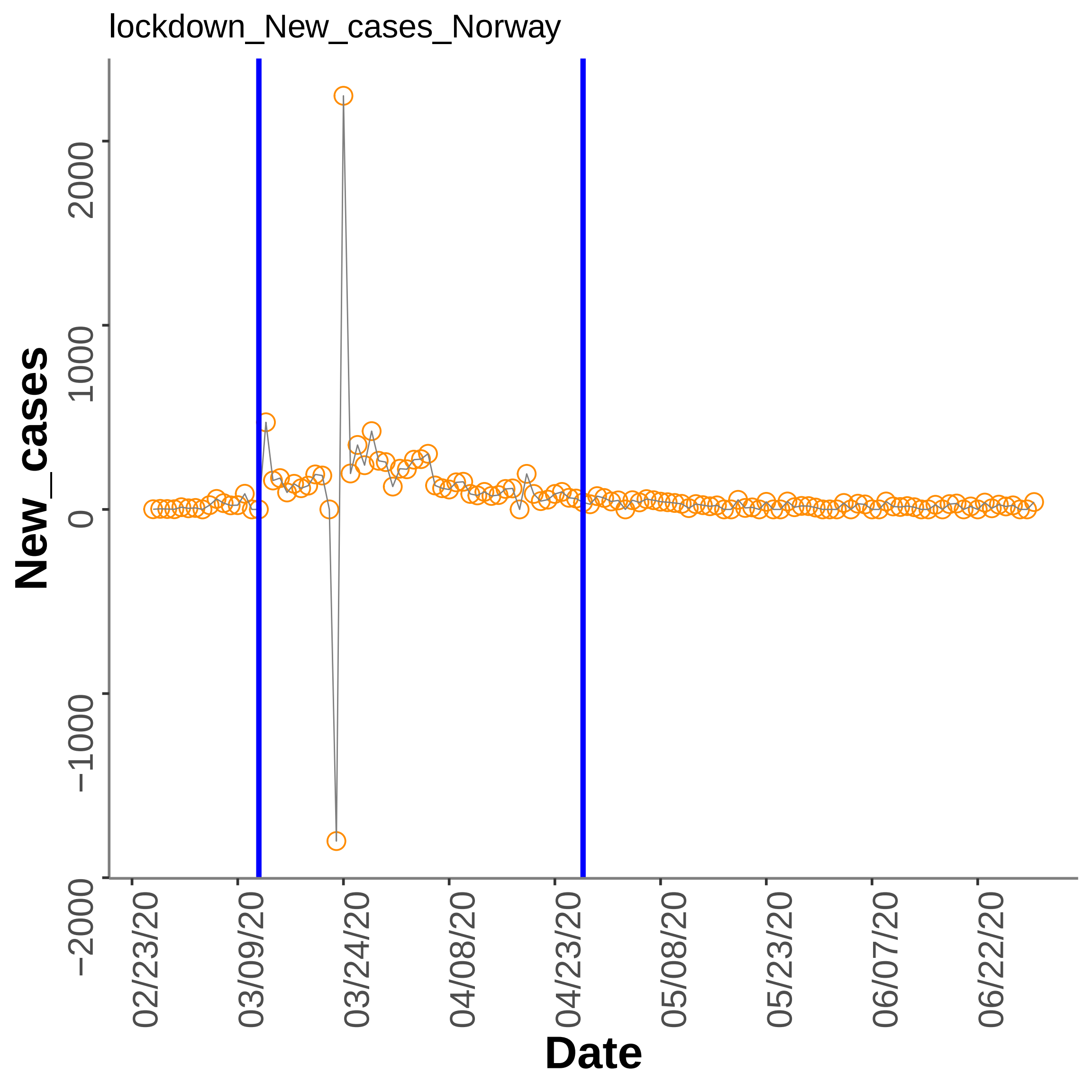
lockdown_New_cases_Norway
New_cases
2000
1000
0
−1000
−2000
02/23/20
03/09/20
03/24/20
04/08/20
04/23/20
05/08/20
05/23/20
06/07/20
06/22/20
Date

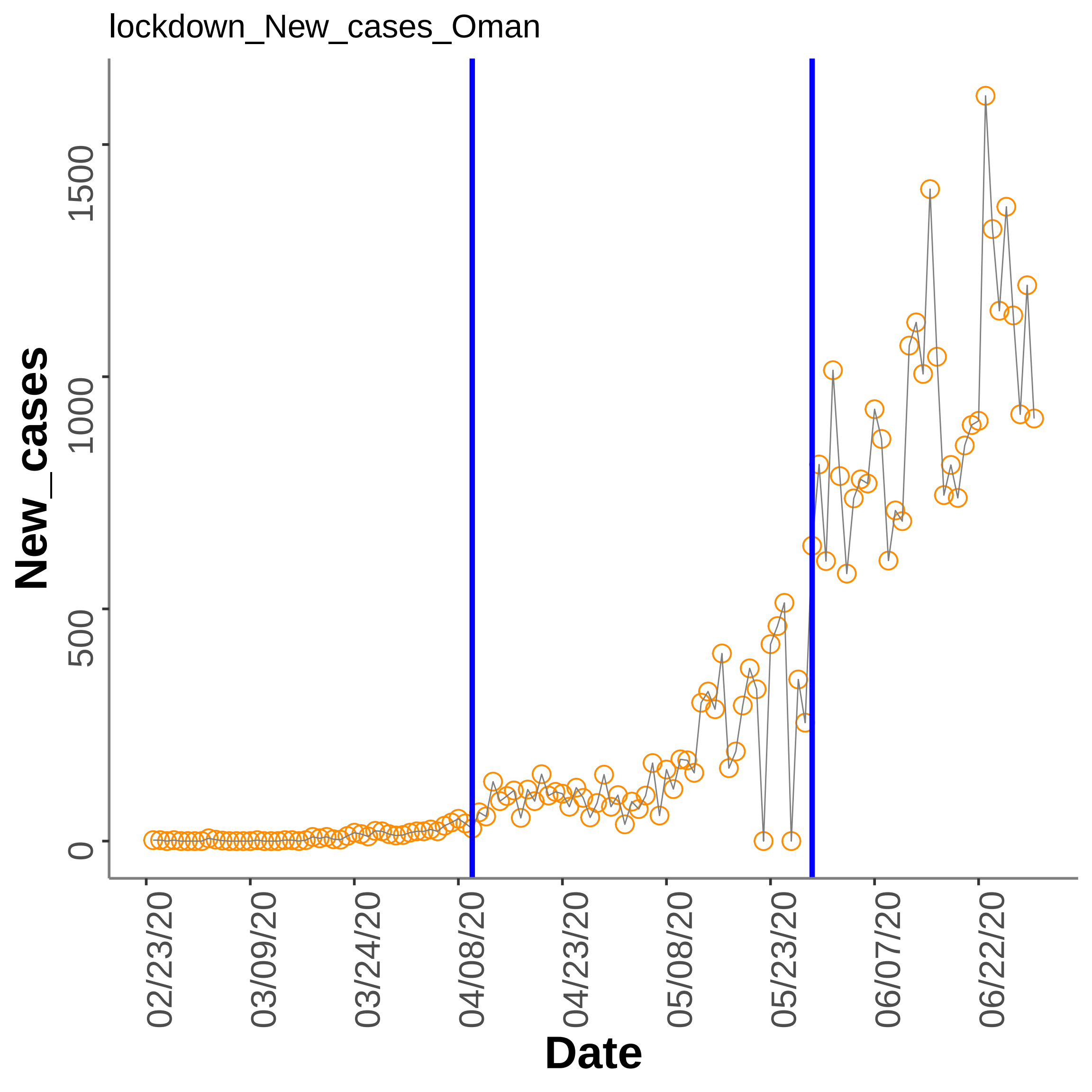
lockdown_New_cases_Oman
New_cases
0
500
1000
1500
02/23/20
03/09/20
03/24/20
04/08/20
04/23/20
05/08/20
05/23/20
06/07/20
06/22/20
Date

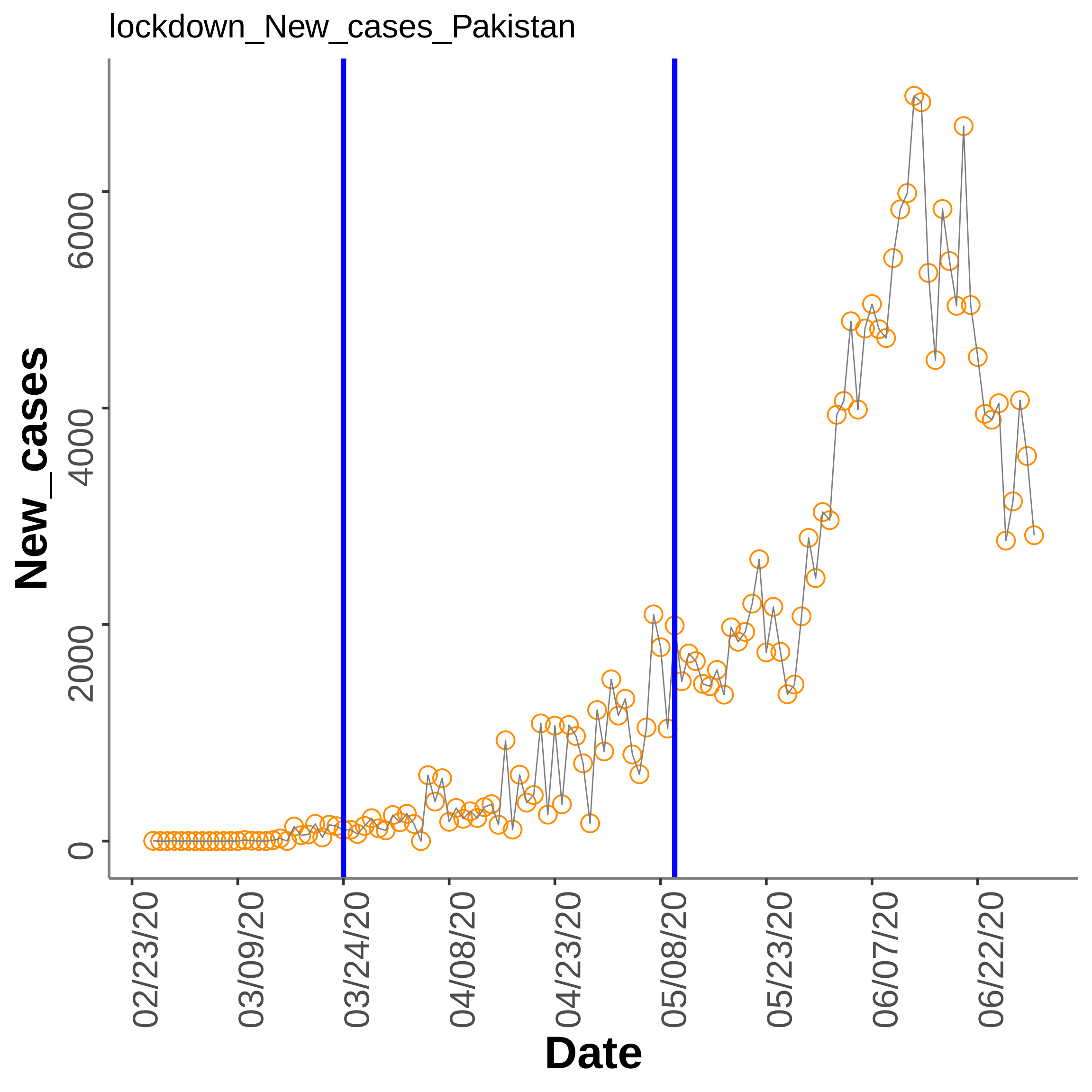
lockdown_New_cases_Pakistan
New_cases
0
2000
4000
6000
02/23/20
03/09/20
03/24/20
04/08/20
04/23/20
05/08/20
05/23/20
06/07/20
06/22/20
Date

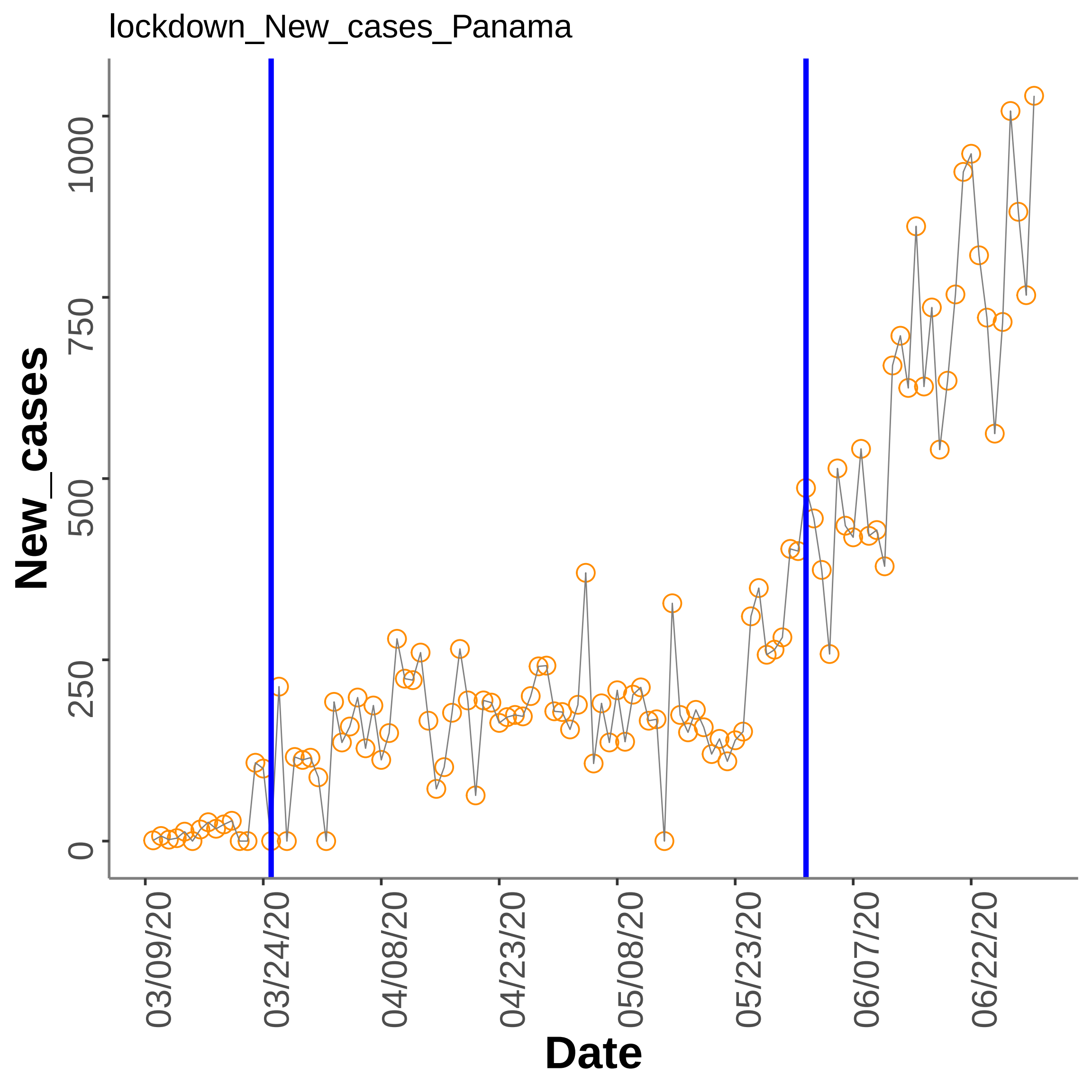

lockdown_New_cases_Panama
New_cases
0
250
500
750
1000
Date
03/09/20
03/24/20
04/08/20
04/23/20
05/08/20
05/23/20
06/07/20
06/22/20

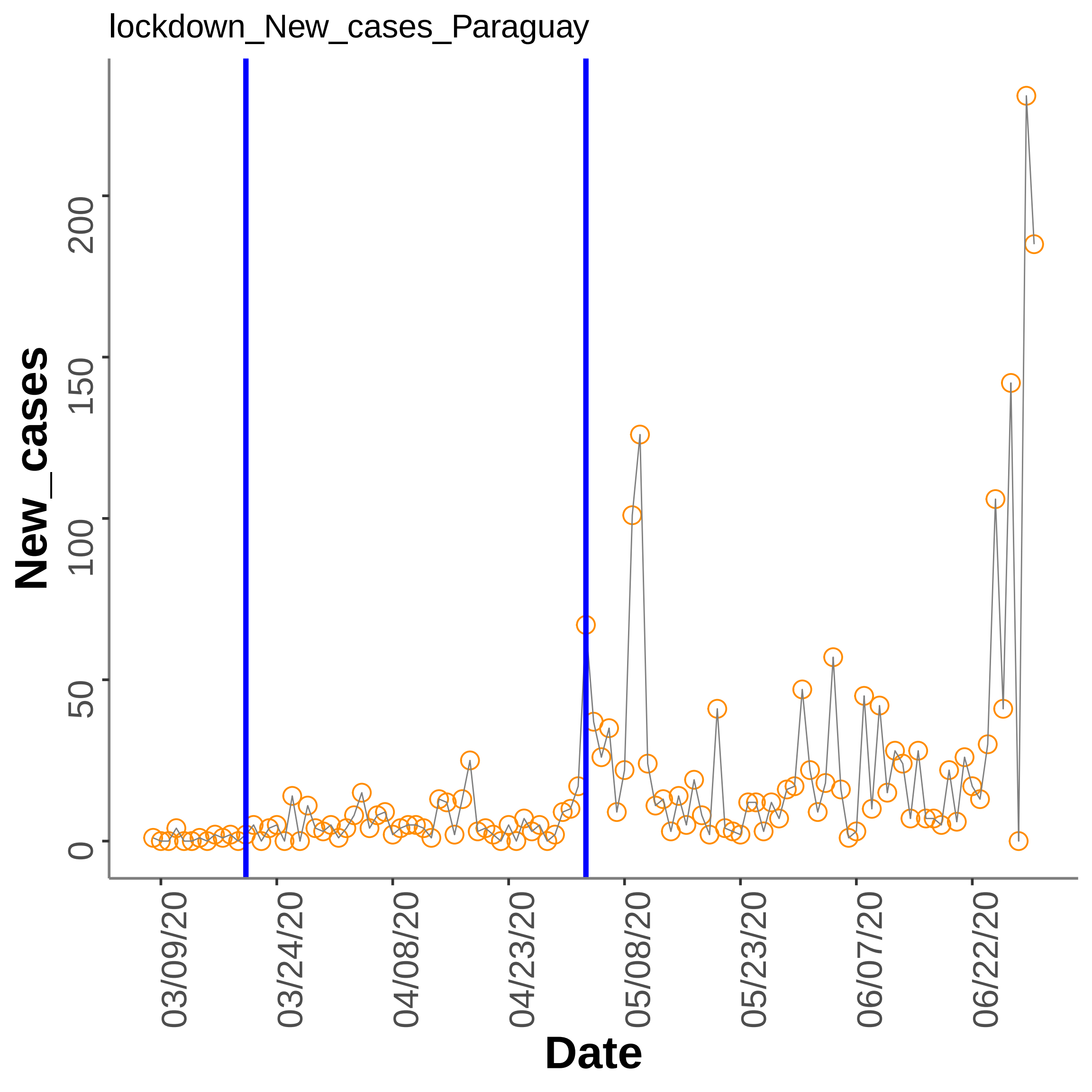
lockdown_New_cases_Paraguay
New_cases
0
50
100
150
200
03/09/20
03/24/20
04/08/20
04/23/20
05/08/20
05/23/20
06/07/20
06/22/20
Date

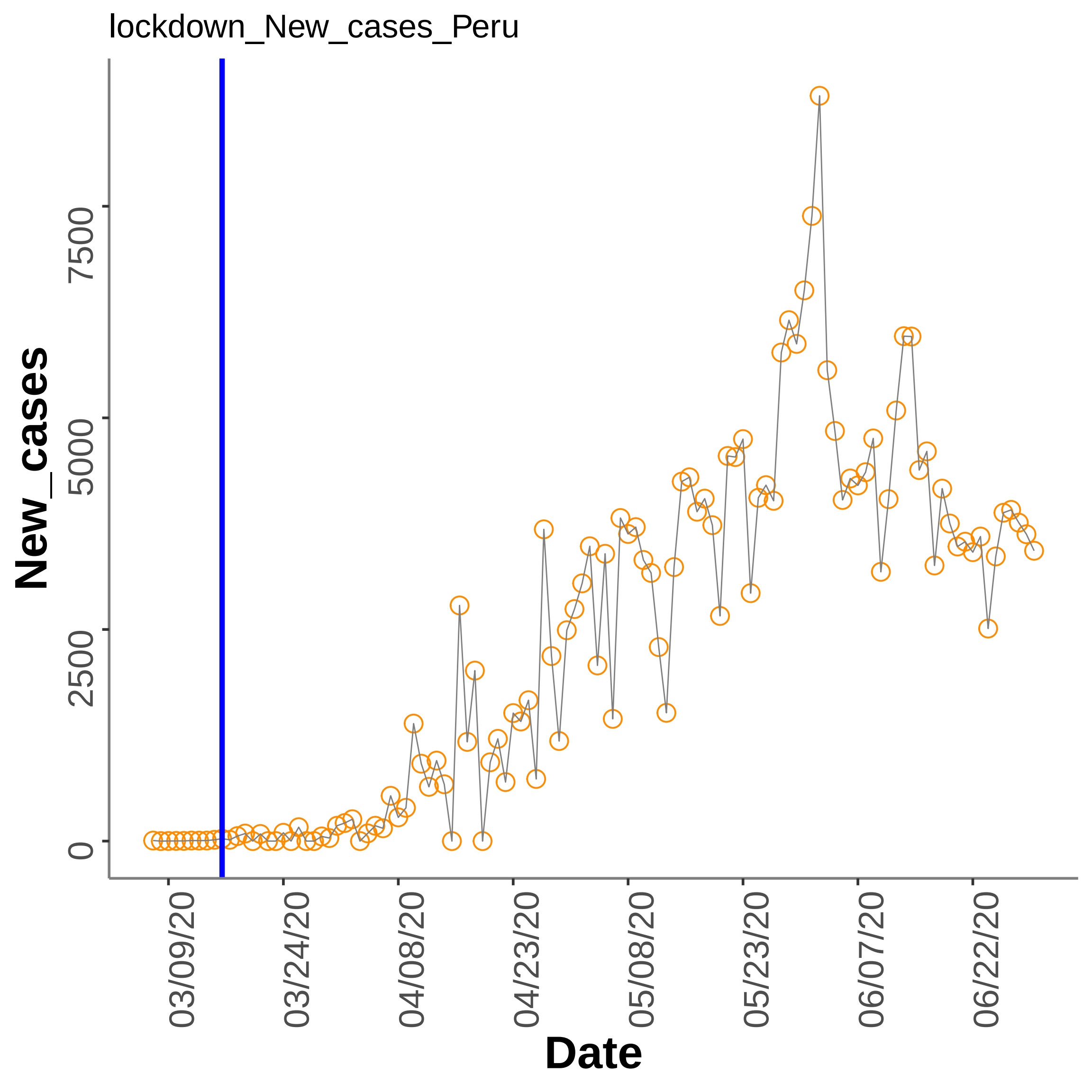
lockdown_New_cases_Peru
New_cases
0
2500
5000
7500
Date
03/09/20
03/24/20
04/08/20
04/23/20
05/08/20
05/23/20
06/07/20
06/22/20

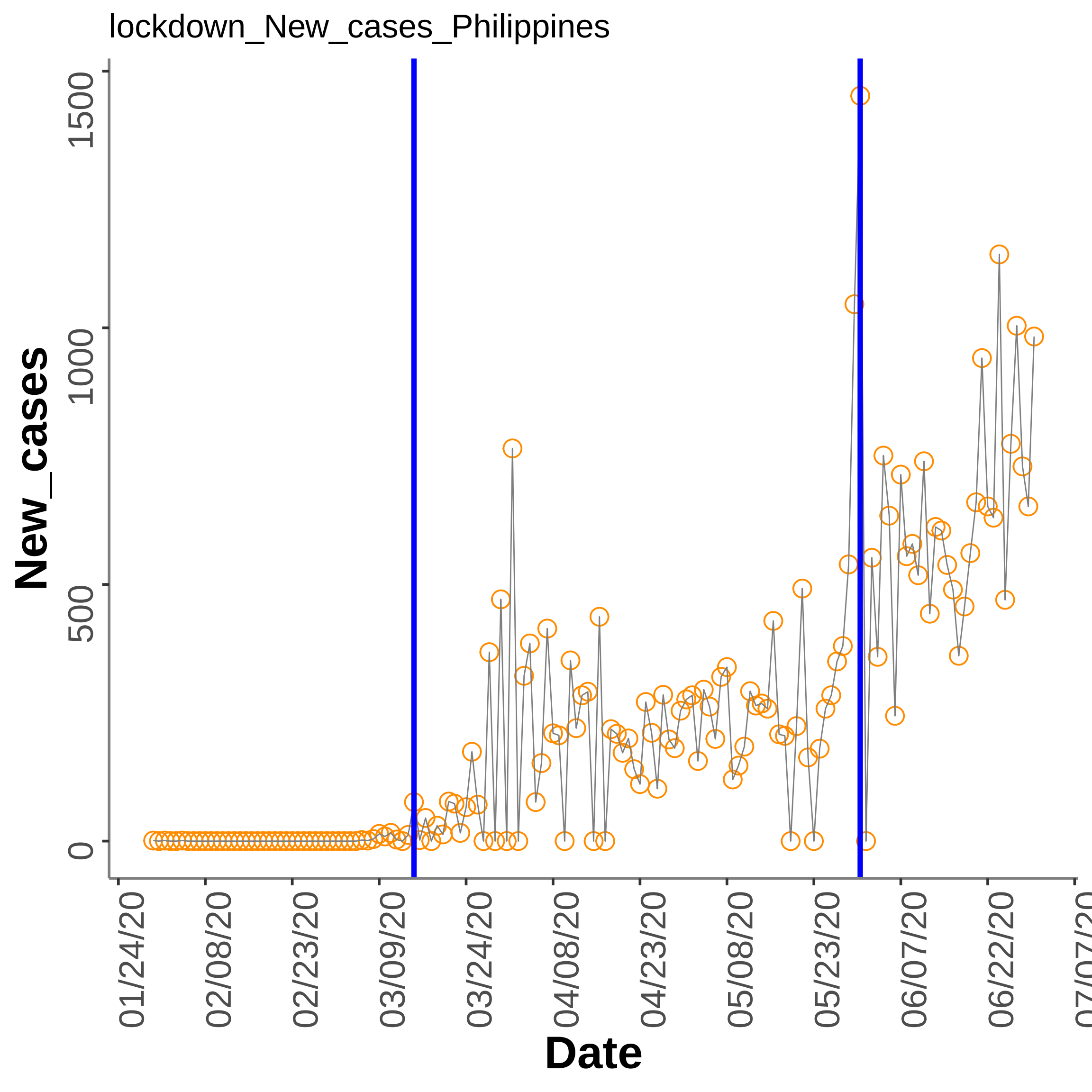

lockdown_New_cases_Philippines
New_cases
0
500
1000
1500
Date
01/24/20
02/08/20
02/23/20
03/09/20
03/24/20
04/08/20
04/23/20
05/08/20
05/23/20
06/07/20
06/22/20
07/07/20

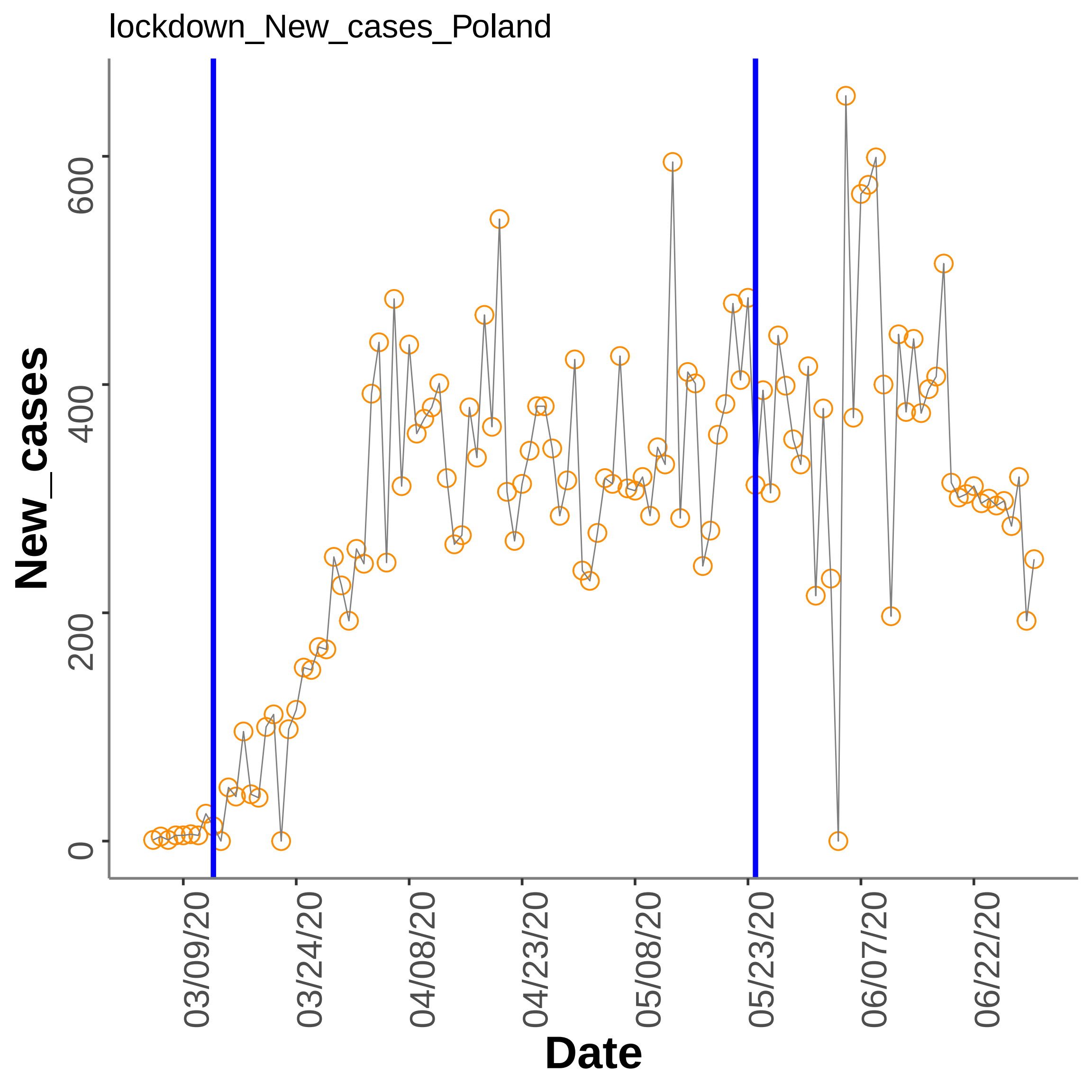
lockdown_New_cases_Poland
New_cases
0
200
400
600
03/09/20
03/24/20
04/08/20
04/23/20
05/08/20
05/23/20
06/07/20
06/22/20
Date

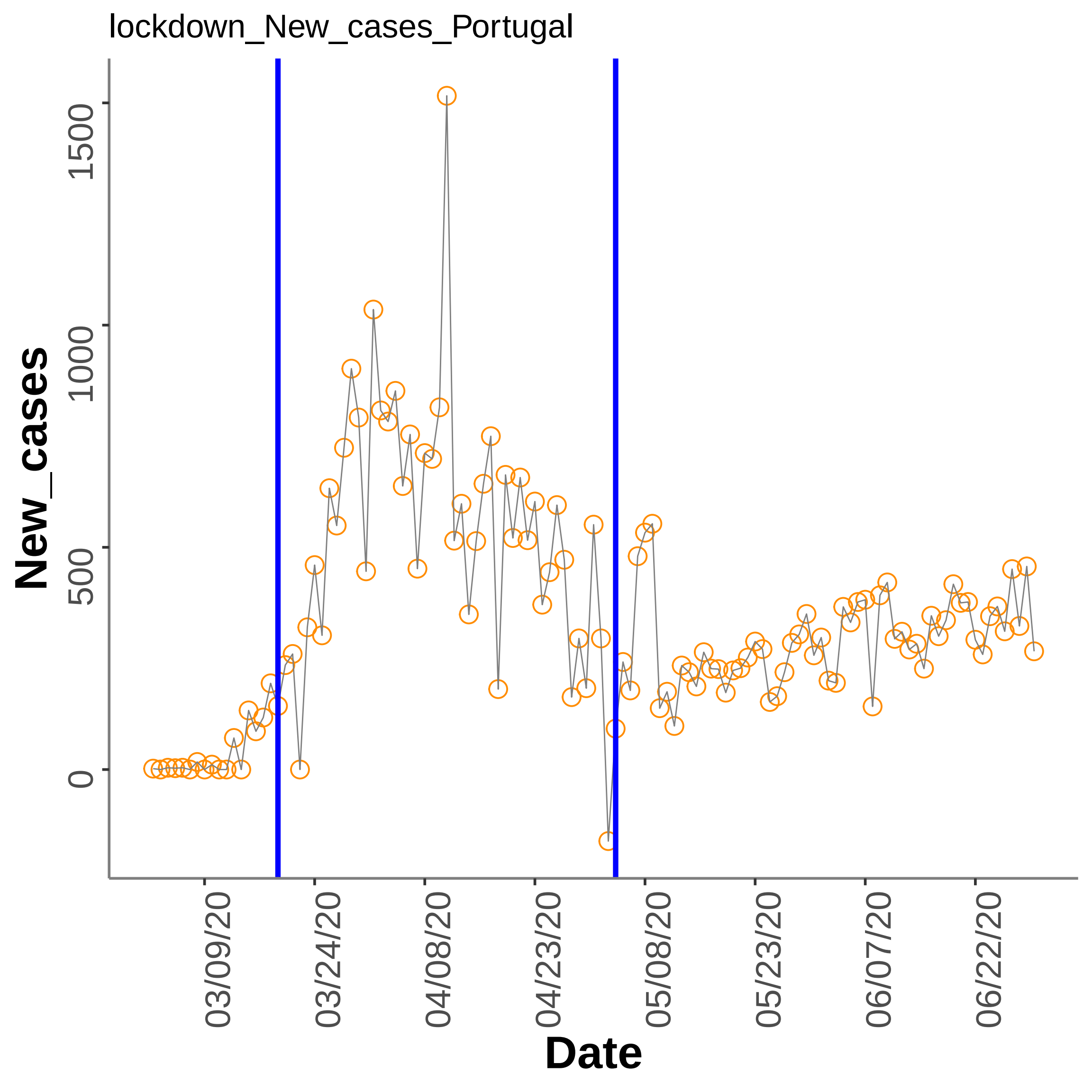
lockdown_New_cases_Portugal
New_cases
Date
0
500
1000
1500
03/09/20
03/24/20
04/08/20
04/23/20
05/08/20
05/23/20
06/07/20
06/22/20

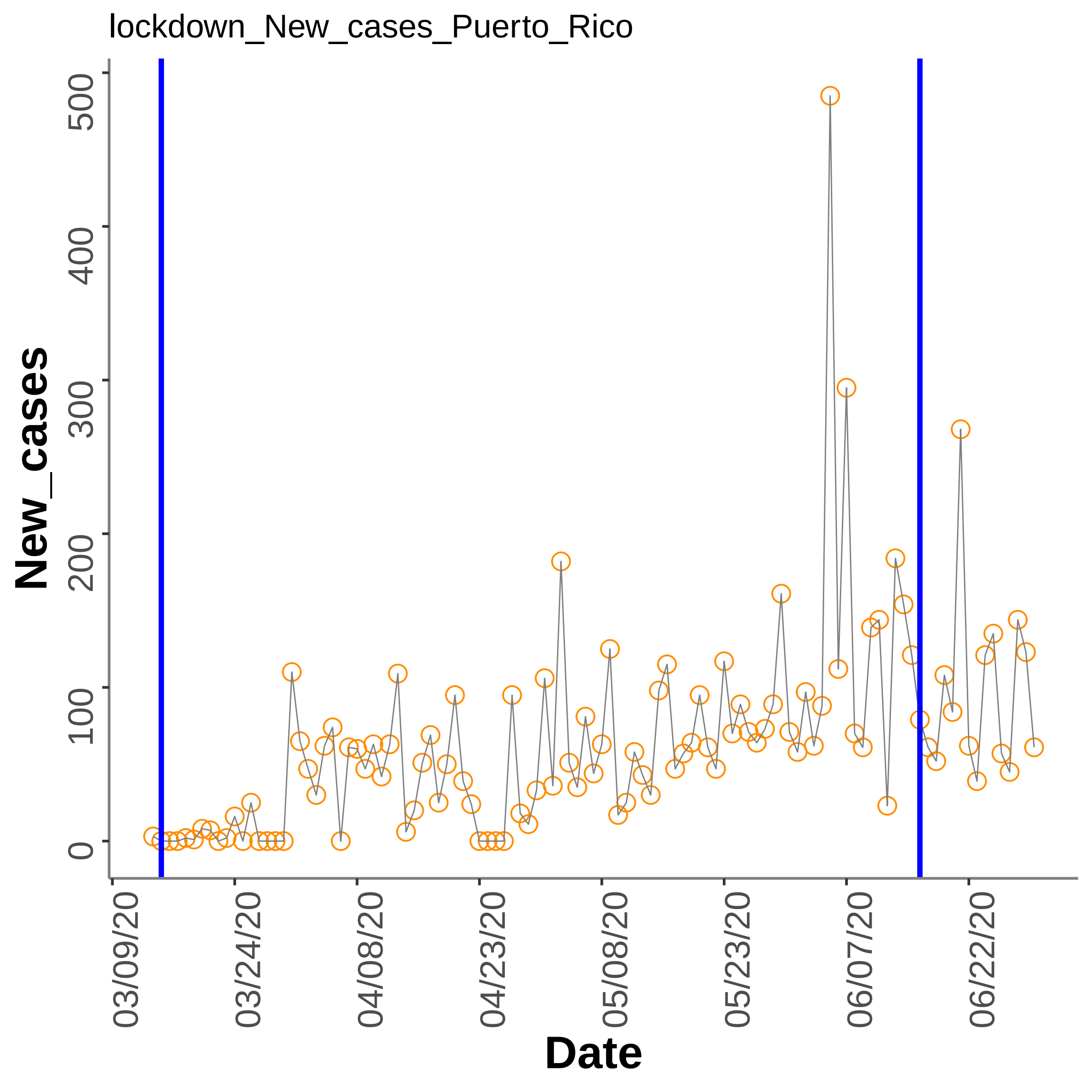
lockdown_New_cases_Puerto_Rico
New_cases
0
100
200
300
400
500
03/09/20
03/24/20
04/08/20
04/23/20
05/08/20
05/23/20
06/07/20
06/22/20
Date

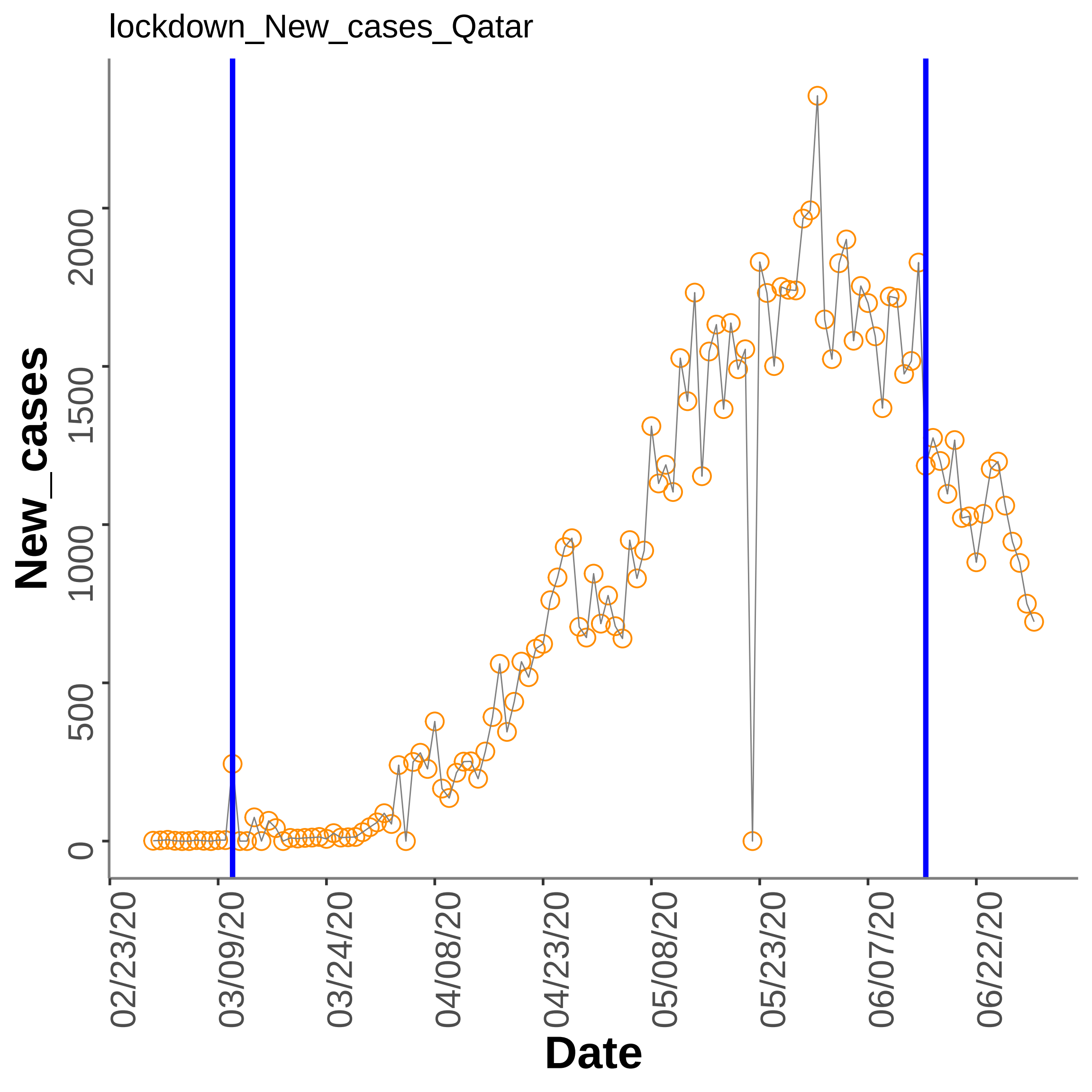

lockdown_New_cases_Qatar
New_cases
0
500
1000
1500
2000
Date
02/23/20
03/09/20
03/24/20
04/08/20
04/23/20
05/08/20
05/23/20
06/07/20
06/22/20

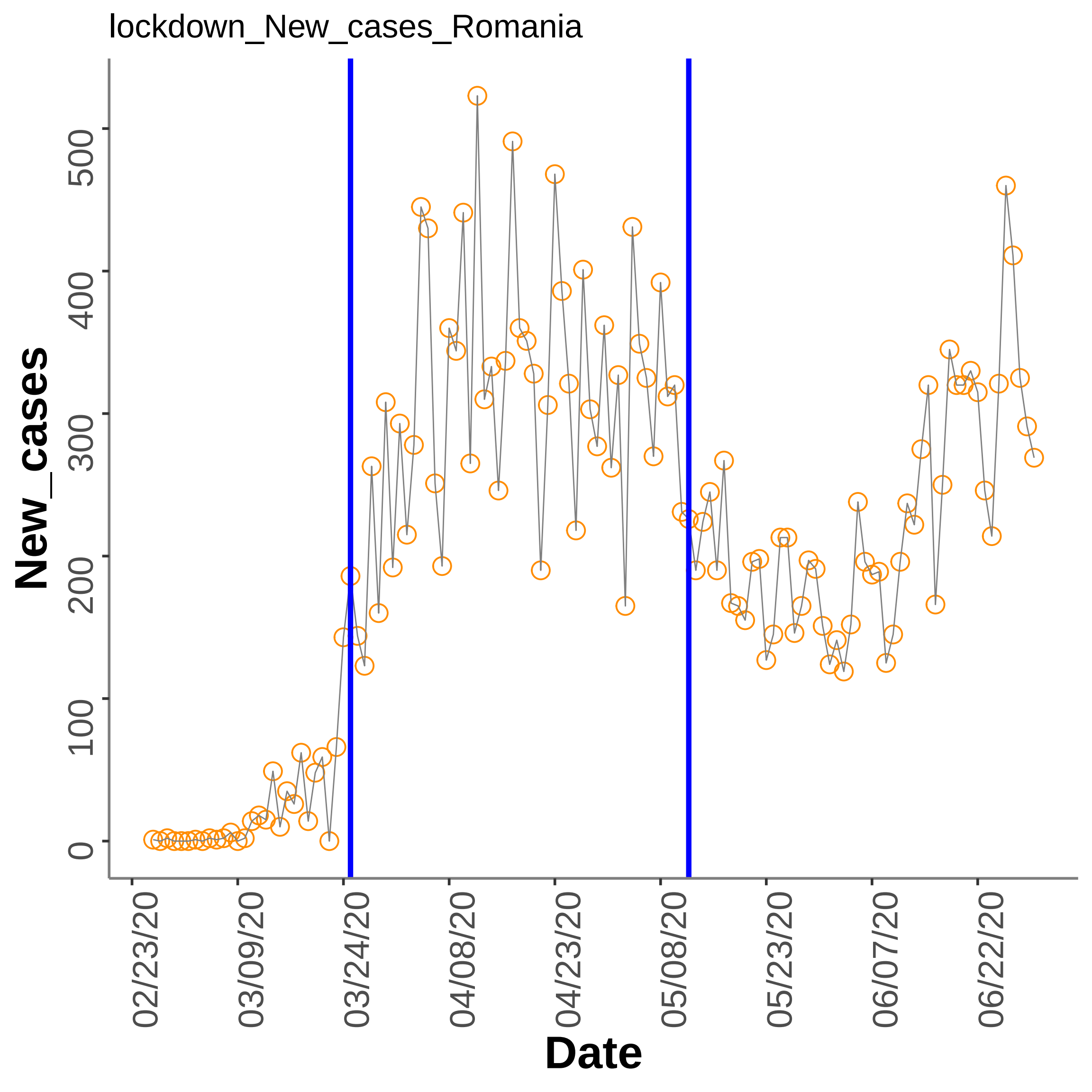
lockdown_New_cases_Romania
New_cases
0
100
200
300
400
500
Date
02/23/20
03/09/20
03/24/20
04/08/20
04/23/20
05/08/20
05/23/20
06/07/20
06/22/20

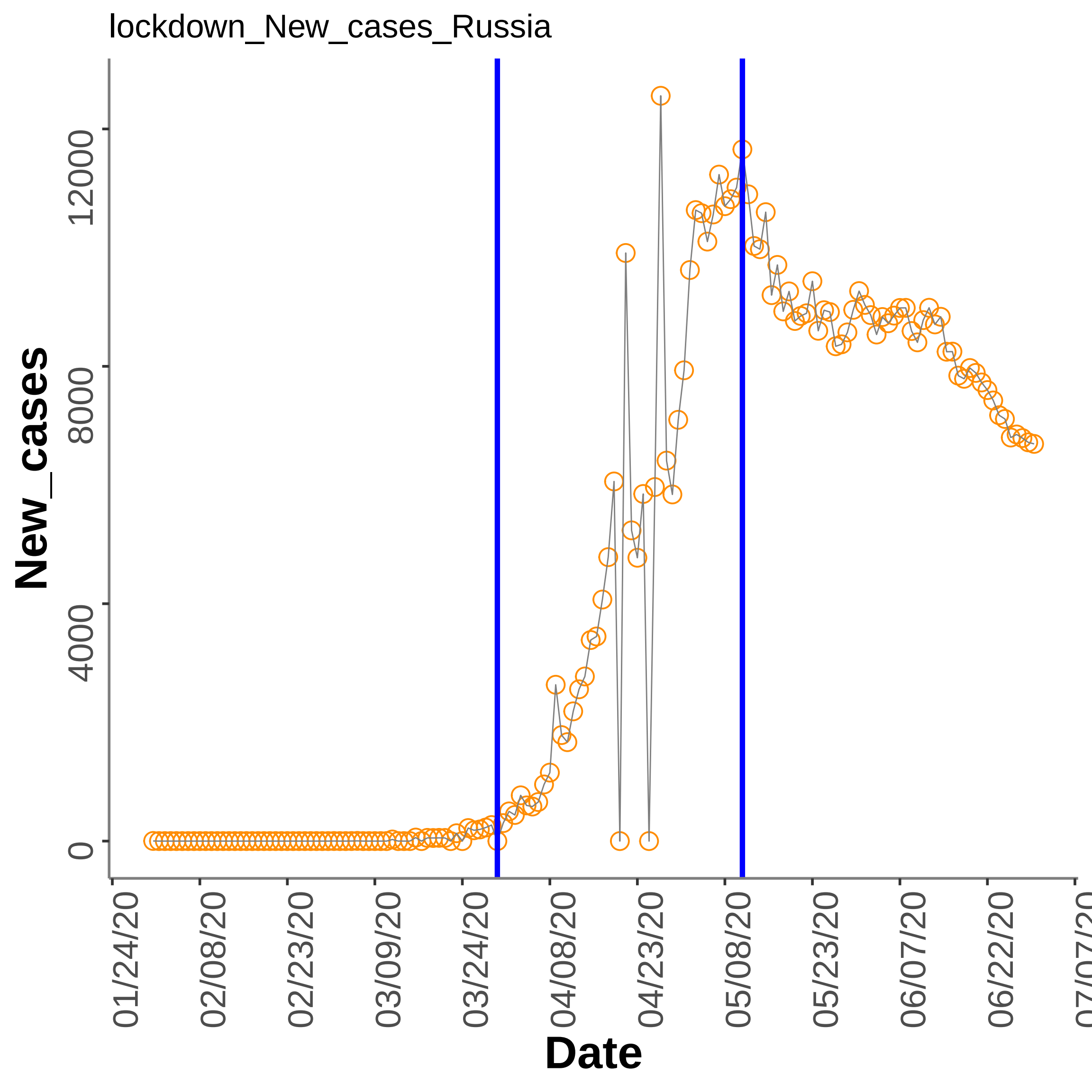
lockdown_New_cases_Russia
New_cases
0
4000
8000
12000
Date
01/24/20
02/08/20
02/23/20
03/09/20
03/24/20
04/08/20
04/23/20
05/08/20
05/23/20
06/07/20
06/22/20

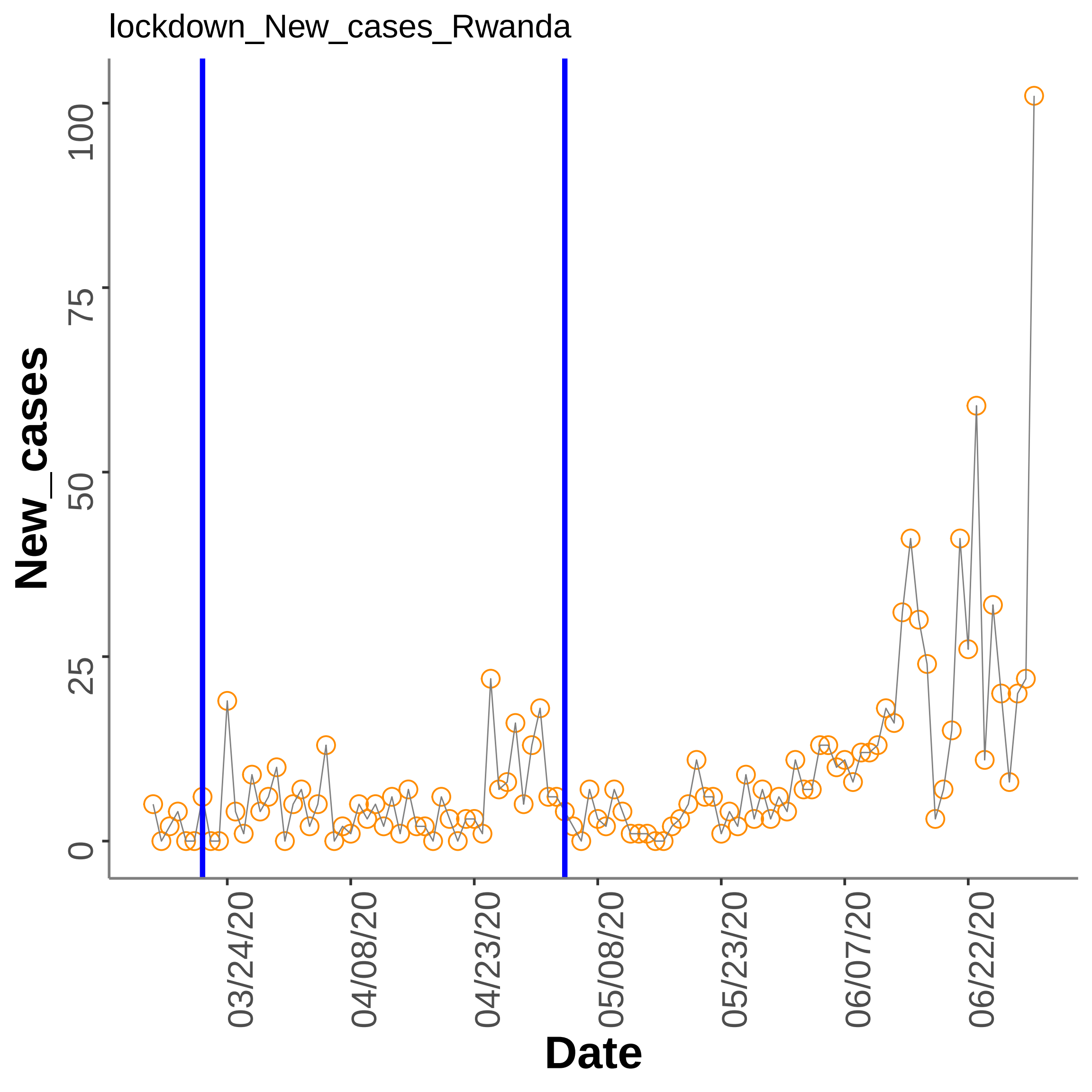
lockdown_New_cases_Rwanda
New_cases
100
75
50
25
0
03/24/20
04/08/20
04/23/20
05/08/20
05/23/20
06/07/20
06/22/20
Date

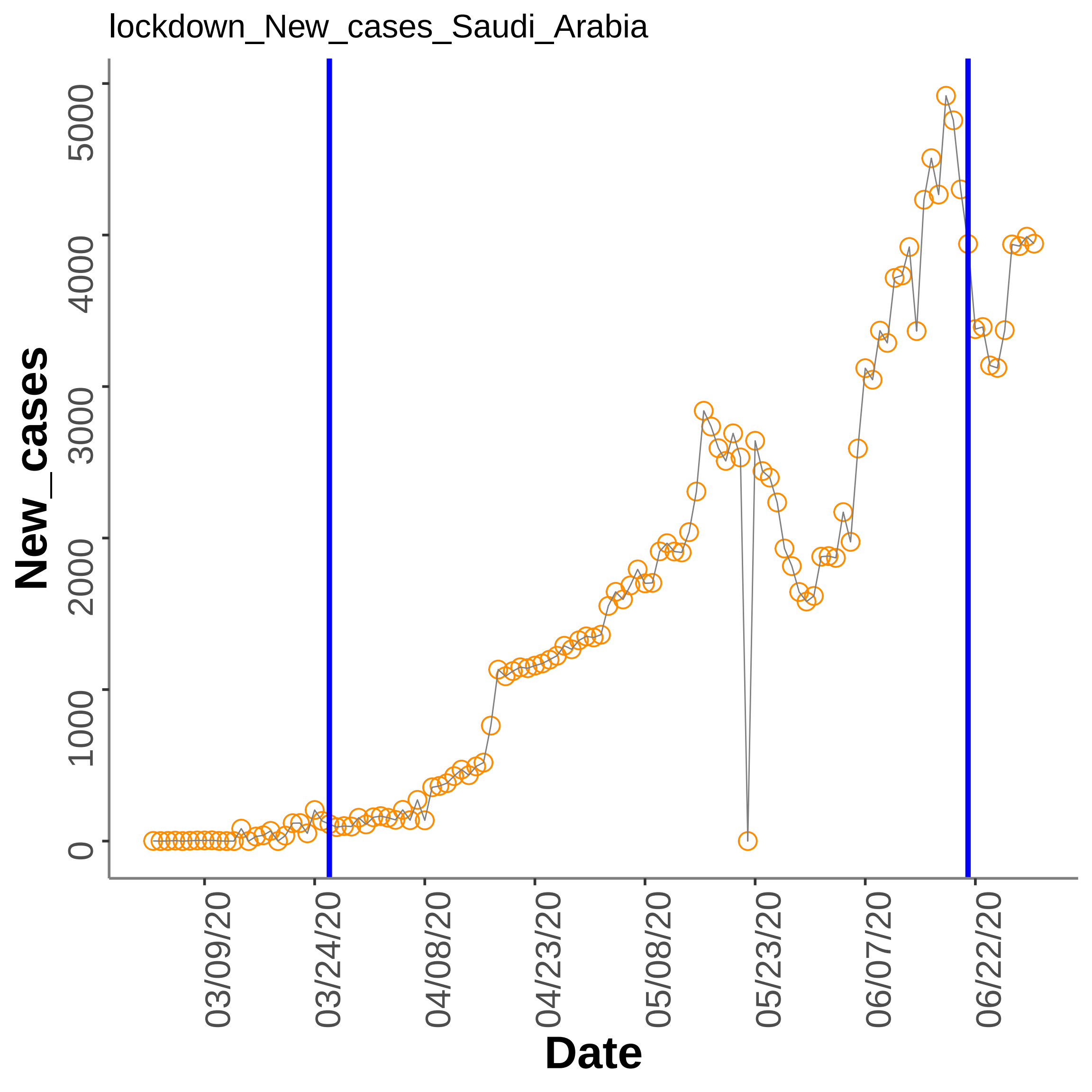

lockdown_New_cases_Saudi_Arabia
New_cases
0
1000
2000
3000
4000
5000
Date
03/09/20
03/24/20
04/08/20
04/23/20
05/08/20
05/23/20
06/07/20
06/22/20

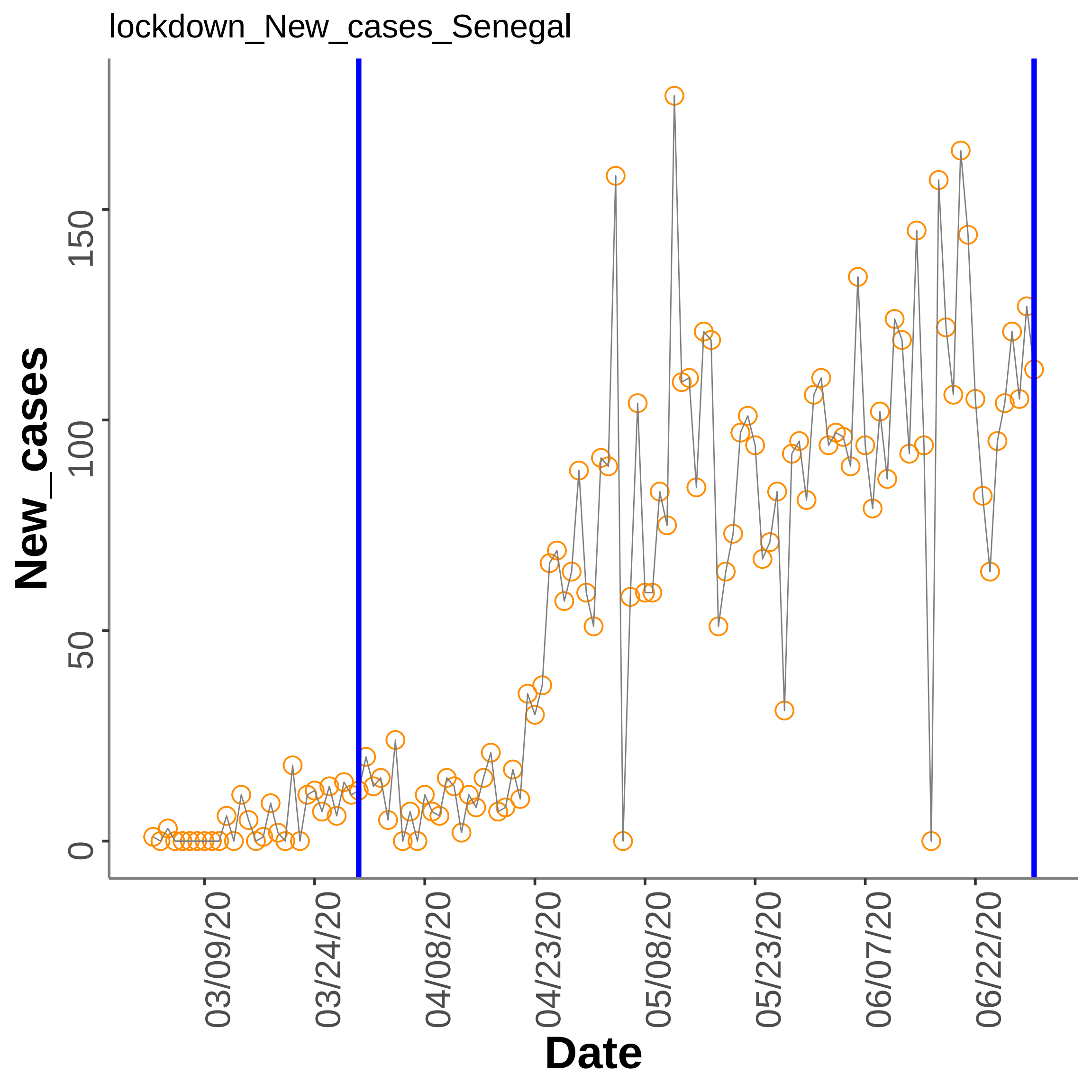
lockdown_New_cases_Senegal
New_cases
0
50
100
150
Date
03/09/20
03/24/20
04/08/20
04/23/20
05/08/20
05/23/20
06/07/20
06/22/20

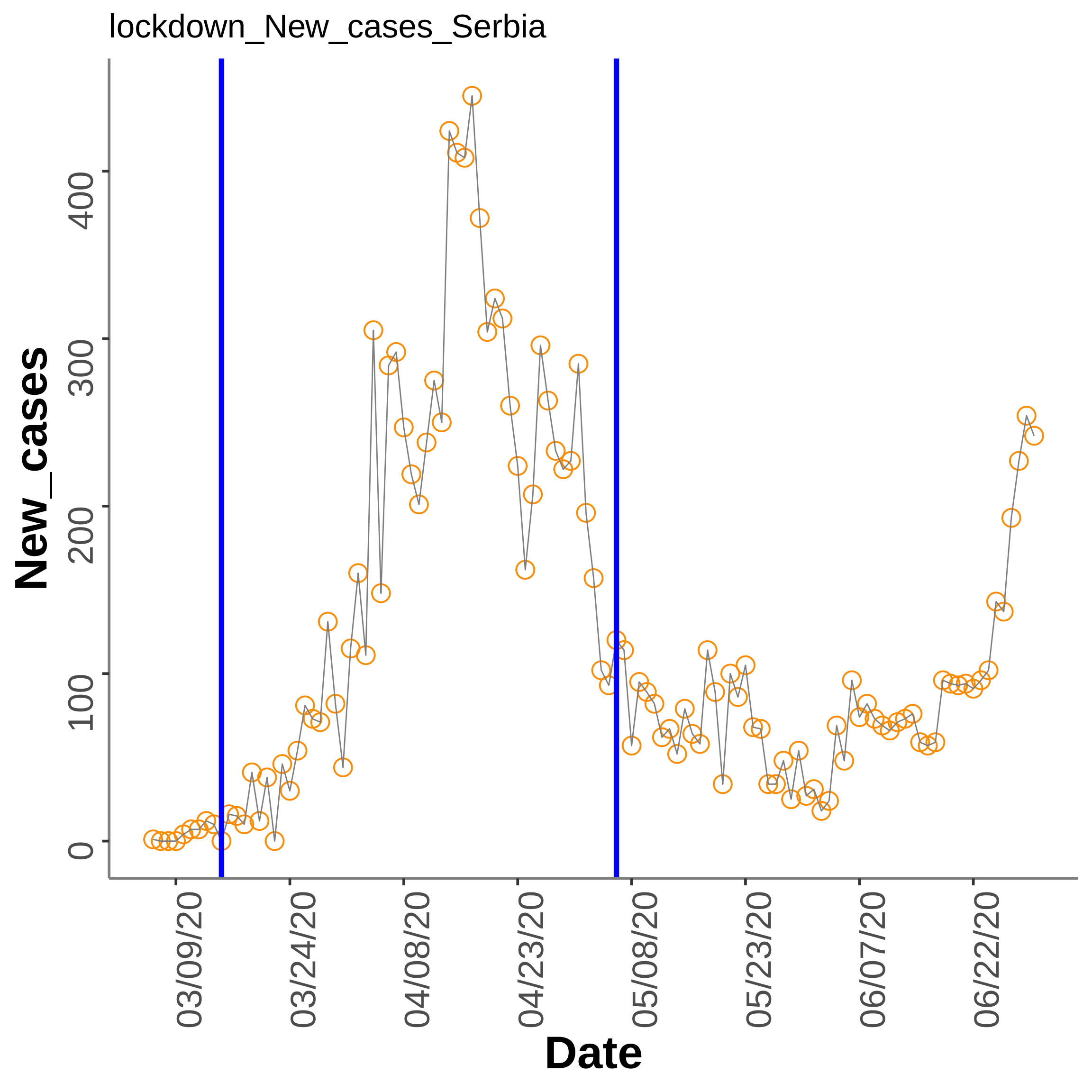

lockdown_New_cases_Serbia
New_cases
0
100
200
300
400
Date
03/09/20
03/24/20
04/08/20
04/23/20
05/08/20
05/23/20
06/07/20
06/22/20

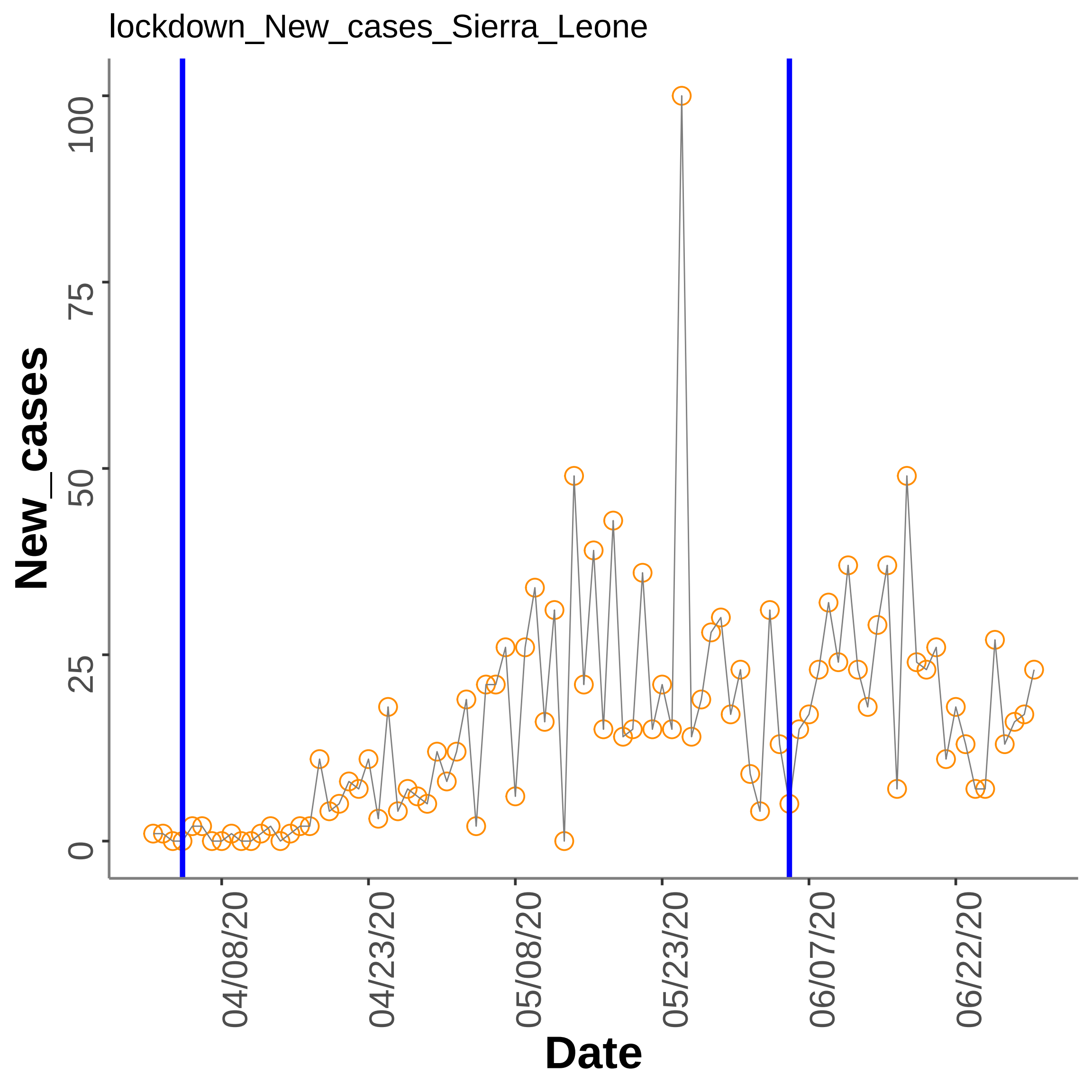

lockdown_New_cases_Sierra_Leone
New_cases
0
25
50
75
100
04/08/20
04/23/20
05/08/20
05/23/20
06/07/20
06/22/20
Date

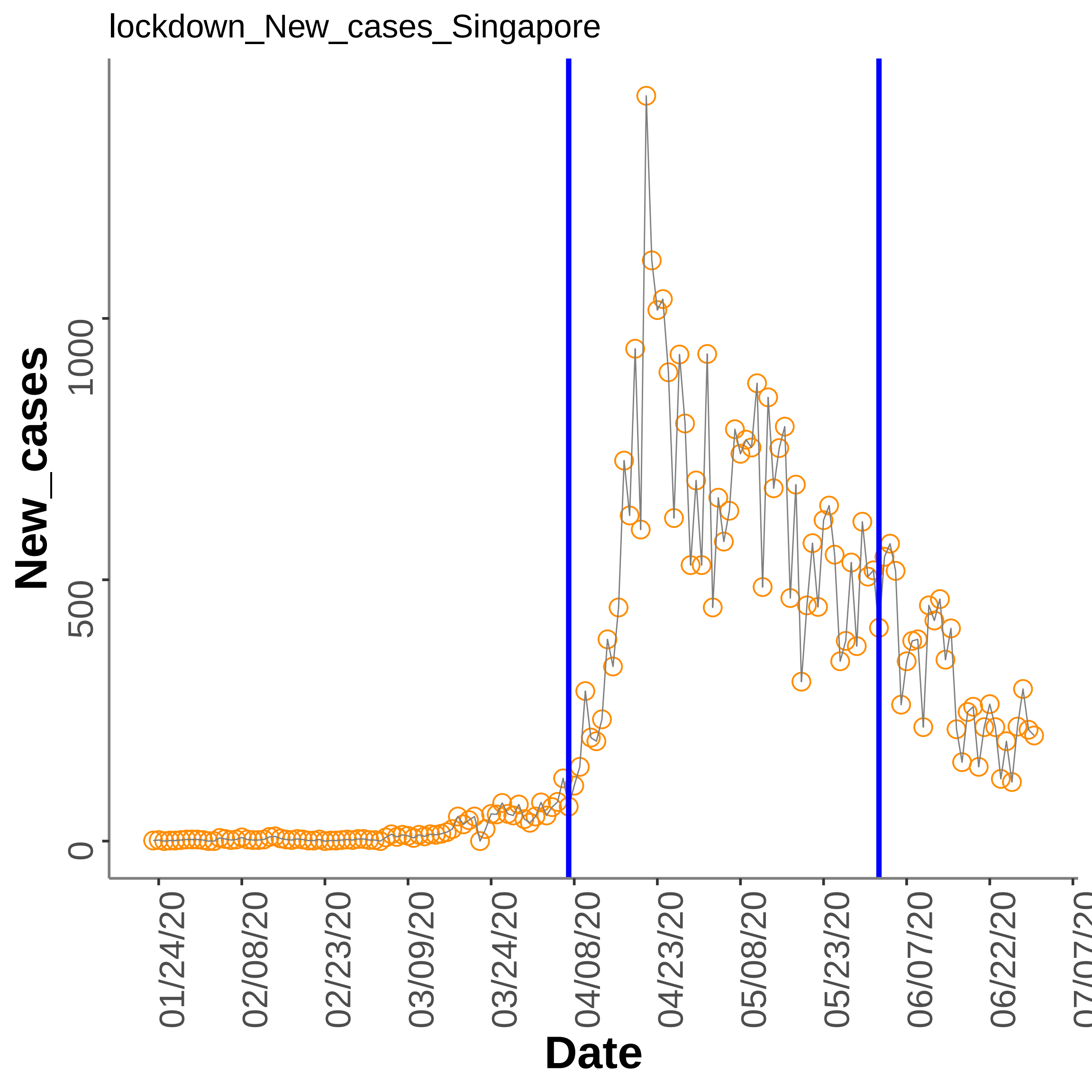

lockdown_New_cases_Singapore
New_cases
0
500
1000
Date
01/24/20
02/08/20
02/23/20
03/09/20
03/24/20
04/08/20
04/23/20
05/08/20
05/23/20
06/07/20
06/22/20
07/07/20

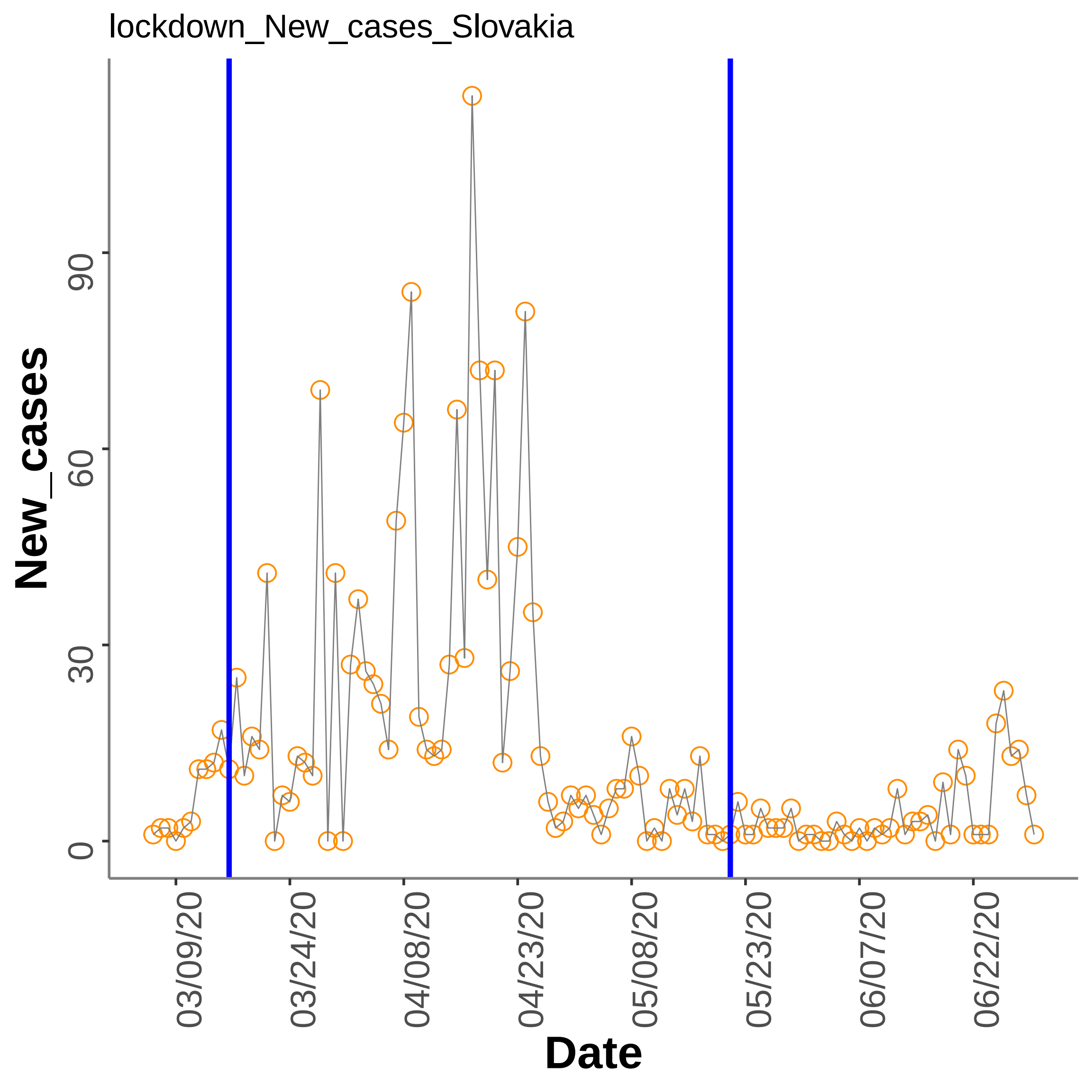

lockdown_New_cases_Slovakia
New_cases
0
30
60
90
Date
03/09/20
03/24/20
04/08/20
04/23/20
05/08/20
05/23/20
06/07/20
06/22/20

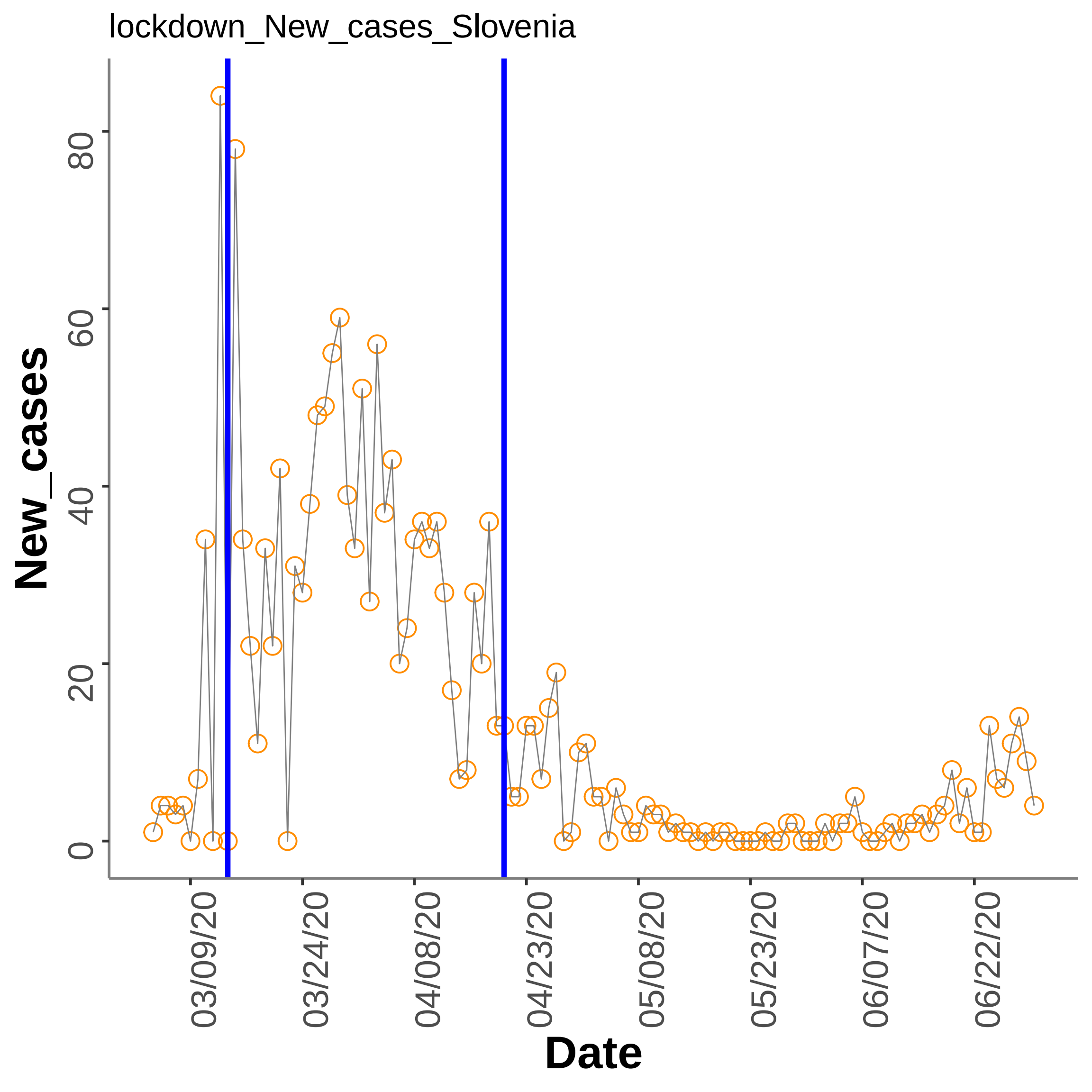

lockdown_New_cases_Slovenia
New_cases
0
20
40
60
80
Date
03/09/20
03/24/20
04/08/20
04/23/20
05/08/20
05/23/20
06/07/20
06/22/20

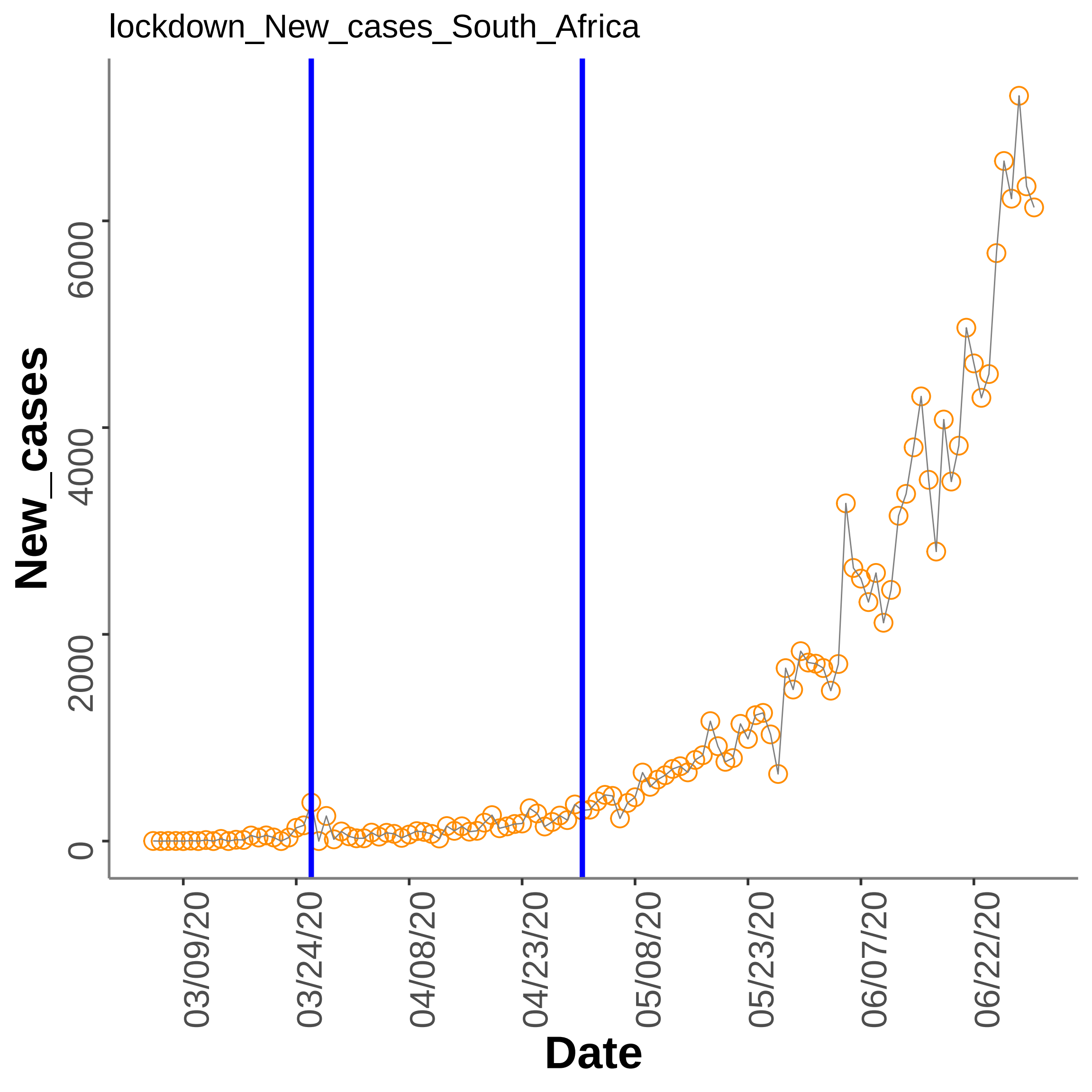

lockdown_New_cases_South_Africa
New_cases
0
2000
4000
6000
Date
03/09/20
03/24/20
04/08/20
04/23/20
05/08/20
05/23/20
06/07/20
06/22/20

lockdown_New_cases_Spain

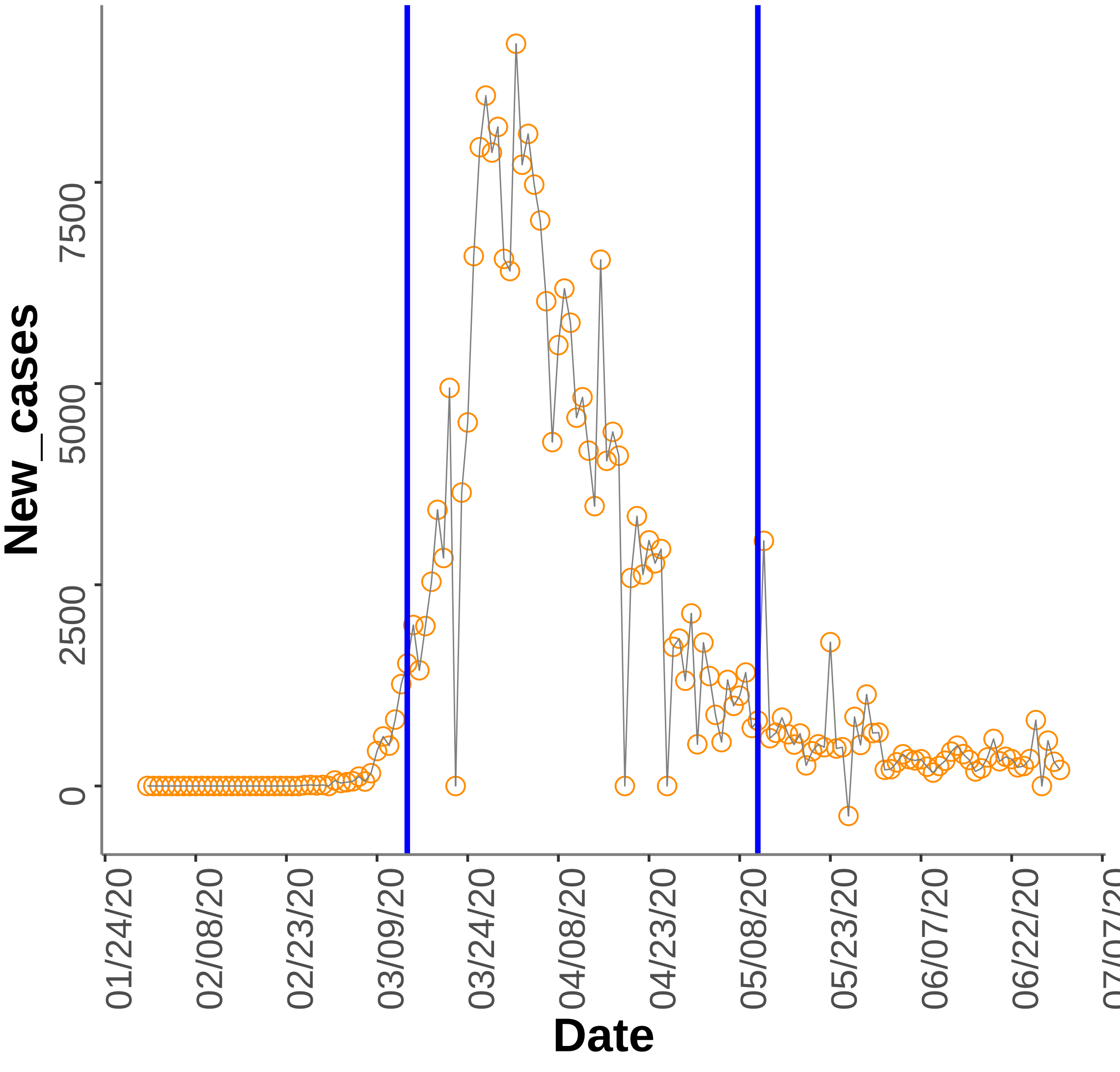

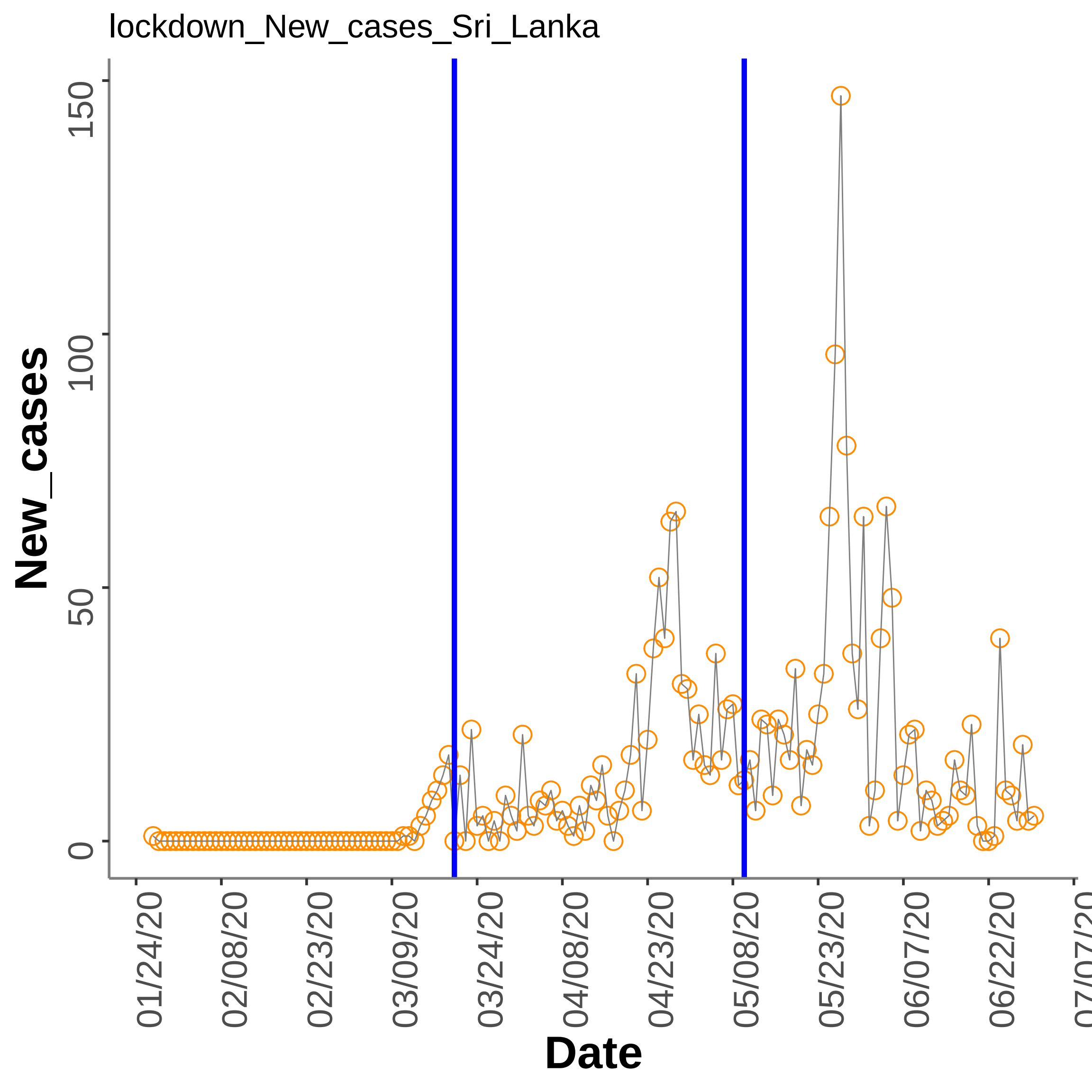

lockdown_New_cases_Sri_Lanka
New_cases
0
50
100
150
01/24/20
02/08/20
02/23/20
03/09/20
03/24/20
04/08/20
04/23/20
05/08/20
05/23/20
06/07/20
06/22/20
07/07/20
Date

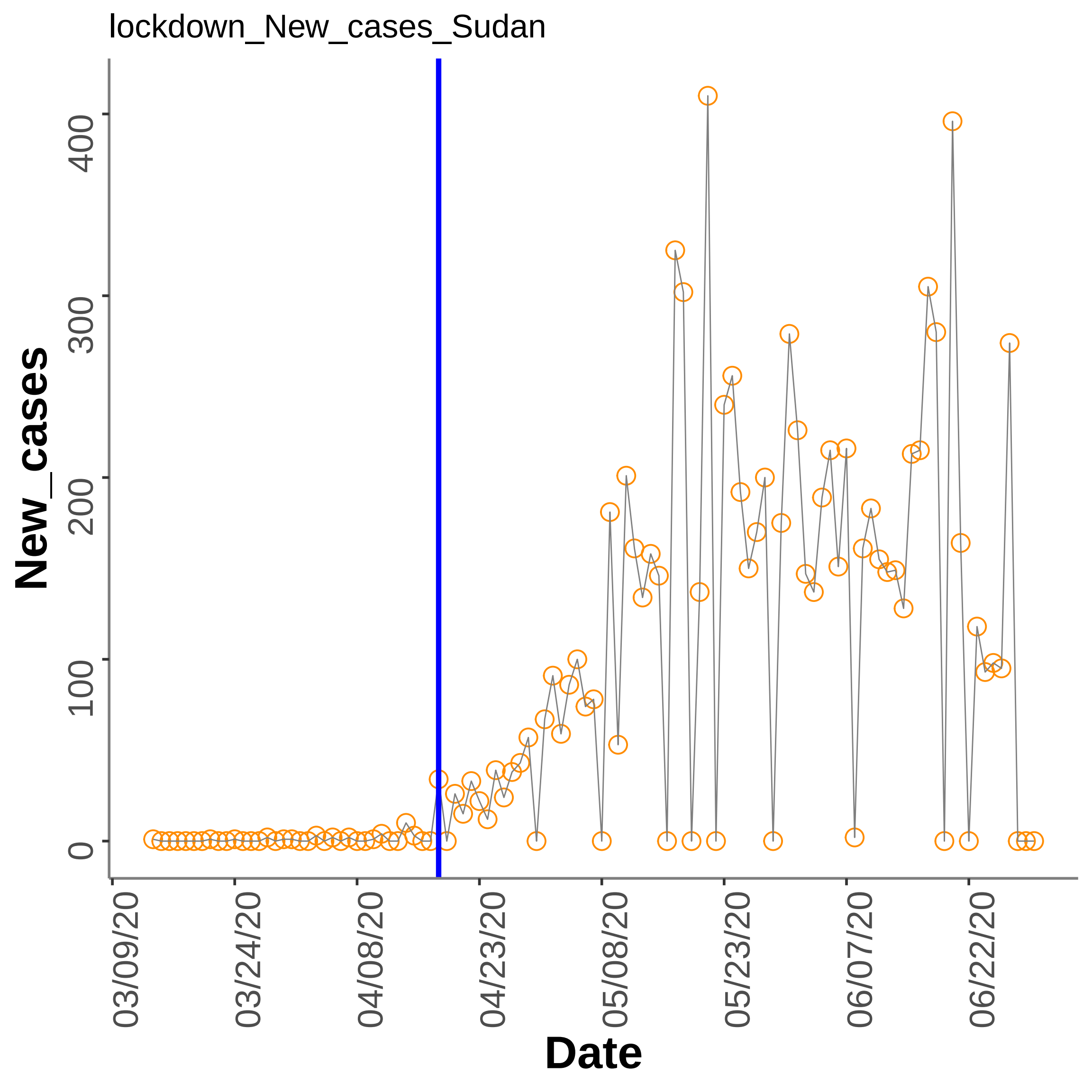
lockdown_New_cases_Sudan
New_cases
0
100
200
300
400
Date
03/09/20
03/24/20
04/08/20
04/23/20
05/08/20
05/23/20
06/07/20
06/22/20

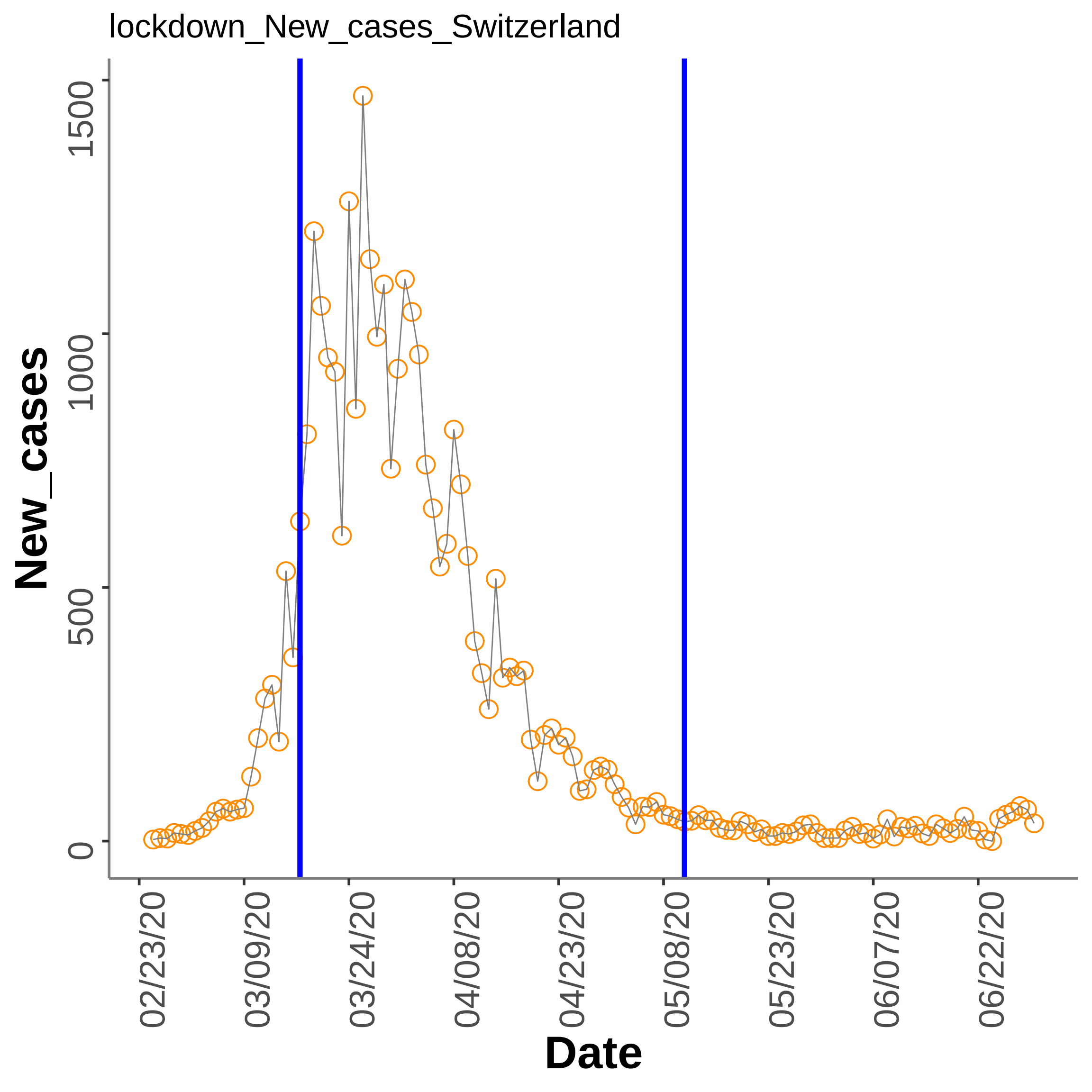

lockdown_New_cases_Switzerland
New_cases
0
500
1000
1500
02/23/20
03/09/20
03/24/20
04/08/20
04/23/20
05/08/20
05/23/20
06/07/20
06/22/20
Date

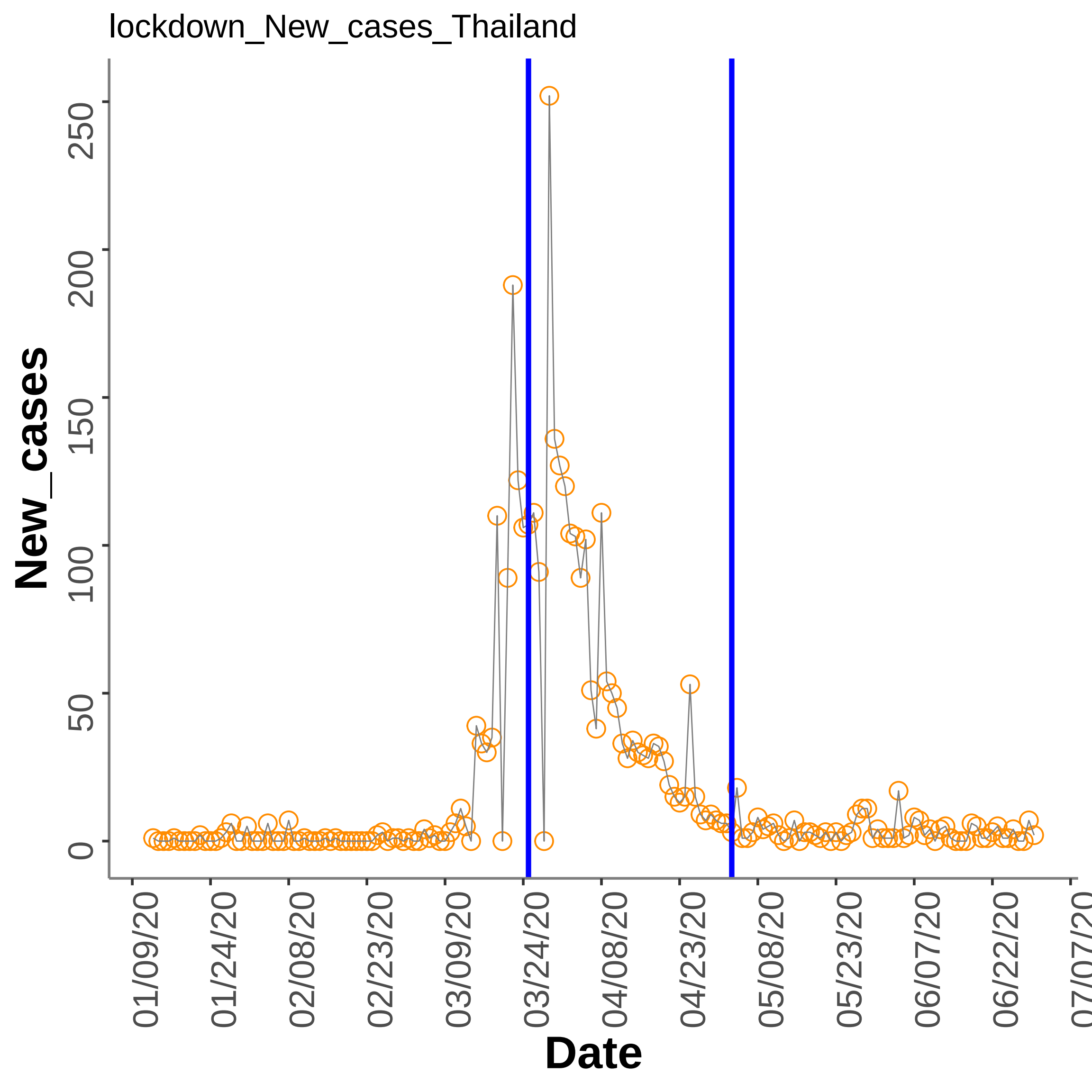
lockdown_New_cases_Thailand
New_cases
0
50
100
150
200
250
01/09/20
01/24/20
02/08/20
02/23/20
03/09/20
03/24/20
04/08/20
04/23/20
05/08/20
05/23/20
06/07/20
06/22/20
07/07/20
Date

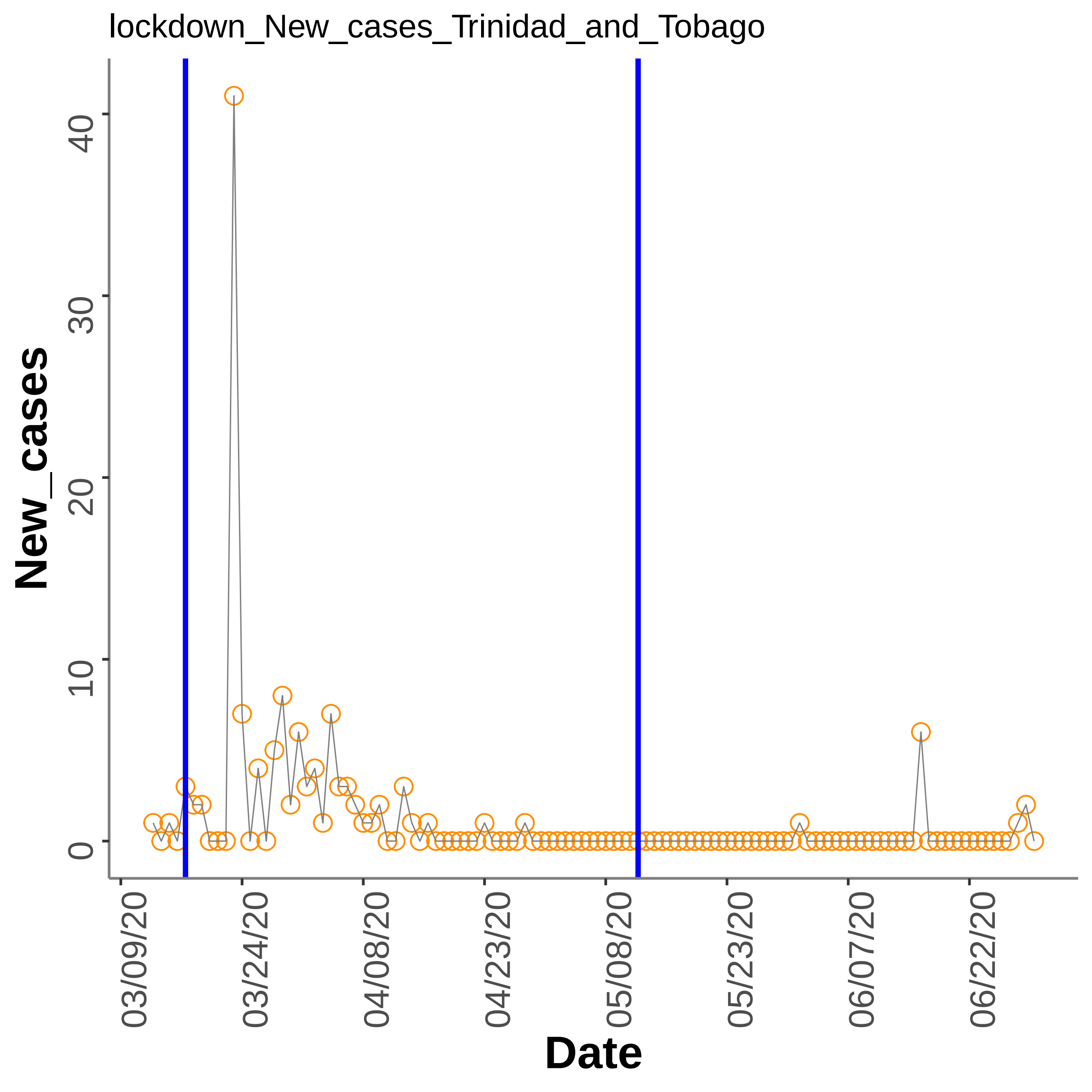
lockdown_New_cases_Trinidad_and_Tobago
New_cases
0
10
20
30
40
Date
03/09/20
03/24/20
04/08/20
04/23/20
05/08/20
05/23/20
06/07/20
06/22/20

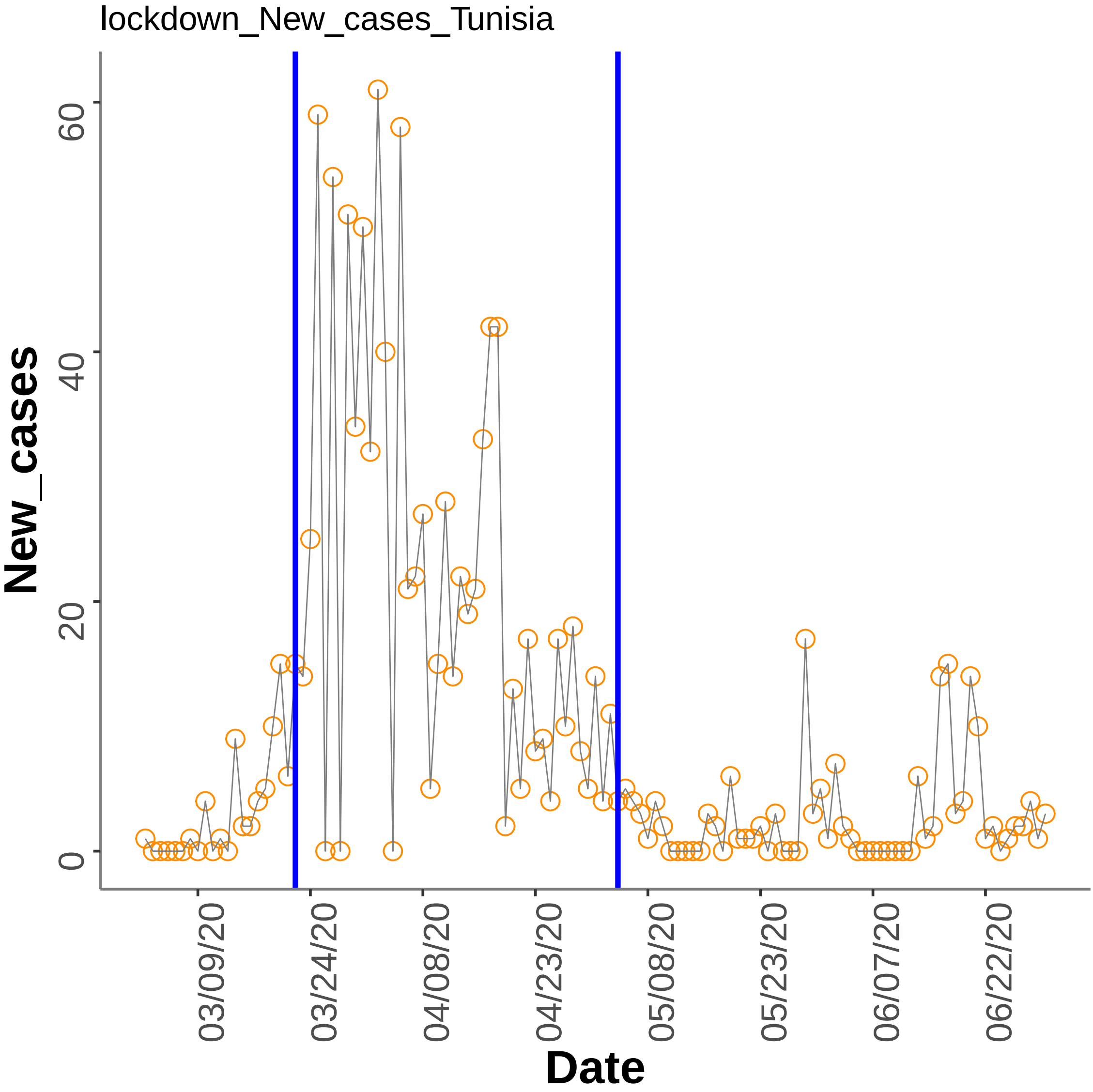
lockdown_New_cases_Tunisia
New_cases
Date
0
20
40
60
03/09/20
03/24/20
04/08/20
04/23/20
05/08/20
05/23/20
06/07/20
06/22/20

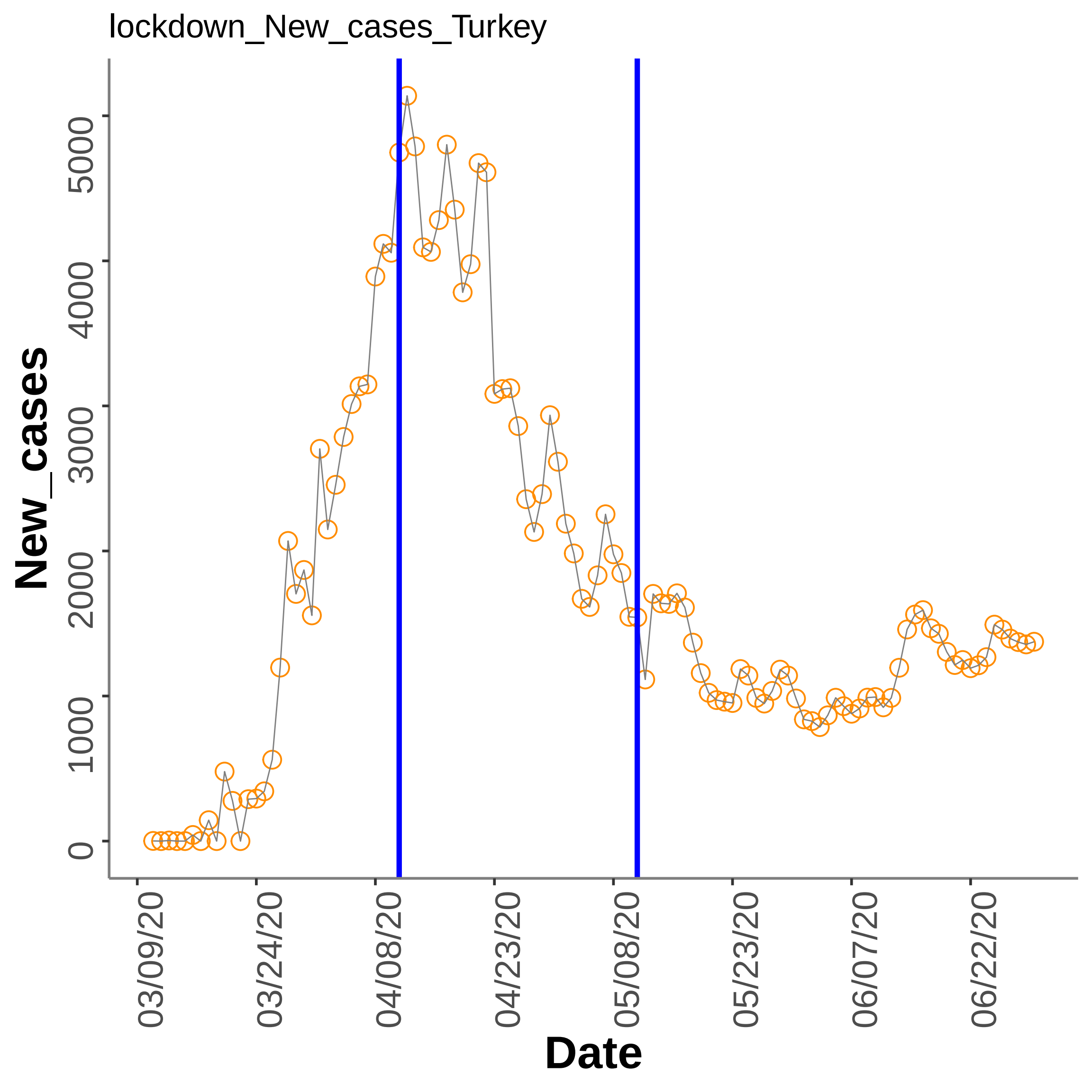

lockdown_New_cases_Turkey
New_cases
0
1000
2000
3000
4000
5000
Date
03/09/20
03/24/20
04/08/20
04/23/20
05/08/20
05/23/20
06/07/20
06/22/20

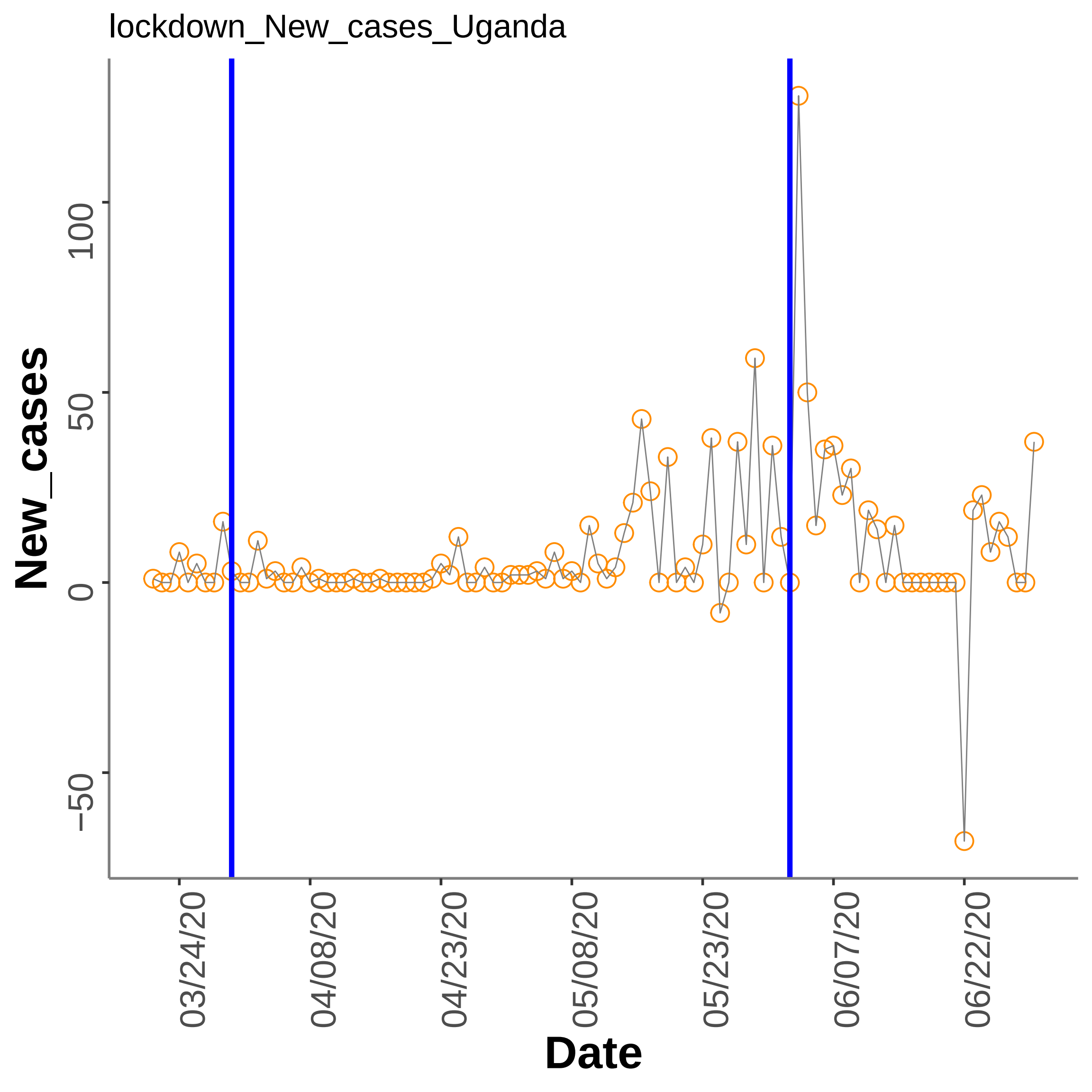

lockdown_New_cases_Uganda
New_cases
100
50
0
−50
03/24/20
04/08/20
04/23/20
05/08/20
05/23/20
06/07/20
06/22/20
Date

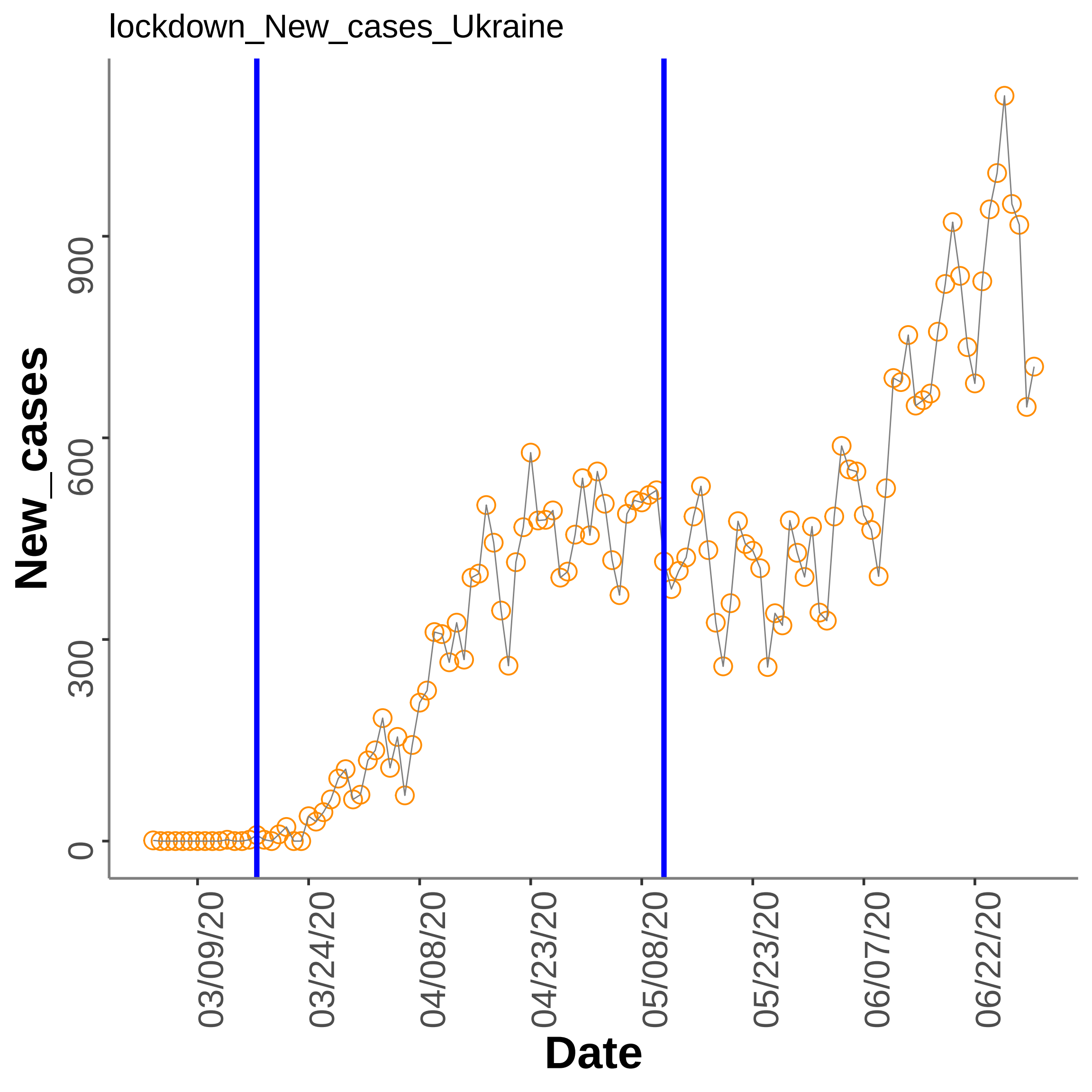

lockdown_New_cases_Ukraine
New_cases
0
300
600
900
03/09/20
03/24/20
04/08/20
04/23/20
05/08/20
05/23/20
06/07/20
06/22/20
Date

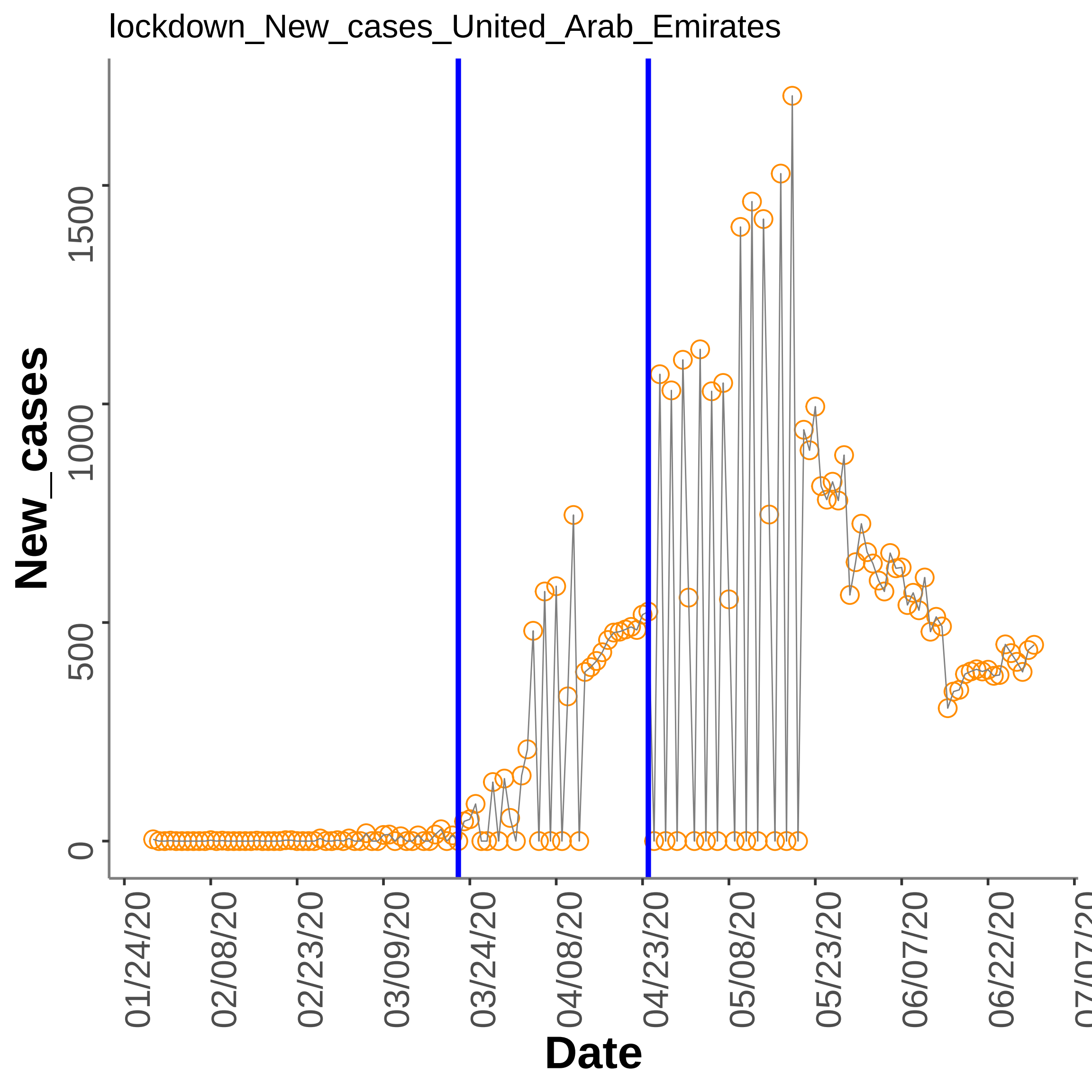
lockdown_New_cases_United_Arab_Emirates
New_cases
0
500
1000
1500
01/24/20
02/08/20
02/23/20
03/09/20
03/24/20
04/08/20
04/23/20
05/08/20
05/23/20
06/07/20
06/22/20
07/07/20
Date

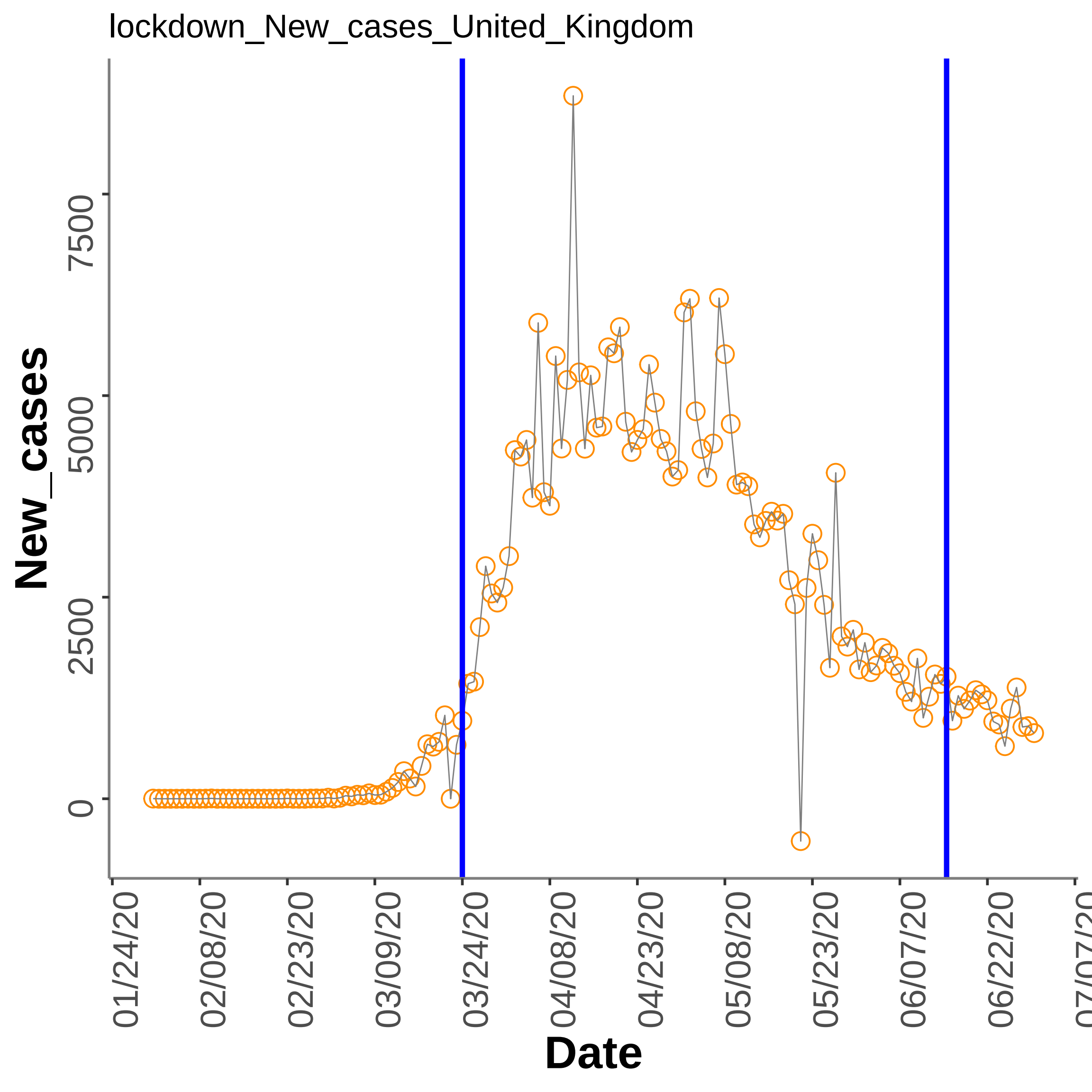

lockdown_New_cases_United_Kingdom
New_cases
7500
5000
2500
0
01/24/20
02/08/20
02/23/20
03/09/20
03/24/20
04/08/20
04/23/20
05/08/20
05/23/20
06/07/20
06/22/20
Date

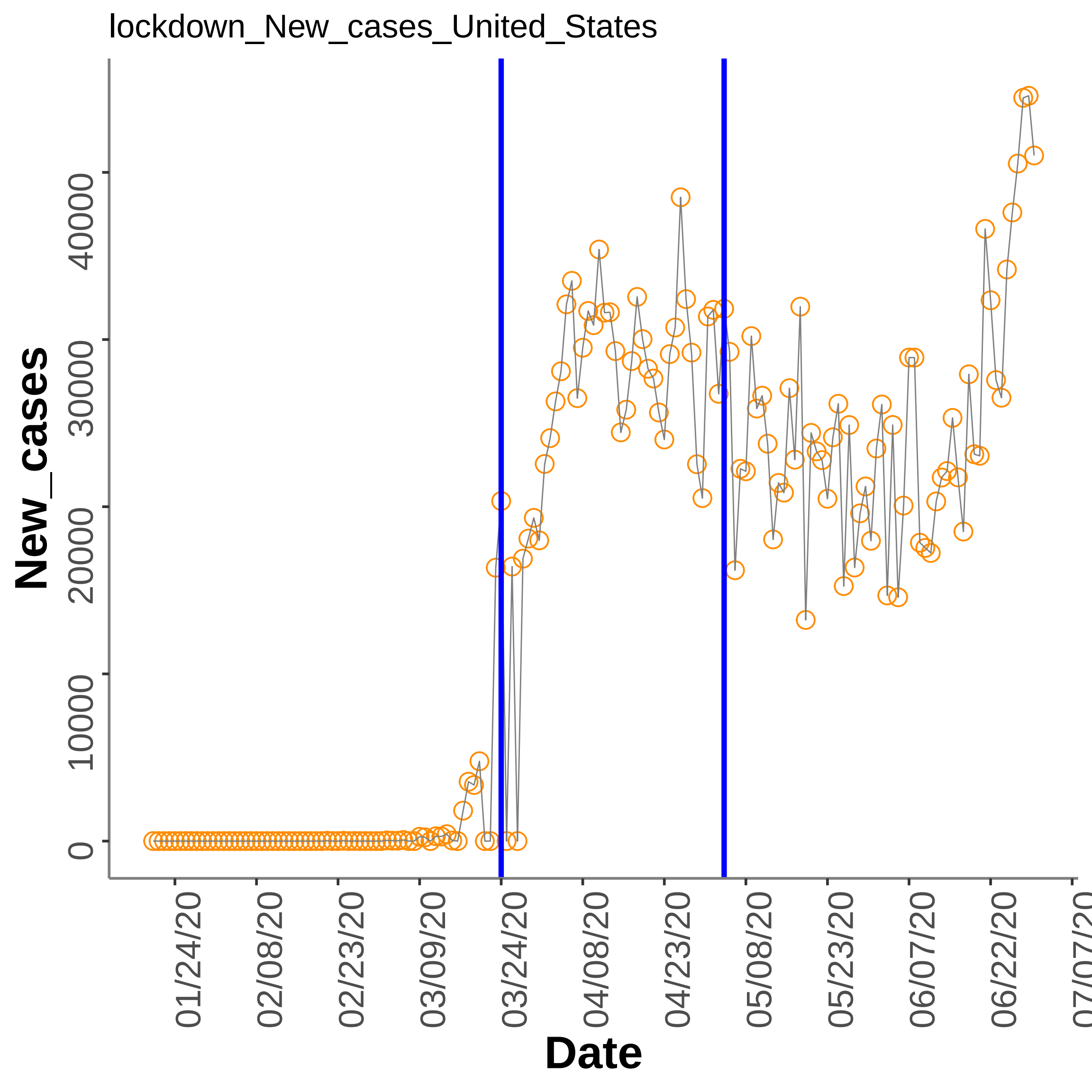
lockdown_New_cases_United_States
New_cases
0
10000
20000
30000
40000
Date
01/24/20
02/08/20
02/23/20
03/09/20
03/24/20
04/08/20
04/23/20
05/08/20
05/23/20
06/07/20
06/22/20
07/07/20

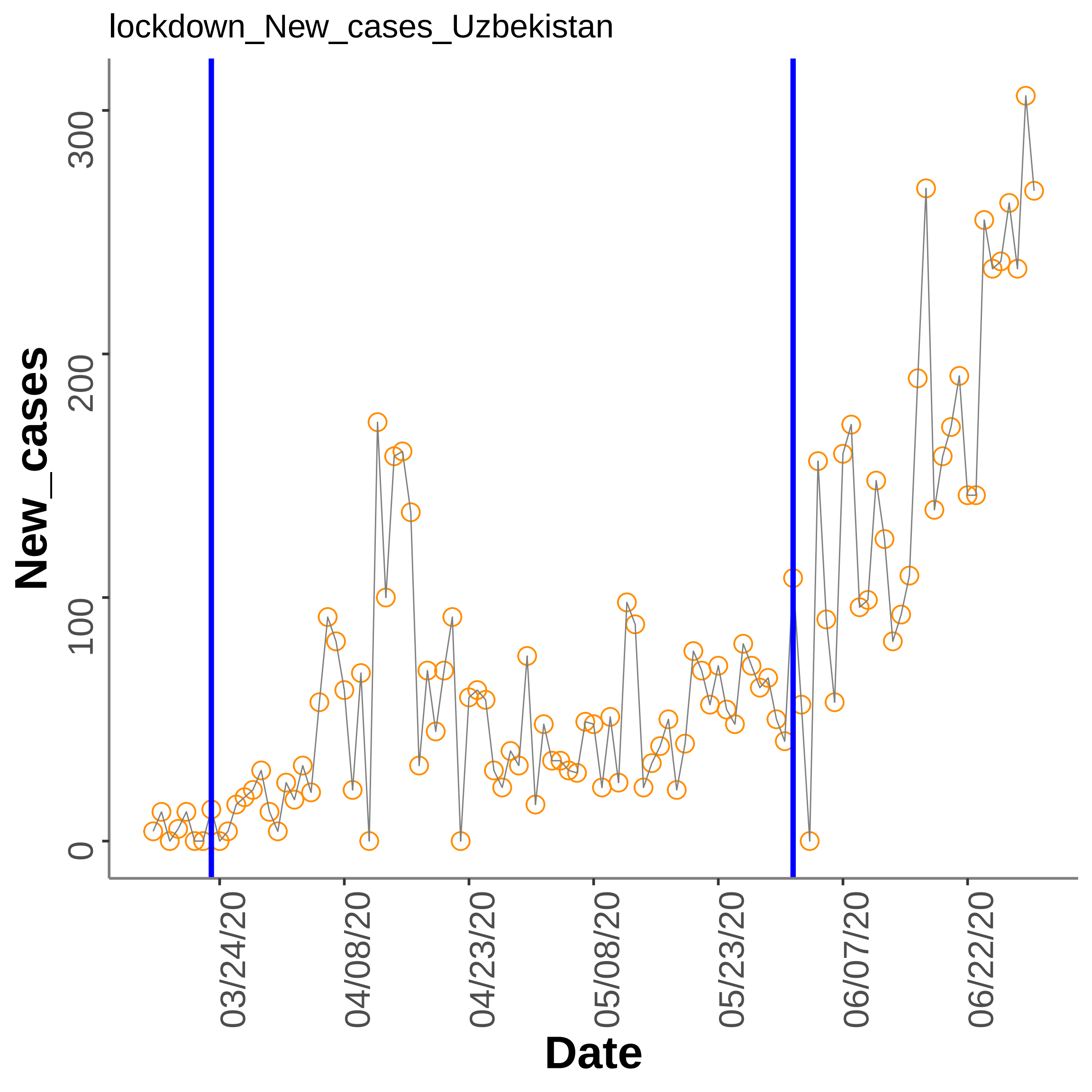
lockdown_New_cases_Uzbekistan
New_cases
0
100
200
300
03/24/20
04/08/20
04/23/20
05/08/20
05/23/20
06/07/20
06/22/20
Date

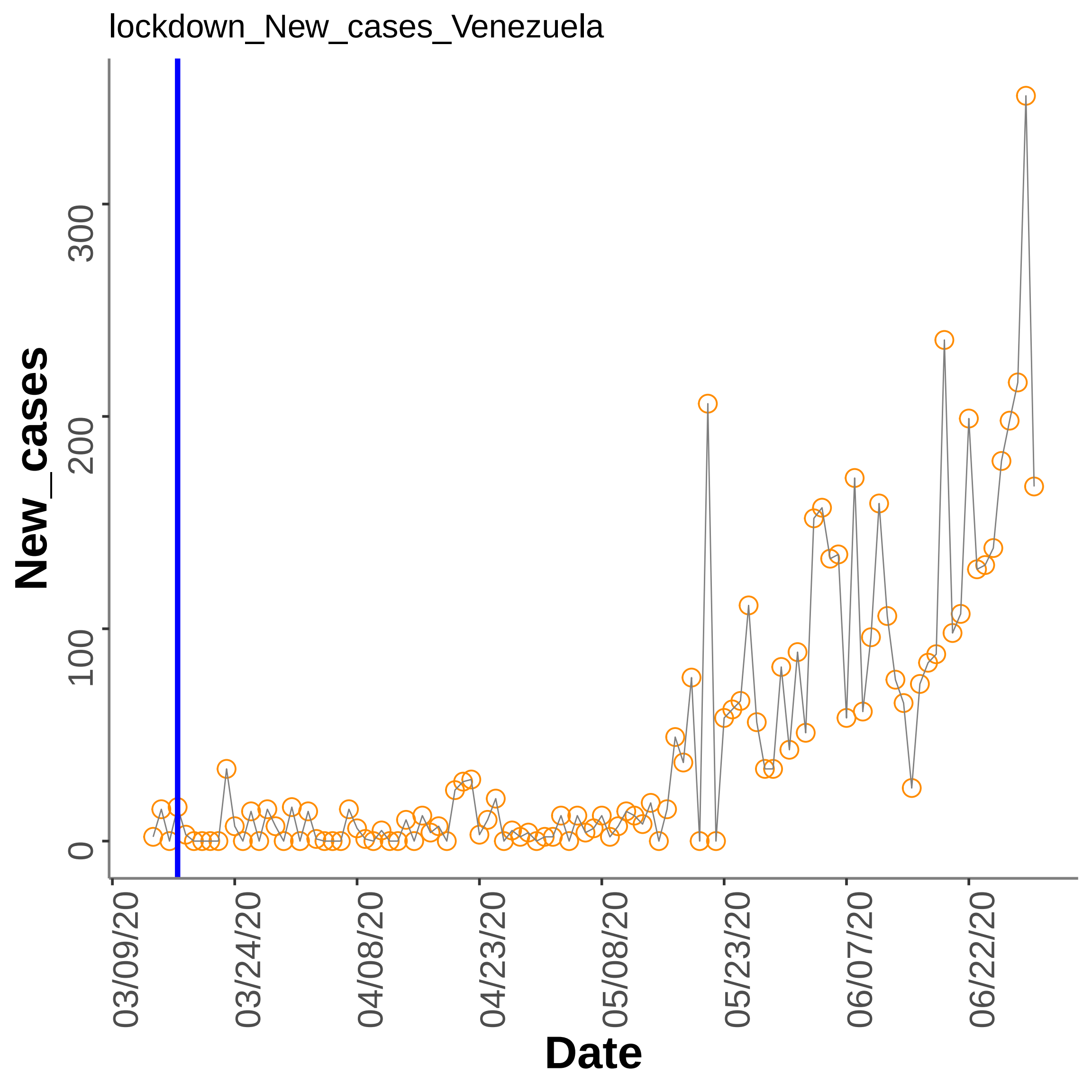
lockdown_New_cases_Venezuela
New_cases
0
100
200
300
Date
03/09/20
03/24/20
04/08/20
04/23/20
05/08/20
05/23/20
06/07/20
06/22/20

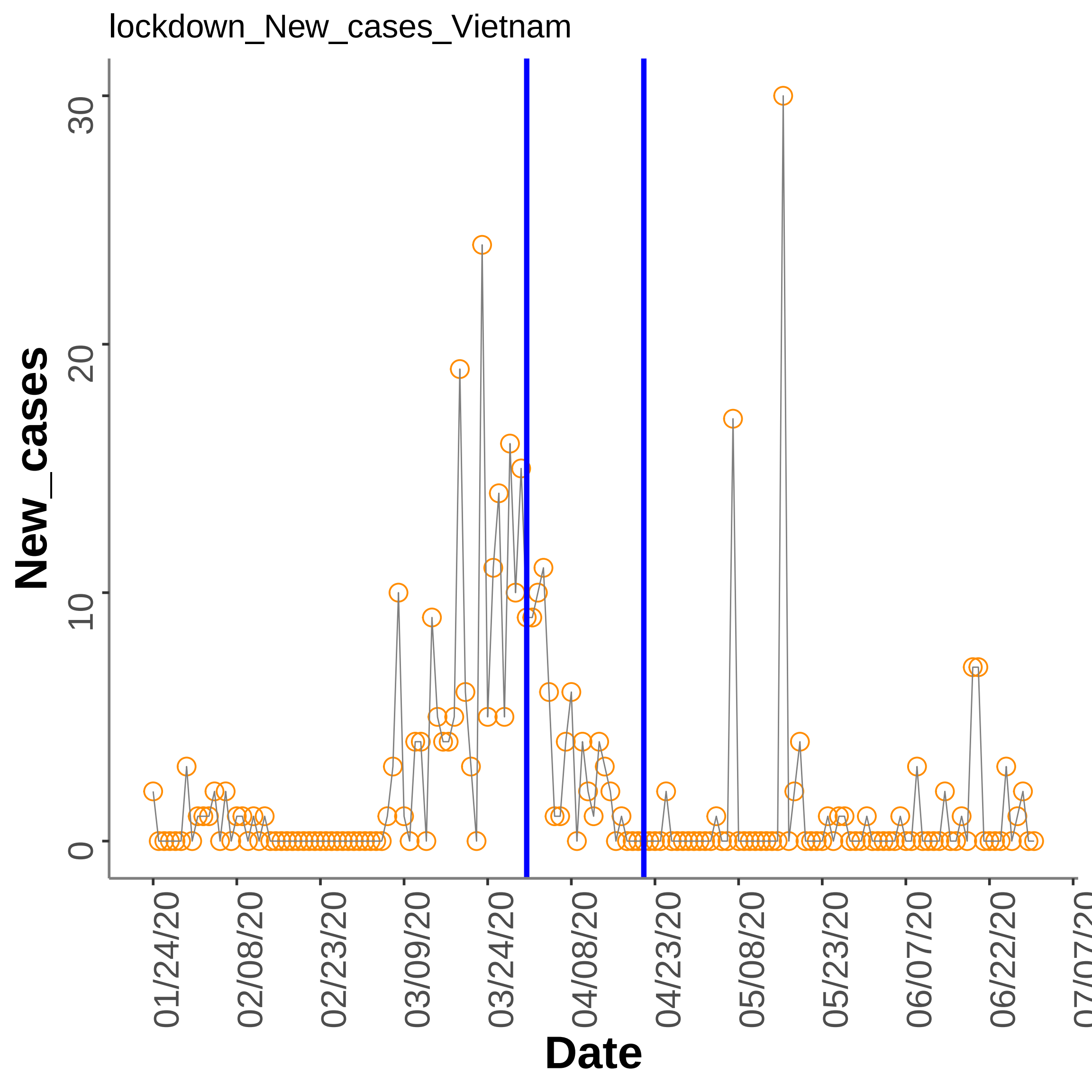

lockdown_New_cases_Vietnam
New_cases
30
20
10
0
Date
01/24/20
02/08/20
02/23/20
03/09/20
03/24/20
04/08/20
04/23/20
05/08/20
05/23/20
06/07/20
06/22/20
07/07/20

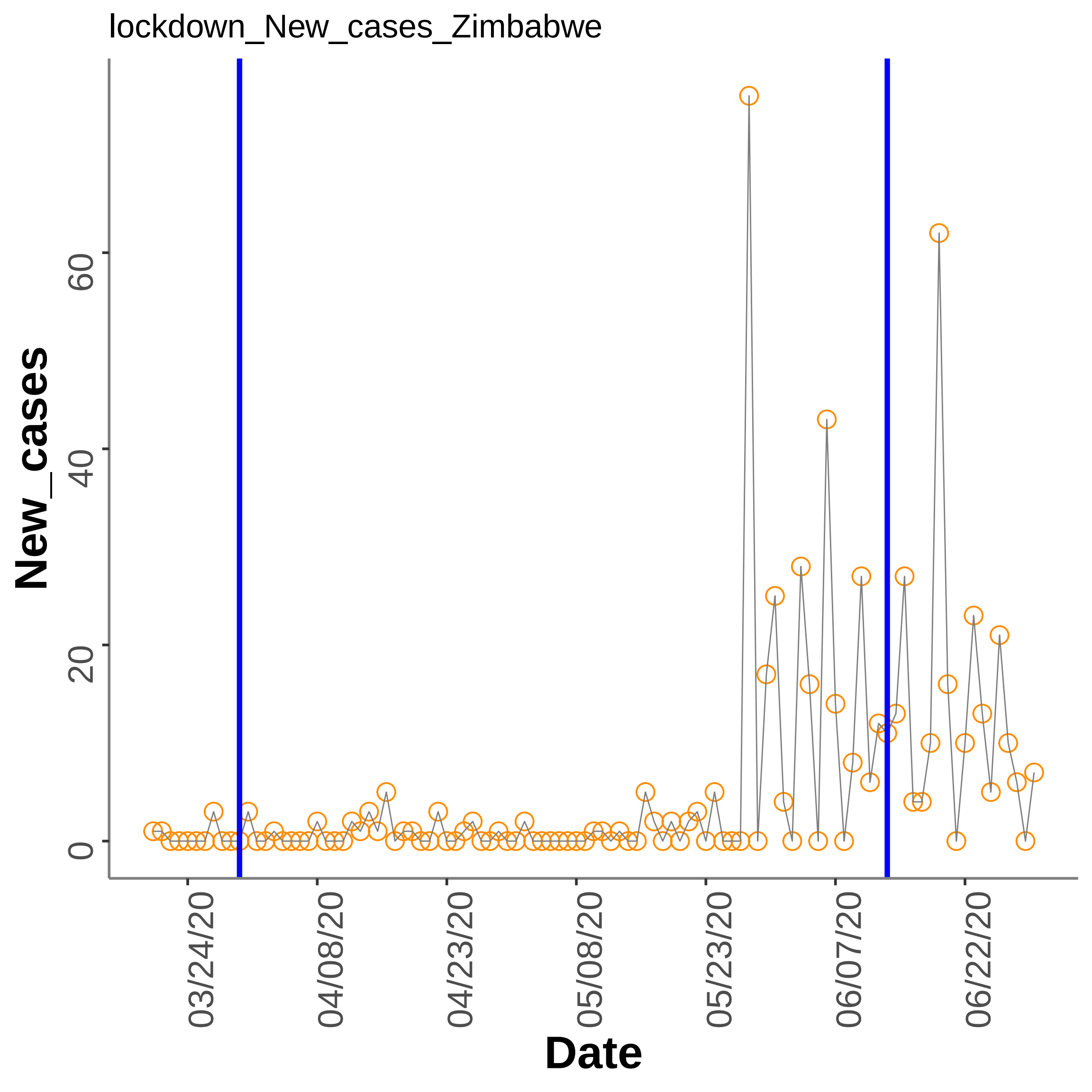

lockdown_New_cases_Zimbabwe
New_cases
0
20
40
60
03/24/20
04/08/20
04/23/20
05/08/20
05/23/20
06/07/20
06/22/20
Date
